# Design of siRNA molecules for silencing of membrane glycoprotein, nucleocapsid phosphoprotein, and surface glycoprotein genes of SARS-CoV2

**DOI:** 10.1186/s43141-022-00346-z

**Published:** 2022-04-28

**Authors:** Vijaya Sai Ayyagari

**Affiliations:** Department of Biotechnology, Vignan’s Foundation for Science, Technology & Research (Deemed to be University), Guntur District, Vadlamudi, Andhra Pradesh 522 213 India

**Keywords:** SARS-CoV2, COVID-19, siRNA design tools, siRNA, Membrane glycoprotein, Nucleocapsid phosphoprotein, Surface glycoprotein

## Abstract

The global COVID-19 pandemic caused by SARS-CoV2 infected millions of people and resulted in more than 4 million deaths worldwide. Apart from vaccines and drugs, RNA silencing is a novel approach for treating COVID-19. In the present study, siRNAs were designed for the conserved regions targeting three structural genes, M, N, and S, from forty whole-genome sequences of SARS-CoV2 using four different software, RNAxs, siDirect, *i-Score* Designer, and OligoWalk. Only siRNAs which were predicted in common by all the four servers were considered for further shortlisting. A multistep filtering approach has been adopted in the present study for the final selection of siRNAs by the usage of different online tools, viz., siRNA scales, MaxExpect, DuplexFold, and SMEpred. All these web-based tools consider several important parameters for designing functional siRNAs, e.g., target-site accessibility, duplex stability, position-specific nucleotide preference, inhibitory score, thermodynamic parameters, GC content, and efficacy in cleaving the target. In addition, a few parameters like GC content and dG value of the entire siRNA were also considered for shortlisting of the siRNAs. Antisense strands were subjected to check for any off-target similarities using BLAST. Molecular docking was carried out to study the interactions of guide strands with AGO2 protein. A total of six functional siRNAs (two for each gene) have been finally selected for targeting M, N, and S genes of SARS-CoV2. The siRNAs have not shown any off-target effects, interacted with the domain(s) of AGO2 protein, and were efficacious in cleaving the target mRNA. However, the siRNAs designed in the present study need to be tested in vitro and in vivo in the future.

**Supplementary Information:**

The online version contains supplementary material available at 10.1186/s43141-022-00346-z.

## Background

The ongoing global coronavirus disease 2019 (COVID-19) pandemic caused by severe acute respiratory syndrome coronavirus-2 (SARS-CoV2) resulted in over 195 million confirmed cases with 4,180,161 deaths reported globally (https://covid19.who.int/ accessed on 29th July 2021, 16:31 IST) [1]. Coronavirus consists of a single-strand, positive-sense RNA, whose genome size ranges from 27 to 30 kilobases [1, 2]. Two-thirds of the genome of the coronavirus codes for nonstructural polyproteins 1ab. The remaining one-third of the genome of the coronavirus codes for structural proteins consisting of Envelope (E) protein, Membrane (M) glycoprotein, Surface (S) glycoprotein (S), and Nucleocapsid (N) phosphoprotein [1–3].

With the onset of the global COVID-19 pandemic, several research laboratories and biopharmaceutical companies have been actively engaged in the development of vaccines for COVID-19. A few vaccines have been approved and rolled out for public usage in this line. However, the emergence of new variants of SARS-CoV2 across the globe has resulted in the vaccines’ reduced efficacy [4]. Besides this, shortfalls in the supply of vaccines against the demand are another constraint [5]. In these cases, there is a need for the development of alternate strategies for treating the patients infected with SARS-CoV2 to ensure that a greater number of successful candidate molecules are available for combating COVID-19. One such measure is the development of short interfering RNA (siRNA) as an antiviral agent.

A messenger RNA (mRNA) is cleaved by a single-stranded RNA known as short interfering RNA (siRNA). The process is known as RNA interference (RNAi). In this process, a double-stranded RNA is cleaved by a ribonuclease III-like enzyme known as Dicer, resulting in the formation of a duplex consisting of 21–23 nucleotides. One strand is known as a sense (passenger) strand, and another is known as an antisense (guide) strand. Before the inhibition of target mRNA, the duplex is loaded into an RNA-induced silencing complex (RISC). Subsequently, the passenger strand is lost, and the guide strand pairs with the mRNA by complementary base pairing and results in cleavage of the target mRNA [6, 7].

To induce the silencing of the target mRNA, a rational design of siRNA is necessary. This is achieved by the usage of several online tools, viz. siDirect v2.0 [8], *i-Score* Designer [9], RNAxs [10], siRNA scales [11], siDRM [12], and siDESIGN Center [*https://horizondiscovery.com/en/ordering-and-calculation-tools/sidesign-center*]. For further reading, a comparative account of the first-generation and second-generation siRNA design rules was given in Liu et al. [13].

In the recent past, there are reports available on the in silico design of siRNAs against different human viruses, e.g., Zika virus [14–16], MERS-CoV [17–19], Influenza virus [20–22], human adenovirus type-3 [23], hepatitis C virus [24], rabies virus [25], and respiratory syncytial virus [26]. siRNAs designed in silico from a majority of the studies mentioned above were tested in vitro and were found to be effective [14, 17–22, 24]. A comprehensive review on the usage of siRNA for gene silencing of coronaviruses is given in Sajid et al. [4] and Uludag et al. [27]. Bioinformatics tools have also been utilized for the design of siRNAs targeting various regions in the genome of SARS-CoV2. e.g., leader sequence [28, 29], nucleocapsid phosphoprotein and surface glycoprotein [30], RdRp gene [31], and surface glycoprotein [32].

In the present study, functional siRNAs were designed in silico for targeting the structural genes, viz. M, N, and S in the genome of SARS-CoV2. This paves the way forward for further studies in vitro and in vivo for the shortlisting of the effective siRNA candidates designed in the present study for usage by humans for combating COVID-19.

## Methods

### Retrieval of nucleotide sequences from NCBI — nucleotide database

In the present study, forty whole-genome sequences of SARS-CoV2 were retrieved from the nucleotide database of the National Centre for Biotechnology Information (NCBI) (https://www.ncbi.nlm.nih.gov/nucleotide/) (Table 1). Nucleotide sequences corresponding to M, N, and S genes were used in the present study as targets for the design of potential siRNA.

**Table 1:** List of accession numbers of the sequences and their country of origin retrieved from nucleotide database of NCBI for the design of siRNAs

Sr. no.	Accession	Country of origin
1	MW425837	India
2	MW425563	India
3	MT758436	India
4	MT954922	India
5	MZ336032	India
6	OK356470	India
7	OK356467	India
8	OK189653	India
9	OL456172	India
10	MZ318159	India
11	MZ292150	India
12	MT339041	United States of America (USA)
13	OK375092	USA
14	MN985325	USA
15	MW738481	USA
16	MZ211240	USA
17	OK375091	USA
18	OL669603	USA
19	OL673374	USA
20	OL688470	USA
21	OL688471	USA
22	OK375082	USA
23	MT259226	China: Wuhan
24	OL664038	China: Guangzhou
25	OL663925	China: Guangzhou
26	MT066156	Italy
27	MT292577	Spain
28	MW156805	Australia
29	MW531680	Bangladesh
30	LC650048	Tokyo
31	LC650055	Tokyo
32	LC650057	Tokyo
33	LC650052	Tokyo
34	OK091660	South Africa
35	MZ345001	South Africa
36	MZ344999	South Africa
37	MZ362445	Italy
38	MZ362440	Italy
39	MZ362451	Italy
40	MZ362449	Italy

### Multiple sequence alignment

Multiple sequence alignment of the respective gene sequences was carried out using Clustal Omega (https://www.ebi.ac.uk/Tools/msa/clustalo/) for the identification of the conserved gene sequences.

Nucleotide sequences of the forty accessions corresponding to a particular gene were aligned using Clustal Omega. After alignment, stretches of nucleotide sequences without mismatches were identified and considered as conserved regions. The conserved regions were represented by an asterisk in the output obtained from the Clustal Omega. These regions were used for the design of siRNAs (Supplementary Tables 1, 2 and 3). Those conserved regions which were less than 73 nucleotides long were not considered for further analyses.

### Tools used for the design and validation of siRNAs

In the present study, four different online tools, RNAxs (http://rna.tbi.univie.ac.at/cgi-bin/RNAxs/RNAxs.cgi), siDirect v2.0 (http://sidirect2.rnai.jp/), *i-Score* Designer (https://www.med.nagoya-u.ac.jp/neurogenetics/i_Score/i_score.html), and OligoWalk (http://rna.urmc.rochester.edu/cgi-bin/server_exe/oligowalk/oligowalk_form.cgi) [7, 33], were used for the design of functional siRNAs.

#### Design of siRNAs by RNAxs

RNAxs was utilized in the present study for the design of functional siRNAs at default parameters (8nt (seed) accessibility threshold, 0.01157; 16nt accessibility threshold, 0.001002; self-folding energy, 0.9022; sequence asymmetry, 0.5; energy asymmetry, 0.4655; free end, 0.625; custom sequence rules, NN).

#### Design of siRNAs by siDirect

The algorithm of siDirect v2.0 selects functional siRNAs which follow the first three out of four rules specified by Ui-Tei et al. [34]. Apart from this, there is an option to select siRNAs by choosing the two other algorithms, viz. Reynolds et al. [35] and Amarzguioui and Prydz [6]. In the present study, the combined rule “Ui-Tei (U) + Reynolds (R) + Amarzguioui (A)” was chosen for the selection of siRNAs. Furthermore, the seed-duplex stability (T_m_) value was set to 21.5 °C.

#### Design of siRNAs by i-score designer

*i-Score* Designer was used in the present study for the prediction of active siRNAs. It works on an algorithm “*i-Score*” (inhibitory score) based on a linear regression model [9]. The conserved region sequences corresponding to the target genes of SARS-CoV2 were given as an input to *i-Score* Designer. In the *i-Score* designer, siRNAs were ranked according to *i-Score* which ranges from 100 to 0. Apart from calculating the *i-Score* and rank, the *i-Score* designer also calculates (i) ΔG value of the secondary structure of a siRNA strand, (ii) dinucleotide ΔG value at 5′ and 3′ ends, (iii) ΔG value for the entire siRNA (iv) number of GC stretches (v) % GC content (vi) scores of Ui-Tei, Amarzguioui, Hsieh, Takasaki, Reynolds, and Katoh, and (vii) scores and ranks of s-Biopredsi and DSIR.

#### Design of siRNAs by OligoWalk

OligoWalk online server [33] was utilized in the present study for predicting the efficient siRNAs at default parameters. The output of the program displays the predicted siRNAs along with the values for “probability of being an efficient siRNA” for each of the siRNAs. Besides probability values, the program also gives energy values (kcal/mol) for various parameters, viz. overall, duplex, break target, intraoligo, interoligo, and end_diff, besides T_m_ — dup (in °C) and prefilter score. siRNAs predicted by OligoWalk have an efficiency of 78.6 % in silencing the target [33].

#### siRNA scales

siRNA scales web server available at http://gesteland.genetics.utah.edu/siRNA_scales/ was utilized for the screening of the siRNAs predicted by the four tools, viz. RNAxs, siDirect, *i-Score* Designer, and OligoWalk. The antisense strand sequences were given as an input to the siRNA scales. Those siRNAs whose predicted value of efficacy ranged from 1 to ≤ 30 were considered for further analyses.

#### Calculation of the free energy of folding of the guide strand

MaxExpect web server (https://rna.urmc.rochester.edu/RNAstructureWeb/Servers/MaxExpect/MaxExpect.html) [36] was used for the prediction of the structure of the guide strand at default parameters (maximum % energy difference, 50; maximum number of structures, 1000; Windows size, 5; gamma, 1). The output of the program displays the structure of the RNA along with its score (also termed as energy). The cutoff value of the free energy of folding was set to 1.5 for shortlisting of the siRNAs in the present study [32].

#### Calculation of the free energy of binding between the guide strand and the target

DuplexFold web server (https://rna.urmc.rochester.edu/RNAstructureWeb/Servers/DuplexFold/DuplexFold.html) [37] was used for calculating the free energy of binding of the guide strand with the target at default parameters (maximum % energy difference, 5; maximum number of structures, 20; Window size, 0; maximum loop size, 30; temperature, 310.15 K). Guide-target duplexes with energy values ≤ −30 were shortlisted for further analyses [31, 32].

#### Validation of the efficacy of siRNAs

Validation of the efficacy of the predicted siRNA molecules against their consequence targets was carried out using SMEpred (https://bioinfo.imtech.res.in/manojk/smepred/) [38]. siRNAs (i.e., without any chemical modifications) with efficacy ≥ 85 were considered for shortlisting.

#### Off-target effects of siRNAs

BLAST® (Basic Local Alignment Search Tool) (https://blast.ncbi.nlm.nih.gov/Blast.cgi) was utilized to find out the off-target matches of the siRNAs designed in the present study. Reverse complement of the guide strand sequences was subjected for similarity check. BLAST® search was carried out with the following parameters: (i) choose search set — human genomic plus transcript (human G + T), models (XM/XP) excluded [28], and (ii) program selection — somewhat similar sequences (blastn). The remaining parameters were set to their default. Search parameters were automatically adjusted to search for a short input sequence by the program.

#### Molecular docking of the guide strand and argonaute protein

Molecular docking was carried out between the guide strands of siRNAs and human argonaute-2 protein using the HDOCK server (http://hdock.phys.hust.edu.cn/). In HDOCK, molecular docking is carried out between the ligand and receptor by using a fast Fourier transform (FFT)-based hierarchical approach [39]. The server builds the three-dimensional (3D) structure of the protein from a given amino acid sequence by searching for a homologous template in the Protein Data Bank (PDB). In the present study, the amino acid sequence of the human Argonaute-2 protein (AGO2) (PDB ID 4F3T) was retrieved from PDB and given as an input for building the 3D model of the protein for docking. Nucleotide sequences of the guide strand of siRNA molecules were given as an input (as “ligand”). The server generates the three-dimensional model of RNA from the given input nucleotide sequence.

The server returns with top 100 predictions along with docking scores, ligand root-mean square deviation (RMSD) values, ranks of the models, etc. The user can download the structures of the docked complexes besides the PDB files of receptor and ligand that were generated by homology modeling. The server also provides the homology quality scores of the receptor and ligand.

In the present study, the docked complexes that were ranked as one were considered for further analyses. The interacting residues of AGO protein with nucleotides of the guide strand of siRNA within 5 Å were reported.

### siRNA design workflow strategy

The combinatorial strategy for the design of functional siRNAs in the present study is summarized below:

(i) Identification of conserved regions across all the target genes by carrying out multiple sequence alignment
(ii) Design of siRNAs for the conserved regions of the three target genes by RNAxs at default parameters
(iii) Design of functional siRNAs by siDirect by considering the following parameters:
  i. Combined rule of the algorithm used for the functional siRNA selection: U + R + A
  ii. *T*_m_ = 21.5 °C (maximum)
(iv) Design of functional siRNAs by *i-Score* Designer and selection of siRNAs whose *i-Score* ≥ 65
(v) Design of functional siRNAs by OligoWalk online server
(vi) Pooling of siRNAs that were predicted in common by all the four online servers
(vii) Filtering of siRNAs obtained from step (vi) using siRNA scales at a cutoff value of ≤ 30
(viii) Shortlisting of siRNAs obtained from the above step whose dG ≥ −34.6 kcal/mol and GC content in the range from ≥ 31.6 to 53 %
(ix) Secondary structure prediction of siRNAs obtained from step (viii) by MaxExpect web server and further shortlisting of siRNAs whose free energy of folding ≥ 1.5
(x) Calculation of the free energy of binding between the antisense strand and the target region by DuplexFold server and further shortlisting of siRNAs whose energy is ≤ −30
(xi) Prediction of the efficacy of siRNAs in inhibiting the target mRNA obtained from the above step by SMEpred. A cutoff value of ≥ 85 was set for further shortlisting
(xii) Study of the off-target matches of siRNAs obtained from the preceding step using BLAST®
(xiii) Molecular docking of the shortlisted guide strands with AGO protein

## Results and discussion

In the present study, three structural genes M, N, and S that code for a membrane glycoprotein, nucleocapsid phosphoprotein, and surface glycoprotein, respectively, were used for the design of function siRNAs. This is the first study to screen most of the structural genes in the genome of SARS-CoV2 for the design of functional siRNAs. Apart from vaccines and drug candidates, target gene silencing by siRNA is an important approach for combating COVID-19. In the present study, four different online tools, viz. RNAxs, siDirect v2.0, *i-Score* Designer, and OligoWalk, were used for the design of functional siRNAs.

Accessibility of siRNA to the target site in mRNA is an essential prerequisite for RNAi. RNAxs, a web server, was designed by considering the target site accessibility as a criterion along with other known siRNA design rules for the prediction of highly functional siRNAs [10]. The number of siRNAs predicted by RNAxs and the range of worst rank (WR) for the three genes were as follows: (i) M — 60 (WR — 9 to 41), (ii) N — 145 (WR — 3 to 52), and (iii) S — 596 (WR — 0 to 74) (Supplementary Tables 4, 5 and 6).

The number of functional siRNAs predicted by siDirect (those satisfied the laid criteria) for the three genes was as follows: (i) M — 2, (ii) N — 28, and (iii) S — 240 (Supplementary Tables 7, 8 and 9). Only those siRNAs which have satisfied the rules that affect the siRNA activity as prescribed by the three functional siRNA selection algorithms [6, 34, 35] in any one of the following ways, viz. U, R, A, URA, UR, RA, and UA, were selected.

For the activity of siRNA, a near-perfect [8, 40, 41] complementary base pairing is required between the guide strand of the siRNA and the target mRNA. However, if the siRNA has complementarity with the nontarget region, it may result in silencing of the nontarget genes; especially, off-target silencing happens if the unintended regions base pair with the seed region of siRNA due to complementarity [8]. To overcome this effect, siDirect selects the guide and passenger strands of siRNAs whose T_*m*_ value is below 21.5 °C for reducing the seed-dependent off-target effects [8, 42]. This is another unique feature of siDirect. In the present study, siRNAs whose T_*m*_ value was below 21.5 °C, satisfying the rules prescribed by Ui-Tei et al. [34], Reynolds et al. [35], and Amarzguioui and Prydz [6], was selected.

The next online tool used in the present study for the design of siRNAs was “*i-Score* Designer,” a second-generation algorithm that adopts a linear regression model for computing the nucleotide preferences at each position on the antisense strand. The tool calculates *i-Score* for each of the siRNA which is completely based on the nucleotide preferences at each site on a scale of 0–100. The number of functional siRNAs predicted by *i-Score* Designer for the conserved regions of the three genes was as follows: (i) M — 193, (ii) N — 712, and (iii) S — 2296 (Supplementary Tables 10, 11 and 12). siRNAs whose *i-Score* ≥ 65 alone were considered for further analyses.

OligoWalk web server was utilized in the present study for the prediction of siRNAs that have an efficiency of more than 70% in silencing the target mRNA. The program was designed by taking into consideration the thermodynamic aspects (the free energy changes of different equilibrium states, viz. unimolecular (ΔG^o^_intra-siRNA_) and bimolecular (ΔG^o^_inter-siRNA_) siRNA folding, unimolecular folding state of mRNA at the siRNA binding region (ΔG^o^_target structure_) in addition to the hybridized state of siRNA, and target mRNA (ΔG^o^_duplex_)) and sequence features of siRNA for predicting the efficacy of the siRNA molecule by the help of support vector machine (SVM) [7, 33]. The number of siRNAs predicted by the OligoWalk web server for the three genes was as follows: (i) M — 50, (ii) N — 140, and (iii) S — 682 (Supplementary Tables 13, 14 and 15).

siRNAs that were predicted in common by all the four siRNA prediction tools were further screened using the siRNA scales web server for their efficiency to cleave the target mRNA at a cutoff value of ≤ 30 (except for the “M” gene, where none of the siRNAs designed by siDirect had in common with those predicted by RNAxs, *i-Score* Designer, and OligoWalk. Hence siRNAs predicted in common by RNAxs, *i-Score* Designer, and OligoWalk were considered for further analyses). A few antisense strands were eliminated in the present study as their predicted value of efficiency was > 30. siRNA scales were built on a linear regression model by considering the following three parameters: (i) thermodynamic stability (ΔG values) of the first two base pairs and the last two base pairs in the antisense strand of siRNA, (ii) nucleotide preferences at specific positions, and (iii) G + C content [11].

The total number of siRNAs predicted in common by all the four siRNA design tools and further shortlisted by siRNA scales was as follows: (i) M — 14, (ii) N — 6, and (iii) S — 66 (Supplementary Table 16) — (step 1).

One of the important parameters about the functionality of a siRNA is the Gibbs free energy (dG) [43]. *i-Score* Designer calculates the whole dG values of all the predicted siRNAs. Ichihara et al. [9] observed a high correlation coefficient between observed and predicted siRNAs with *i-Score* when dG values were elevated from −52.0 to −34.6 kcal/mol. A cutoff dG value of ≥ −34.6 kcal/mol was imposed in the present study for the shortlisting of siRNAs [44]. In addition to this, in the same study, it was found that a median number of 65 active siRNAs per mRNA from the human RefSeq database was predicted when *i-Score* and dG values were > 65 and ≥ −34.6, respectively [9]. As a result, siRNAs designed by the *i-Score* designer, whose *i-Score* and dG values ≥ 65 and ≥ −34.6, respectively, were shortlisted for further analyses.

Another important feature concerning the functionality of siRNA is the percentage content of GC. Higher GC content hinders the unwinding of the duplex siRNA. Lower GC content may lead to weak interactions between the antisense (guide) strand of siRNA and target mRNA [6]. Amarzguioui and Prydz [6] suggested the GC range of 31.6–57.9% to be optimal for functionality. However, optimal % GC content was varying from one study to another. For example, Chalk et al. [45] suggested % GC content from 36 to 53 to be effective, whereas Elbashir et al. [46] suggested % GC content from 32 to 79 to be effective [13]. In the present study, the optimal GC content was set in the range of 31.6–53%. In particular, those siRNAs whose % GC < 31.6 were eliminated.

The number of siRNAs obtained from step 1 whose dG ≥ −34.6 kcal/mol and GC content in the range from 31.6 to 53% was 30 (M gene — 7, N gene — 4, and S gene — 19) (Supplementary Table 17) (step 2).

**Fig. 1:**
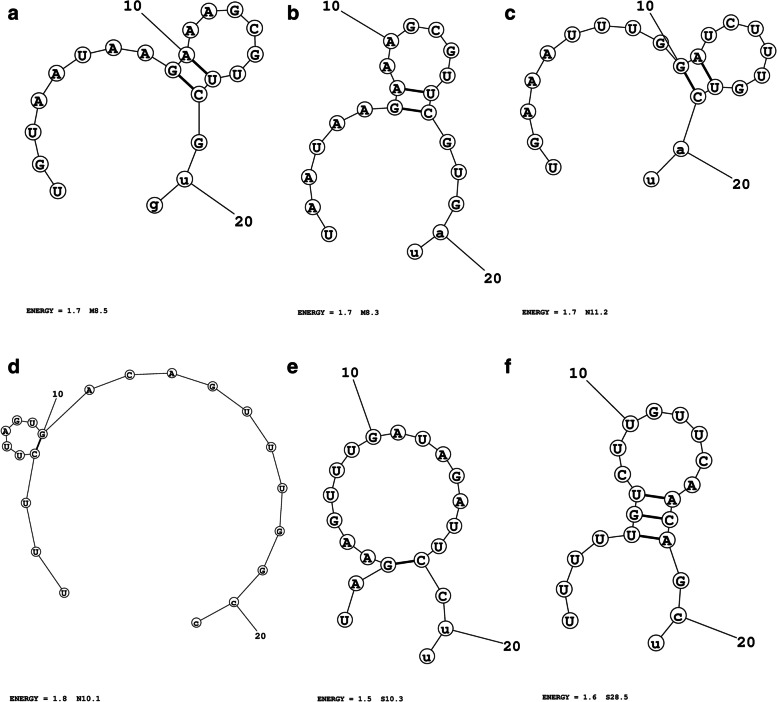
**a**–**f** Secondary structure of the guide strands of siRNAs and their energy values

MaxExpect web server was utilized in the present study for the prediction of the secondary structure of the antisense strands of the shortlisted siRNAs. The server generates the structure(s), in which the base pairs have the highest possibility of being accurate [36]. siRNAs whose free energy of folding ≥ 1.5 were shortlisted for further analyses [32]. The number of siRNAs obtained from step 2 that has passed the set criterion (energy ≥ 1.5) for M, N, and S genes was 7, 4, and 19, respectively (Supplementary Table 18), (Supplementary Figs. S1 a–c), (Fig. 1 a–f), (step 3).

**Fig. 2:**
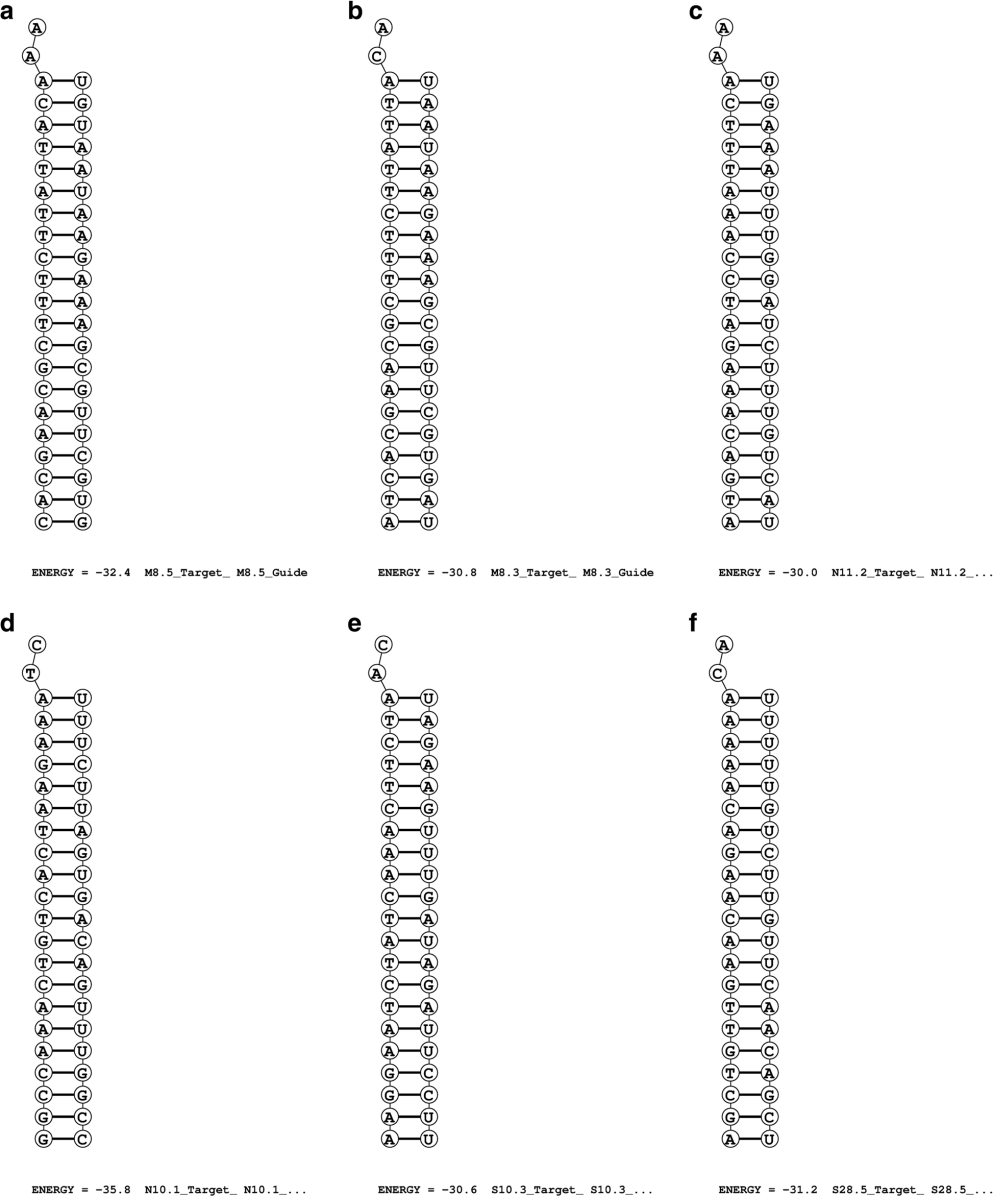
**a**–**f** Lowest free energy structures of guide strands of siRNAs with their corresponding target regions and their energy values

DuplexFold web server was used in the present study for calculating the free energy of folding between the guide strand and the target region. The server generates the structure between two RNA strands that has the lowest free energy state without allowing the intramolecular base pairs formation [37]. Those siRNAs whose free energy of folding values ≤ −30 were considered for further analyses [30, 32]. The number of siRNAs obtained from step 3, which have passed the set criterion (energy ≤ −30) for M, N, and S genes was 5, 4, and 16, respectively (Supplementary Table 19), (Supplementary Fig. S2 a–c and Fig. 2 a–f — Step 4).

SMEpred is a SVM-based method for predicting the efficacy of normal siRNAs as well as chemically modified siRNAs that are designed from a given mRNA or a gene sequence [38]. SMEpred web server was utilized in the present study to further screen the siRNAs obtained from the preceding steps for their efficacy. siRNAs with efficacy scores ≥ 85 were shortlisted for further analyses. The number of siRNAs at this stage for M, N, and S genes was 5, 3, and 13, respectively (step 5).

BLAST® was used to assess the extent of off-target matches of siRNAs designed in the present study. None of the 21 nucleotides of the guide strand of siRNAs targeting M, N, and S regions matched/aligned with the subject sequences in the human G + T database. The minimum to maximum identity ranged from 12/12 to 19/21, respectively. The identity of 19/21 had a very high expect value of 184, which is not significant at the default expect (E) threshold value of 0.05. Only S31.1 siRNA had the identity of 20/21 at an E-value of 184 (data not shown). Thus, none of the siRNAs designed in the present study had off-target effects at the default E-value [28]. The number of siRNAs at this stage for M, N, and S genes was 5, 3, and 13, respectively (Supplementary Tables 20, 21 and 22) (step 6).

The top 2 siRNAs obtained from step 6 for each of the three regions in terms of the SMEpred score were finalized and were used for molecular docking studies (Tables 2, 3 and 4).

**Table 2:** siRNAs designed in the present study for the “M” gene and various parameters

siRNA	Conserved region ID	Target sequence (21 + 2 nt)	siRNA sequence (antisense/guide) 21 nt	Sense/passenger (19 nt)	SMEpred (efficacy)	Free energy of binding	Free energy of folding	Whole dG (kcal/mol)	GC content (%)	siRNA scales	RNAxs (position)	OligoWalk (probability value)	Guide (T_***m***_)	Passenger (T_***m***_)	Position (siDirect)	***i-Score***
M8.5	8	CACGAACGCTTTCTTATTACAAA	UGUAAUAAGAAAGCGUUCGug	CGAACGCUUUCUUAUUACA	99.5	−32.4	1.7	−31.9	36.8	4	118/(98–120)	0.808041				75.2
M8.3	8	ATCACGAACGCTTTCTTATTACA	UAAUAAGAAAGCGUUCGUGau	CACGAACGCUUUCUUAUUA	93.3	−30.8	−	−	36.8	8	116/(96–118)	0.880858				70.3

**Table 3:** siRNAs designed in the present study for the “N” gene and various parameters

siRNA	Conserved region ID	Target sequence (21 + 2 nt)	siRNA sequence (antisense/guide) 21 nt	Sense/passenger (19 nt)	SMEpred (efficacy)	Free energy of binding	Free energy of folding	Whole dG (kcal/mol)	GC content (%)	siRNA scales	RNAxs (position)	OligoWalk (probability value)	Guide (T_***m***_)	Passenger (T_***m***_)	Position (siDirect)	***i-Score***
N11.2	11	ATGACAAAGATCCAAATTTCAAA	UGAAAUUUGGAUCUUUGUCau	GACAAAGAUCCAAAUUUCA	94.5	−30	1.7	−31.1	31.6	15	58	0.78544	0.4	19.2	38–60	73.6
N10.1	10	GGCCAAACTGTCACTAAGAAATC	UUUCUUAGUGACAGUUUGGcc	CCAAACUGUCACUAAGAAA	87.1	−35.8	1.8	−33.1	36.8	17	43	0.923617	11.7	16.7	23–45	74.5

**Table 4:** siRNAs designed in the present study for the “S” gene and various parameters

siRNA	Conserved region ID	Target sequence (21 + 2 nt)	siRNA sequence antisense/guide) 21 nt	Sense/passenger (19 nt)	SMEpred (efficacy)	Free energy of binding	Free energy of folding	Whole dG (kcal/mol)	GC content (%)	siRNA scales	RNAxs (position)	OligoWalk (probability value)	Guide (T_***m***_)	Passenger (T_***m***_)	siDirect (position)	***i-Score***
S10.3	10	AAGGAATCTATCAAACTTCTAAC	UAGAAGUUUGAUAGAUUCCuu	GGAAUCUAUCAAACUUCUA	100.7	−30.6	1.5	−31.9	31.6	7	67	0.90315	17.7	16	47–69	79
S28.5	28	AGCTGTTGAACAAGACAAAAACA	UUUUUGUCUUGUUCAACAGcu	CUGUUGAACAAGACAAAAA	97.3	−31.2	1.6	−30.3	31.6	11	113	0.893136	14.9	20.5	93–115	72.7

The sense strand sequences of the six shortlisted siRNAs (Tables 2, 3 and 4) were screened for on/off-target similarities against the forty whole-genome sequences of SARS-CoV2. It was found that all the six shortlisted siRNAs showed an on-target effect (i.e., matched with the intended regions), and none of them showed off-target matches.

The human AGO proteins consist of four functional domains, namely N-terminal domain (N), PIWI/Argonaute/Zwille (PAZ) domain, MID domain, and P-element-induced wimpy tested (PIWI) domain. The cap-binding-like domain (MC) is found within the MID domain [47, 48]. The PIWI domain in AGO2 and 3 contains four amino acids: aspartic acid (D), glutamic acid (E), aspartic acid (D), and histidine (H) known as “catalytic tetrad,” which is essential for the cleavage of the mRNA. The PAZ and MID domains bind with the 3′ and 5′ end of the guide strand, respectively [47]. In the present study, the docked complex with the lowest binding energy, i.e., model 1 among the output complexes, was considered to be the best one. Besides considering the binding score, the domains with which RNA interacted were also studied. This was known by looking at the interacting residues of the AGO protein with the guide strand of siRNA (Supplementary Table 23). The amino acid sequence positions of the PAZ and PIWI domains were known from the UniProt database by giving the PDB ID of the protein that was used as a template by HDOCK.

**Fig. 3:**
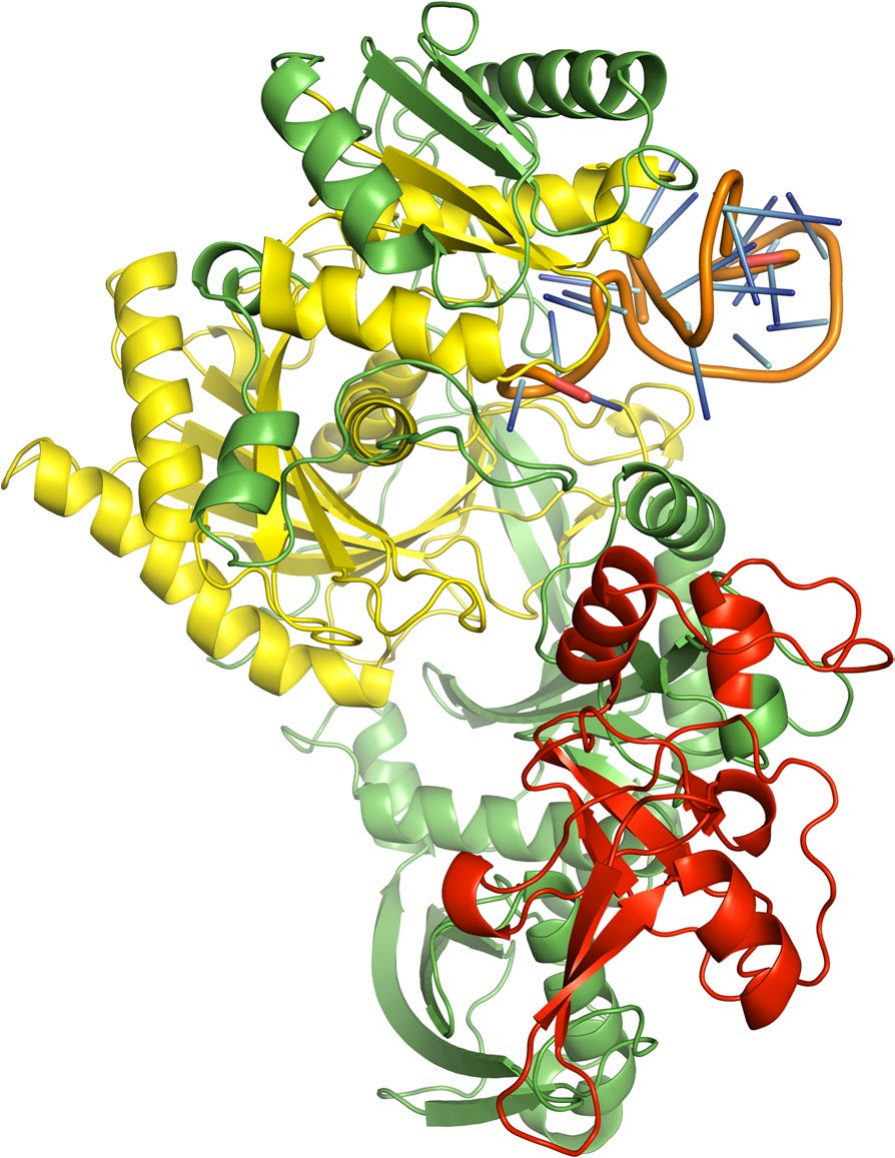
A docked complex of AGO2 and M8.5. RNA, PAZ, PIWI, and the remaining regions are represented in orange, red, yellow, and green colors, respectively

**Fig. 4:**
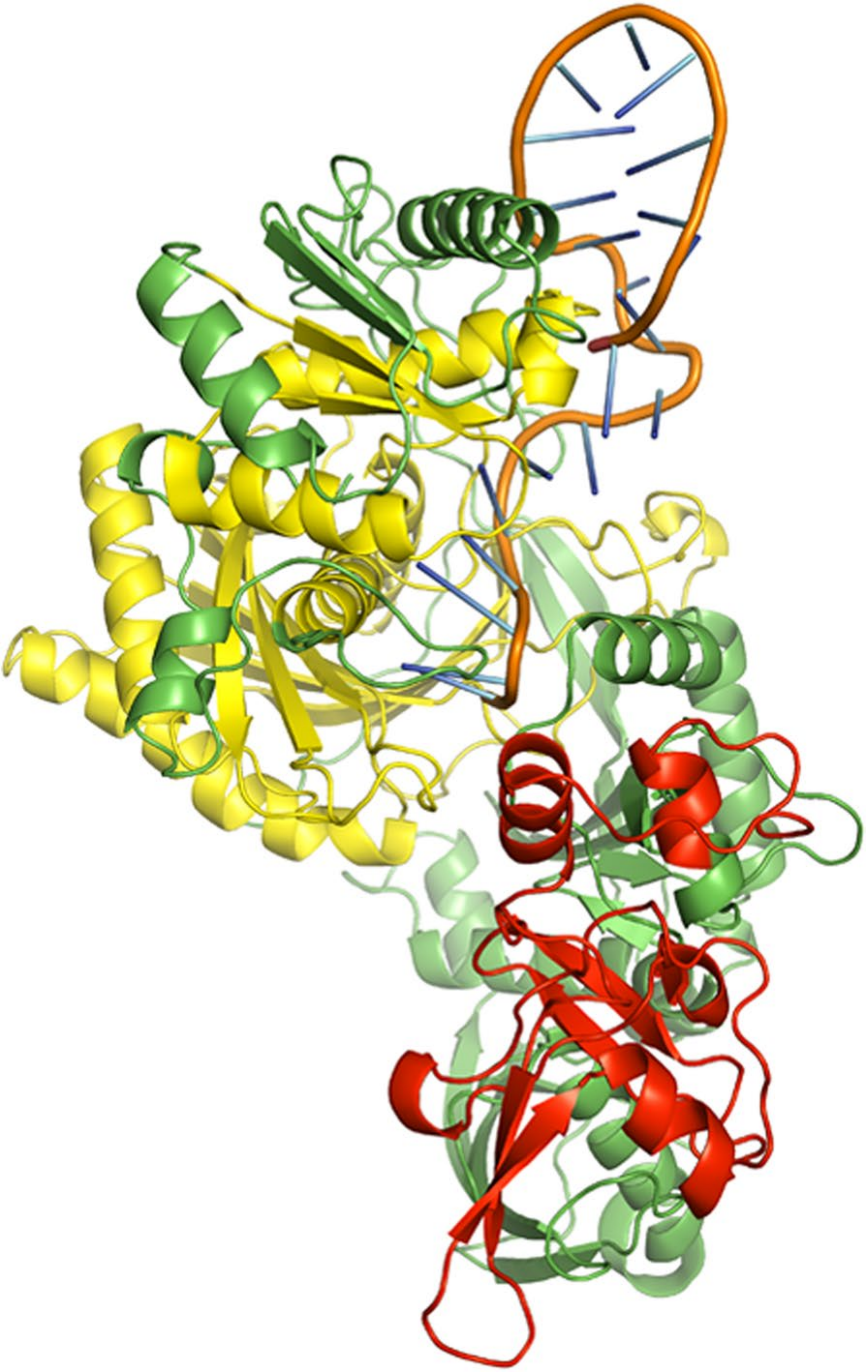
A docked complex of AGO2 and M8.3. RNA, PAZ, PIWI, and the remaining regions are represented in orange, red, yellow, and green colors, respectively

**Fig. 5:**
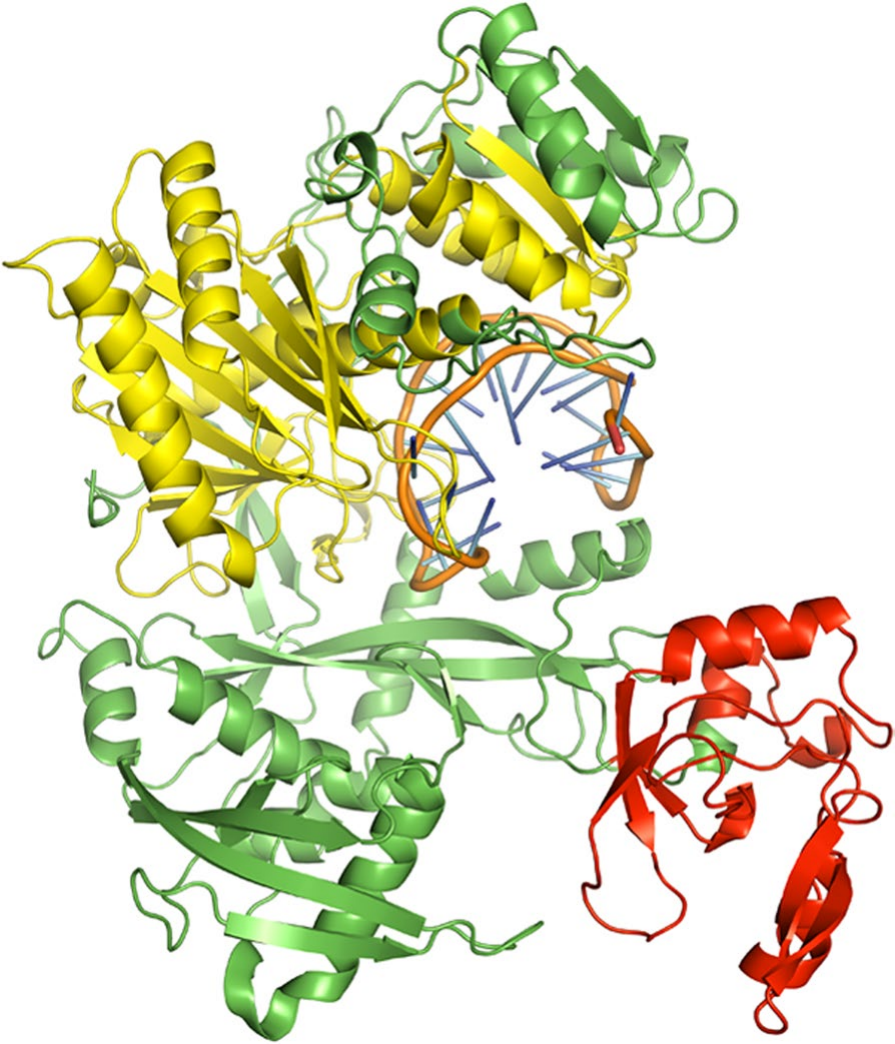
A docked complex of AGO2 and N11.2. RNA, PAZ, PIWI, and the remaining regions are represented in orange, red, yellow, and green colors, respectively

**Fig. 6:**
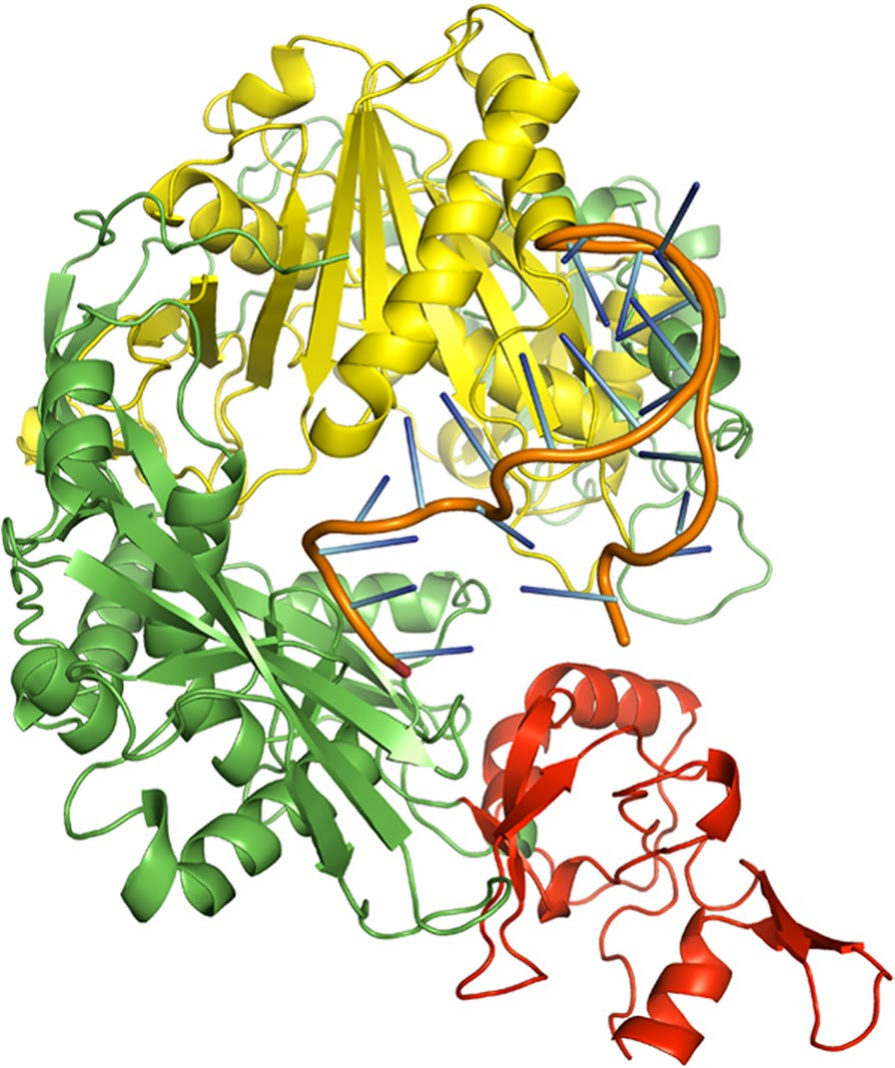
A docked complex of AGO2 and S10.3. RNA, PAZ, PIWI, and the remaining regions are represented in orange, red, yellow, and green colors, respectively

**Fig. 7:**
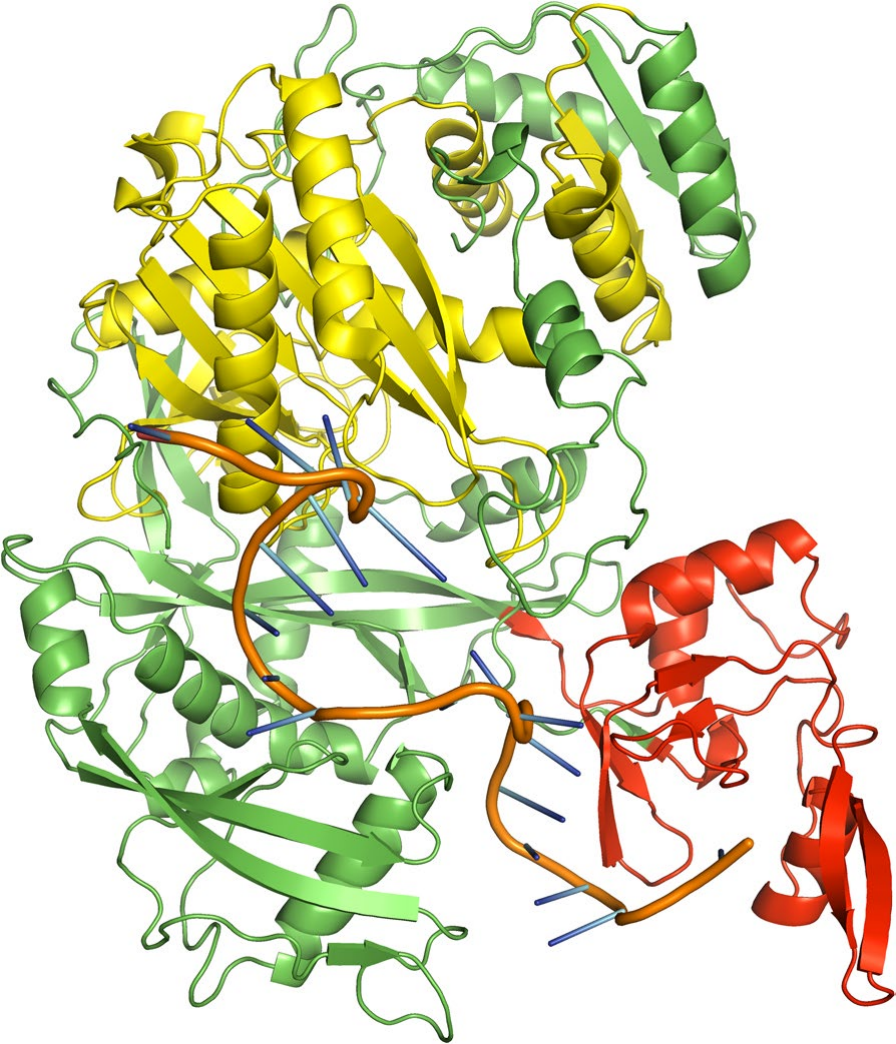
A docked complex of AGO2 and S28.5. RNA, PAZ, PIWI, and the remaining regions are represented in orange, red, yellow, and green colors, respectively

**Fig. 8:**
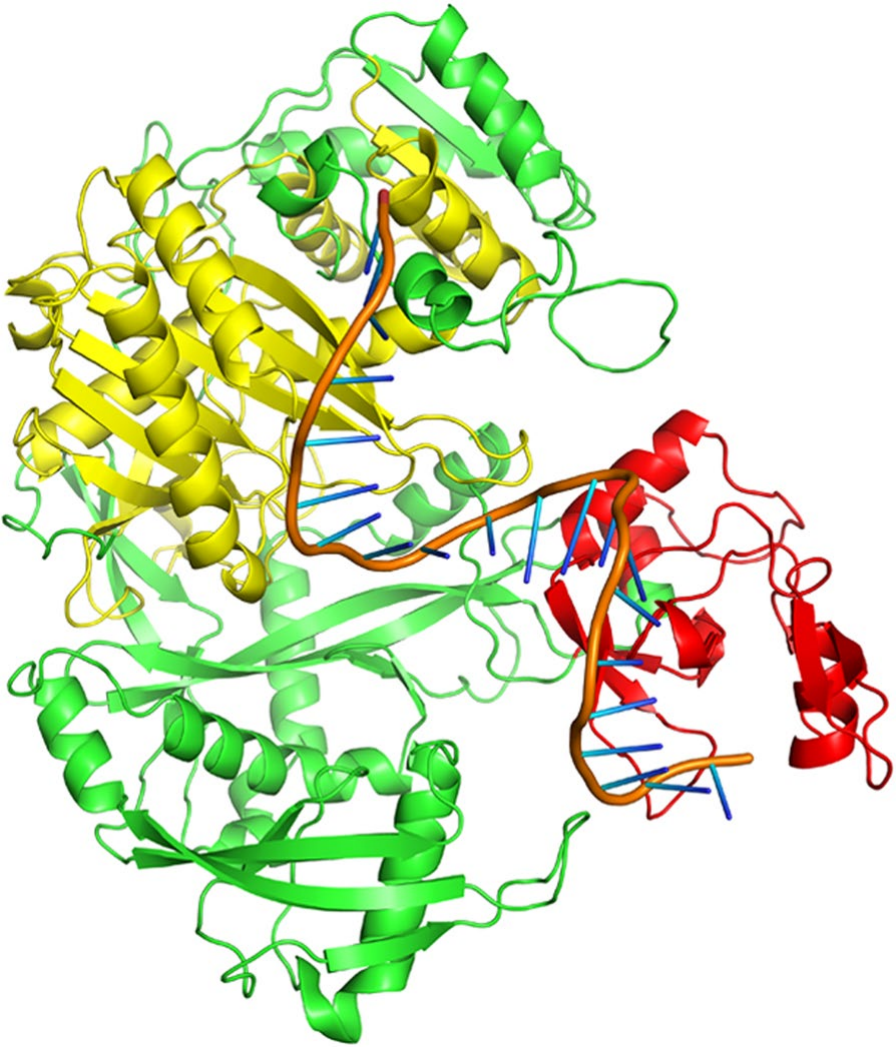
A docked complex of AGO2 and N10.1. RNA, PAZ, PIWI, and the remaining regions are represented in orange, red, yellow, and green colors, respectively

A few bases of the guide strands of siRNAs were protruded from the docked complexes of AGO with siRNAs M8.5 (Fig. 3), M8.3 (Fig. 4), N11.2 (Fig. 5), S10.3 (Fig. 6), and S28.5 (Fig. 7). This was also observed in the studies of Chowdhury et al. [30] and Shawan et al. [31]. siRNA N10.1 has spread across the domains in the docked complex when compared with the remaining siRNAs (Fig. 8). All the siRNAs in the docked complexes were found to interact with the PIWI domain. Concerning the PAZ domain, only N10.1 and S28.5 siRNAs were found to show interaction. Among the siRNAs designed for S, M, and N genes, S28.5, M8.3, and N11.2 have the lowest (more negative value) docking scores of −356.13, −350.68, and −347.70, respectively (Supplementary Table 23). Although only N10.1 and S28.5 siRNAs have interacted with both PAZ and PIWI domains, the remaining siRNAs are efficacious in terms of the SMEpred score. More or less a similar situation was also observed in the study of Chowdhury et al. [30], where siRNAs from cluster 2 had fewer interactions with AGO2 protein in the docked complex compared to cluster 1. However, siRNAs from cluster 2 outperformed in terms of siRNAPred scores by showing better efficacy than the g15 siRNA from cluster 1, which is the best candidate (g15) in terms of molecular interactions with the AGO2 protein.

Apart from designing functional siRNAs in silico, efficient delivery of siRNAs is essential for achieving the target gene silencing. Different methods for in vivo delivery of siRNAs are available from Chen et al. [49], Van den Berg et al. [50], and Lundstrom [51]. Also, strategies to reduce off-target gene silencing are outlined in Liu et al. [13], Dar et al. [38], and Chen et al. [49].

In the present study, six siRNAs were shortlisted targeting M, N, and S genes of SARS-CoV2 that were found to be effective based on the outcomes of various tools that were used. However, in the future, siRNAs designed in the present study need to be further tested in vitro and in vivo to confirm their efficacy for the treatment of COVID-19.

## Conclusion

siRNA-mediated gene silencing is one of the promising approaches for treating COVID-19. In this study, a total of six different functional siRNAs targeting three structural genes M, N, and S (two siRNAs for each of the three genes) were designed and tested for their efficacy using different online tools, along with molecular docking studies. The efficiency in the design of siRNAs against SARS-CoV2 was maximized in the present study by using diverse online tools and subsequent shortlisting of the designed siRNAs based on a few important parameters. All the six siRNAs designed in the present study were found to be effective in inhibiting the target. Experimental validations of siRNAs designed in the present study need to be carried out in the future.

## Supplementary Information

**Additional file 1: Supplementary Table 1.** Conserved regions of gene ‘M’ used for the design of siRNAs.

**Additional file 2: Supplementary Table 2.** Conserved regions of gene ‘N’ used for the design of siRNAs.

**Additional file 3: Supplementary Table 3.** Conserved regions of gene ‘S’ used for the design of siRNAs.

**Additional file 4:** **Supplementary Table 4.** List of siRNAs predicted by RNAxs for various conserved regions of ‘M’ gene.

**Additional file 5: Supplementary Table 5.** List of siRNAs predicted by RNAxs for various conserved regions of ‘N’ gene.

**Additional file 6: Supplementary Table 6.** List of siRNAs predicted by RNAxs for various conserved regions of ‘S’ gene.

**Additional file 7: Supplementary Table 7.** List of siRNAs predicted by siDirect for various conserved regions of the M gene.

**Additional file 8: Supplementary Table 8.** List of siRNAs predicted by siDirect for various conserved regions of the ‘N’ gene.

**Additional file 9: Supplementary Table 9.** List of siRNAs predicted by siDirect for various conserved regions of the ‘S’ gene.

**Additional file 10: Supplementary Table 10.** List of siRNAs predicted by *i-Score* Designer for various conserved regions of the 'M' gene

**Additional file 11: Supplementary Table 11.** List of siRNAs predicted by *i-Score* Designer for various conserved regions of the 'N' gene

**Additional file 12: Supplementary Table 12.** List of siRNAs predicted by *i-Score* Designer for various conserved regions of the 'S' gene

**Additional file 13: Supplementary Table 13.** List of siRNAs predicted by OligoWalk for various conserved regions of the ‘M’ gene.

**Additional file 14: Supplementary Table 14.** List of siRNAs predicted by OligoWalk for various conserved regions of the ‘N’ gene.

**Additional file 15: Supplementary Table 15.** List of siRNAs predicted by OligoWalk for various conserved regions of the ‘S’ gene.

**Additional file 16: Supplementary Table 16.** List of siRNAs obtained at step 1 for M, N & S genes

**Additional file 17: Supplementary Table 17.** List of siRNAs obtained at step 2 for M, N & S genes

**Additional file 18: Supplementary Table 18.** List of siRNAs obtained at step 3 for M, N & S genes

**Additional file 19: Supplementary Table 19.** List of siRNAs obtained at step 4 for M, N & S genes

**Additional file 20: Supplementary Table 20.** siRNAs predicted for M gene at Step 5/ 6 and their parameters.

**Additional file 21: Supplementary Table 21.** siRNAs predicted for N gene at Step 5/ 6 and their parameters.

**Additional file 22: Supplementary Table 22.** siRNAs predicted for S gene at Step 5/ 6 and their parameters.

**Additional file 23: Supplementary Table 23.** List of interacting residues of human AGO2 protein with the nucleotides of the guide strands of siRNAs of M, N & S genes within 5.0 Å.

**Additional file 24: Supplementary Fig. S1 a–c.** Structures of guide strands of siRNAs of M, N & S genes and their energy values.

**Additional file 25: Supplementary Fig. S2 a–c.** Lowest free energy structures of guide strands of siRNAs of M, N & S genes and their corresponding target regions and their energy values.

## Data Availability

The datasets generated during the current study are included within the main text of the article and in the supplementary materials. Data (if any) shall be provided upon suitable request to the corresponding author.

## Abbreviations

SARS-CoV2: Severe acute respiratory syndrome coronavirus-2
COVID-19: Coronavirus disease 2019
siRNA: Short interfering RNA
RNAi: RNA interference
mRNA: Messenger RNA
RISC: RNA-induced silencing complex
E: Envelope
M: Membrane
S: Spike
N: Nucleocapsid
NCBI: National Centre for Biotechnology Information
U: Ui-Tei
R: Reynolds
A: Amarzguioui
T_m_: Seed-duplex stability
*i-Score*: Inhibitory score
BLAST: Basic Local Alignment Search Tool
WR: Worst rank
FFT: Fast Fourier transform
3D: Three dimensional
PDB: Protein Data Bank
AGO2: Argonaute-2 protein;
AGO: Argonaute
RMSD: Root-mean-square deviation
SVM: Support vector machine
dG: Gibbs free energy
E: Expect
N: N-terminal domain
PAZ: PIWI/Argonaute/Zwille
PIWI: P-element-induced wimpy
D: Aspartic acid
E: Glutamic acid
D: Aspartic acid
H: Histidine
