# Supplementary material for: Design of siRNA molecules for silencing of membrane glycoprotein, nucleocapsid phosphoprotein, and surface glycoprotein genes of SARS-CoV2
Source: J Genet Eng Biotechnol. 2022 Apr 28;20:65. doi: 10.1186/s43141-022-00346-z (PMC9047631; doi:10.1186/s43141-022-00346-z)
Supplement: Supplementary file 1 — Additional file 1: Supplementary Table 1. Conserved regions of gene ‘M’ used for the design of siRNAs. [file 43141_2022_346_MOESM1_ESM.docx]

**Supplementary Table 1: Conserved regions of gene ‘M’ used for the design of siRNAs**

| **Sr. No.** | **Conserved region (Conserved region ID)** |
| --- | --- |
| 1. | AGCTACTTCATTGCTTCTTTCAGACTGTTTGCGCGTACGCGTTCCATGTGGTCATTCAATCCAGAAACTAACATTCTTCT (6) |
| 2. | GGAGCTGTGATCCTTCGTGGACATCTTCGTATTGCTGGACACCATCTAGGACGCTGTGACATCAAGGACCTGCCTAAAGAAATCACTGTTGCTACATCACGAACGCTTTCTTATTACAAATTGGGAGCTTCGCAGCGTGTAGCAGGTGA (8) |
