# Supplementary material for: Design of siRNA molecules for silencing of membrane glycoprotein, nucleocapsid phosphoprotein, and surface glycoprotein genes of SARS-CoV2
Source: J Genet Eng Biotechnol. 2022 Apr 28;20:65. doi: 10.1186/s43141-022-00346-z (PMC9047631; doi:10.1186/s43141-022-00346-z)
Supplement: Supplementary file 2 — Additional file 2: Supplementary Table 2. Conserved regions of gene ‘N’ used for the design of siRNAs. [file 43141_2022_346_MOESM2_ESM.docx]

**Supplementary Table 2 Conserved regions of gene ‘N’ used for the design of siRNAs**

| **Sr. No.** | **Conserved region (Conserved region ID)** |
| --- | --- |
| 1. | CAGTGGGGCGCGATCAAAACAACGTCGGCCCCAAGGTTTACCCAATAATACTGCGTCTTGGTTCACCGCTCTCACTCAACATGGCAAGGAAG (3) |
| 2. | CTCGAGGACAAGGCGTTCCAATTAACACCAATAGCAGTCCAGATGACCAAATTGGCTACTACCGAAGAGCTACCAGACGAATTCGTGGTGGTGACGGTAAAATGAAAGATCTCAGTCCAAGATGGTATTTCTACTACCTAGGAACTGGGCCAGAA (4) |
| 3. | CCCGCAATCCTGCTAACAATGCTGCAATCGTGCTACAACTTCCTCAAGGAACAACATTGCCAAAAGGCTTCTACGCAGAAGGGAGCAGAGGCGGCAGTCAAGCCTCTTCTCGTTCCT (7) |
| 4. | TGGTAAAGGCCAACAACAACAAGGCCAAACTGTCACTAAGAAATCTGCTGCTGAGGCTTCTAAGAAGCCTCGGCAAAAACGTACTGCCACTAAAGCATACAATGTAACACAAGCTTTCGGCAGACGTGGTCCAGAACAAACCCAAGGAAATTTTGGGGACCAGGAACTAATCAGACAAGGAACTGATTACAAACATTGGCCGCAAATTGCACAATTTGCCCCCAGCGCTTCAGCGTTCTTCGGAATGTCGCGCATTGGCATGGAAGTCAC (10) |
| 5. | GGAACGTGGTTGACCTACACAGGTGCCATCAAATTGGATGACAAAGATCCAAATTTCAAAGATCAAGTCATTTTGCTGAA (11) |
| 6. | ATGAAACTCAAGCCTTACCGCAGAGACAGAAGAAACAGCAAACTGTGACTCTTCTTCCTGCTGCAGATTTGGATGATTTCTCCAAACAATTGCAACAATCCATGAG (14) |
