# Supplementary material for: Design of siRNA molecules for silencing of membrane glycoprotein, nucleocapsid phosphoprotein, and surface glycoprotein genes of SARS-CoV2
Source: J Genet Eng Biotechnol. 2022 Apr 28;20:65. doi: 10.1186/s43141-022-00346-z (PMC9047631; doi:10.1186/s43141-022-00346-z)
Supplement: Supplementary file 4 — Additional file 4: Supplementary Table 4. List of siRNAs predicted by RNAxs for various conserved regions of ‘M’ gene. [file 43141_2022_346_MOESM4_ESM.docx]

**Supplementary Table 4:** List of siRNAs predicted by RNAxs for various conserved regions of ‘M’ gene

**List of siRNAs predicted by RNAxs for the ‘M’ gene for the ‘conserved region 6’**

| **WORST RANK** | **Position** | **Target sequence** | **siRNA sequence** | **Access 8nts** | **Access 16nts** | **Energy A.** | **Sequence A.** | **Self-Folding** | **Free End** |  |
| --- | --- | --- | --- | --- | --- | --- | --- | --- | --- | --- |
|  |  |  |  |  |  |  |  |  |  |  |
| 9 | 75 | CAATCCAGAAACTAACATT | AATGTTAGTTTCTGGATTG | 0.5553 | 0.2122 | 0.6379 | 0.7500 | 1.0000 | 1 | 1 |
| 10 | 19 | AGCTACTTCATTGCTTCTT | AAGAAGCAATGAAGTAGCT | 0.1571 | 0.1529 | 0.7155 | 0.7500 | 0.9783 | 0.625 | 1 |
| 10 | 74 | TCAATCCAGAAACTAACAT | ATGTTAGTTTCTGGATTGA | 0.5854 | 0.2113 | 0.569 | 0.7500 | 1.0000 | 1 | 1 |
| 11 | 20 | GCTACTTCATTGCTTCTTT | AAAGAAGCAATGAAGTAGC | 0.1556 | 0.1517 | 0.8534 | 1.0000 | 1.0000 | 1 | 1 |
| 11 | 68 | GGTCATTCAATCCAGAAAC | GTTTCTGGATTGAATGACC | 0.2143 | 0.0109 | 0.7069 | 0.7500 | 0.9837 | 0.75 | 1 |
| 12 | 70 | TCATTCAATCCAGAAACTA | TAGTTTCTGGATTGAATGA | 0.2190 | 0.2087 | 0.5517 | 0.7500 | 0.9837 | 0.875 | 1 |
| 12 | 67 | TGGTCATTCAATCCAGAAA | TTTCTGGATTGAATGACCA | 0.2143 | 0.0107 | 0.8448 | 0.7500 | 0.9837 | 0.625 | 1 |
| 13 | 30 | TGCTTCTTTCAGACTGTTT | AAACAGTCTGAAAGAAGCA | 0.1323 | 0.0492 | 0.8534 | 0.7500 | 1.0000 | 1 | 1 |
| 14 | 61 | TCCATGTGGTCATTCAATC | GATTGAATGACCACATGGA | 0.5100 | 0.0017 | 0.6897 | 0.5000 | 0.9837 | 0.75 | 1 |
| 14 | 21 | CTACTTCATTGCTTCTTTC | GAAAGAAGCAATGAAGTAG | 0.1588 | 0.1494 | 0.5 | 0.5000 | 1.0000 | 1 | 1 |
| 15 | 29 | TTGCTTCTTTCAGACTGTT | AACAGTCTGAAAGAAGCAA | 0.1418 | 0.0245 | 0.5 | 0.5000 | 1.0000 | 1 | 1 |
| 15 | 60 | TTCCATGTGGTCATTCAAT | ATTGAATGACCACATGGAA | 0.0589 | 0.0016 | 0.6638 | 0.5000 | 0.9837 | 0.75 | 1 |
| 16 | 69 | GTCATTCAATCCAGAAACT | AGTTTCTGGATTGAATGAC | 0.2156 | 0.2085 | 0.6121 | 0.5000 | 0.9837 | 0.875 | 1 |
| 16 | 58 | CGTTCCATGTGGTCATTCA | TGAATGACCACATGGAACG | 0.0154 | 0.0015 | 0.5517 | 0.7500 | 0.9837 | 1 | 1 |
| 17 | 31 | GCTTCTTTCAGACTGTTTG | CAAACAGTCTGAAAGAAGC | 0.0132 | 0.0125 | 0.7069 | 0.7500 | 1.0000 | 1 | 1 |
| 17 | 26 | TCATTGCTTCTTTCAGACT | AGTCTGAAAGAAGCAATGA | 0.1652 | 0.0281 | 0.5172 | 0.5000 | 1.0000 | 1 | 1 |
| 17 | 59 | GTTCCATGTGGTCATTCAA | TTGAATGACCACATGGAAC | 0.0544 | 0.0015 | 0.5 | 0.7500 | 0.9837 | 0.875 | 1 |
| 17 | 62 | CCATGTGGTCATTCAATCC | GGATTGAATGACCACATGG | 0.5101 | 0.0020 | 0.4741 | 0.5000 | 1.0000 | 1 | 1 |

**List of siRNAs predicted by RNAxs for the ‘M’ gene for the ‘conserved region 8’**

| **WORST RANK** | **Position** | **Target sequence** | **siRNA sequence** | **Access 8nts** | **Access 16nts** | **Energy A.** | **Sequence A.** | **Self-Folding** | **Free End** |  |
| --- | --- | --- | --- | --- | --- | --- | --- | --- | --- | --- |
|  |  |  |  |  |  |  |  |  |  |  |
| 14 | 109 | GCTACATCACGAACGCTTT | AAAGCGTTCGTGATGTAGC | 0.1555 | 0.1284 | 0.8534 | 1.0000 | 0.9348 | 0.75 | 1 |
| 17 | 122 | CGCTTTCTTATTACAAATT | AATTTGTAATAAGAAAGCG | 0.7265 | 0.0363 | 0.9138 | 1.0000 | 1.0000 | 1 | 1 |
| 20 | 108 | TGCTACATCACGAACGCTT | AAGCGTTCGTGATGTAGCA | 0.1775 | 0.1516 | 0.7155 | 0.7500 | 0.9348 | 0.75 | 1 |
| 20 | 116 | CACGAACGCTTTCTTATTA | TAATAAGAAAGCGTTCGTG | 0.1086 | 0.0683 | 0.7586 | 0.7500 | 1.0000 | 1 | 1 |
| 20 | 34 | CGTGGACATCTTCGTATTG | CAATACGAAGATGTCCACG | 0.2204 | 0.0287 | 0.6293 | 0.7500 | 1.0000 | 1 | 1 |
| 20 | 113 | CATCACGAACGCTTTCTTA | TAAGAAAGCGTTCGTGATG | 0.1213 | 0.0933 | 0.6207 | 0.7500 | 1.0000 | 1 | 1 |
| 22 | 121 | ACGCTTTCTTATTACAAAT | ATTTGTAATAAGAAAGCGT | 0.8482 | 0.0404 | 0.7672 | 0.7500 | 1.0000 | 1 | 1 |
| 22 | 83 | AGGACCTGCCTAAAGAAAT | ATTTCTTTAGGCAGGTCCT | 0.1196 | 0.0209 | 0.8362 | 0.7500 | 1.0000 | 1 | 1 |
| 24 | 115 | TCACGAACGCTTTCTTATT | AATAAGAAAGCGTTCGTGA | 0.1174 | 0.0739 | 0.7155 | 0.7500 | 1.0000 | 1 | 1 |
| 24 | 118 | CGAACGCTTTCTTATTACA | TGTAATAAGAAAGCGTTCG | 0.6401 | 0.0576 | 0.5862 | 0.7500 | 1.0000 | 1 | 1 |
| 25 | 123 | GCTTTCTTATTACAAATTG | CAATTTGTAATAAGAAAGC | 0.0865 | 0.0260 | 0.7069 | 0.7500 | 1.0000 | 1 | 1 |
| 26 | 33 | TCGTGGACATCTTCGTATT | AATACGAAGATGTCCACGA | 0.3552 | 0.0180 | 0.7845 | 0.7500 | 1.0000 | 1 | 1 |
| 27 | 85 | GACCTGCCTAAAGAAATCA | TGATTTCTTTAGGCAGGTC | 0.1076 | 0.0321 | 0.5948 | 0.5000 | 1.0000 | 1 | 1 |
| 28 | 103 | ACTGTTGCTACATCACGAA | TTCGTGATGTAGCAACAGT | 0.3198 | 0.0380 | 0.5345 | 0.7500 | 1.0000 | 1 | 1 |
| 28 | 86 | ACCTGCCTAAAGAAATCAC | GTGATTTCTTTAGGCAGGT | 0.1045 | 0.0319 | 0.5603 | 0.5000 | 1.0000 | 1 | 1 |
| 28 | 87 | CCTGCCTAAAGAAATCACT | AGTGATTTCTTTAGGCAGG | 0.0743 | 0.0190 | 0.6379 | 0.7500 | 1.0000 | 1 | 1 |
| 29 | 90 | GCCTAAAGAAATCACTGTT | AACAGTGATTTCTTTAGGC | 0.0672 | 0.0204 | 0.8534 | 1.0000 | 1.0000 | 1 | 1 |
| 30 | 119 | GAACGCTTTCTTATTACAA | TTGTAATAAGAAAGCGTTC | 0.6652 | 0.0504 | 0.5259 | 0.7500 | 1.0000 | 1 | 1 |
| 31 | 96 | AGAAATCACTGTTGCTACA | TGTAGCAACAGTGATTTCT | 0.1936 | 0.0672 | 0.5172 | 0.5000 | 1.0000 | 1 | 1 |
| 32 | 97 | GAAATCACTGTTGCTACAT | ATGTAGCAACAGTGATTTC | 0.3785 | 0.0676 | 0.5172 | 0.7500 | 1.0000 | 1 | 1 |
| 32 | 117 | ACGAACGCTTTCTTATTAC | GTAATAAGAAAGCGTTCGT | 0.1004 | 0.0631 | 0.5948 | 0.5000 | 1.0000 | 1 | 1 |
| 32 | 91 | CCTAAAGAAATCACTGTTG | CAACAGTGATTTCTTTAGG | 0.0649 | 0.0462 | 0.6983 | 0.7500 | 1.0000 | 1 | 1 |
| 32 | 30 | CCTTCGTGGACATCTTCGT | ACGAAGATGTCCACGAAGG | 0.1710 | 0.0077 | 0.569 | 0.7500 | 1.0000 | 1 | 1 |
| 33 | 89 | TGCCTAAAGAAATCACTGT | ACAGTGATTTCTTTAGGCA | 0.0659 | 0.0171 | 0.6466 | 0.5000 | 1.0000 | 1 | 1 |
| 34 | 102 | CACTGTTGCTACATCACGA | TCGTGATGTAGCAACAGTG | 0.3249 | 0.0420 | 0.5 | 0.5000 | 1.0000 | 1 | 1 |
| 35 | 120 | AACGCTTTCTTATTACAAA | TTTGTAATAAGAAAGCGTT | 0.7426 | 0.0461 | 0.6466 | 0.5000 | 1.0000 | 1 | 1 |
| 35 | 110 | CTACATCACGAACGCTTTC | GAAAGCGTTCGTGATGTAG | 0.1329 | 0.1086 | 0.5 | 0.5000 | 0.9348 | 0.75 | 1 |
| 36 | 52 | GCTGGACACCATCTAGGAC | GTCCTAGATGGTGTCCAGC | 0.0805 | 0.0041 | 0.5345 | 0.7500 | 0.9783 | 0.75 | 1 |
| 37 | 88 | CTGCCTAAAGAAATCACTG | CAGTGATTTCTTTAGGCAG | 0.0653 | 0.0155 | 0.5 | 0.5000 | 1.0000 | 1 | 1 |
| 38 | 114 | ATCACGAACGCTTTCTTAT | ATAAGAAAGCGTTCGTGAT | 0.1333 | 0.0837 | 0.6379 | 0.5000 | 1.0000 | 1 | 1 |
| 38 | 45 | TCGTATTGCTGGACACCAT | ATGGTGTCCAGCAATACGA | 0.0363 | 0.0031 | 0.6379 | 0.7500 | 0.9457 | 0.625 | 1 |
| 38 | 149 | CGCAGCGTGTAGCAGGTGA | TCACCTGCTACACGCTGCG | 0.0234 | 0.0193 | 0.6983 | 0.7500 | 0.9185 | 0.75 | 1 |
| 39 | 37 | GGACATCTTCGTATTGCTG | CAGCAATACGAAGATGTCC | 0.0147 | 0.0049 | 0.5862 | 0.7500 | 1.0000 | 1 | 1 |
| 39 | 80 | TCAAGGACCTGCCTAAAGA | TCTTTAGGCAGGTCCTTGA | 0.0485 | 0.0075 | 0.5 | 0.5000 | 0.9891 | 0.875 | 1 |
| 39 | 46 | CGTATTGCTGGACACCATC | GATGGTGTCCAGCAATACG | 0.0535 | 0.0030 | 0.5948 | 0.7500 | 0.9457 | 0.625 | 1 |
| 39 | 36 | TGGACATCTTCGTATTGCT | AGCAATACGAAGATGTCCA | 0.0991 | 0.0058 | 0.4914 | 0.5000 | 1.0000 | 1 | 1 |
| 40 | 32 | TTCGTGGACATCTTCGTAT | ATACGAAGATGTCCACGAA | 0.2383 | 0.0125 | 0.6293 | 0.5000 | 1.0000 | 1 | 1 |
| 40 | 71 | GCTGTGACATCAAGGACCT | AGGTCCTTGATGTCACAGC | 0.0146 | 0.0032 | 0.5086 | 0.7500 | 1.0000 | 1 | 1 |
| 41 | 82 | AAGGACCTGCCTAAAGAAA | TTTCTTTAGGCAGGTCCTT | 0.0576 | 0.0148 | 0.6379 | 0.5000 | 0.9891 | 0.625 | 1 |
| 41 | 72 | CTGTGACATCAAGGACCTG | CAGGTCCTTGATGTCACAG | 0.0140 | 0.0025 | 0.5 | 0.5000 | 1.0000 | 1 | 1 |
| 41 | 81 | CAAGGACCTGCCTAAAGAA | TTCTTTAGGCAGGTCCTTG | 0.0507 | 0.0101 | 0.4741 | 0.7500 | 0.9891 | 0.75 | 1 |
| 41 | 51 | TGCTGGACACCATCTAGGA | TCCTAGATGGTGTCCAGCA | 0.0822 | 0.0020 | 0.4828 | 0.5000 | 0.9783 | 0.75 | 1 |
