# Supplementary material for: Design of siRNA molecules for silencing of membrane glycoprotein, nucleocapsid phosphoprotein, and surface glycoprotein genes of SARS-CoV2
Source: J Genet Eng Biotechnol. 2022 Apr 28;20:65. doi: 10.1186/s43141-022-00346-z (PMC9047631; doi:10.1186/s43141-022-00346-z)
Supplement: Supplementary file 5 — Additional file 5: Supplementary Table 5. List of siRNAs predicted by RNAxs for various conserved regions of ‘N’ gene. [file 43141_2022_346_MOESM5_ESM.docx]

**Supplementary Table 5:** List of siRNAs predicted by RNAxs for various conserved regions of ‘N’ gene

**List of siRNAs predicted by RNAxs for the ‘conserved region 3’ of the ‘N’ gene**

| **WORST RANK** | **Position** | **Target sequence** | **siRNA sequence** | **Access 8nts** | **Access 16nts** | **Energy A.** | **Sequence A.** | **Self-Folding** | **Free End** |  |
| --- | --- | --- | --- | --- | --- | --- | --- | --- | --- | --- |
|  |  |  |  |  |  |  |  |  |  |  |
| 10 | 59 | CCCAATAATACTGCGTCTT | AAGACGCAGTATTATTGGG | 0.1680 | 0.1673 | 0.8534 | 1.0000 | 0.9511 | 0.625 | 1 |
| 11 | 82 | CACCGCTCTCACTCAACAT | ATGTTGAGTGAGAGCGGTG | 0.7022 | 0.0531 | 0.6379 | 0.7500 | 0.9946 | 0.75 | 1 |
| 11 | 78 | GGTTCACCGCTCTCACTCA | TGAGTGAGAGCGGTGAACC | 0.6818 | 0.0676 | 0.6293 | 0.7500 | 1.0000 | 1 | 1 |
| 13 | 50 | CAAGGTTTACCCAATAATA | TATTATTGGGTAAACCTTG | 0.3876 | 0.0088 | 0.6034 | 0.7500 | 0.9293 | 0.875 | 1 |
| 14 | 49 | CCAAGGTTTACCCAATAAT | ATTATTGGGTAAACCTTGG | 0.2200 | 0.0087 | 0.8362 | 1.0000 | 0.9293 | 1 | 1 |
| 16 | 48 | CCCAAGGTTTACCCAATAA | TTATTGGGTAAACCTTGGG | 0.1658 | 0.0065 | 0.9569 | 1.0000 | 0.9293 | 1 | 1 |
| 16 | 53 | GGTTTACCCAATAATACTG | CAGTATTATTGGGTAAACC | 0.5274 | 0.0966 | 0.569 | 0.7500 | 0.9946 | 0.75 | 1 |
| 16 | 24 | GGGCGCGATCAAAACAACG | CGTTGTTTTGATCGCGCCC | 0.1313 | 0.0077 | 0.7155 | 0.5000 | 1.0000 | 1 | 1 |
| 17 | 47 | CCCCAAGGTTTACCCAATA | TATTGGGTAAACCTTGGGG | 0.1530 | 0.0053 | 0.9483 | 1.0000 | 0.9293 | 1 | 1 |
| 17 | 87 | CTCTCACTCAACATGGCAA | TTGCCATGTTGAGTGAGAG | 0.1204 | 0.1108 | 0.6638 | 0.7500 | 0.9891 | 1 | 1 |
| 17 | 84 | CCGCTCTCACTCAACATGG | CCATGTTGAGTGAGAGCGG | 0.2246 | 0.2055 | 0.569 | 0.5000 | 0.9891 | 0.625 | 1 |
| 18 | 46 | GCCCCAAGGTTTACCCAAT | ATTGGGTAAACCTTGGGGC | 0.1554 | 0.0048 | 0.9914 | 1.0000 | 0.9293 | 0.875 | 1 |
| 19 | 45 | GGCCCCAAGGTTTACCCAA | TTGGGTAAACCTTGGGGCC | 0.1397 | 0.0041 | 0.8534 | 1.0000 | 0.9293 | 0.75 | 1 |
| 20 | 77 | TGGTTCACCGCTCTCACTC | GAGTGAGAGCGGTGAACCA | 0.5206 | 0.0559 | 0.5345 | 0.5000 | 0.9837 | 0.75 | 1 |
| 20 | 86 | GCTCTCACTCAACATGGCA | TGCCATGTTGAGTGAGAGC | 0.1369 | 0.1238 | 0.5 | 0.7500 | 0.9891 | 0.875 | 1 |
| 21 | 83 | ACCGCTCTCACTCAACATG | CATGTTGAGTGAGAGCGGT | 0.3218 | 0.2695 | 0.6983 | 0.5000 | 0.9946 | 0.75 | 1 |
| 21 | 73 | GTCTTGGTTCACCGCTCTC | GAGAGCGGTGAACCAAGAC | 0.1104 | 0.0032 | 0.5086 | 0.5000 | 0.9837 | 1 | 1 |
| 21 | 74 | TCTTGGTTCACCGCTCTCA | TGAGAGCGGTGAACCAAGA | 0.0960 | 0.0032 | 0.5 | 0.5000 | 0.9837 | 1 | 1 |
| 21 | 92 | ACTCAACATGGCAAGGAAG | CTTCCTTGCCATGTTGAGT | 0.0754 | 0.0495 | 0.569 | 0.5000 | 1.0000 | 1 | 1 |
| 22 | 72 | CGTCTTGGTTCACCGCTCT | AGAGCGGTGAACCAAGACG | 0.1095 | 0.0023 | 0.5517 | 0.7500 | 0.9837 | 1 | 1 |
| 22 | 52 | AGGTTTACCCAATAATACT | AGTATTATTGGGTAAACCT | 0.4029 | 0.0881 | 0.6379 | 0.5000 | 0.9293 | 0.75 | 1 |
| 22 | 79 | GTTCACCGCTCTCACTCAA | TTGAGTGAGAGCGGTGAAC | 0.7717 | 0.0677 | 0.5 | 0.7500 | 1.0000 | 1 | 1 |
| 22 | 91 | CACTCAACATGGCAAGGAA | TTCCTTGCCATGTTGAGTG | 0.0619 | 0.0509 | 0.6207 | 0.7500 | 1.0000 | 1 | 1 |

**List of siRNAs predicted by RNAxs for the ‘conserved region 4’ of the ‘N’ gene**

| Position | Target sequence | siRNA sequence | Access 8nts | Access 16nts | Energy A. | Sequence A. | Self Folding | Free End |  |
| --- | --- | --- | --- | --- | --- | --- | --- | --- | --- |
|  |  |  |  |  |  |  |  |  |  |
| 107 | TGGTGACGGTAAAATGAAA | TTTCATTTTACCGTCACCA | 0.5756 | 0.0431 | 0.8448 | 0.7500 | 1.0000 | 1 | 1 |
| 32 | CGTTCCAATTAACACCAAT | ATTGGTGTTAATTGGAACG | 0.1462 | 0.1593 | 0.7672 | 1.0000 | 1.0000 | 1 | 1 |
| 108 | GGTGACGGTAAAATGAAAG | CTTTCATTTTACCGTCACC | 0.5239 | 0.1060 | 0.7155 | 0.7500 | 1.0000 | 1 | 1 |
| 24 | GGACAAGGCGTTCCAATTA | TAATTGGAACGCCTTGTCC | 0.5125 | 0.0054 | 0.8362 | 1.0000 | 0.9728 | 0.625 | 1 |
| 105 | GGTGGTGACGGTAAAATGA | TCATTTTACCGTCACCACC | 0.1925 | 0.0052 | 0.6293 | 0.7500 | 1.0000 | 1 | 1 |
| 106 | GTGGTGACGGTAAAATGAA | TTCATTTTACCGTCACCAC | 0.2634 | 0.0078 | 0.6207 | 0.7500 | 1.0000 | 1 | 1 |
| 25 | GACAAGGCGTTCCAATTAA | TTAATTGGAACGCCTTGTC | 0.4476 | 0.0149 | 0.7845 | 0.7500 | 0.9891 | 1 | 1 |
| 81 | CGAAGAGCTACCAGACGAA | TTCGTCTGGTAGCTCTTCG | 0.0763 | 0.0044 | 0.6207 | 1.0000 | 1.0000 | 1 | 1 |
| 113 | CGGTAAAATGAAAGATCTC | GAGATCTTTCATTTTACCG | 0.2233 | 0.0552 | 0.6034 | 0.7500 | 1.0000 | 1 | 1 |
| 33 | GTTCCAATTAACACCAATA | TATTGGTGTTAATTGGAAC | 0.1237 | 0.1431 | 0.6034 | 0.7500 | 1.0000 | 1 | 1 |
| 82 | GAAGAGCTACCAGACGAAT | ATTCGTCTGGTAGCTCTTC | 0.0596 | 0.0036 | 0.6638 | 0.7500 | 1.0000 | 1 | 1 |
| 46 | CCAATAGCAGTCCAGATGA | TCATCTGGACTGCTATTGG | 0.0452 | 0.0136 | 0.6207 | 0.7500 | 1.0000 | 1 | 1 |
| 111 | GACGGTAAAATGAAAGATC | GATCTTTCATTTTACCGTC | 0.3027 | 0.0766 | 0.6379 | 0.5000 | 1.0000 | 1 | 1 |
| 23 | AGGACAAGGCGTTCCAATT | AATTGGAACGCCTTGTCCT | 0.3560 | 0.0035 | 0.8362 | 0.7500 | 0.9076 | 0.625 | 1 |
| 103 | GTGGTGGTGACGGTAAAAT | ATTTTACCGTCACCACCAC | 0.1547 | 0.0035 | 0.7845 | 0.7500 | 1.0000 | 1 | 1 |
| 45 | ACCAATAGCAGTCCAGATG | CATCTGGACTGCTATTGGT | 0.0342 | 0.0135 | 0.6983 | 0.5000 | 1.0000 | 1 | 1 |
| 26 | ACAAGGCGTTCCAATTAAC | GTTAATTGGAACGCCTTGT | 0.4779 | 0.0192 | 0.5603 | 0.5000 | 0.9891 | 1 | 1 |
| 44 | CACCAATAGCAGTCCAGAT | ATCTGGACTGCTATTGGTG | 0.0322 | 0.0040 | 0.6121 | 0.7500 | 1.0000 | 1 | 1 |
| 102 | CGTGGTGGTGACGGTAAAA | TTTTACCGTCACCACCACG | 0.1324 | 0.0034 | 0.7759 | 1.0000 | 1.0000 | 1 | 1 |
| 109 | GTGACGGTAAAATGAAAGA | TCTTTCATTTTACCGTCAC | 0.4465 | 0.1306 | 0.569 | 0.5000 | 1.0000 | 1 | 1 |
| 83 | AAGAGCTACCAGACGAATT | AATTCGTCTGGTAGCTCTT | 0.1309 | 0.0026 | 0.6293 | 0.5000 | 1.0000 | 1 | 1 |
| 133 | GTCCAAGATGGTATTTCTA | TAGAAATACCATCTTGGAC | 0.0219 | 0.0028 | 0.6466 | 0.7500 | 0.9674 | 0.75 | 1 |
| 112 | ACGGTAAAATGAAAGATCT | AGATCTTTCATTTTACCGT | 0.2875 | 0.0701 | 0.5517 | 0.5000 | 1.0000 | 1 | 1 |
| 110 | TGACGGTAAAATGAAAGAT | ATCTTTCATTTTACCGTCA | 0.4275 | 0.1332 | 0.5431 | 0.7500 | 1.0000 | 1 | 1 |
| 120 | ATGAAAGATCTCAGTCCAA | TTGGACTGAGATCTTTCAT | 0.1020 | 0.0618 | 0.5086 | 0.5000 | 0.9837 | 0.625 | 1 |
| 22 | GAGGACAAGGCGTTCCAAT | ATTGGAACGCCTTGTCCTC | 0.3124 | 0.0023 | 0.8017 | 0.7500 | 0.9076 | 0.625 | 1 |
| 128 | TCTCAGTCCAAGATGGTAT | ATACCATCTTGGACTGAGA | 0.0164 | 0.0063 | 0.681 | 0.7500 | 0.9674 | 0.875 | 1 |
| 104 | TGGTGGTGACGGTAAAATG | CATTTTACCGTCACCACCA | 0.1577 | 0.0043 | 0.6897 | 0.5000 | 1.0000 | 1 | 1 |
| 36 | CCAATTAACACCAATAGCA | TGCTATTGGTGTTAATTGG | 0.0202 | 0.0138 | 0.4914 | 0.7500 | 1.0000 | 1 | 1 |
| 134 | TCCAAGATGGTATTTCTAC | GTAGAAATACCATCTTGGA | 0.0156 | 0.0024 | 0.6897 | 0.5000 | 0.9674 | 0.625 | 1 |
| 121 | TGAAAGATCTCAGTCCAAG | CTTGGACTGAGATCTTTCA | 0.0981 | 0.0515 | 0.5862 | 0.5000 | 1.0000 | 1 | 1 |
| 84 | AGAGCTACCAGACGAATTC | GAATTCGTCTGGTAGCTCT | 0.1152 | 0.0017 | 0.5517 | 0.5000 | 1.0000 | 1 | 1 |
| 135 | CCAAGATGGTATTTCTACT | AGTAGAAATACCATCTTGG | 0.0138 | 0.0020 | 0.6379 | 0.7500 | 0.9946 | 0.625 | 1 |
| 43 | ACACCAATAGCAGTCCAGA | TCTGGACTGCTATTGGTGT | 0.0281 | 0.0035 | 0.4828 | 0.5000 | 1.0000 | 1 | 1 |

**List of siRNAs predicted by RNAxs for the ‘conserved region 7’ of the ‘N’ gene**

| **WORST RANK** | **Position** | **Target sequence** | **siRNA sequence** | **Access 8nts** | **Access 16nts** | **Energy A.** | **Sequence A.** | **Self-Folding** | **Free End** |  |
| --- | --- | --- | --- | --- | --- | --- | --- | --- | --- | --- |
|  |  |  |  |  |  |  |  |  |  |  |
| 3 | 65 | AGGAACAACATTGCCAAAA | TTTTGGCAATGTTGTTCCT | 0.0814 | 0.0119 | 0.8448 | 0.7500 | 1.0000 | 1 | 1 |
| 4 | 66 | GGAACAACATTGCCAAAAG | CTTTTGGCAATGTTGTTCC | 0.0814 | 0.0303 | 0.7328 | 0.7500 | 1.0000 | 1 | 1 |
| 9 | 22 | GCAATCCTGCTAACAATGC | GCATTGTTAGCAGGATTGC | 0.3539 | 0.0490 | 0.5 | 0.5000 | 0.9674 | 0.75 | 1 |
| 10 | 59 | TCCTCAAGGAACAACATTG | CAATGTTGTTCCTTGAGGA | 0.1271 | 0.0022 | 0.7241 | 0.5000 | 0.9565 | 0.625 | 1 |
| 11 | 47 | CGTGCTACAACTTCCTCAA | TTGAGGAAGTTGTAGCACG | 0.0232 | 0.0058 | 0.6293 | 1.0000 | 1.0000 | 1 | 1 |
| 11 | 40 | CTGCAATCGTGCTACAACT | AGTTGTAGCACGATTGCAG | 0.0662 | 0.0112 | 0.5776 | 0.5000 | 0.9130 | 0.75 | 1 |
| 11 | 64 | AAGGAACAACATTGCCAAA | TTTGGCAATGTTGTTCCTT | 0.0808 | 0.0020 | 0.6379 | 0.5000 | 1.0000 | 1 | 1 |
| 12 | 30 | GCTAACAATGCTGCAATCG | CGATTGCAGCATTGTTAGC | 0.0290 | 0.0054 | 0.5603 | 0.5000 | 1.0000 | 1 | 1 |
| 12 | 48 | GTGCTACAACTTCCTCAAG | CTTGAGGAAGTTGTAGCAC | 0.0170 | 0.0057 | 0.6552 | 0.5000 | 1.0000 | 1 | 1 |
| 12 | 60 | CCTCAAGGAACAACATTGC | GCAATGTTGTTCCTTGAGG | 0.0815 | 0.0015 | 0.4914 | 0.5000 | 1.0000 | 1 | 1 |
| 13 | 46 | TCGTGCTACAACTTCCTCA | TGAGGAAGTTGTAGCACGA | 0.0418 | 0.0029 | 0.569 | 0.5000 | 1.0000 | 1 | 1 |
| 13 | 63 | CAAGGAACAACATTGCCAA | TTGGCAATGTTGTTCCTTG | 0.0807 | 0.0015 | 0.5 | 0.7500 | 1.0000 | 1 | 1 |
| 13 | 35 | CAATGCTGCAATCGTGCTA | TAGCACGATTGCAGCATTG | 0.0766 | 0.0057 | 0.4741 | 0.7500 | 0.9130 | 0.75 | 1 |
| 13 | 50 | GCTACAACTTCCTCAAGGA | TCCTTGAGGAAGTTGTAGC | 0.0165 | 0.0108 | 0.4828 | 0.7500 | 0.9239 | 0.625 | 1 |

**List of siRNAs predicted by RNAxs for the ‘conserved region 10’ of the ‘N’ gene**

| **WORST RANK** | **Position** | **Target sequence** | **siRNA sequence** | **Access 8nts** | **Access 16nts** | **Energy A.** | **Sequence A.** | **Self-Folding** | **Free End** |  |
| --- | --- | --- | --- | --- | --- | --- | --- | --- | --- | --- |
|  |  |  |  |  |  |  |  |  |  |  |
| 4 | 42 | GCCAAACTGTCACTAAGAA | TTCTTAGTGACAGTTTGGC | 0.7634 | 0.3631 | 0.8276 | 1.0000 | 1.0000 | 1 | 1 |
| 5 | 43 | CCAAACTGTCACTAAGAAA | TTTCTTAGTGACAGTTTGG | 0.7659 | 0.3739 | 0.8448 | 1.0000 | 1.0000 | 1 | 1 |
| 16 | 196 | AGGAACTGATTACAAACAT | ATGTTTGTAATCAGTTCCT | 0.2693 | 0.1073 | 0.6897 | 0.7500 | 1.0000 | 1 | 1 |
| 25 | 41 | GGCCAAACTGTCACTAAGA | TCTTAGTGACAGTTTGGCC | 0.5745 | 0.1599 | 0.7759 | 0.7500 | 1.0000 | 1 | 1 |
| 26 | 192 | GACAAGGAACTGATTACAA | TTGTAATCAGTTCCTTGTC | 0.1524 | 0.0616 | 0.6724 | 0.7500 | 1.0000 | 1 | 1 |
| 27 | 28 | CCAACAACAACAAGGCCAA | TTGGCCTTGTTGTTGTTGG | 0.0817 | 0.0394 | 0.6983 | 1.0000 | 1.0000 | 1 | 1 |
| 27 | 197 | GGAACTGATTACAAACATT | AATGTTTGTAATCAGTTCC | 0.2199 | 0.0227 | 0.8621 | 1.0000 | 1.0000 | 1 | 1 |
| 28 | 193 | ACAAGGAACTGATTACAAA | TTTGTAATCAGTTCCTTGT | 0.1907 | 0.0745 | 0.7069 | 0.7500 | 1.0000 | 1 | 1 |
| 29 | 29 | CAACAACAACAAGGCCAAA | TTTGGCCTTGTTGTTGTTG | 0.0914 | 0.0456 | 0.6466 | 0.7500 | 1.0000 | 1 | 1 |
| 29 | 44 | CAAACTGTCACTAAGAAAT | ATTTCTTAGTGACAGTTTG | 0.7708 | 0.3844 | 0.6379 | 0.7500 | 1.0000 | 1 | 1 |
| 31 | 139 | CAGACGTGGTCCAGAACAA | TTGTTCTGGACCACGTCTG | 0.5107 | 0.0738 | 0.6379 | 0.7500 | 0.9130 | 0.625 | 1 |
| 31 | 113 | GCATACAATGTAACACAAG | CTTGTGTTACATTGTATGC | 0.1007 | 0.0142 | 0.7155 | 0.7500 | 1.0000 | 1 | 1 |
| 34 | 107 | ACTAAAGCATACAATGTAA | TTACATTGTATGCTTTAGT | 0.6857 | 0.0086 | 0.6724 | 0.7500 | 1.0000 | 1 | 1 |
| 34 | 39 | AAGGCCAAACTGTCACTAA | TTAGTGACAGTTTGGCCTT | 0.4866 | 0.0844 | 0.6034 | 0.5000 | 1.0000 | 1 | 1 |
| 35 | 117 | ACAATGTAACACAAGCTTT | AAAGCTTGTGTTACATTGT | 0.0561 | 0.0354 | 0.7069 | 0.7500 | 0.9946 | 0.75 | 1 |
| 36 | 190 | CAGACAAGGAACTGATTAC | GTAATCAGTTCCTTGTCTG | 0.1253 | 0.0370 | 0.6034 | 0.5000 | 0.9783 | 0.625 | 1 |
| 38 | 116 | TACAATGTAACACAAGCTT | AAGCTTGTGTTACATTGTA | 0.0723 | 0.0462 | 0.5431 | 0.5000 | 0.9946 | 0.625 | 1 |
| 39 | 108 | CTAAAGCATACAATGTAAC | GTTACATTGTATGCTTTAG | 0.7966 | 0.0113 | 0.5259 | 0.5000 | 1.0000 | 1 | 1 |
| 40 | 207 | ACAAACATTGGCCGCAAAT | ATTTGCGGCCAATGTTTGT | 0.0477 | 0.0059 | 0.6983 | 0.7500 | 1.0000 | 1 | 1 |
| 40 | 152 | GAACAAACCCAAGGAAATT | AATTTCCTTGGGTTTGTTC | 0.0432 | 0.0063 | 0.6638 | 0.7500 | 1.0000 | 1 | 1 |
| 41 | 141 | GACGTGGTCCAGAACAAAC | GTTTGTTCTGGACCACGTC | 0.3887 | 0.0326 | 0.6724 | 0.5000 | 1.0000 | 1 | 1 |
| 41 | 94 | AAAACGTACTGCCACTAAA | TTTAGTGGCAGTACGTTTT | 0.0429 | 0.0387 | 0.5 | 0.5000 | 0.9946 | 1 | 1 |
| 42 | 40 | AGGCCAAACTGTCACTAAG | CTTAGTGACAGTTTGGCCT | 0.5050 | 0.1100 | 0.7069 | 0.5000 | 1.0000 | 1 | 1 |
| 42 | 179 | CAGGAACTAATCAGACAAG | CTTGTCTGATTAGTTCCTG | 0.0742 | 0.0046 | 0.6466 | 0.5000 | 1.0000 | 1 | 1 |
| 43 | 151 | AGAACAAACCCAAGGAAAT | ATTTCCTTGGGTTTGTTCT | 0.0364 | 0.0075 | 0.7155 | 0.7500 | 1.0000 | 1 | 1 |
| 43 | 182 | GAACTAATCAGACAAGGAA | TTCCTTGTCTGATTAGTTC | 0.9838 | 0.0404 | 0.5 | 0.7500 | 1.0000 | 1 | 1 |
| 43 | 178 | CCAGGAACTAATCAGACAA | TTGTCTGATTAGTTCCTGG | 0.0865 | 0.0039 | 0.6983 | 1.0000 | 1.0000 | 1 | 1 |
| 44 | 150 | CAGAACAAACCCAAGGAAA | TTTCCTTGGGTTTGTTCTG | 0.0348 | 0.0077 | 0.7845 | 0.7500 | 1.0000 | 1 | 1 |
| 44 | 181 | GGAACTAATCAGACAAGGA | TCCTTGTCTGATTAGTTCC | 0.0752 | 0.0534 | 0.5 | 0.7500 | 1.0000 | 1 | 1 |
| 45 | 31 | ACAACAACAAGGCCAAACT | AGTTTGGCCTTGTTGTTGT | 0.1103 | 0.0502 | 0.5 | 0.5000 | 1.0000 | 1 | 1 |
| 46 | 153 | AACAAACCCAAGGAAATTT | AAATTTCCTTGGGTTTGTT | 0.0346 | 0.0062 | 0.6466 | 0.5000 | 1.0000 | 1 | 1 |
| 46 | 79 | TAAGAAGCCTCGGCAAAAA | TTTTTGCCGAGGCTTCTTA | 0.0793 | 0.0023 | 0.5345 | 0.5000 | 0.9402 | 1 | 1 |
| 46 | 184 | ACTAATCAGACAAGGAACT | AGTTCCTTGTCTGATTAGT | 0.0704 | 0.0144 | 0.5 | 0.5000 | 1.0000 | 1 | 1 |
| 47 | 194 | CAAGGAACTGATTACAAAC | GTTTGTAATCAGTTCCTTG | 0.2004 | 0.0853 | 0.5 | 0.5000 | 1.0000 | 1 | 1 |
| 47 | 78 | CTAAGAAGCCTCGGCAAAA | TTTTGCCGAGGCTTCTTAG | 0.0557 | 0.0020 | 0.6724 | 0.7500 | 0.9402 | 1 | 1 |
| 47 | 149 | CCAGAACAAACCCAAGGAA | TTCCTTGGGTTTGTTCTGG | 0.0208 | 0.0085 | 0.6724 | 1.0000 | 1.0000 | 1 | 1 |
| 48 | 198 | GAACTGATTACAAACATTG | CAATGTTTGTAATCAGTTC | 0.1007 | 0.0032 | 0.5259 | 0.5000 | 1.0000 | 1 | 1 |
| 48 | 208 | CAAACATTGGCCGCAAATT | AATTTGCGGCCAATGTTTG | 0.0458 | 0.0019 | 0.6379 | 0.7500 | 1.0000 | 1 | 1 |
| 48 | 155 | CAAACCCAAGGAAATTTTG | CAAAATTTCCTTGGGTTTG | 0.0189 | 0.0026 | 0.5 | 0.5000 | 1.0000 | 1 | 1 |
| 49 | 191 | AGACAAGGAACTGATTACA | TGTAATCAGTTCCTTGTCT | 0.1376 | 0.0489 | 0.5172 | 0.5000 | 1.0000 | 1 | 1 |
| 49 | 77 | TCTAAGAAGCCTCGGCAAA | TTTGCCGAGGCTTCTTAGA | 0.0420 | 0.0017 | 0.7241 | 0.7500 | 0.9402 | 0.875 | 1 |
| 49 | 38 | CAAGGCCAAACTGTCACTA | TAGTGACAGTTTGGCCTTG | 0.4657 | 0.0686 | 0.4741 | 0.7500 | 1.0000 | 1 | 1 |
| 49 | 154 | ACAAACCCAAGGAAATTTT | AAAATTTCCTTGGGTTTGT | 0.0181 | 0.0047 | 0.7069 | 0.7500 | 1.0000 | 1 | 1 |
| 50 | 37 | ACAAGGCCAAACTGTCACT | AGTGACAGTTTGGCCTTGT | 0.4440 | 0.0647 | 0.5 | 0.5000 | 1.0000 | 1 | 1 |
| 50 | 270 | GCATTGGCATGGAAGTCAC | GTGACTTCCATGCCAATGC | 0.0176 | 0.0152 | 0.5603 | 0.7500 | 1.0000 | 1 | 1 |
| 50 | 76 | TTCTAAGAAGCCTCGGCAA | TTGCCGAGGCTTCTTAGAA | 0.0265 | 0.0015 | 0.5259 | 0.5000 | 0.9402 | 0.75 | 1 |
| 51 | 92 | CAAAAACGTACTGCCACTA | TAGTGGCAGTACGTTTTTG | 0.0663 | 0.0603 | 0.4741 | 0.7500 | 0.9946 | 0.75 | 1 |
| 51 | 138 | GCAGACGTGGTCCAGAACA | TGTTCTGGACCACGTCTGC | 0.2085 | 0.0013 | 0.6466 | 0.7500 | 0.9130 | 0.75 | 1 |
| 51 | 269 | CGCATTGGCATGGAAGTCA | TGACTTCCATGCCAATGCG | 0.0175 | 0.0152 | 0.6983 | 0.7500 | 0.9946 | 0.75 | 1 |
| 52 | 118 | CAATGTAACACAAGCTTTC | GAAAGCTTGTGTTACATTG | 0.0489 | 0.0280 | 0.4741 | 0.5000 | 0.9946 | 0.875 | 1 |
| 52 | 177 | ACCAGGAACTAATCAGACA | TGTCTGATTAGTTCCTGGT | 0.1018 | 0.0012 | 0.6466 | 0.5000 | 1.0000 | 1 | 1 |
| 52 | 93 | AAAAACGTACTGCCACTAA | TTAGTGGCAGTACGTTTTT | 0.0623 | 0.0481 | 0.4655 | 0.5000 | 0.9946 | 0.875 | 1 |
| 52 | 206 | TACAAACATTGGCCGCAAA | TTTGCGGCCAATGTTTGTA | 0.0156 | 0.0101 | 0.681 | 0.5000 | 1.0000 | 1 | 1 |

**List of siRNAs predicted by RNAxs for the ‘conserved region 11’ of the ‘N’ gene**

| **WORST RANK** | **Position** | **Target sequence** | **siRNA sequence** | **Access 8nts** | **Access 16nts** | **Energy A.** | **Sequence A.** | **Self-Folding** | **Free End** |  |
| --- | --- | --- | --- | --- | --- | --- | --- | --- | --- | --- |
|  |  |  |  |  |  |  |  |  |  |  |
| 6 | 66 | TCCAAATTTCAAAGATCAA | TTGATCTTTGAAATTTGGA | 0.0784 | 0.0030 | 0.7241 | 0.7500 | 1.0000 | 1 | 1 |
| 7 | 63 | AGATCCAAATTTCAAAGAT | ATCTTTGAAATTTGGATCT | 0.1131 | 0.0014 | 0.5431 | 0.7500 | 1.0000 | 1 | 1 |
| 7 | 67 | CCAAATTTCAAAGATCAAG | CTTGATCTTTGAAATTTGG | 0.0747 | 0.0113 | 0.7069 | 0.7500 | 1.0000 | 1 | 1 |
| 8 | 80 | ATCAAGTCATTTTGCTGAA | TTCAGCAAAATGACTTGAT | 0.2494 | 0.0384 | 0.5086 | 0.5000 | 0.9728 | 0.625 | 1 |
| 8 | 60 | CAAAGATCCAAATTTCAAA | TTTGAAATTTGGATCTTTG | 0.6407 | 0.0013 | 0.6466 | 0.7500 | 1.0000 | 1 | 1 |
| 9 | 59 | ACAAAGATCCAAATTTCAA | TTGAAATTTGGATCTTTGT | 0.5092 | 0.0013 | 0.5603 | 0.7500 | 1.0000 | 1 | 1 |
| 9 | 75 | CAAAGATCAAGTCATTTTG | CAAAATGACTTGATCTTTG | 0.0632 | 0.0414 | 0.5 | 0.5000 | 1.0000 | 1 | 1 |
| 9 | 73 | TTCAAAGATCAAGTCATTT | AAATGACTTGATCTTTGAA | 0.0536 | 0.0370 | 0.6724 | 0.5000 | 1.0000 | 1 | 1 |
| 10 | 58 | GACAAAGATCCAAATTTCA | TGAAATTTGGATCTTTGTC | 0.4648 | 0.0013 | 0.5948 | 0.5000 | 1.0000 | 1 | 1 |
| 10 | 74 | TCAAAGATCAAGTCATTTT | AAAATGACTTGATCTTTGA | 0.0500 | 0.0413 | 0.7241 | 0.7500 | 1.0000 | 1 | 1 |
| 10 | 72 | TTTCAAAGATCAAGTCATT | AATGACTTGATCTTTGAAA | 0.0846 | 0.0366 | 0.4914 | 0.5000 | 1.0000 | 1 | 1 |

**List of siRNAs predicted by RNAxs for the ‘conserved region 14’ of the ‘N’ gene**

| **WORST RANK** | **Position** | **Target sequence** | **siRNA sequence** | **Access 8nts** | **Access 16nts** | **Energy A.** | **Sequence A.** | **Self-Folding** | **Free End** |  |
| --- | --- | --- | --- | --- | --- | --- | --- | --- | --- | --- |
|  |  |  |  |  |  |  |  |  |  |  |
| 4 | 78 | GCTGCAGATTTGGATGATT | AATCATCCAAATCTGCAGC | 0.0278 | 0.0195 | 0.8448 | 1.0000 | 1.0000 | 1 | 1 |
| 5 | 77 | TGCTGCAGATTTGGATGAT | ATCATCCAAATCTGCAGCA | 0.0278 | 0.0187 | 0.6724 | 0.7500 | 1.0000 | 1 | 1 |
| 6 | 81 | GCAGATTTGGATGATTTCT | AGAAATCATCCAAATCTGC | 0.0603 | 0.0120 | 0.6293 | 0.7500 | 1.0000 | 1 | 1 |
| 6 | 79 | CTGCAGATTTGGATGATTT | AAATCATCCAAATCTGCAG | 0.0245 | 0.0195 | 0.7845 | 0.7500 | 1.0000 | 1 | 1 |
| 6 | 80 | TGCAGATTTGGATGATTTC | GAAATCATCCAAATCTGCA | 0.0605 | 0.0195 | 0.681 | 0.5000 | 1.0000 | 1 | 1 |
| 7 | 75 | CCTGCTGCAGATTTGGATG | CATCCAAATCTGCAGCAGG | 0.0279 | 0.0021 | 0.6897 | 0.7500 | 0.9837 | 0.625 | 1 |
| 8 | 74 | TCCTGCTGCAGATTTGGAT | ATCCAAATCTGCAGCAGGA | 0.0225 | 0.0013 | 0.6897 | 0.7500 | 0.9837 | 0.75 | 1 |
| 8 | 76 | CTGCTGCAGATTTGGATGA | TCATCCAAATCTGCAGCAG | 0.0279 | 0.0187 | 0.5603 | 0.5000 | 1.0000 | 1 | 1 |
| 9 | 71 | TCTTCCTGCTGCAGATTTG | CAAATCTGCAGCAGGAAGA | 0.0240 | 0.0012 | 0.5776 | 0.5000 | 0.9837 | 1 | 1 |
| 9 | 82 | CAGATTTGGATGATTTCTC | GAGAAATCATCCAAATCTG | 0.0151 | 0.0075 | 0.4741 | 0.5000 | 1.0000 | 1 | 1 |
