# Supplementary material for: Design of siRNA molecules for silencing of membrane glycoprotein, nucleocapsid phosphoprotein, and surface glycoprotein genes of SARS-CoV2
Source: J Genet Eng Biotechnol. 2022 Apr 28;20:65. doi: 10.1186/s43141-022-00346-z (PMC9047631; doi:10.1186/s43141-022-00346-z)
Supplement: Supplementary file 6 — Additional file 6: Supplementary Table 6. List of siRNAs predicted by RNAxs for various conserved regions of ‘S’ gene. [file 43141_2022_346_MOESM6_ESM.docx]

**Supplementary Table 6:** List of siRNAs predicted by RNAxs for various conserved regions of ‘S’ gene

**List of siRNAs predicted by RNAxs for the ‘conserved region 1’ of the ‘S’ gene**

| **WORST RANK** | **Position** | **Target sequence** | **siRNA sequence** | **Access 8nts** | **Access 16nts** | **Energy A.** | **Sequence A.** | **Self-Folding** | **Free End** |  |
| --- | --- | --- | --- | --- | --- | --- | --- | --- | --- | --- |
|  |  |  |  |  |  |  |  |  |  |  |
| 7 | 92 | AGATCCTCAGTTTTACATT | AATGTAAAACTGAGGATCT | 0.8030 | 0.7260 | 0.7155 | 0.7500 | 1.0000 | 1 | 1 |
| 11 | 95 | TCCTCAGTTTTACATTCAA | TTGAATGTAAAACTGAGGA | 0.6972 | 0.6292 | 0.7241 | 0.7500 | 1.0000 | 1 | 1 |
| 13 | 96 | CCTCAGTTTTACATTCAAC | GTTGAATGTAAAACTGAGG | 0.6163 | 0.5558 | 0.6983 | 0.7500 | 0.9837 | 0.625 | 1 |
| 14 | 91 | CAGATCCTCAGTTTTACAT | ATGTAAAACTGAGGATCTG | 0.8154 | 0.7432 | 0.6293 | 0.7500 | 1.0000 | 1 | 1 |
| 14 | 39 | CTGCATACACTAATTCTTT | AAAGAATTAGTGTATGCAG | 0.3965 | 0.1779 | 0.7845 | 0.7500 | 1.0000 | 1 | 1 |
| 15 | 41 | GCATACACTAATTCTTTCA | TGAAAGAATTAGTGTATGC | 0.7422 | 0.1635 | 0.6293 | 0.7500 | 1.0000 | 1 | 1 |
| 16 | 27 | CTCAATTACCCCCTGCATA | TATGCAGGGGGTAATTGAG | 0.3072 | 0.2795 | 0.7672 | 0.7500 | 1.0000 | 1 | 1 |
| 16 | 38 | CCTGCATACACTAATTCTT | AAGAATTAGTGTATGCAGG | 0.3351 | 0.1522 | 0.7069 | 1.0000 | 1.0000 | 1 | 1 |
| 18 | 26 | ACTCAATTACCCCCTGCAT | ATGCAGGGGGTAATTGAGT | 0.6576 | 0.6007 | 0.5517 | 0.7500 | 1.0000 | 1 | 1 |
| 19 | 34 | ACCCCCTGCATACACTAAT | ATTAGTGTATGCAGGGGGT | 0.2178 | 0.1763 | 0.8448 | 0.7500 | 1.0000 | 1 | 1 |
| 19 | 28 | TCAATTACCCCCTGCATAC | GTATGCAGGGGGTAATTGA | 0.2448 | 0.2212 | 0.5431 | 0.5000 | 1.0000 | 1 | 1 |
| 20 | 35 | CCCCCTGCATACACTAATT | AATTAGTGTATGCAGGGGG | 0.2174 | 0.1767 | 0.9828 | 1.0000 | 1.0000 | 1 | 1 |
| 21 | 86 | GTTTTCAGATCCTCAGTTT | AAACTGAGGATCTGAAAAC | 0.8047 | 0.0398 | 0.6466 | 0.7500 | 1.0000 | 1 | 1 |
| 21 | 37 | CCCTGCATACACTAATTCT | AGAATTAGTGTATGCAGGG | 0.2005 | 0.1533 | 0.7672 | 0.7500 | 1.0000 | 1 | 1 |
| 22 | 85 | AGTTTTCAGATCCTCAGTT | AACTGAGGATCTGAAAACT | 0.8133 | 0.0387 | 0.5603 | 0.7500 | 1.0000 | 1 | 1 |
| 23 | 36 | CCCCTGCATACACTAATTC | GAATTAGTGTATGCAGGGG | 0.1970 | 0.1533 | 0.819 | 0.7500 | 1.0000 | 1 | 1 |
| 23 | 40 | TGCATACACTAATTCTTTC | GAAAGAATTAGTGTATGCA | 0.7540 | 0.1754 | 0.681 | 0.5000 | 1.0000 | 1 | 1 |
| 23 | 93 | GATCCTCAGTTTTACATTC | GAATGTAAAACTGAGGATC | 0.9436 | 0.7042 | 0.5086 | 0.5000 | 1.0000 | 1 | 1 |
| 24 | 90 | TCAGATCCTCAGTTTTACA | TGTAAAACTGAGGATCTGA | 0.7707 | 0.0809 | 0.5172 | 0.5000 | 1.0000 | 1 | 1 |
| 25 | 46 | CACTAATTCTTTCACACGT | ACGTGTGAAAGAATTAGTG | 0.2002 | 0.1314 | 0.5172 | 0.5000 | 1.0000 | 1 | 1 |
| 25 | 87 | TTTTCAGATCCTCAGTTTT | AAAACTGAGGATCTGAAAA | 0.7957 | 0.0494 | 0.5 | 0.5000 | 1.0000 | 1 | 1 |
| 26 | 33 | TACCCCCTGCATACACTAA | TTAGTGTATGCAGGGGGTA | 0.2188 | 0.1757 | 0.6466 | 0.5000 | 1.0000 | 1 | 1 |
| 26 | 72 | ATTACCCTGACAAAGTTTT | AAAACTTTGTCAGGGTAAT | 0.0698 | 0.0072 | 0.5086 | 0.5000 | 1.0000 | 1 | 1 |
| 26 | 58 | CACACGTGGTGTTTATTAC | GTAATAAACACCACGTGTG | 0.1118 | 0.0019 | 0.6121 | 0.5000 | 0.9076 | 0.625 | 1 |
| 26 | 80 | GACAAAGTTTTCAGATCCT | AGGATCTGAAAACTTTGTC | 0.0949 | 0.0197 | 0.4741 | 0.5000 | 1.0000 | 1 | 1 |
| 27 | 57 | TCACACGTGGTGTTTATTA | TAATAAACACCACGTGTGA | 0.0145 | 0.0019 | 0.6897 | 0.7500 | 0.9076 | 0.75 | 1 |
| 27 | 71 | TATTACCCTGACAAAGTTT | AAACTTTGTCAGGGTAATA | 0.8083 | 0.0071 | 0.5431 | 0.5000 | 1.0000 | 1 | 1 |
| 27 | 88 | TTTCAGATCCTCAGTTTTA | TAAAACTGAGGATCTGAAA | 0.7864 | 0.0767 | 0.4655 | 0.5000 | 1.0000 | 1 | 1 |

**List of siRNAs predicted by RNAxs for the ‘conserved region 5’ of the ‘S’ gene**

| **WORST RANK** | **Position** | **Target sequence** | **siRNA sequence** | **Access 8nts** | **Access 16nts** | **Energy A.** | **Sequence A.** | **Self-Folding** | **Free End** |  |
| --- | --- | --- | --- | --- | --- | --- | --- | --- | --- | --- |
|  |  |  |  |  |  |  |  |  |  |  |
| 6 | 76 | TGAATTTCAATTTTGTAAT | ATTACAAAATTGAAATTCA | 0.4035 | 0.2699 | 0.7155 | 0.7500 | 1.0000 | 1 | 1 |
| 7 | 75 | GTGAATTTCAATTTTGTAA | TTACAAAATTGAAATTCAC | 0.4732 | 0.2654 | 0.7586 | 0.7500 | 1.0000 | 1 | 1 |
| 8 | 22 | GACCCAGTCCCTACTTATT | AATAAGTAGGGACTGGGTC | 0.3887 | 0.2353 | 0.8103 | 0.7500 | 1.0000 | 1 | 1 |
| 10 | 71 | GTCTGTGAATTTCAATTTT | AAAATTGAAATTCACAGAC | 0.3423 | 0.2411 | 0.819 | 0.7500 | 1.0000 | 1 | 1 |
| 12 | 74 | TGTGAATTTCAATTTTGTA | TACAAAATTGAAATTCACA | 0.4807 | 0.2594 | 0.5259 | 0.7500 | 1.0000 | 1 | 1 |
| 13 | 70 | AGTCTGTGAATTTCAATTT | AAATTGAAATTCACAGACT | 0.3212 | 0.2247 | 0.7069 | 0.7500 | 1.0000 | 1 | 1 |
| 14 | 77 | GAATTTCAATTTTGTAATG | CATTACAAAATTGAAATTC | 0.3430 | 0.2489 | 0.5172 | 0.5000 | 1.0000 | 1 | 1 |
| 15 | 23 | ACCCAGTCCCTACTTATTG | CAATAAGTAGGGACTGGGT | 0.3800 | 0.2441 | 0.7069 | 0.5000 | 1.0000 | 1 | 1 |
| 16 | 72 | TCTGTGAATTTCAATTTTG | CAAAATTGAAATTCACAGA | 0.3382 | 0.2389 | 0.5776 | 0.5000 | 1.0000 | 1 | 1 |
| 16 | 86 | TTTTGTAATGATCCATTTT | AAAATGGATCATTACAAAA | 0.3322 | 0.2013 | 0.5 | 0.5000 | 1.0000 | 1 | 1 |
| 17 | 69 | AAGTCTGTGAATTTCAATT | AATTGAAATTCACAGACTT | 0.3550 | 0.1559 | 0.6293 | 0.5000 | 1.0000 | 1 | 1 |
| 17 | 84 | AATTTTGTAATGATCCATT | AATGGATCATTACAAAATT | 0.3062 | 0.2233 | 0.5 | 0.5000 | 1.0000 | 1 | 1 |
| 17 | 85 | ATTTTGTAATGATCCATTT | AAATGGATCATTACAAAAT | 0.2998 | 0.2138 | 0.5086 | 0.5000 | 1.0000 | 1 | 1 |
| 18 | 87 | TTTGTAATGATCCATTTTT | AAAAATGGATCATTACAAA | 0.3509 | 0.1818 | 0.5 | 0.5000 | 1.0000 | 1 | 1 |
| 19 | 24 | CCCAGTCCCTACTTATTGT | ACAATAAGTAGGGACTGGG | 0.1196 | 0.0828 | 0.7845 | 0.7500 | 1.0000 | 1 | 1 |
| 19 | 63 | TTATTAAAGTCTGTGAATT | AATTCACAGACTTTAATAA | 0.3709 | 0.0051 | 0.5259 | 0.5000 | 1.0000 | 1 | 1 |
| 19 | 68 | AAAGTCTGTGAATTTCAAT | ATTGAAATTCACAGACTTT | 0.3190 | 0.1410 | 0.4914 | 0.5000 | 1.0000 | 1 | 1 |
| 20 | 73 | CTGTGAATTTCAATTTTGT | ACAAAATTGAAATTCACAG | 0.3601 | 0.2654 | 0.5776 | 0.5000 | 1.0000 | 1 | 1 |
| 20 | 64 | TATTAAAGTCTGTGAATTT | AAATTCACAGACTTTAATA | 0.3013 | 0.0049 | 0.5431 | 0.5000 | 1.0000 | 1 | 1 |
| 20 | 83 | CAATTTTGTAATGATCCAT | ATGGATCATTACAAAATTG | 0.2872 | 0.2315 | 0.4914 | 0.7500 | 1.0000 | 1 | 1 |
| 20 | 25 | CCAGTCCCTACTTATTGTT | AACAATAAGTAGGGACTGG | 0.0203 | 0.0145 | 0.6983 | 1.0000 | 1.0000 | 1 | 1 |

**List of siRNAs predicted by RNAxs for the ‘conserved region 6’ of the ‘S’ gene**

| **WORST RANK** | **Position** | **Target sequence** | **siRNA sequence** | **Access 8nts** | **Access 16nts** | **Energy A.** | **Sequence A.** | **Self-Folding** | **Free End** |  |
| --- | --- | --- | --- | --- | --- | --- | --- | --- | --- | --- |
|  |  |  |  |  |  |  |  |  |  |  |
| 14 | 96 | GGTAATTTCAAAAATCTTA | TAAGATTTTTGAAATTACC | 0.3232 | 0.0976 | 0.819 | 1.0000 | 1.0000 | 1 | 1 |
| 14 | 57 | GTCTCTCAGCCTTTTCTTA | TAAGAAAAGGCTGAGAGAC | 0.5272 | 0.1312 | 0.7845 | 0.7500 | 1.0000 | 1 | 1 |
| 16 | 58 | TCTCTCAGCCTTTTCTTAT | ATAAGAAAAGGCTGAGAGA | 0.5347 | 0.1254 | 0.681 | 0.7500 | 1.0000 | 1 | 1 |
| 27 | 42 | TGCACTTTTGAATATGTCT | AGACATATTCAAAAGTGCA | 0.4457 | 0.1404 | 0.6293 | 0.5000 | 1.0000 | 1 | 1 |
| 28 | 32 | TGCGAATAATTGCACTTTT | AAAAGTGCAATTATTCGCA | 0.1859 | 0.0387 | 0.8534 | 0.7500 | 0.9348 | 0.625 | 1 |
| 30 | 95 | GGGTAATTTCAAAAATCTT | AAGATTTTTGAAATTACCC | 0.3299 | 0.0228 | 0.8534 | 1.0000 | 1.0000 | 1 | 1 |
| 31 | 44 | CACTTTTGAATATGTCTCT | AGAGACATATTCAAAAGTG | 0.3898 | 0.1806 | 0.569 | 0.5000 | 1.0000 | 1 | 1 |
| 32 | 56 | TGTCTCTCAGCCTTTTCTT | AAGAAAAGGCTGAGAGACA | 0.2415 | 0.1250 | 0.569 | 0.7500 | 1.0000 | 1 | 1 |
| 33 | 34 | CGAATAATTGCACTTTTGA | TCAAAAGTGCAATTATTCG | 0.1698 | 0.0566 | 0.569 | 0.7500 | 1.0000 | 1 | 1 |
| 33 | 33 | GCGAATAATTGCACTTTTG | CAAAAGTGCAATTATTCGC | 0.1396 | 0.0423 | 0.7759 | 0.7500 | 1.0000 | 1 | 1 |
| 33 | 94 | AGGGTAATTTCAAAAATCT | AGATTTTTGAAATTACCCT | 0.3320 | 0.0134 | 0.6207 | 0.5000 | 1.0000 | 1 | 1 |
| 34 | 43 | GCACTTTTGAATATGTCTC | GAGACATATTCAAAAGTGC | 0.4781 | 0.1548 | 0.5431 | 0.7500 | 1.0000 | 1 | 1 |
| 35 | 134 | TATTGATGGTTATTTTAAA | TTTAAAATAACCATCAATA | 0.3285 | 0.1368 | 0.5431 | 0.5000 | 1.0000 | 1 | 1 |
| 35 | 137 | TGATGGTTATTTTAAAATA | TATTTTAAAATAACCATCA | 0.0871 | 0.0765 | 0.681 | 0.7500 | 1.0000 | 1 | 1 |
| 36 | 103 | TCAAAAATCTTAGGGAATT | AATTCCCTAAGATTTTTGA | 0.0561 | 0.0391 | 0.7155 | 0.7500 | 1.0000 | 1 | 1 |
| 37 | 92 | ACAGGGTAATTTCAAAAAT | ATTTTTGAAATTACCCTGT | 0.1666 | 0.0080 | 0.6983 | 0.7500 | 1.0000 | 1 | 1 |
| 37 | 51 | GAATATGTCTCTCAGCCTT | AAGGCTGAGAGACATATTC | 0.1956 | 0.1102 | 0.5345 | 0.7500 | 1.0000 | 1 | 1 |
| 38 | 130 | AGAATATTGATGGTTATTT | AAATAACCATCAATATTCT | 0.4166 | 0.0078 | 0.7241 | 0.7500 | 1.0000 | 1 | 1 |
| 38 | 136 | TTGATGGTTATTTTAAAAT | ATTTTAAAATAACCATCAA | 0.2687 | 0.1590 | 0.6379 | 0.5000 | 1.0000 | 1 | 1 |
| 39 | 53 | ATATGTCTCTCAGCCTTTT | AAAAGGCTGAGAGACATAT | 0.1749 | 0.1244 | 0.5431 | 0.5000 | 1.0000 | 1 | 1 |
| 39 | 138 | GATGGTTATTTTAAAATAT | ATATTTTAAAATAACCATC | 0.0516 | 0.0422 | 0.6379 | 0.7500 | 1.0000 | 1 | 1 |
| 40 | 93 | CAGGGTAATTTCAAAAATC | GATTTTTGAAATTACCCTG | 0.1374 | 0.0088 | 0.6034 | 0.5000 | 1.0000 | 1 | 1 |
| 40 | 135 | ATTGATGGTTATTTTAAAA | TTTTAAAATAACCATCAAT | 0.2881 | 0.1416 | 0.5086 | 0.5000 | 1.0000 | 1 | 1 |
| 40 | 139 | ATGGTTATTTTAAAATATA | TATATTTTAAAATAACCAT | 0.0413 | 0.0293 | 0.6121 | 0.5000 | 1.0000 | 1 | 1 |
| 41 | 131 | GAATATTGATGGTTATTTT | AAAATAACCATCAATATTC | 0.4098 | 0.0071 | 0.6724 | 0.7500 | 1.0000 | 1 | 1 |
| 41 | 140 | TGGTTATTTTAAAATATAT | ATATATTTTAAAATAACCA | 0.0397 | 0.0265 | 0.8017 | 0.7500 | 1.0000 | 1 | 1 |
| 42 | 133 | ATATTGATGGTTATTTTAA | TTAAAATAACCATCAATAT | 0.3597 | 0.0116 | 0.5086 | 0.5000 | 1.0000 | 1 | 1 |
| 42 | 104 | CAAAAATCTTAGGGAATTT | AAATTCCCTAAGATTTTTG | 0.0391 | 0.0257 | 0.6466 | 0.7500 | 1.0000 | 1 | 1 |
| 43 | 141 | GGTTATTTTAAAATATATT | AATATATTTTAAAATAACC | 0.0369 | 0.0241 | 0.8448 | 1.0000 | 1.0000 | 1 | 1 |
| 43 | 88 | GAAAACAGGGTAATTTCAA | TTGAAATTACCCTGTTTTC | 0.2924 | 0.0054 | 0.5259 | 0.7500 | 1.0000 | 1 | 1 |
| 44 | 87 | GGAAAACAGGGTAATTTCA | TGAAATTACCCTGTTTTCC | 0.0524 | 0.0051 | 0.6466 | 0.7500 | 1.0000 | 1 | 1 |
| 44 | 37 | ATAATTGCACTTTTGAATA | TATTCAAAAGTGCAATTAT | 0.3112 | 0.0928 | 0.5 | 0.5000 | 1.0000 | 1 | 1 |
| 45 | 97 | GTAATTTCAAAAATCTTAG | CTAAGATTTTTGAAATTAC | 0.3021 | 0.0864 | 0.5086 | 0.5000 | 1.0000 | 1 | 1 |
| 45 | 89 | AAAACAGGGTAATTTCAAA | TTTGAAATTACCCTGTTTT | 0.2768 | 0.0058 | 0.5 | 0.5000 | 1.0000 | 1 | 1 |
| 46 | 102 | TTCAAAAATCTTAGGGAAT | ATTCCCTAAGATTTTTGAA | 0.0542 | 0.0419 | 0.6638 | 0.5000 | 1.0000 | 1 | 1 |
| 46 | 90 | AAACAGGGTAATTTCAAAA | TTTTGAAATTACCCTGTTT | 0.2647 | 0.0071 | 0.5 | 0.5000 | 1.0000 | 1 | 1 |
| 47 | 52 | AATATGTCTCTCAGCCTTT | AAAGGCTGAGAGACATATT | 0.1816 | 0.1068 | 0.5086 | 0.5000 | 1.0000 | 1 | 1 |
| 47 | 36 | AATAATTGCACTTTTGAAT | ATTCAAAAGTGCAATTATT | 0.2408 | 0.0817 | 0.5 | 0.5000 | 1.0000 | 1 | 1 |
| 48 | 35 | GAATAATTGCACTTTTGAA | TTCAAAAGTGCAATTATTC | 0.1951 | 0.0698 | 0.5 | 0.7500 | 1.0000 | 1 | 1 |
| 48 | 59 | CTCTCAGCCTTTTCTTATG | CATAAGAAAAGGCTGAGAG | 0.2231 | 0.1248 | 0.6552 | 0.5000 | 1.0000 | 1 | 1 |
| 48 | 84 | GAAGGAAAACAGGGTAATT | AATTACCCTGTTTTCCTTC | 0.0235 | 0.0030 | 0.6638 | 0.7500 | 1.0000 | 1 | 1 |
| 49 | 86 | AGGAAAACAGGGTAATTTC | GAAATTACCCTGTTTTCCT | 0.0257 | 0.0037 | 0.6724 | 0.5000 | 1.0000 | 1 | 1 |
| 49 | 85 | AAGGAAAACAGGGTAATTT | AAATTACCCTGTTTTCCTT | 0.0227 | 0.0032 | 0.6379 | 0.5000 | 1.0000 | 1 | 1 |
| 50 | 38 | TAATTGCACTTTTGAATAT | ATATTCAAAAGTGCAATTA | 0.3030 | 0.0977 | 0.4914 | 0.5000 | 1.0000 | 1 | 1 |
| 50 | 132 | AATATTGATGGTTATTTTA | TAAAATAACCATCAATATT | 0.3901 | 0.0076 | 0.4741 | 0.5000 | 1.0000 | 1 | 1 |
| 50 | 83 | TGAAGGAAAACAGGGTAAT | ATTACCCTGTTTTCCTTCA | 0.0207 | 0.0024 | 0.7155 | 0.7500 | 1.0000 | 1 | 1 |
| 51 | 142 | GTTATTTTAAAATATATTC | GAATATATTTTAAAATAAC | 0.0337 | 0.0206 | 0.4741 | 0.5000 | 1.0000 | 1 | 1 |
| 51 | 129 | AAGAATATTGATGGTTATT | AATAACCATCAATATTCTT | 0.5148 | 0.0016 | 0.6293 | 0.5000 | 1.0000 | 1 | 1 |
| 51 | 111 | CTTAGGGAATTTGTGTTTA | TAAACACAAATTCCCTAAG | 0.0141 | 0.0025 | 0.6034 | 0.7500 | 1.0000 | 1 | 1 |
| 52 | 91 | AACAGGGTAATTTCAAAAA | TTTTTGAAATTACCCTGTT | 0.2492 | 0.0088 | 0.6466 | 0.5000 | 1.0000 | 1 | 1 |
| 52 | 145 | ATTTTAAAATATATTCTAA | TTAGAATATATTTTAAAAT | 0.0301 | 0.0167 | 0.4741 | 0.5000 | 1.0000 | 1 | 1 |
| 52 | 109 | ATCTTAGGGAATTTGTGTT | AACACAAATTCCCTAAGAT | 0.0131 | 0.0044 | 0.5345 | 0.5000 | 1.0000 | 1 | 1 |
| 52 | 82 | TTGAAGGAAAACAGGGTAA | TTACCCTGTTTTCCTTCAA | 0.0229 | 0.0015 | 0.6121 | 0.5000 | 1.0000 | 1 | 1 |

**List of siRNAs predicted by RNAxs for the ‘conserved region 9’ of the ‘S’ gene**

| **WORST RANK** | **Position** | **Target sequence** | **siRNA sequence** | **Access 8nts** | **Access 16nts** | **Energy A.** | **Sequence A.** | **Self-Folding** | **Free End** |  |
| --- | --- | --- | --- | --- | --- | --- | --- | --- | --- | --- |
|  |  |  |  |  |  |  |  |  |  |  |
| 8 | 79 | GACTTTTCTATTAAAATAT | ATATTTTAATAGAAAAGTC | 0.2069 | 0.0745 | 0.7759 | 0.7500 | 1.0000 | 1 | 1 |
| 8 | 41 | GGTGCTGCAGCTTATTATG | CATAATAAGCTGCAGCACC | 0.2632 | 0.0320 | 0.6983 | 0.7500 | 0.9837 | 0.875 | 1 |
| 10 | 78 | GGACTTTTCTATTAAAATA | TATTTTAATAGAAAAGTCC | 0.1717 | 0.0747 | 0.8276 | 1.0000 | 1.0000 | 1 | 1 |
| 10 | 40 | TGGTGCTGCAGCTTATTAT | ATAATAAGCTGCAGCACCA | 0.1737 | 0.0243 | 0.8017 | 0.7500 | 0.9837 | 1 | 1 |
| 12 | 80 | ACTTTTCTATTAAAATATA | TATATTTTAATAGAAAAGT | 0.4716 | 0.0747 | 0.6638 | 0.7500 | 1.0000 | 1 | 1 |
| 12 | 39 | CTGGTGCTGCAGCTTATTA | TAATAAGCTGCAGCACCAG | 0.2708 | 0.0222 | 0.75 | 0.7500 | 0.9837 | 1 | 1 |
| 13 | 81 | CTTTTCTATTAAAATATAA | TTATATTTTAATAGAAAAG | 0.4676 | 0.0635 | 0.6034 | 0.7500 | 1.0000 | 1 | 1 |
| 14 | 77 | AGGACTTTTCTATTAAAAT | ATTTTAATAGAAAAGTCCT | 0.1612 | 0.0722 | 0.8362 | 0.7500 | 1.0000 | 1 | 1 |
| 14 | 75 | CTAGGACTTTTCTATTAAA | TTTAATAGAAAAGTCCTAG | 0.0797 | 0.0352 | 0.6724 | 0.7500 | 1.0000 | 1 | 1 |
| 17 | 42 | GTGCTGCAGCTTATTATGT | ACATAATAAGCTGCAGCAC | 0.0785 | 0.0081 | 0.5862 | 0.5000 | 0.9837 | 0.75 | 1 |
| 18 | 74 | CCTAGGACTTTTCTATTAA | TTAATAGAAAAGTCCTAGG | 0.0795 | 0.0075 | 0.8103 | 1.0000 | 1.0000 | 1 | 1 |
| 18 | 85 | TCTATTAAAATATAATGAA | TTCATTATATTTTAATAGA | 0.3753 | 0.0292 | 0.5517 | 0.7500 | 1.0000 | 1 | 1 |
| 19 | 86 | CTATTAAAATATAATGAAA | TTTCATTATATTTTAATAG | 0.0623 | 0.0230 | 0.6724 | 0.7500 | 1.0000 | 1 | 1 |
| 20 | 107 | GGAACCATTACAGATGCTG | CAGCATCTGTAATGGTTCC | 0.0363 | 0.0085 | 0.5862 | 0.7500 | 1.0000 | 1 | 1 |
| 20 | 100 | TGAAAATGGAACCATTACA | TGTAATGGTTCCATTTTCA | 0.1404 | 0.0031 | 0.5172 | 0.5000 | 1.0000 | 1 | 1 |
| 21 | 123 | CTGTAGACTGTGCACTTGA | TCAAGTGCACAGTCTACAG | 0.3495 | 0.0032 | 0.5603 | 0.5000 | 1.0000 | 1 | 1 |
| 21 | 87 | TATTAAAATATAATGAAAA | TTTTCATTATATTTTAATA | 0.0204 | 0.0192 | 0.5431 | 0.5000 | 1.0000 | 1 | 1 |
| 21 | 122 | GCTGTAGACTGTGCACTTG | CAAGTGCACAGTCTACAGC | 0.3106 | 0.0019 | 0.7069 | 0.7500 | 1.0000 | 1 | 1 |
| 22 | 73 | ACCTAGGACTTTTCTATTA | TAATAGAAAAGTCCTAGGT | 0.0790 | 0.0014 | 0.819 | 0.7500 | 1.0000 | 1 | 1 |
| 22 | 88 | ATTAAAATATAATGAAAAT | ATTTTCATTATATTTTAAT | 0.0145 | 0.0128 | 0.5 | 0.5000 | 1.0000 | 1 | 1 |
| 22 | 82 | TTTTCTATTAAAATATAAT | ATTATATTTTAATAGAAAA | 0.4500 | 0.0510 | 0.4914 | 0.5000 | 1.0000 | 1 | 1 |
| 23 | 76 | TAGGACTTTTCTATTAAAA | TTTTAATAGAAAAGTCCTA | 0.1336 | 0.0354 | 0.6724 | 0.5000 | 1.0000 | 1 | 1 |
| 23 | 43 | TGCTGCAGCTTATTATGTG | CACATAATAAGCTGCAGCA | 0.0127 | 0.0011 | 0.5603 | 0.5000 | 0.9837 | 0.75 | 1 |
| 23 | 106 | TGGAACCATTACAGATGCT | AGCATCTGTAATGGTTCCA | 0.0782 | 0.0123 | 0.4914 | 0.5000 | 1.0000 | 1 | 1 |

**List of siRNAs predicted by RNAxs for the ‘conserved region 10’ of the ‘S’ gene**

| **Position** | **Target sequence** | **siRNA sequence** | **Access 8nts** | **Access 16nts** | **Energy A.** | **Sequence A.** | **Self-Folding** | **Free End** |  |
| --- | --- | --- | --- | --- | --- | --- | --- | --- | --- |
|  |  |  |  |  |  |  |  |  |  |
| 61 | GAAAAAGGAATCTATCAAA | TTTGATAGATTCCTTTTTC | 0.1650 | 0.0294 | 0.6724 | 0.7500 | 1.0000 | 1 | 1 |
| 91 | AGAGTCCAACCAACAGAAT | ATTCTGTTGGTTGGACTCT | 0.2275 | 0.0036 | 0.7155 | 0.7500 | 0.9946 | 0.875 | 1 |
| 119 | GATTTCCTAATATTACAAA | TTTGTAATATTAGGAAATC | 0.9662 | 0.6641 | 0.681 | 0.7500 | 1.0000 | 1 | 1 |
| 95 | TCCAACCAACAGAATCTAT | ATAGATTCTGTTGGTTGGA | 0.1415 | 0.1154 | 0.8276 | 0.7500 | 1.0000 | 1 | 1 |
| 71 | TCTATCAAACTTCTAACTT | AAGTTAGAAGTTTGATAGA | 0.4691 | 0.0106 | 0.5862 | 0.7500 | 1.0000 | 1 | 1 |
| 60 | AGAAAAAGGAATCTATCAA | TTGATAGATTCCTTTTTCT | 0.1641 | 0.0294 | 0.5776 | 0.7500 | 1.0000 | 1 | 1 |
| 118 | AGATTTCCTAATATTACAA | TTGTAATATTAGGAAATCT | 0.9652 | 0.7138 | 0.5776 | 0.7500 | 1.0000 | 1 | 1 |
| 94 | GTCCAACCAACAGAATCTA | TAGATTCTGTTGGTTGGAC | 0.1474 | 0.0029 | 0.6466 | 0.7500 | 1.0000 | 1 | 1 |
| 53 | TCACTGTAGAAAAAGGAAT | ATTCCTTTTTCTACAGTGA | 0.0382 | 0.0040 | 0.7155 | 0.7500 | 1.0000 | 1 | 1 |
| 54 | CACTGTAGAAAAAGGAATC | GATTCCTTTTTCTACAGTG | 0.0318 | 0.0045 | 0.6121 | 0.5000 | 1.0000 | 1 | 1 |
| 122 | TTCCTAATATTACAAACTT | AAGTTTGTAATATTAGGAA | 0.9686 | 0.9168 | 0.5345 | 0.5000 | 1.0000 | 1 | 1 |
| 90 | TAGAGTCCAACCAACAGAA | TTCTGTTGGTTGGACTCTA | 0.3548 | 0.0028 | 0.5 | 0.5000 | 0.9946 | 1 | 1 |
| 36 | GTGTACGTTGAAATCCTTC | GAAGGATTTCAACGTACAC | 0.0257 | 0.0023 | 0.6207 | 0.5000 | 1.0000 | 1 | 1 |
| 45 | GAAATCCTTCACTGTAGAA | TTCTACAGTGAAGGATTTC | 0.1082 | 0.0022 | 0.5 | 0.7500 | 0.9837 | 0.625 | 1 |
| 92 | GAGTCCAACCAACAGAATC | GATTCTGTTGGTTGGACTC | 0.2005 | 0.0022 | 0.6293 | 0.5000 | 0.9946 | 0.75 | 1 |
| 52 | TTCACTGTAGAAAAAGGAA | TTCCTTTTTCTACAGTGAA | 0.2693 | 0.0033 | 0.5 | 0.5000 | 1.0000 | 1 | 1 |
| 22 | CTCTCAGAAACAAAGTGTA | TACACTTTGTTTCTGAGAG | 0.0219 | 0.0100 | 0.6293 | 0.7500 | 1.0000 | 1 | 1 |
| 35 | AGTGTACGTTGAAATCCTT | AAGGATTTCAACGTACACT | 0.0419 | 0.0019 | 0.569 | 0.7500 | 1.0000 | 1 | 1 |
| 23 | TCTCAGAAACAAAGTGTAC | GTACACTTTGTTTCTGAGA | 0.0187 | 0.0079 | 0.5431 | 0.5000 | 1.0000 | 1 | 1 |
| 55 | ACTGTAGAAAAAGGAATCT | AGATTCCTTTTTCTACAGT | 0.0315 | 0.0052 | 0.4828 | 0.5000 | 0.9783 | 0.625 | 1 |
| 30 | AACAAAGTGTACGTTGAAA | TTTCAACGTACACTTTGTT | 0.0251 | 0.0031 | 0.6466 | 0.5000 | 1.0000 | 1 | 1 |
| 93 | AGTCCAACCAACAGAATCT | AGATTCTGTTGGTTGGACT | 0.1743 | 0.0031 | 0.4828 | 0.5000 | 0.9946 | 0.625 | 1 |
| 66 | AGGAATCTATCAAACTTCT | AGAAGTTTGATAGATTCCT | 0.4733 | 0.0017 | 0.6207 | 0.5000 | 1.0000 | 1 | 1 |
| 44 | TGAAATCCTTCACTGTAGA | TCTACAGTGAAGGATTTCA | 0.1082 | 0.0019 | 0.5 | 0.5000 | 1.0000 | 1 | 1 |
| 31 | ACAAAGTGTACGTTGAAAT | ATTTCAACGTACACTTTGT | 0.0171 | 0.0025 | 0.6983 | 0.7500 | 1.0000 | 1 | 1 |
| 32 | CAAAGTGTACGTTGAAATC | GATTTCAACGTACACTTTG | 0.0181 | 0.0024 | 0.4655 | 0.5000 | 1.0000 | 1 | 1 |
| 67 | GGAATCTATCAAACTTCTA | TAGAAGTTTGATAGATTCC | 0.4744 | 0.0012 | 0.6983 | 1.0000 | 1.0000 | 1 | 1 |

**List of siRNAs predicted by RNAxs for the ‘conserved region 12’ of the ‘S’ gene**

| **WORST RANK** | **Position** | **Target sequence** | **siRNA sequence** | **Access 8nts** | **Access 16nts** | **Energy A.** | **Sequence A.** | **Self-Folding** | **Free End** |  |
| --- | --- | --- | --- | --- | --- | --- | --- | --- | --- | --- |
|  |  |  |  |  |  |  |  |  |  |  |
| 3 | 93 | CCGCATCATTTTCCACTTT | AAAGTGGAAAATGATGCGG | 0.6162 | 0.3530 | 0.9138 | 1.0000 | 1.0000 | 1 | 1 |
| 4 | 94 | CGCATCATTTTCCACTTTT | AAAAGTGGAAAATGATGCG | 0.5941 | 0.3163 | 0.9224 | 1.0000 | 1.0000 | 1 | 1 |
| 5 | 92 | TCCGCATCATTTTCCACTT | AAGTGGAAAATGATGCGGA | 0.4215 | 0.3902 | 0.7328 | 0.7500 | 1.0000 | 1 | 1 |
| 6 | 82 | CCTATATAATTCCGCATCA | *TGATGCGGAATTATATAGG* | 0.7977 | 0.7914 | 0.6207 | 0.7500 | 1.0000 | 1 | 1 |
| 7 | 95 | GCATCATTTTCCACTTTTA | *TAAAAGTGGAAAATGATGC* | 0.2988 | 0.2700 | 0.819 | 1.0000 | 1.0000 | 1 | 1 |
| 8 | 96 | CATCATTTTCCACTTTTAA | *TTAAAAGTGGAAAATGATG* | 0.2323 | 0.2214 | 0.6207 | 0.7500 | 1.0000 | 1 | 1 |
| 8 | 80 | GTCCTATATAATTCCGCAT | ATGCGGAATTATATAGGAC | 0.8407 | 0.0539 | 0.6638 | 0.7500 | 1.0000 | 1 | 1 |
| 8 | 81 | TCCTATATAATTCCGCATC | GATGCGGAATTATATAGGA | 0.8091 | 0.1550 | 0.6897 | 0.5000 | 1.0000 | 1 | 1 |
| 9 | 98 | TCATTTTCCACTTTTAAGT | ACTTAAAAGTGGAAAATGA | 0.0829 | 0.0773 | 0.5172 | 0.5000 | 1.0000 | 1 | 1 |
| 10 | 24 | CATCTGTTTATGCTTGGAA | TTCCAAGCATAAACAGATG | 0.3858 | 0.0503 | 0.4828 | 0.7500 | 1.0000 | 1 | 1 |
| 10 | 26 | TCTGTTTATGCTTGGAACA | TGTTCCAAGCATAAACAGA | 0.0636 | 0.0075 | 0.5172 | 0.5000 | 1.0000 | 1 | 1 |

**List of siRNAs predicted by RNAxs for the ‘conserved region 13’ of the ‘S’ gene**

| **WORST RANK** | **Position** | **Target sequence** | **siRNA sequence** | **Access 8nts** | **Access 16nts** | **Energy A.** | **Sequence A.** | **Self-Folding** | **Free End** |  |
| --- | --- | --- | --- | --- | --- | --- | --- | --- | --- | --- |
|  |  |  |  |  |  |  |  |  |  |  |
| 8 | 80 | GAGGTGATGAAGTCAGACA | TGTCTGACTTCATCACCTC | 0.5639 | 0.0295 | 0.6034 | 0.5000 | 1.0000 | 1 | 1 |
| 9 | 81 | AGGTGATGAAGTCAGACAA | TTGTCTGACTTCATCACCT | 0.5741 | 0.0245 | 0.6983 | 0.7500 | 1.0000 | 1 | 1 |
| 10 | 82 | GGTGATGAAGTCAGACAAA | TTTGTCTGACTTCATCACC | 0.0851 | 0.0211 | 0.8534 | 1.0000 | 1.0000 | 1 | 1 |
| 10 | 73 | GTAATTAGAGGTGATGAAG | CTTCATCACCTCTAATTAC | 0.0372 | 0.0304 | 0.5431 | 0.5000 | 1.0000 | 1 | 1 |
| 11 | 72 | TGTAATTAGAGGTGATGAA | TTCATCACCTCTAATTACA | 0.0374 | 0.0315 | 0.5345 | 0.7500 | 1.0000 | 1 | 1 |
| 11 | 19 | TCTCCTACTAAATTAAATG | CATTTAATTTAGTAGGAGA | 0.0226 | 0.0528 | 0.569 | 0.5000 | 1.0000 | 1 | 1 |
| 11 | 83 | GTGATGAAGTCAGACAAAT | ATTTGTCTGACTTCATCAC | 0.0788 | 0.0205 | 0.7845 | 0.7500 | 1.0000 | 1 | 1 |
| 12 | 62 | CAGATTCATTTGTAATTAG | CTAATTACAAATGAATCTG | 0.4400 | 0.0127 | 0.6121 | 0.5000 | 1.0000 | 1 | 1 |
| 12 | 20 | CTCCTACTAAATTAAATGA | TCATTTAATTTAGTAGGAG | 0.0209 | 0.0469 | 0.5862 | 0.5000 | 1.0000 | 1 | 1 |
| 13 | 63 | AGATTCATTTGTAATTAGA | TCTAATTACAAATGAATCT | 0.4022 | 0.0458 | 0.5 | 0.5000 | 1.0000 | 1 | 1 |
| 13 | 21 | TCCTACTAAATTAAATGAT | ATCATTTAATTTAGTAGGA | 0.0195 | 0.0430 | 0.6897 | 0.7500 | 1.0000 | 1 | 1 |
| 14 | 84 | TGATGAAGTCAGACAAATC | GATTTGTCTGACTTCATCA | 0.0786 | 0.0421 | 0.5431 | 0.5000 | 1.0000 | 1 | 1 |
| 14 | 37 | GATCTCTGCTTTACTAATG | CATTAGTAAAGCAGAGATC | 0.1634 | 0.0015 | 0.5259 | 0.5000 | 1.0000 | 1 | 1 |
| 14 | 95 | GACAAATCGCTCCAGGGCA | TGCCCTGGAGCGATTTGTC | 0.0918 | 0.0023 | 0.4655 | 0.5000 | 0.9620 | 0.625 | 1 |
| 14 | 22 | CCTACTAAATTAAATGATC | GATCATTTAATTTAGTAGG | 0.0183 | 0.0374 | 0.6638 | 0.7500 | 1.0000 | 1 | 1 |

**List of siRNAs predicted by RNAxs for the ‘conserved region 14’ of the ‘S’ gene**

| **WORST RANK** | **Position** | **Target sequence** | **siRNA sequence** | **Access 8nts** | **Access 16nts** | **Energy A.** | **Sequence A.** | **Self-Folding** | **Free End** |  |
| --- | --- | --- | --- | --- | --- | --- | --- | --- | --- | --- |
|  |  |  |  |  |  |  |  |  |  |  |
| 4 | 23 | CTGATTATAATTATAAATT | AATTTATAATTATAATCAG | 0.4531 | 0.3302 | 0.7759 | 0.7500 | 1.0000 | 1 | 1 |
| 5 | 22 | GCTGATTATAATTATAAAT | ATTTATAATTATAATCAGC | 0.4525 | 0.2312 | 0.8448 | 1.0000 | 1.0000 | 1 | 1 |
| 6 | 24 | TGATTATAATTATAAATTA | TAATTTATAATTATAATCA | 0.4597 | 0.3569 | 0.6897 | 0.7500 | 1.0000 | 1 | 1 |
| 8 | 21 | TGCTGATTATAATTATAAA | TTTATAATTATAATCAGCA | 0.4494 | 0.1809 | 0.8534 | 0.7500 | 1.0000 | 1 | 1 |
| 11 | 35 | ATAAATTACCAGATGATTT | AAATCATCTGGTAATTTAT | 0.2683 | 0.2002 | 0.5431 | 0.5000 | 1.0000 | 1 | 1 |
| 11 | 34 | TATAAATTACCAGATGATT | AATCATCTGGTAATTTATA | 0.2905 | 0.1999 | 0.5345 | 0.5000 | 1.0000 | 1 | 1 |
| 13 | 20 | TTGCTGATTATAATTATAA | TTATAATTATAATCAGCAA | 0.4864 | 0.1413 | 0.6121 | 0.5000 | 1.0000 | 1 | 1 |
| 13 | 79 | TCTAACAATCTTGATTCTA | TAGAATCAAGATTGTTAGA | 0.0505 | 0.0242 | 0.5517 | 0.7500 | 1.0000 | 1 | 1 |
| 14 | 25 | GATTATAATTATAAATTAC | GTAATTTATAATTATAATC | 0.4782 | 0.3688 | 0.5 | 0.5000 | 1.0000 | 1 | 1 |
| 15 | 36 | TAAATTACCAGATGATTTT | AAAATCATCTGGTAATTTA | 0.2756 | 0.2217 | 0.5345 | 0.5000 | 1.0000 | 1 | 1 |
| 15 | 80 | CTAACAATCTTGATTCTAA | TTAGAATCAAGATTGTTAG | 0.0271 | 0.0245 | 0.6379 | 0.7500 | 1.0000 | 1 | 1 |
| 16 | 101 | TTGGTGGTAATTATAATTA | TAATTATAATTACCACCAA | 0.1794 | 0.0285 | 0.6121 | 0.5000 | 1.0000 | 1 | 1 |
| 16 | 74 | GGAATTCTAACAATCTTGA | TCAAGATTGTTAGAATTCC | 0.0260 | 0.0042 | 0.6466 | 0.7500 | 1.0000 | 1 | 1 |
| 17 | 75 | GAATTCTAACAATCTTGAT | ATCAAGATTGTTAGAATTC | 0.0256 | 0.0055 | 0.4914 | 0.7500 | 1.0000 | 1 | 1 |
| 17 | 72 | TTGGAATTCTAACAATCTT | AAGATTGTTAGAATTCCAA | 0.0975 | 0.0038 | 0.5086 | 0.5000 | 1.0000 | 1 | 1 |
| 17 | 19 | ATTGCTGATTATAATTATA | TATAATTATAATCAGCAAT | 0.4943 | 0.1191 | 0.4655 | 0.5000 | 1.0000 | 1 | 1 |
| 18 | 76 | AATTCTAACAATCTTGATT | AATCAAGATTGTTAGAATT | 0.0254 | 0.0057 | 0.5 | 0.5000 | 1.0000 | 1 | 1 |
| 18 | 73 | TGGAATTCTAACAATCTTG | CAAGATTGTTAGAATTCCA | 0.0296 | 0.0038 | 0.6983 | 0.5000 | 1.0000 | 1 | 1 |
| 18 | 37 | AAATTACCAGATGATTTTA | TAAAATCATCTGGTAATTT | 0.2818 | 0.2454 | 0.4655 | 0.5000 | 1.0000 | 1 | 1 |

**List of siRNAs predicted by RNAxs for the ‘conserved region 15’ of the ‘S’ gene**

| **WORST RANK** | **Position** | **Target sequence** | **siRNA sequence** | **Access 8nts** | **Access 16nts** | **Energy A.** | **Sequence A.** | **Self-Folding** | **Free End** |  |
| --- | --- | --- | --- | --- | --- | --- | --- | --- | --- | --- |
|  |  |  |  |  |  |  |  |  |  |  |
| 0 | 74 | GAAATCTATCAGGCCGGTA | TACCGGCCTGATAGATTTC | 0.1497 | 0.0047 | 0.4914 | 0.7500 | 1.0000 | 1 | 1 |

**List of siRNAs predicted by RNAxs for the ‘conserved region 19’ of the ‘S’ gene**

| **WORST RANK** | **Position** | **Target sequence** | **siRNA sequence** | **Access 8nts** | **Access 16nts** | **Energy A.** | **Sequence A.** | **Self-Folding** | **Free End** |  |
| --- | --- | --- | --- | --- | --- | --- | --- | --- | --- | --- |
|  |  |  |  |  |  |  |  |  |  |  |
| 6 | 130 | GTGTTCTTACTGAGTCTAA | TTAGACTCAGTAAGAACAC | 0.2679 | 0.0492 | 0.7586 | 0.7500 | 1.0000 | 1 | 1 |
| 8 | 95 | TGTCAATTTCAACTTCAAT | ATTGAAGTTGAAATTGACA | 0.1318 | 0.0924 | 0.6983 | 0.7500 | 1.0000 | 1 | 1 |
| 11 | 94 | GTGTCAATTTCAACTTCAA | TTGAAGTTGAAATTGACAC | 0.1552 | 0.0858 | 0.6466 | 0.7500 | 1.0000 | 1 | 1 |
| 13 | 129 | GGTGTTCTTACTGAGTCTA | TAGACTCAGTAAGAACACC | 0.0886 | 0.0448 | 0.681 | 1.0000 | 1.0000 | 1 | 1 |
| 16 | 96 | GTCAATTTCAACTTCAATG | CATTGAAGTTGAAATTGAC | 0.1083 | 0.0783 | 0.6638 | 0.5000 | 1.0000 | 1 | 1 |
| 20 | 88 | ACAAATGTGTCAATTTCAA | TTGAAATTGACACATTTGT | 0.1474 | 0.0192 | 0.5603 | 0.7500 | 1.0000 | 1 | 1 |
| 21 | 102 | TTCAACTTCAATGGTTTAA | TTAAACCATTGAAGTTGAA | 0.0392 | 0.0350 | 0.6379 | 0.5000 | 1.0000 | 1 | 1 |
| 22 | 128 | AGGTGTTCTTACTGAGTCT | AGACTCAGTAAGAACACCT | 0.0509 | 0.0398 | 0.6207 | 0.5000 | 1.0000 | 1 | 1 |
| 22 | 80 | GGTTAAAAACAAATGTGTC | GACACATTTGTTTTTAACC | 0.0385 | 0.0163 | 0.5345 | 0.7500 | 1.0000 | 1 | 1 |
| 23 | 59 | ACCTAAAAAGTCTACTAAT | ATTAGTAGACTTTTTAGGT | 0.0367 | 0.0235 | 0.8448 | 0.7500 | 1.0000 | 1 | 1 |
| 24 | 85 | AAAACAAATGTGTCAATTT | AAATTGACACATTTGTTTT | 0.1545 | 0.0285 | 0.5 | 0.5000 | 0.9891 | 0.75 | 1 |
| 24 | 74 | TAATTTGGTTAAAAACAAA | TTTGTTTTTAACCAAATTA | 0.1039 | 0.0159 | 0.5345 | 0.5000 | 1.0000 | 1 | 1 |
| 25 | 73 | CTAATTTGGTTAAAAACAA | TTGTTTTTAACCAAATTAG | 0.1029 | 0.0150 | 0.5259 | 0.7500 | 1.0000 | 1 | 1 |
| 25 | 83 | TAAAAACAAATGTGTCAAT | ATTGACACATTTGTTTTTA | 0.1355 | 0.0304 | 0.5259 | 0.5000 | 0.9891 | 0.875 | 1 |
| 26 | 126 | ACAGGTGTTCTTACTGAGT | ACTCAGTAAGAACACCTGT | 0.0780 | 0.0666 | 0.5 | 0.5000 | 1.0000 | 1 | 1 |
| 26 | 72 | ACTAATTTGGTTAAAAACA | TGTTTTTAACCAAATTAGT | 0.0614 | 0.0148 | 0.5 | 0.5000 | 1.0000 | 1 | 1 |
| 27 | 79 | TGGTTAAAAACAAATGTGT | ACACATTTGTTTTTAACCA | 0.0340 | 0.0097 | 0.6379 | 0.5000 | 1.0000 | 1 | 1 |
| 28 | 100 | ATTTCAACTTCAATGGTTT | AAACCATTGAAGTTGAAAT | 0.0518 | 0.0397 | 0.5086 | 0.5000 | 1.0000 | 1 | 1 |
| 28 | 70 | CTACTAATTTGGTTAAAAA | TTTTTAACCAAATTAGTAG | 0.7967 | 0.0078 | 0.6724 | 0.7500 | 1.0000 | 1 | 1 |
| 29 | 89 | CAAATGTGTCAATTTCAAC | GTTGAAATTGACACATTTG | 0.1305 | 0.0210 | 0.5 | 0.5000 | 0.9946 | 0.625 | 1 |
| 29 | 69 | TCTACTAATTTGGTTAAAA | TTTTAACCAAATTAGTAGA | 0.0232 | 0.0073 | 0.7241 | 0.7500 | 1.0000 | 1 | 1 |
| 29 | 125 | CACAGGTGTTCTTACTGAG | CTCAGTAAGAACACCTGTG | 0.0857 | 0.0510 | 0.4828 | 0.5000 | 1.0000 | 1 | 1 |
| 29 | 60 | CCTAAAAAGTCTACTAATT | AATTAGTAGACTTTTTAGG | 0.0225 | 0.0176 | 0.8362 | 1.0000 | 1.0000 | 1 | 1 |
| 30 | 68 | GTCTACTAATTTGGTTAAA | TTTAACCAAATTAGTAGAC | 0.0227 | 0.0065 | 0.819 | 0.7500 | 1.0000 | 1 | 1 |
| 30 | 103 | TCAACTTCAATGGTTTAAC | GTTAAACCATTGAAGTTGA | 0.0343 | 0.0311 | 0.5776 | 0.5000 | 1.0000 | 1 | 1 |
| 30 | 20 | TTCTTTTGAACTTCTACAT | ATGTAGAAGTTCAAAAGAA | 0.0208 | 0.0213 | 0.5172 | 0.5000 | 1.0000 | 1 | 1 |
| 31 | 93 | TGTGTCAATTTCAACTTCA | TGAAGTTGAAATTGACACA | 0.1752 | 0.0804 | 0.4828 | 0.5000 | 1.0000 | 1 | 1 |
| 31 | 67 | AGTCTACTAATTTGGTTAA | TTAACCAAATTAGTAGACT | 0.0250 | 0.0062 | 0.6724 | 0.7500 | 1.0000 | 1 | 1 |
| 31 | 127 | CAGGTGTTCTTACTGAGTC | GACTCAGTAAGAACACCTG | 0.0620 | 0.0482 | 0.4655 | 0.5000 | 1.0000 | 1 | 1 |
| 32 | 84 | AAAAACAAATGTGTCAATT | AATTGACACATTTGTTTTT | 0.1368 | 0.0293 | 0.4914 | 0.5000 | 0.9891 | 0.875 | 1 |
| 32 | 66 | AAGTCTACTAATTTGGTTA | TAACCAAATTAGTAGACTT | 0.0203 | 0.0062 | 0.6034 | 0.5000 | 1.0000 | 1 | 1 |
| 32 | 101 | TTTCAACTTCAATGGTTTA | TAAACCATTGAAGTTGAAA | 0.0457 | 0.0402 | 0.4655 | 0.5000 | 1.0000 | 1 | 1 |
| 32 | 61 | CTAAAAAGTCTACTAATTT | AAATTAGTAGACTTTTTAG | 0.0200 | 0.0162 | 0.6724 | 0.7500 | 1.0000 | 1 | 1 |

**List of siRNAs predicted by RNAxs for the ‘conserved region 22’ of the ‘S’ gene**

| **WORST RANK** | **Position** | **Target sequence** | **siRNA sequence** | **Access 8nts** | **Access 16nts** | **Energy A.** | **Sequence A.** | **Self-Folding** | **Free End** |  |
| --- | --- | --- | --- | --- | --- | --- | --- | --- | --- | --- |
|  |  |  |  |  |  |  |  |  |  |  |
| 2 | 68 | CAGGAACAAATACTTCTAA | TTAGAAGTATTTGTTCCTG | 0.1654 | 0.0042 | 0.75 | 0.7500 | 1.0000 | 1 | 1 |
| 3 | 66 | ACCAGGAACAAATACTTCT | AGAAGTATTTGTTCCTGGT | 0.1646 | 0.0043 | 0.6293 | 0.5000 | 1.0000 | 1 | 1 |
| 4 | 67 | CCAGGAACAAATACTTCTA | TAGAAGTATTTGTTCCTGG | 0.1644 | 0.0040 | 0.6724 | 1.0000 | 1.0000 | 1 | 1 |
| 5 | 65 | CACCAGGAACAAATACTTC | GAAGTATTTGTTCCTGGTG | 0.1652 | 0.0042 | 0.6207 | 0.5000 | 1.0000 | 1 | 1 |
| 5 | 64 | ACACCAGGAACAAATACTT | AAGTATTTGTTCCTGGTGT | 0.1670 | 0.0014 | 0.569 | 0.7500 | 1.0000 | 1 | 1 |
| 5 | 69 | AGGAACAAATACTTCTAAC | GTTAGAAGTATTTGTTCCT | 0.1599 | 0.0042 | 0.6983 | 0.5000 | 1.0000 | 1 | 1 |

**List of siRNAs predicted by RNAxs for the ‘conserved region 23’ of the ‘S’ gene**

| **WORST RANK** | **Position** | **Target sequence** | **siRNA sequence** | **Access 8nts** | **Access 16nts** | **Energy A.** | **Sequence A.** | **Self-Folding** | **Free End** |  |
| --- | --- | --- | --- | --- | --- | --- | --- | --- | --- | --- |
|  |  |  |  |  |  |  |  |  |  |  |
| 8 | 92 | GGTTCTAATGTTTTTCAAA | TTTGAAAAACATTAGAACC | 0.1546 | 0.0558 | 0.8534 | 1.0000 | 1.0000 | 1 | 1 |
| 9 | 53 | GCAGATCAACTTACTCCTA | TAGGAGTAAGTTGATCTGC | 0.5040 | 0.2201 | 0.681 | 1.0000 | 1.0000 | 1 | 1 |
| 16 | 87 | CTACAGGTTCTAATGTTTT | AAAACATTAGAACCTGTAG | 0.1244 | 0.0488 | 0.6724 | 0.7500 | 1.0000 | 1 | 1 |
| 18 | 91 | AGGTTCTAATGTTTTTCAA | TTGAAAAACATTAGAACCT | 0.1222 | 0.0532 | 0.6983 | 0.7500 | 1.0000 | 1 | 1 |
| 18 | 43 | TGCTATTCATGCAGATCAA | TTGATCTGCATGAATAGCA | 0.1280 | 0.0395 | 0.7069 | 0.7500 | 0.9674 | 0.625 | 1 |
| 19 | 86 | TCTACAGGTTCTAATGTTT | AAACATTAGAACCTGTAGA | 0.1303 | 0.0489 | 0.7241 | 0.7500 | 1.0000 | 1 | 1 |
| 24 | 56 | GATCAACTTACTCCTACTT | AAGTAGGAGTAAGTTGATC | 0.5215 | 0.3694 | 0.5431 | 0.7500 | 1.0000 | 1 | 1 |
| 25 | 113 | CGTGCAGGCTGTTTAATAG | CTATTAAACAGCCTGCACG | 0.1237 | 0.0135 | 0.6034 | 0.7500 | 1.0000 | 1 | 1 |
| 27 | 89 | ACAGGTTCTAATGTTTTTC | GAAAAACATTAGAACCTGT | 0.1139 | 0.0524 | 0.5345 | 0.5000 | 1.0000 | 1 | 1 |
| 27 | 85 | TTCTACAGGTTCTAATGTT | AACATTAGAACCTGTAGAA | 0.1344 | 0.0431 | 0.5259 | 0.5000 | 1.0000 | 1 | 1 |
| 29 | 55 | AGATCAACTTACTCCTACT | AGTAGGAGTAAGTTGATCT | 0.4980 | 0.3617 | 0.5172 | 0.5000 | 1.0000 | 1 | 1 |
| 30 | 77 | CGTGTTTATTCTACAGGTT | AACCTGTAGAATAAACACG | 0.0883 | 0.0042 | 0.6293 | 1.0000 | 1.0000 | 1 | 1 |
| 31 | 40 | TGTTGCTATTCATGCAGAT | ATCTGCATGAATAGCAACA | 0.0963 | 0.0039 | 0.5259 | 0.7500 | 0.9185 | 0.75 | 1 |
| 32 | 112 | ACGTGCAGGCTGTTTAATA | TATTAAACAGCCTGCACGT | 0.1151 | 0.0025 | 0.7328 | 0.7500 | 1.0000 | 1 | 1 |
| 32 | 90 | CAGGTTCTAATGTTTTTCA | TGAAAAACATTAGAACCTG | 0.1096 | 0.0504 | 0.5603 | 0.5000 | 1.0000 | 1 | 1 |
| 32 | 93 | GTTCTAATGTTTTTCAAAC | GTTTGAAAAACATTAGAAC | 0.1519 | 0.0542 | 0.5 | 0.5000 | 1.0000 | 1 | 1 |
| 33 | 136 | TGAACATGTCAACAACTCA | TGAGTTGTTGACATGTTCA | 0.0881 | 0.0145 | 0.5 | 0.5000 | 1.0000 | 1 | 1 |
| 33 | 170 | CCCATTGGTGCAGGTATAT | ATATACCTGCACCAATGGG | 0.0826 | 0.0044 | 0.9483 | 1.0000 | 0.9620 | 0.625 | 1 |
| 34 | 88 | TACAGGTTCTAATGTTTTT | AAAAACATTAGAACCTGTA | 0.1187 | 0.0508 | 0.681 | 0.5000 | 1.0000 | 1 | 1 |
| 36 | 32 | GAAGTCCCTGTTGCTATTC | GAATAGCAACAGGGACTTC | 0.1197 | 0.0019 | 0.5 | 0.5000 | 1.0000 | 1 | 1 |
| 36 | 79 | TGTTTATTCTACAGGTTCT | AGAACCTGTAGAATAAACA | 0.1171 | 0.0270 | 0.4828 | 0.5000 | 1.0000 | 1 | 1 |
| 37 | 137 | GAACATGTCAACAACTCAT | ATGAGTTGTTGACATGTTC | 0.0736 | 0.0096 | 0.5172 | 0.7500 | 1.0000 | 1 | 1 |
| 37 | 49 | TCATGCAGATCAACTTACT | AGTAAGTTGATCTGCATGA | 0.5005 | 0.0856 | 0.5172 | 0.5000 | 1.0000 | 1 | 1 |
| 37 | 135 | CTGAACATGTCAACAACTC | GAGTTGTTGACATGTTCAG | 0.1015 | 0.0193 | 0.4741 | 0.5000 | 1.0000 | 1 | 1 |
| 37 | 38 | CCTGTTGCTATTCATGCAG | CTGCATGAATAGCAACAGG | 0.0779 | 0.0018 | 0.5603 | 0.7500 | 0.9185 | 0.625 | 1 |
| 38 | 39 | CTGTTGCTATTCATGCAGA | TCTGCATGAATAGCAACAG | 0.0864 | 0.0023 | 0.5603 | 0.5000 | 0.9185 | 0.75 | 1 |
| 38 | 138 | AACATGTCAACAACTCATA | TATGAGTTGTTGACATGTT | 0.0616 | 0.0154 | 0.6034 | 0.5000 | 1.0000 | 1 | 1 |
| 39 | 80 | GTTTATTCTACAGGTTCTA | TAGAACCTGTAGAATAAAC | 0.1437 | 0.0356 | 0.4741 | 0.7500 | 1.0000 | 1 | 1 |
| 39 | 82 | TTATTCTACAGGTTCTAAT | ATTAGAACCTGTAGAATAA | 0.1416 | 0.0501 | 0.5259 | 0.5000 | 1.0000 | 1 | 1 |
| 39 | 139 | ACATGTCAACAACTCATAT | ATATGAGTTGTTGACATGT | 0.0504 | 0.0109 | 0.6638 | 0.7500 | 1.0000 | 1 | 1 |
| 39 | 37 | CCCTGTTGCTATTCATGCA | TGCATGAATAGCAACAGGG | 0.0807 | 0.0015 | 0.6379 | 0.7500 | 0.9293 | 0.625 | 1 |
| 40 | 34 | AGTCCCTGTTGCTATTCAT | ATGAATAGCAACAGGGACT | 0.1269 | 0.0014 | 0.5517 | 0.7500 | 1.0000 | 1 | 1 |
| 40 | 47 | ATTCATGCAGATCAACTTA | TAAGTTGATCTGCATGAAT | 0.4971 | 0.0681 | 0.4741 | 0.5000 | 1.0000 | 1 | 1 |
| 40 | 140 | CATGTCAACAACTCATATG | CATATGAGTTGTTGACATG | 0.0354 | 0.0018 | 0.5 | 0.5000 | 1.0000 | 1 | 1 |
| 40 | 81 | TTTATTCTACAGGTTCTAA | TTAGAACCTGTAGAATAAA | 0.8016 | 0.0446 | 0.4655 | 0.5000 | 1.0000 | 1 | 1 |
| 41 | 171 | CCATTGGTGCAGGTATATG | CATATACCTGCACCAATGG | 0.0223 | 0.0023 | 0.6897 | 0.7500 | 1.0000 | 1 | 1 |
| 41 | 54 | CAGATCAACTTACTCCTAC | GTAGGAGTAAGTTGATCTG | 0.5254 | 0.3532 | 0.6034 | 0.5000 | 1.0000 | 1 | 1 |
| 41 | 45 | CTATTCATGCAGATCAACT | AGTTGATCTGCATGAATAG | 0.2849 | 0.0585 | 0.4655 | 0.5000 | 1.0000 | 1 | 1 |
| 41 | 35 | GTCCCTGTTGCTATTCATG | CATGAATAGCAACAGGGAC | 0.1143 | 0.0013 | 0.6638 | 0.5000 | 1.0000 | 1 | 1 |
| 42 | 78 | GTGTTTATTCTACAGGTTC | GAACCTGTAGAATAAACAC | 0.1080 | 0.0189 | 0.6207 | 0.5000 | 1.0000 | 1 | 1 |
| 42 | 41 | GTTGCTATTCATGCAGATC | GATCTGCATGAATAGCAAC | 0.1038 | 0.0077 | 0.4655 | 0.5000 | 0.9185 | 0.75 | 1 |
| 42 | 76 | GCGTGTTTATTCTACAGGT | ACCTGTAGAATAAACACGC | 0.0779 | 0.0011 | 0.569 | 0.7500 | 1.0000 | 1 | 1 |
| 42 | 31 | AGAAGTCCCTGTTGCTATT | AATAGCAACAGGGACTTCT | 0.0198 | 0.0019 | 0.7155 | 0.7500 | 1.0000 | 1 | 1 |

**List of siRNAs predicted by RNAxs for the ‘conserved region 28’ of the ‘S’ gene**

| **WORST RANK** | **Position** | **Target sequence** | **siRNA sequence** | **Access 8nts** | **Access 16nts** | **Energy A.** | **Sequence A.** | **Self-Folding** | **Free End** |  |
| --- | --- | --- | --- | --- | --- | --- | --- | --- | --- | --- |
|  |  |  |  |  |  |  |  |  |  |  |
| 9 | 196 | GGTTTTAATTTTTCACAAA | TTTGTGAAAAATTAAAACC | 0.8832 | 0.4739 | 0.8534 | 1.0000 | 1.0000 | 1 | 1 |
| 17 | 148 | GCACAAGTCAAACAAATTT | AAATTTGTTTGACTTGTGC | 0.7285 | 0.4440 | 0.8534 | 1.0000 | 1.0000 | 1 | 1 |
| 18 | 147 | TGCACAAGTCAAACAAATT | AATTTGTTTGACTTGTGCA | 0.7234 | 0.4431 | 0.8448 | 0.7500 | 1.0000 | 1 | 1 |
| 22 | 153 | AGTCAAACAAATTTACAAA | TTTGTAAATTTGTTTGACT | 0.6230 | 0.3530 | 0.7069 | 0.7500 | 1.0000 | 1 | 1 |
| 29 | 172 | ACACCACCAATTAAAGATT | AATCTTTAATTGGTGGTGT | 0.9104 | 0.0603 | 0.6983 | 0.7500 | 1.0000 | 1 | 1 |
| 30 | 195 | TGGTTTTAATTTTTCACAA | TTGTGAAAAATTAAAACCA | 0.8844 | 0.0602 | 0.6983 | 0.7500 | 1.0000 | 1 | 1 |
| 31 | 197 | GTTTTAATTTTTCACAAAT | ATTTGTGAAAAATTAAAAC | 0.8842 | 0.4772 | 0.6379 | 0.7500 | 1.0000 | 1 | 1 |
| 32 | 167 | ACAAAACACCACCAATTAA | TTAATTGGTGGTGTTTTGT | 0.1698 | 0.1581 | 0.6724 | 0.7500 | 1.0000 | 1 | 1 |
| 33 | 154 | GTCAAACAAATTTACAAAA | TTTTGTAAATTTGTTTGAC | 0.6062 | 0.3807 | 0.819 | 0.7500 | 1.0000 | 1 | 1 |
| 34 | 51 | CAATCTTTTGTTGCAATAT | ATATTGCAACAAAAGATTG | 0.1548 | 0.0507 | 0.6034 | 0.7500 | 1.0000 | 1 | 1 |
| 35 | 149 | CACAAGTCAAACAAATTTA | TAAATTTGTTTGACTTGTG | 0.7450 | 0.4526 | 0.7586 | 0.7500 | 1.0000 | 1 | 1 |
| 35 | 168 | CAAAACACCACCAATTAAA | TTTAATTGGTGGTGTTTTG | 0.1500 | 0.1383 | 0.6466 | 0.7500 | 1.0000 | 1 | 1 |
| 41 | 74 | GTTTTTGTACACAATTAAA | TTTAATTGTGTACAAAAAC | 0.7716 | 0.0188 | 0.6466 | 0.7500 | 1.0000 | 1 | 1 |
| 42 | 73 | AGTTTTTGTACACAATTAA | TTAATTGTGTACAAAAACT | 0.7699 | 0.0155 | 0.6724 | 0.7500 | 1.0000 | 1 | 1 |
| 44 | 120 | ACAAGACAAAAACACCCAA | TTGGGTGTTTTTGTCTTGT | 0.1404 | 0.0140 | 0.5603 | 0.7500 | 1.0000 | 1 | 1 |
| 44 | 105 | TGGAATAGCTGTTGAACAA | TTGTTCAACAGCTATTCCA | 0.1008 | 0.0852 | 0.6983 | 0.7500 | 1.0000 | 1 | 1 |
| 44 | 208 | TCACAAATATTACCAGATC | GATCTGGTAATATTTGTGA | 0.8449 | 0.7810 | 0.5431 | 0.5000 | 1.0000 | 1 | 1 |
| 45 | 173 | CACCACCAATTAAAGATTT | AAATCTTTAATTGGTGGTG | 0.0931 | 0.0415 | 0.7931 | 0.7500 | 1.0000 | 1 | 1 |
| 46 | 106 | GGAATAGCTGTTGAACAAG | CTTGTTCAACAGCTATTCC | 0.0747 | 0.0568 | 0.7328 | 0.7500 | 1.0000 | 1 | 1 |
| 46 | 22 | TACATTTGTGGTGATTCAA | TTGAATCACCACAAATGTA | 0.1768 | 0.0555 | 0.5345 | 0.5000 | 1.0000 | 1 | 1 |
| 49 | 104 | CTGGAATAGCTGTTGAACA | TGTTCAACAGCTATTCCAG | 0.1310 | 0.0899 | 0.5776 | 0.5000 | 1.0000 | 1 | 1 |
| 49 | 150 | ACAAGTCAAACAAATTTAC | GTAAATTTGTTTGACTTGT | 0.7857 | 0.4451 | 0.5259 | 0.5000 | 1.0000 | 1 | 1 |
| 49 | 103 | ACTGGAATAGCTGTTGAAC | GTTCAACAGCTATTCCAGT | 0.1105 | 0.0122 | 0.5603 | 0.5000 | 1.0000 | 1 | 1 |
| 50 | 166 | TACAAAACACCACCAATTA | TAATTGGTGGTGTTTTGTA | 0.1847 | 0.1732 | 0.6466 | 0.5000 | 1.0000 | 1 | 1 |
| 51 | 135 | CCAAGAAGTTTTTGCACAA | TTGTGCAAAAACTTCTTGG | 0.0527 | 0.0132 | 0.6983 | 1.0000 | 1.0000 | 1 | 1 |
| 51 | 19 | ATGTACATTTGTGGTGATT | AATCACCACAAATGTACAT | 0.2266 | 0.1332 | 0.6466 | 0.5000 | 1.0000 | 1 | 1 |
| 52 | 215 | TATTACCAGATCCATCAAA | TTTGATGGATCTGGTAATA | 0.8989 | 0.7941 | 0.5431 | 0.5000 | 1.0000 | 1 | 1 |
| 52 | 121 | CAAGACAAAAACACCCAAG | CTTGGGTGTTTTTGTCTTG | 0.1320 | 0.0119 | 0.5086 | 0.5000 | 1.0000 | 1 | 1 |
| 53 | 124 | GACAAAAACACCCAAGAAG | CTTCTTGGGTGTTTTTGTC | 0.0705 | 0.0147 | 0.681 | 0.5000 | 1.0000 | 1 | 1 |
| 53 | 136 | CAAGAAGTTTTTGCACAAG | CTTGTGCAAAAACTTCTTG | 0.0643 | 0.0128 | 0.5086 | 0.5000 | 1.0000 | 1 | 1 |
| 53 | 110 | TAGCTGTTGAACAAGACAA | TTGTCTTGTTCAACAGCTA | 0.0515 | 0.0112 | 0.5259 | 0.5000 | 1.0000 | 1 | 1 |
| 54 | 23 | ACATTTGTGGTGATTCAAC | GTTGAATCACCACAAATGT | 0.1267 | 0.0539 | 0.5603 | 0.5000 | 1.0000 | 1 | 1 |
| 54 | 72 | CAGTTTTTGTACACAATTA | TAATTGTGTACAAAAACTG | 0.0503 | 0.0124 | 0.75 | 0.7500 | 1.0000 | 1 | 1 |
| 55 | 20 | TGTACATTTGTGGTGATTC | GAATCACCACAAATGTACA | 0.1623 | 0.1263 | 0.5345 | 0.5000 | 1.0000 | 1 | 1 |
| 55 | 125 | ACAAAAACACCCAAGAAGT | ACTTCTTGGGTGTTTTTGT | 0.0615 | 0.0393 | 0.5 | 0.5000 | 1.0000 | 1 | 1 |
| 56 | 111 | AGCTGTTGAACAAGACAAA | TTTGTCTTGTTCAACAGCT | 0.0525 | 0.0103 | 0.8534 | 0.7500 | 1.0000 | 1 | 1 |
| 57 | 126 | CAAAAACACCCAAGAAGTT | AACTTCTTGGGTGTTTTTG | 0.0492 | 0.0303 | 0.5 | 0.7500 | 1.0000 | 1 | 1 |
| 57 | 114 | TGTTGAACAAGACAAAAAC | GTTTTTGTCTTGTTCAACA | 0.0430 | 0.0099 | 0.5603 | 0.5000 | 1.0000 | 1 | 1 |
| 58 | 200 | TTAATTTTTCACAAATATT | AATATTTGTGAAAAATTAA | 0.8573 | 0.8193 | 0.5259 | 0.5000 | 1.0000 | 1 | 1 |
| 59 | 112 | GCTGTTGAACAAGACAAAA | TTTTGTCTTGTTCAACAGC | 0.0393 | 0.0108 | 0.8534 | 1.0000 | 1.0000 | 1 | 1 |
| 59 | 123 | AGACAAAAACACCCAAGAA | TTCTTGGGTGTTTTTGTCT | 0.8232 | 0.0095 | 0.5517 | 0.7500 | 1.0000 | 1 | 1 |
| 60 | 28 | TGTGGTGATTCAACTGAAT | ATTCAGTTGAATCACCACA | 0.0387 | 0.0129 | 0.6983 | 0.7500 | 1.0000 | 1 | 1 |
| 60 | 152 | AAGTCAAACAAATTTACAA | TTGTAAATTTGTTTGACTT | 0.6999 | 0.3971 | 0.4914 | 0.5000 | 1.0000 | 1 | 1 |
| 60 | 71 | GCAGTTTTTGTACACAATT | AATTGTGTACAAAAACTGC | 0.0560 | 0.0082 | 0.8448 | 1.0000 | 1.0000 | 1 | 1 |
| 61 | 207 | TTCACAAATATTACCAGAT | ATCTGGTAATATTTGTGAA | 0.8355 | 0.7850 | 0.4914 | 0.5000 | 1.0000 | 1 | 1 |
| 62 | 113 | CTGTTGAACAAGACAAAAA | TTTTTGTCTTGTTCAACAG | 0.0369 | 0.0103 | 0.7845 | 0.7500 | 1.0000 | 1 | 1 |
| 62 | 216 | ATTACCAGATCCATCAAAA | TTTTGATGGATCTGGTAAT | 0.9009 | 0.8060 | 0.5086 | 0.5000 | 1.0000 | 1 | 1 |
| 62 | 211 | CAAATATTACCAGATCCAT | ATGGATCTGGTAATATTTG | 0.8750 | 0.7675 | 0.4914 | 0.7500 | 1.0000 | 1 | 1 |
| 62 | 97 | GCTTTAACTGGAATAGCTG | CAGCTATTCCAGTTAAAGC | 0.2399 | 0.0056 | 0.569 | 0.7500 | 0.9946 | 0.625 | 1 |
| 63 | 70 | GGCAGTTTTTGTACACAAT | ATTGTGTACAAAAACTGCC | 0.1037 | 0.0036 | 0.9914 | 1.0000 | 1.0000 | 1 | 1 |
| 63 | 165 | TTACAAAACACCACCAATT | AATTGGTGGTGTTTTGTAA | 0.1989 | 0.1847 | 0.5259 | 0.5000 | 1.0000 | 1 | 1 |
| 63 | 164 | TTTACAAAACACCACCAAT | ATTGGTGGTGTTTTGTAAA | 0.2145 | 0.1985 | 0.4914 | 0.5000 | 1.0000 | 1 | 1 |
| 63 | 134 | CCCAAGAAGTTTTTGCACA | TGTGCAAAAACTTCTTGGG | 0.0366 | 0.0118 | 0.7845 | 0.7500 | 1.0000 | 1 | 1 |
| 64 | 94 | CGTGCTTTAACTGGAATAG | CTATTCCAGTTAAAGCACG | 0.1192 | 0.0021 | 0.6034 | 0.7500 | 1.0000 | 1 | 1 |
| 64 | 201 | TAATTTTTCACAAATATTA | TAATATTTGTGAAAAATTA | 0.8537 | 0.8207 | 0.5 | 0.5000 | 1.0000 | 1 | 1 |
| 64 | 140 | AAGTTTTTGCACAAGTCAA | TTGACTTGTGCAAAAACTT | 0.4866 | 0.0229 | 0.4914 | 0.5000 | 1.0000 | 1 | 1 |
| 65 | 93 | CCGTGCTTTAACTGGAATA | TATTCCAGTTAAAGCACGG | 0.0382 | 0.0018 | 0.8707 | 1.0000 | 1.0000 | 1 | 1 |
| 65 | 102 | AACTGGAATAGCTGTTGAA | TTCAACAGCTATTCCAGTT | 0.1178 | 0.0078 | 0.4741 | 0.5000 | 1.0000 | 1 | 1 |
| 66 | 146 | TTGCACAAGTCAAACAAAT | ATTTGTTTGACTTGTGCAA | 0.5694 | 0.3041 | 0.6379 | 0.5000 | 1.0000 | 1 | 1 |
| 66 | 174 | ACCACCAATTAAAGATTTT | AAAATCTTTAATTGGTGGT | 0.0302 | 0.0245 | 0.8534 | 0.7500 | 1.0000 | 1 | 1 |
| 66 | 69 | TGGCAGTTTTTGTACACAA | TTGTGTACAAAAACTGCCA | 0.0427 | 0.0017 | 0.6983 | 0.7500 | 1.0000 | 1 | 1 |
| 67 | 155 | TCAAACAAATTTACAAAAC | GTTTTGTAAATTTGTTTGA | 0.6093 | 0.5316 | 0.5776 | 0.5000 | 1.0000 | 1 | 1 |
| 67 | 27 | TTGTGGTGATTCAACTGAA | TTCAGTTGAATCACCACAA | 0.1839 | 0.0618 | 0.4741 | 0.5000 | 1.0000 | 1 | 1 |
| 67 | 133 | ACCCAAGAAGTTTTTGCAC | GTGCAAAAACTTCTTGGGT | 0.0297 | 0.0108 | 0.5603 | 0.5000 | 1.0000 | 1 | 1 |
| 67 | 92 | ACCGTGCTTTAACTGGAAT | ATTCCAGTTAAAGCACGGT | 0.0440 | 0.0016 | 0.8448 | 0.7500 | 1.0000 | 1 | 1 |
| 68 | 127 | AAAAACACCCAAGAAGTTT | AAACTTCTTGGGTGTTTTT | 0.0334 | 0.0202 | 0.5 | 0.5000 | 1.0000 | 1 | 1 |
| 68 | 171 | AACACCACCAATTAAAGAT | ATCTTTAATTGGTGGTGTT | 0.9294 | 0.0818 | 0.4655 | 0.5000 | 1.0000 | 1 | 1 |
| 68 | 91 | AACCGTGCTTTAACTGGAA | TTCCAGTTAAAGCACGGTT | 0.0350 | 0.0015 | 0.4741 | 0.5000 | 1.0000 | 1 | 1 |
| 68 | 129 | AAACACCCAAGAAGTTTTT | AAAAACTTCTTGGGTGTTT | 0.0192 | 0.0097 | 0.5 | 0.5000 | 1.0000 | 1 | 1 |

**List of siRNAs predicted by RNAxs for the ‘conserved region 29’ of the ‘S’ gene**

| **WORST RANK** | **Position** | **Target sequence** | **siRNA sequence** | **Access 8nts** | **Access 16nts** | **Energy A.** | **Sequence A.** | **Self -Folding** | **Free End** |  |
| --- | --- | --- | --- | --- | --- | --- | --- | --- | --- | --- |
|  |  |  |  |  |  |  |  |  |  |  |
| 0 | 55 | CAAAGTGACACTTGCAGAT | ATCTGCAAGTGTCACTTTG | 0.1509 | 0.0254 | 0.4655 | 0.7500 | 1.0000 | 1 | 1 |

**List of siRNAs predicted by RNAxs for the ‘conserved region 30’ of the ‘S’ gene**

| **WORST RANK** | **Position** | **Target sequence** | **siRNA sequence** | **Access 8nts** | **Access 16nts** | **Energy A.** | **Sequence A.** | **Self-Folding** | **Free End** |  |
| --- | --- | --- | --- | --- | --- | --- | --- | --- | --- | --- |
|  |  |  |  |  |  |  |  |  |  |  |
| 6 | 84 | GATGAAATGATTGCTCAAT | ATTGAGCAATCATTTCATC | 0.2387 | 0.1007 | 0.6724 | 0.7500 | 0.9783 | 0.75 | 1 |
| 14 | 72 | CCTTTGCTCACAGATGAAA | TTTCATCTGTGAGCAAAGG | 0.1641 | 0.0437 | 0.8448 | 1.0000 | 1.0000 | 1 | 1 |
| 15 | 73 | CTTTGCTCACAGATGAAAT | ATTTCATCTGTGAGCAAAG | 0.1135 | 0.0327 | 0.6293 | 0.7500 | 1.0000 | 1 | 1 |
| 15 | 83 | AGATGAAATGATTGCTCAA | TTGAGCAATCATTTCATCT | 0.2449 | 0.1058 | 0.5776 | 0.7500 | 0.9783 | 0.625 | 1 |
| 19 | 31 | TCATTTGTGCACAAAAGTT | AACTTTTGTGCACAAATGA | 0.1083 | 0.0179 | 0.5776 | 0.7500 | 1.0000 | 1 | 1 |
| 20 | 21 | GCTAGAGACCTCATTTGTG | CACAAATGAGGTCTCTAGC | 0.0919 | 0.0488 | 0.5603 | 0.7500 | 1.0000 | 1 | 1 |
| 20 | 32 | CATTTGTGCACAAAAGTTT | AAACTTTTGTGCACAAATG | 0.1131 | 0.0175 | 0.6552 | 0.7500 | 1.0000 | 1 | 1 |
| 21 | 26 | AGACCTCATTTGTGCACAA | TTGTGCACAAATGAGGTCT | 0.0737 | 0.0552 | 0.5776 | 0.7500 | 1.0000 | 1 | 1 |
| 22 | 19 | CTGCTAGAGACCTCATTTG | CAAATGAGGTCTCTAGCAG | 0.4192 | 0.1381 | 0.6379 | 0.5000 | 1.0000 | 1 | 1 |
| 22 | 86 | TGAAATGATTGCTCAATAC | GTATTGAGCAATCATTTCA | 0.2133 | 0.1631 | 0.5431 | 0.5000 | 0.9783 | 1 | 1 |
| 25 | 27 | GACCTCATTTGTGCACAAA | TTTGTGCACAAATGAGGTC | 0.0667 | 0.0584 | 0.819 | 0.7500 | 1.0000 | 1 | 1 |
| 25 | 90 | ATGATTGCTCAATACACTT | AAGTGTATTGAGCAATCAT | 0.2426 | 0.0846 | 0.5172 | 0.5000 | 0.9783 | 0.625 | 1 |
| 25 | 41 | ACAAAAGTTTAACGGCCTT | AAGGCCGTTAAACTTTTGT | 0.0910 | 0.0064 | 0.569 | 0.7500 | 1.0000 | 1 | 1 |
| 26 | 20 | TGCTAGAGACCTCATTTGT | ACAAATGAGGTCTCTAGCA | 0.4474 | 0.2062 | 0.6466 | 0.5000 | 1.0000 | 1 | 1 |
| 26 | 28 | ACCTCATTTGTGCACAAAA | TTTTGTGCACAAATGAGGT | 0.0661 | 0.0607 | 0.8534 | 0.7500 | 1.0000 | 1 | 1 |
| 27 | 91 | TGATTGCTCAATACACTTC | GAAGTGTATTGAGCAATCA | 0.1057 | 0.0296 | 0.5517 | 0.5000 | 1.0000 | 1 | 1 |
| 27 | 47 | GTTTAACGGCCTTACTGTT | AACAGTAAGGCCGTTAAAC | 0.0972 | 0.0021 | 0.5 | 0.7500 | 1.0000 | 1 | 1 |
| 28 | 82 | CAGATGAAATGATTGCTCA | TGAGCAATCATTTCATCTG | 0.2373 | 0.0803 | 0.5603 | 0.5000 | 0.9891 | 0.625 | 1 |
| 28 | 49 | TTAACGGCCTTACTGTTTT | AAAACAGTAAGGCCGTTAA | 0.0977 | 0.0021 | 0.5345 | 0.5000 | 1.0000 | 1 | 1 |
| 29 | 85 | ATGAAATGATTGCTCAATA | TATTGAGCAATCATTTCAT | 0.2324 | 0.1305 | 0.6121 | 0.5000 | 0.9783 | 0.875 | 1 |
| 30 | 48 | TTTAACGGCCTTACTGTTT | AAACAGTAAGGCCGTTAAA | 0.0972 | 0.0023 | 0.5 | 0.5000 | 1.0000 | 1 | 1 |
| 30 | 30 | CTCATTTGTGCACAAAAGT | ACTTTTGTGCACAAATGAG | 0.0439 | 0.0183 | 0.6034 | 0.5000 | 1.0000 | 1 | 1 |
| 30 | 57 | CTTACTGTTTTGCCACCTT | AAGGTGGCAAAACAGTAAG | 0.0697 | 0.0018 | 0.5 | 0.7500 | 0.9783 | 0.75 | 1 |
| 31 | 46 | AGTTTAACGGCCTTACTGT | ACAGTAAGGCCGTTAAACT | 0.0954 | 0.0020 | 0.5 | 0.5000 | 1.0000 | 1 | 1 |
| 31 | 111 | GCACTGTTAGCGGGTACAA | TTGTACCCGCTAACAGTGC | 0.0337 | 0.0119 | 0.7069 | 1.0000 | 0.9565 | 0.625 | 1 |
| 31 | 33 | ATTTGTGCACAAAAGTTTA | TAAACTTTTGTGCACAAAT | 0.0566 | 0.0134 | 0.4741 | 0.5000 | 1.0000 | 1 | 1 |
| 31 | 55 | GCCTTACTGTTTTGCCACC | GGTGGCAAAACAGTAAGGC | 0.0831 | 0.0018 | 0.6466 | 0.5000 | 0.9946 | 0.625 | 1 |
| 32 | 42 | CAAAAGTTTAACGGCCTTA | TAAGGCCGTTAAACTTTTG | 0.0293 | 0.0076 | 0.6121 | 0.7500 | 1.0000 | 1 | 1 |
| 32 | 25 | GAGACCTCATTTGTGCACA | TGTGCACAAATGAGGTCTC | 0.0670 | 0.0541 | 0.6034 | 0.5000 | 1.0000 | 1 | 1 |
| 32 | 87 | GAAATGATTGCTCAATACA | TGTATTGAGCAATCATTTC | 0.3055 | 0.1563 | 0.4655 | 0.5000 | 0.9783 | 1 | 1 |
| 33 | 58 | TTACTGTTTTGCCACCTTT | AAAGGTGGCAAAACAGTAA | 0.0448 | 0.0017 | 0.5345 | 0.5000 | 0.9783 | 0.75 | 1 |
| 33 | 56 | CCTTACTGTTTTGCCACCT | AGGTGGCAAAACAGTAAGG | 0.0719 | 0.0016 | 0.5 | 0.7500 | 0.9783 | 0.75 | 1 |
| 33 | 29 | CCTCATTTGTGCACAAAAG | CTTTTGTGCACAAATGAGG | 0.0254 | 0.0182 | 0.7069 | 0.7500 | 1.0000 | 1 | 1 |
| 33 | 34 | TTTGTGCACAAAAGTTTAA | TTAAACTTTTGTGCACAAA | 0.0511 | 0.0119 | 0.4655 | 0.5000 | 1.0000 | 1 | 1 |

**List of siRNAs predicted by RNAxs for the ‘conserved region 31’ of the ‘S’ gene**

| **WORST RANK** | **Position** | **Target sequence** | **siRNA sequence** | **Access 8nts** | **Access 16nts** | **Energy A.** | **Sequence A.** | **Self-Folding** | **Free End** |  |
| --- | --- | --- | --- | --- | --- | --- | --- | --- | --- | --- |
|  |  |  |  |  |  |  |  |  |  |  |
| 9 | 25 | CAGGTGCTGCATTACAAAT | ATTTGTAATGCAGCACCTG | 0.2530 | 0.0332 | 0.7759 | 0.7500 | 1.0000 | 1 | 1 |
| 10 | 62 | GGCTTATAGGTTTAATGGT | ACCATTAAACCTATAAGCC | 0.0811 | 0.0410 | 0.6466 | 0.7500 | 1.0000 | 1 | 1 |
| 11 | 63 | GCTTATAGGTTTAATGGTA | TACCATTAAACCTATAAGC | 0.0796 | 0.0417 | 0.6724 | 1.0000 | 1.0000 | 1 | 1 |
| 12 | 26 | AGGTGCTGCATTACAAATA | TATTTGTAATGCAGCACCT | 0.2935 | 0.0366 | 0.8017 | 0.7500 | 1.0000 | 1 | 1 |
| 12 | 64 | CTTATAGGTTTAATGGTAT | ATACCATTAAACCTATAAG | 0.0771 | 0.0441 | 0.5948 | 0.7500 | 1.0000 | 1 | 1 |
| 13 | 78 | GGTATTGGAGTTACACAGA | TCTGTGTAACTCCAATACC | 0.0838 | 0.0117 | 0.6293 | 0.7500 | 1.0000 | 1 | 1 |
| 14 | 83 | TGGAGTTACACAGAATGTT | AACATTCTGTGTAACTCCA | 0.0740 | 0.0112 | 0.6983 | 0.7500 | 0.9837 | 0.625 | 1 |
| 16 | 84 | GGAGTTACACAGAATGTTC | GAACATTCTGTGTAACTCC | 0.0427 | 0.0080 | 0.6983 | 0.7500 | 0.9837 | 0.75 | 1 |
| 17 | 77 | TGGTATTGGAGTTACACAG | CTGTGTAACTCCAATACCA | 0.0765 | 0.0106 | 0.5603 | 0.5000 | 1.0000 | 1 | 1 |
| 18 | 79 | GTATTGGAGTTACACAGAA | TTCTGTGTAACTCCAATAC | 0.1033 | 0.0166 | 0.5086 | 0.7500 | 1.0000 | 1 | 1 |
| 18 | 104 | CTATGAGAACCAAAAATTG | CAATTTTTGGTTCTCATAG | 0.4770 | 0.0040 | 0.5259 | 0.5000 | 1.0000 | 1 | 1 |
| 19 | 71 | GTTTAATGGTATTGGAGTT | AACTCCAATACCATTAAAC | 0.0288 | 0.0104 | 0.5 | 0.7500 | 1.0000 | 1 | 1 |
| 19 | 65 | TTATAGGTTTAATGGTATT | AATACCATTAAACCTATAA | 0.0350 | 0.0202 | 0.5259 | 0.5000 | 1.0000 | 1 | 1 |
| 19 | 103 | TCTATGAGAACCAAAAATT | AATTTTTGGTTCTCATAGA | 0.4003 | 0.0014 | 0.7155 | 0.7500 | 1.0000 | 1 | 1 |
| 19 | 85 | GAGTTACACAGAATGTTCT | AGAACATTCTGTGTAACTC | 0.0269 | 0.0043 | 0.5862 | 0.5000 | 0.9837 | 0.875 | 1 |
| 20 | 70 | GGTTTAATGGTATTGGAGT | ACTCCAATACCATTAAACC | 0.0232 | 0.0104 | 0.6466 | 0.7500 | 1.0000 | 1 | 1 |
| 20 | 80 | TATTGGAGTTACACAGAAT | ATTCTGTGTAACTCCAATA | 0.2059 | 0.0469 | 0.5345 | 0.5000 | 1.0000 | 1 | 1 |
| 20 | 106 | ATGAGAACCAAAAATTGAT | ATCAATTTTTGGTTCTCAT | 0.8832 | 0.0043 | 0.4741 | 0.5000 | 1.0000 | 1 | 1 |
| 20 | 56 | GCAAATGGCTTATAGGTTT | AAACCTATAAGCCATTTGC | 0.2752 | 0.0011 | 0.8534 | 1.0000 | 1.0000 | 1 | 1 |
| 21 | 69 | AGGTTTAATGGTATTGGAG | CTCCAATACCATTAAACCT | 0.0205 | 0.0082 | 0.5345 | 0.5000 | 1.0000 | 1 | 1 |
| 21 | 72 | TTTAATGGTATTGGAGTTA | TAACTCCAATACCATTAAA | 0.0617 | 0.0102 | 0.4655 | 0.5000 | 1.0000 | 1 | 1 |
| 21 | 57 | CAAATGGCTTATAGGTTTA | TAAACCTATAAGCCATTTG | 0.2440 | 0.0010 | 0.6121 | 0.7500 | 1.0000 | 1 | 1 |

**List of siRNAs predicted by RNAxs for the ‘conserved region 33’ of the ‘S’ gene**

| **WORST RANK** | **Position** | **Target sequence** | **siRNA sequence** | **Access 8nts** | **Access 16nts** | **Energy A.** | **Sequence A.** | **Self-Folding** | **Free End** |  |
| --- | --- | --- | --- | --- | --- | --- | --- | --- | --- | --- |
|  |  |  |  |  |  |  |  |  |  |  |
| 8 | 68 | TCAAGTGTTTTAAATGATA | TATCATTTAAAACACTTGA | 0.4629 | 0.0988 | 0.681 | 0.7500 | 1.0000 | 1 | 1 |
| 9 | 62 | GCAATTTCAAGTGTTTTAA | TTAAAACACTTGAAATTGC | 0.1281 | 0.0937 | 0.819 | 1.0000 | 1.0000 | 1 | 1 |
| 11 | 63 | CAATTTCAAGTGTTTTAAA | TTTAAAACACTTGAAATTG | 0.1422 | 0.0988 | 0.6466 | 0.7500 | 1.0000 | 1 | 1 |
| 11 | 61 | TGCAATTTCAAGTGTTTTA | TAAAACACTTGAAATTGCA | 0.1202 | 0.0926 | 0.819 | 0.7500 | 1.0000 | 1 | 1 |
| 12 | 19 | AGCTTTAAACACGCTTGTT | AACAAGCGTGTTTAAAGCT | 0.1147 | 0.1132 | 0.7069 | 0.7500 | 0.9348 | 0.625 | 1 |
| 12 | 69 | CAAGTGTTTTAAATGATAT | ATATCATTTAAAACACTTG | 0.4632 | 0.0992 | 0.6034 | 0.7500 | 1.0000 | 1 | 1 |
| 13 | 73 | TGTTTTAAATGATATCCTT | AAGGATATCATTTAAAACA | 0.4768 | 0.1446 | 0.569 | 0.7500 | 1.0000 | 1 | 1 |
| 13 | 28 | CACGCTTGTTAAACAACTT | AAGTTGTTTAACAAGCGTG | 0.2715 | 0.0473 | 0.6552 | 0.7500 | 0.9728 | 0.625 | 1 |
| 14 | 29 | ACGCTTGTTAAACAACTTA | TAAGTTGTTTAACAAGCGT | 0.3025 | 0.0469 | 0.7414 | 0.7500 | 0.9728 | 0.625 | 1 |
| 14 | 27 | ACACGCTTGTTAAACAACT | AGTTGTTTAACAAGCGTGT | 0.2739 | 0.0487 | 0.5 | 0.5000 | 0.9728 | 0.625 | 1 |
| 15 | 22 | TTTAAACACGCTTGTTAAA | TTTAACAAGCGTGTTTAAA | 0.0758 | 0.0531 | 0.5 | 0.5000 | 1.0000 | 1 | 1 |
| 15 | 60 | GTGCAATTTCAAGTGTTTT | AAAACACTTGAAATTGCAC | 0.1208 | 0.0016 | 0.7931 | 0.7500 | 1.0000 | 1 | 1 |
| 16 | 59 | GGTGCAATTTCAAGTGTTT | AAACACTTGAAATTGCACC | 0.1059 | 0.0013 | 0.8534 | 1.0000 | 1.0000 | 1 | 1 |
| 16 | 64 | AATTTCAAGTGTTTTAAAT | ATTTAAAACACTTGAAATT | 0.1327 | 0.0989 | 0.5 | 0.5000 | 1.0000 | 1 | 1 |
| 16 | 20 | GCTTTAAACACGCTTGTTA | TAACAAGCGTGTTTAAAGC | 0.0711 | 0.0703 | 0.819 | 1.0000 | 1.0000 | 1 | 1 |
| 17 | 67 | TTCAAGTGTTTTAAATGAT | ATCATTTAAAACACTTGAA | 0.4598 | 0.0988 | 0.4914 | 0.5000 | 1.0000 | 1 | 1 |
| 17 | 58 | TGGTGCAATTTCAAGTGTT | AACACTTGAAATTGCACCA | 0.1040 | 0.0013 | 0.6983 | 0.7500 | 1.0000 | 1 | 1 |
| 17 | 21 | CTTTAAACACGCTTGTTAA | TTAACAAGCGTGTTTAAAG | 0.0574 | 0.0564 | 0.6034 | 0.7500 | 1.0000 | 1 | 1 |

**List of siRNAs predicted by RNAxs for the ‘conserved region 34’ of the ‘S’ gene**

| **WORST RANK** | **Position** | **Target sequence** | **siRNA sequence** | **Access 8nts** | **Access 16nts** | **Energy A.** | **Sequence A.** | **Self-Folding** | **Free End** |  |
| --- | --- | --- | --- | --- | --- | --- | --- | --- | --- | --- |
|  |  |  |  |  |  |  |  |  |  |  |
| 12 | 95 | TGTGACTCAACAATTAATT | AATTAATTGTTGAGTCACA | 0.6886 | 0.1026 | 0.6983 | 0.7500 | 1.0000 | 1 | 1 |
| 17 | 138 | GCTAATCTTGCTGCTACTA | TAGTAGCAGCAAGATTAGC | 0.2915 | 0.1085 | 0.681 | 1.0000 | 1.0000 | 1 | 1 |
| 21 | 96 | GTGACTCAACAATTAATTA | TAATTAATTGTTGAGTCAC | 0.6447 | 0.0699 | 0.7586 | 0.7500 | 1.0000 | 1 | 1 |
| 23 | 90 | ACATATGTGACTCAACAAT | ATTGTTGAGTCACATATGT | 0.2428 | 0.0487 | 0.6983 | 0.7500 | 1.0000 | 1 | 1 |
| 26 | 39 | GCTGAAGTGCAAATTGATA | TATCAATTTGCACTTCAGC | 0.1699 | 0.0301 | 0.8103 | 1.0000 | 1.0000 | 1 | 1 |
| 27 | 89 | GACATATGTGACTCAACAA | TTGTTGAGTCACATATGTC | 0.2252 | 0.0465 | 0.6724 | 0.7500 | 1.0000 | 1 | 1 |
| 27 | 38 | GGCTGAAGTGCAAATTGAT | ATCAATTTGCACTTCAGCC | 0.1661 | 0.0240 | 0.819 | 1.0000 | 0.9837 | 0.75 | 1 |
| 29 | 91 | CATATGTGACTCAACAATT | AATTGTTGAGTCACATATG | 0.3392 | 0.0509 | 0.6466 | 0.7500 | 1.0000 | 1 | 1 |
| 29 | 230 | CCCTCAGTCAGCACCTCAT | ATGAGGTGCTGACTGAGGG | 0.1521 | 0.0232 | 0.8362 | 1.0000 | 0.9891 | 1 | 1 |
| 31 | 139 | CTAATCTTGCTGCTACTAA | TTAGTAGCAGCAAGATTAG | 0.3152 | 0.1222 | 0.6379 | 0.7500 | 1.0000 | 1 | 1 |
| 37 | 137 | TGCTAATCTTGCTGCTACT | AGTAGCAGCAAGATTAGCA | 0.2318 | 0.0551 | 0.6466 | 0.5000 | 0.9783 | 0.75 | 1 |
| 38 | 142 | ATCTTGCTGCTACTAAAAT | ATTTTAGTAGCAGCAAGAT | 0.2562 | 0.0537 | 0.6724 | 0.5000 | 1.0000 | 1 | 1 |
| 40 | 231 | CCTCAGTCAGCACCTCATG | CATGAGGTGCTGACTGAGG | 0.0791 | 0.0132 | 0.6897 | 0.7500 | 0.9891 | 1 | 1 |
| 41 | 86 | GCAGACATATGTGACTCAA | TTGAGTCACATATGTCTGC | 0.0765 | 0.0383 | 0.7069 | 1.0000 | 1.0000 | 1 | 1 |
| 41 | 98 | GACTCAACAATTAATTAGA | TCTAATTAATTGTTGAGTC | 0.1347 | 0.0428 | 0.5948 | 0.5000 | 1.0000 | 1 | 1 |
| 43 | 45 | GTGCAAATTGATAGGTTGA | TCAACCTATCAATTTGCAC | 0.1023 | 0.0183 | 0.569 | 0.5000 | 1.0000 | 1 | 1 |
| 44 | 40 | CTGAAGTGCAAATTGATAG | CTATCAATTTGCACTTCAG | 0.1740 | 0.0272 | 0.6121 | 0.5000 | 1.0000 | 1 | 1 |
| 47 | 132 | GCTTCTGCTAATCTTGCTG | CAGCAAGATTAGCAGAAGC | 0.1273 | 0.0081 | 0.569 | 0.7500 | 0.9783 | 0.75 | 1 |
| 48 | 143 | TCTTGCTGCTACTAAAATG | CATTTTAGTAGCAGCAAGA | 0.0622 | 0.0110 | 0.569 | 0.5000 | 1.0000 | 1 | 1 |
| 51 | 34 | TTGAGGCTGAAGTGCAAAT | ATTTGCACTTCAGCCTCAA | 0.0541 | 0.0115 | 0.6379 | 0.5000 | 0.9837 | 1 | 1 |
| 51 | 203 | TGGAAAGGGCTATCATCTT | AAGATGATAGCCCTTTCCA | 0.4348 | 0.0045 | 0.7069 | 0.7500 | 1.0000 | 1 | 1 |
| 52 | 136 | CTGCTAATCTTGCTGCTAC | GTAGCAGCAAGATTAGCAG | 0.2166 | 0.0311 | 0.6034 | 0.5000 | 0.9783 | 0.75 | 1 |
| 52 | 135 | TCTGCTAATCTTGCTGCTA | TAGCAGCAAGATTAGCAGA | 0.2035 | 0.0154 | 0.5517 | 0.7500 | 0.9783 | 0.875 | 1 |
| 52 | 33 | GTTGAGGCTGAAGTGCAAA | TTTGCACTTCAGCCTCAAC | 0.0530 | 0.0105 | 0.6466 | 0.7500 | 0.9837 | 0.875 | 1 |
| 53 | 204 | GGAAAGGGCTATCATCTTA | TAAGATGATAGCCCTTTCC | 0.4428 | 0.0040 | 0.8362 | 1.0000 | 1.0000 | 1 | 1 |
| 53 | 97 | TGACTCAACAATTAATTAG | CTAATTAATTGTTGAGTCA | 0.3599 | 0.0917 | 0.5517 | 0.5000 | 1.0000 | 1 | 1 |
| 54 | 32 | AGTTGAGGCTGAAGTGCAA | TTGCACTTCAGCCTCAACT | 0.0507 | 0.0096 | 0.5603 | 0.7500 | 0.9837 | 0.75 | 1 |
| 54 | 94 | ATGTGACTCAACAATTAAT | ATTAATTGTTGAGTCACAT | 0.6791 | 0.0734 | 0.6466 | 0.5000 | 1.0000 | 1 | 1 |
| 55 | 189 | AGAGTTGATTTTTGTGGAA | TTCCACAAAAATCAACTCT | 0.0998 | 0.0036 | 0.5517 | 0.7500 | 1.0000 | 1 | 1 |
| 55 | 147 | GCTGCTACTAAAATGTCAG | CTGACATTTTAGTAGCAGC | 0.0468 | 0.0045 | 0.569 | 0.7500 | 1.0000 | 1 | 1 |
| 56 | 46 | TGCAAATTGATAGGTTGAT | ATCAACCTATCAATTTGCA | 0.0459 | 0.0211 | 0.6724 | 0.7500 | 1.0000 | 1 | 1 |
| 56 | 76 | TTCAAAGTTTGCAGACATA | TATGTCTGCAAACTTTGAA | 0.0742 | 0.0033 | 0.6293 | 0.5000 | 1.0000 | 1 | 1 |
| 57 | 202 | GTGGAAAGGGCTATCATCT | AGATGATAGCCCTTTCCAC | 0.4189 | 0.0068 | 0.569 | 0.5000 | 1.0000 | 1 | 1 |
| 57 | 37 | AGGCTGAAGTGCAAATTGA | TCAATTTGCACTTCAGCCT | 0.0445 | 0.0151 | 0.6207 | 0.5000 | 0.9837 | 0.75 | 1 |
| 57 | 205 | GAAAGGGCTATCATCTTAT | ATAAGATGATAGCCCTTTC | 0.4494 | 0.0033 | 0.6293 | 0.7500 | 1.0000 | 1 | 1 |
| 58 | 229 | TCCCTCAGTCAGCACCTCA | TGAGGTGCTGACTGAGGGA | 0.2414 | 0.0127 | 0.6466 | 0.5000 | 0.9783 | 0.625 | 1 |
| 59 | 77 | TCAAAGTTTGCAGACATAT | ATATGTCTGCAAACTTTGA | 0.1747 | 0.0032 | 0.681 | 0.7500 | 1.0000 | 1 | 1 |
| 59 | 88 | AGACATATGTGACTCAACA | TGTTGAGTCACATATGTCT | 0.1390 | 0.0434 | 0.5172 | 0.5000 | 1.0000 | 1 | 1 |
| 60 | 87 | CAGACATATGTGACTCAAC | GTTGAGTCACATATGTCTG | 0.1044 | 0.0416 | 0.6379 | 0.5000 | 1.0000 | 1 | 1 |
| 60 | 92 | ATATGTGACTCAACAATTA | TAATTGTTGAGTCACATAT | 0.6471 | 0.0540 | 0.5086 | 0.5000 | 1.0000 | 1 | 1 |
| 61 | 44 | AGTGCAAATTGATAGGTTG | CAACCTATCAATTTGCACT | 0.0749 | 0.0141 | 0.5603 | 0.5000 | 1.0000 | 1 | 1 |
| 61 | 26 | TGACAAAGTTGAGGCTGAA | TTCAGCCTCAACTTTGTCA | 0.0414 | 0.0127 | 0.5517 | 0.7500 | 1.0000 | 1 | 1 |
| 61 | 190 | GAGTTGATTTTTGTGGAAA | TTTCCACAAAAATCAACTC | 0.0816 | 0.0028 | 0.8103 | 0.7500 | 1.0000 | 1 | 1 |
| 62 | 35 | TGAGGCTGAAGTGCAAATT | AATTTGCACTTCAGCCTCA | 0.0391 | 0.0107 | 0.7155 | 0.7500 | 0.9837 | 1 | 1 |
| 63 | 146 | TGCTGCTACTAAAATGTCA | TGACATTTTAGTAGCAGCA | 0.0633 | 0.0058 | 0.6293 | 0.5000 | 1.0000 | 1 | 1 |
| 65 | 36 | GAGGCTGAAGTGCAAATTG | CAATTTGCACTTCAGCCTC | 0.0419 | 0.0134 | 0.6638 | 0.5000 | 0.9837 | 0.875 | 1 |
| 65 | 131 | AGCTTCTGCTAATCTTGCT | AGCAAGATTAGCAGAAGCT | 0.1466 | 0.0174 | 0.5 | 0.5000 | 0.9402 | 0.625 | 1 |
| 66 | 109 | TAATTAGAGCTGCAGAAAT | ATTTCTGCAGCTCTAATTA | 0.0432 | 0.0034 | 0.5259 | 0.5000 | 1.0000 | 1 | 1 |
| 66 | 177 | GGACAATCAAAAAGAGTTG | CAACTCTTTTTGATTGTCC | 0.0370 | 0.0022 | 0.7241 | 0.7500 | 1.0000 | 1 | 1 |
| 66 | 47 | GCAAATTGATAGGTTGATC | GATCAACCTATCAATTTGC | 0.0360 | 0.0180 | 0.6724 | 0.7500 | 1.0000 | 1 | 1 |
| 67 | 140 | TAATCTTGCTGCTACTAAA | TTTAGTAGCAGCAAGATTA | 0.3454 | 0.1339 | 0.5345 | 0.5000 | 1.0000 | 1 | 1 |
| 67 | 191 | AGTTGATTTTTGTGGAAAG | CTTTCCACAAAAATCAACT | 0.0733 | 0.0022 | 0.569 | 0.5000 | 1.0000 | 1 | 1 |
| 67 | 43 | AAGTGCAAATTGATAGGTT | AACCTATCAATTTGCACTT | 0.1835 | 0.0267 | 0.4914 | 0.5000 | 1.0000 | 1 | 1 |
| 68 | 85 | TGCAGACATATGTGACTCA | TGAGTCACATATGTCTGCA | 0.0611 | 0.0366 | 0.6293 | 0.5000 | 1.0000 | 1 | 1 |
| 68 | 200 | TTGTGGAAAGGGCTATCAT | ATGATAGCCCTTTCCACAA | 0.0287 | 0.0127 | 0.4914 | 0.5000 | 1.0000 | 1 | 1 |
| 68 | 176 | TGGACAATCAAAAAGAGTT | AACTCTTTTTGATTGTCCA | 0.0550 | 0.0019 | 0.6983 | 0.7500 | 1.0000 | 1 | 1 |
| 69 | 78 | CAAAGTTTGCAGACATATG | CATATGTCTGCAAACTTTG | 0.0753 | 0.0026 | 0.4914 | 0.5000 | 1.0000 | 1 | 1 |
| 69 | 74 | ACTTCAAAGTTTGCAGACA | TGTCTGCAAACTTTGAAGT | 0.0200 | 0.0022 | 0.5 | 0.5000 | 1.0000 | 1 | 1 |
| 70 | 27 | GACAAAGTTGAGGCTGAAG | CTTCAGCCTCAACTTTGTC | 0.0362 | 0.0124 | 0.681 | 0.5000 | 1.0000 | 1 | 1 |
| 70 | 201 | TGTGGAAAGGGCTATCATC | GATGATAGCCCTTTCCACA | 0.0189 | 0.0094 | 0.5259 | 0.5000 | 1.0000 | 1 | 1 |
| 70 | 75 | CTTCAAAGTTTGCAGACAT | ATGTCTGCAAACTTTGAAG | 0.0507 | 0.0025 | 0.4828 | 0.7500 | 1.0000 | 1 | 1 |
| 70 | 129 | AGAGCTTCTGCTAATCTTG | CAAGATTAGCAGAAGCTCT | 0.0940 | 0.0017 | 0.5776 | 0.5000 | 0.9239 | 0.75 | 1 |
| 71 | 93 | TATGTGACTCAACAATTAA | TTAATTGTTGAGTCACATA | 0.6576 | 0.0607 | 0.5086 | 0.5000 | 1.0000 | 1 | 1 |
| 71 | 81 | AGTTTGCAGACATATGTGA | TCACATATGTCTGCAAACT | 0.0656 | 0.0032 | 0.4828 | 0.5000 | 1.0000 | 1 | 1 |
| 71 | 163 | CAGAGTGTGTACTTGGACA | TGTCCAAGTACACACTCTG | 0.0186 | 0.0022 | 0.5776 | 0.5000 | 1.0000 | 1 | 1 |
| 72 | 28 | ACAAAGTTGAGGCTGAAGT | ACTTCAGCCTCAACTTTGT | 0.0436 | 0.0145 | 0.5 | 0.5000 | 1.0000 | 1 | 1 |
| 72 | 173 | ACTTGGACAATCAAAAAGA | TCTTTTTGATTGTCCAAGT | 0.0913 | 0.0013 | 0.4828 | 0.5000 | 1.0000 | 1 | 1 |
| 72 | 73 | GACTTCAAAGTTTGCAGAC | GTCTGCAAACTTTGAAGTC | 0.0130 | 0.0018 | 0.5 | 0.5000 | 1.0000 | 1 | 1 |
| 73 | 148 | CTGCTACTAAAATGTCAGA | TCTGACATTTTAGTAGCAG | 0.0240 | 0.0030 | 0.5603 | 0.5000 | 1.0000 | 1 | 1 |
| 73 | 171 | GTACTTGGACAATCAAAAA | TTTTTGATTGTCCAAGTAC | 0.1653 | 0.0012 | 0.681 | 0.7500 | 1.0000 | 1 | 1 |
| 73 | 154 | CTAAAATGTCAGAGTGTGT | ACACACTCTGACATTTTAG | 0.0127 | 0.0060 | 0.4655 | 0.5000 | 1.0000 | 1 | 1 |
| 74 | 141 | AATCTTGCTGCTACTAAAA | TTTTAGTAGCAGCAAGATT | 0.4744 | 0.1465 | 0.5086 | 0.5000 | 1.0000 | 1 | 1 |
| 74 | 183 | TCAAAAAGAGTTGATTTTT | AAAAATCAACTCTTTTTGA | 0.0123 | 0.0098 | 0.7241 | 0.7500 | 1.0000 | 1 | 1 |
| 74 | 20 | ACGTCTTGACAAAGTTGAG | CTCAACTTTGTCAAGACGT | 0.1933 | 0.0015 | 0.4655 | 0.5000 | 1.0000 | 1 | 1 |
| 74 | 170 | TGTACTTGGACAATCAAAA | TTTTGATTGTCCAAGTACA | 0.0384 | 0.0010 | 0.7069 | 0.7500 | 1.0000 | 1 | 1 |

**List of siRNAs predicted by RNAxs for the ‘conserved region 40’ of the ‘S’ gene**

| **WORST RANK** | **Position** | **Target sequence** | **siRNA sequence** | **Access 8nts** | **Access 16nts** | **Energy A.** | **Sequence A.** | **Self-Folding** | **Free End** |  |
| --- | --- | --- | --- | --- | --- | --- | --- | --- | --- | --- |
|  |  |  |  |  |  |  |  |  |  |  |
| 4 | 116 | GGAGTTAGATAAATATTTT | AAAATATTTATCTAACTCC | 0.6366 | 0.1853 | 0.8707 | 1.0000 | 1.0000 | 1 | 1 |
| 12 | 115 | AGGAGTTAGATAAATATTT | AAATATTTATCTAACTCCT | 0.6461 | 0.0770 | 0.8448 | 0.7500 | 1.0000 | 1 | 1 |
| 15 | 117 | GAGTTAGATAAATATTTTA | TAAAATATTTATCTAACTC | 0.6370 | 0.1989 | 0.7759 | 0.7500 | 1.0000 | 1 | 1 |
| 15 | 118 | AGTTAGATAAATATTTTAA | TTAAAATATTTATCTAACT | 0.6368 | 0.2371 | 0.6724 | 0.7500 | 1.0000 | 1 | 1 |
| 17 | 122 | AGATAAATATTTTAAGAAT | ATTCTTAAAATATTTATCT | 0.2231 | 0.1580 | 0.7155 | 0.7500 | 1.0000 | 1 | 1 |
| 19 | 114 | GAGGAGTTAGATAAATATT | AATATTTATCTAACTCCTC | 0.4449 | 0.0446 | 0.8017 | 0.7500 | 1.0000 | 1 | 1 |
| 20 | 113 | GGAGGAGTTAGATAAATAT | ATATTTATCTAACTCCTCC | 0.4164 | 0.0406 | 0.8276 | 1.0000 | 1.0000 | 1 | 1 |
| 22 | 112 | AGGAGGAGTTAGATAAATA | TATTTATCTAACTCCTCCT | 0.3866 | 0.0388 | 0.8017 | 0.7500 | 1.0000 | 1 | 1 |
| 22 | 83 | TCCTTTGCAACCTGAATTA | TAATTCAGGTTGCAAAGGA | 0.1702 | 0.0951 | 0.8362 | 0.7500 | 1.0000 | 1 | 1 |
| 24 | 62 | TGTCAACAACACAGTTTAT | ATAAACTGTGTTGTTGACA | 0.1220 | 0.0298 | 0.6638 | 0.7500 | 0.9783 | 0.875 | 1 |
| 25 | 110 | CAAGGAGGAGTTAGATAAA | TTTATCTAACTCCTCCTTG | 0.2495 | 0.0258 | 0.6466 | 0.7500 | 1.0000 | 1 | 1 |
| 26 | 164 | AGGTGACATCTCTGGCATT | AATGCCAGAGATGTCACCT | 0.1074 | 0.0551 | 0.8362 | 0.7500 | 1.0000 | 1 | 1 |
| 27 | 166 | GTGACATCTCTGGCATTAA | TTAATGCCAGAGATGTCAC | 0.1065 | 0.0511 | 0.7586 | 0.7500 | 1.0000 | 1 | 1 |
| 27 | 109 | TCAAGGAGGAGTTAGATAA | TTATCTAACTCCTCCTTGA | 0.2022 | 0.0255 | 0.6897 | 0.7500 | 1.0000 | 1 | 1 |
| 28 | 165 | GGTGACATCTCTGGCATTA | TAATGCCAGAGATGTCACC | 0.1052 | 0.0524 | 0.819 | 1.0000 | 1.0000 | 1 | 1 |
| 28 | 111 | AAGGAGGAGTTAGATAAAT | ATTTATCTAACTCCTCCTT | 0.3932 | 0.0257 | 0.6293 | 0.5000 | 1.0000 | 1 | 1 |
| 29 | 74 | AGTTTATGATCCTTTGCAA | TTGCAAAGGATCATAAACT | 0.2515 | 0.0792 | 0.5603 | 0.7500 | 1.0000 | 1 | 1 |
| 30 | 71 | CACAGTTTATGATCCTTTG | CAAAGGATCATAAACTGTG | 0.2600 | 0.0222 | 0.6466 | 0.5000 | 1.0000 | 1 | 1 |
| 31 | 167 | TGACATCTCTGGCATTAAT | ATTAATGCCAGAGATGTCA | 0.1002 | 0.0506 | 0.7155 | 0.7500 | 1.0000 | 1 | 1 |
| 31 | 80 | TGATCCTTTGCAACCTGAA | TTCAGGTTGCAAAGGATCA | 0.2281 | 0.1222 | 0.5517 | 0.7500 | 0.9239 | 0.625 | 1 |
| 31 | 61 | TTGTCAACAACACAGTTTA | TAAACTGTGTTGTTGACAA | 0.1141 | 0.0210 | 0.6121 | 0.5000 | 0.9783 | 0.75 | 1 |
| 32 | 70 | ACACAGTTTATGATCCTTT | AAAGGATCATAAACTGTGT | 0.2370 | 0.0185 | 0.7069 | 0.7500 | 1.0000 | 1 | 1 |
| 32 | 82 | ATCCTTTGCAACCTGAATT | AATTCAGGTTGCAAAGGAT | 0.1892 | 0.1014 | 0.6724 | 0.5000 | 1.0000 | 1 | 1 |
| 32 | 168 | GACATCTCTGGCATTAATG | CATTAATGCCAGAGATGTC | 0.0897 | 0.0390 | 0.6638 | 0.5000 | 1.0000 | 1 | 1 |
| 33 | 63 | GTCAACAACACAGTTTATG | CATAAACTGTGTTGTTGAC | 0.1408 | 0.0364 | 0.6638 | 0.5000 | 0.9783 | 0.875 | 1 |
| 35 | 119 | GTTAGATAAATATTTTAAG | CTTAAAATATTTATCTAAC | 0.3449 | 0.2022 | 0.5086 | 0.5000 | 1.0000 | 1 | 1 |
| 36 | 84 | CCTTTGCAACCTGAATTAG | CTAATTCAGGTTGCAAAGG | 0.0704 | 0.0594 | 0.6724 | 0.7500 | 1.0000 | 1 | 1 |
| 36 | 172 | TCTCTGGCATTAATGCTTC | GAAGCATTAATGCCAGAGA | 0.0809 | 0.0150 | 0.5517 | 0.5000 | 0.9457 | 0.875 | 1 |
| 36 | 60 | ATTGTCAACAACACAGTTT | AAACTGTGTTGTTGACAAT | 0.1027 | 0.0139 | 0.5086 | 0.5000 | 0.9783 | 0.625 | 1 |
| 39 | 121 | TAGATAAATATTTTAAGAA | TTCTTAAAATATTTATCTA | 0.2444 | 0.1686 | 0.5 | 0.5000 | 1.0000 | 1 | 1 |
| 40 | 163 | TAGGTGACATCTCTGGCAT | ATGCCAGAGATGTCACCTA | 0.1039 | 0.0675 | 0.5172 | 0.5000 | 1.0000 | 1 | 1 |
| 40 | 75 | GTTTATGATCCTTTGCAAC | GTTGCAAAGGATCATAAAC | 0.2383 | 0.1777 | 0.5 | 0.5000 | 1.0000 | 1 | 1 |
| 41 | 123 | GATAAATATTTTAAGAATC | GATTCTTAAAATATTTATC | 0.2076 | 0.1892 | 0.5 | 0.5000 | 1.0000 | 1 | 1 |
| 42 | 171 | ATCTCTGGCATTAATGCTT | AAGCATTAATGCCAGAGAT | 0.0410 | 0.0106 | 0.5431 | 0.5000 | 0.9457 | 0.75 | 1 |
| 42 | 104 | CTCATTCAAGGAGGAGTTA | TAACTCCTCCTTGAATGAG | 0.0479 | 0.0067 | 0.7759 | 0.7500 | 1.0000 | 1 | 1 |
| 42 | 136 | AGAATCATACATCACCAGA | TCTGGTGATGTATGATTCT | 0.0322 | 0.0239 | 0.5 | 0.5000 | 1.0000 | 1 | 1 |
| 43 | 105 | TCATTCAAGGAGGAGTTAG | CTAACTCCTCCTTGAATGA | 0.0489 | 0.0102 | 0.5517 | 0.5000 | 1.0000 | 1 | 1 |
| 43 | 90 | CAACCTGAATTAGACTCAT | ATGAGTCTAATTCAGGTTG | 0.0250 | 0.0126 | 0.4914 | 0.7500 | 0.9511 | 0.625 | 1 |
| 44 | 69 | AACACAGTTTATGATCCTT | AAGGATCATAAACTGTGTT | 0.2461 | 0.0169 | 0.5086 | 0.5000 | 1.0000 | 1 | 1 |
| 44 | 57 | GGAATTGTCAACAACACAG | CTGTGTTGTTGACAATTCC | 0.0682 | 0.0032 | 0.5862 | 0.7500 | 0.9837 | 0.625 | 1 |
| 44 | 91 | AACCTGAATTAGACTCATT | AATGAGTCTAATTCAGGTT | 0.0145 | 0.0107 | 0.6379 | 0.5000 | 0.9511 | 0.75 | 1 |
| 44 | 65 | CAACAACACAGTTTATGAT | ATCATAAACTGTGTTGTTG | 0.0704 | 0.0181 | 0.4655 | 0.7500 | 0.9783 | 0.625 | 1 |
| 45 | 108 | TTCAAGGAGGAGTTAGATA | TATCTAACTCCTCCTTGAA | 0.1884 | 0.0251 | 0.6293 | 0.5000 | 1.0000 | 1 | 1 |
| 45 | 137 | GAATCATACATCACCAGAT | ATCTGGTGATGTATGATTC | 0.0132 | 0.0099 | 0.4914 | 0.7500 | 0.9891 | 0.625 | 1 |
| 45 | 56 | AGGAATTGTCAACAACACA | TGTGTTGTTGACAATTCCT | 0.0577 | 0.0013 | 0.6379 | 0.5000 | 0.9946 | 0.625 | 1 |
| 45 | 58 | GAATTGTCAACAACACAGT | ACTGTGTTGTTGACAATTC | 0.0790 | 0.0062 | 0.4655 | 0.5000 | 0.9837 | 0.75 | 1 |

**List of siRNAs predicted by RNAxs for the ‘conserved region 41’ of the ‘S’ gene**

| **WORST RANK** | **Position** | **Target sequence** | **siRNA sequence** | **Access 8nts** | **Access 16nts** | **Energy A.** | **Sequence A.** | **Self-Folding** | **Free End** |  |
| --- | --- | --- | --- | --- | --- | --- | --- | --- | --- | --- |
|  |  |  |  |  |  |  |  |  |  |  |
| 5 | 56 | GGTTGCCAAGAATTTAAAT | ATTTAAATTCTTGGCAACC | 0.4722 | 0.1310 | 0.8448 | 1.0000 | 1.0000 | 1 | 1 |
| 9 | 60 | GCCAAGAATTTAAATGAAT | ATTCATTTAAATTCTTGGC | 0.4462 | 0.1935 | 0.9914 | 1.0000 | 1.0000 | 1 | 1 |
| 14 | 21 | GTAAACATTCAAAAAGAAA | TTTCTTTTTGAATGTTTAC | 0.3119 | 0.1597 | 0.681 | 0.7500 | 1.0000 | 1 | 1 |
| 15 | 55 | AGGTTGCCAAGAATTTAAA | TTTAAATTCTTGGCAACCT | 0.4584 | 0.0517 | 0.8448 | 0.7500 | 1.0000 | 1 | 1 |
| 21 | 61 | CCAAGAATTTAAATGAATC | GATTCATTTAAATTCTTGG | 0.1794 | 0.0740 | 0.6638 | 0.7500 | 1.0000 | 1 | 1 |
| 24 | 59 | TGCCAAGAATTTAAATGAA | TTCATTTAAATTCTTGGCA | 0.4149 | 0.1769 | 0.681 | 0.7500 | 1.0000 | 1 | 1 |
| 24 | 107 | GTATGAGCAGTATATAAAA | TTTTATATACTGCTCATAC | 0.3272 | 0.0116 | 0.681 | 0.7500 | 1.0000 | 1 | 1 |
| 25 | 250 | GCTGCAAATTTGATGAAGA | TCTTCATCAAATTTGCAGC | 0.9236 | 0.0322 | 0.6293 | 0.7500 | 1.0000 | 1 | 1 |
| 25 | 242 | TGGATCCTGCTGCAAATTT | AAATTTGCAGCAGGATCCA | 0.1182 | 0.0753 | 0.8448 | 0.7500 | 1.0000 | 1 | 1 |
| 26 | 54 | GAGGTTGCCAAGAATTTAA | TTAAATTCTTGGCAACCTC | 0.4966 | 0.0187 | 0.7759 | 0.7500 | 1.0000 | 1 | 1 |
| 26 | 106 | AGTATGAGCAGTATATAAA | TTTATATACTGCTCATACT | 0.3210 | 0.0113 | 0.7069 | 0.7500 | 1.0000 | 1 | 1 |
| 26 | 241 | GTGGATCCTGCTGCAAATT | AATTTGCAGCAGGATCCAC | 0.1082 | 0.0657 | 0.7845 | 0.7500 | 1.0000 | 1 | 1 |
| 28 | 103 | GAAAGTATGAGCAGTATAT | ATATACTGCTCATACTTTC | 0.1071 | 0.0104 | 0.6293 | 0.7500 | 1.0000 | 1 | 1 |
| 28 | 243 | GGATCCTGCTGCAAATTTG | CAAATTTGCAGCAGGATCC | 0.0904 | 0.0503 | 0.7241 | 0.7500 | 1.0000 | 1 | 1 |
| 31 | 53 | TGAGGTTGCCAAGAATTTA | TAAATTCTTGGCAACCTCA | 0.3741 | 0.0079 | 0.6897 | 0.7500 | 1.0000 | 1 | 1 |
| 32 | 240 | TGTGGATCCTGCTGCAAAT | ATTTGCAGCAGGATCCACA | 0.0704 | 0.0548 | 0.6983 | 0.7500 | 1.0000 | 1 | 1 |
| 33 | 20 | TGTAAACATTCAAAAAGAA | TTCTTTTTGAATGTTTACA | 0.4700 | 0.5239 | 0.5345 | 0.7500 | 1.0000 | 1 | 1 |
| 34 | 50 | CAATGAGGTTGCCAAGAAT | ATTCTTGGCAACCTCATTG | 0.2675 | 0.0065 | 0.6379 | 0.7500 | 1.0000 | 1 | 1 |
| 34 | 22 | TAAACATTCAAAAAGAAAT | ATTTCTTTTTGAATGTTTA | 0.1534 | 0.0944 | 0.5259 | 0.5000 | 1.0000 | 1 | 1 |
| 35 | 49 | TCAATGAGGTTGCCAAGAA | TTCTTGGCAACCTCATTGA | 0.2188 | 0.0059 | 0.5517 | 0.7500 | 1.0000 | 1 | 1 |
| 35 | 114 | CAGTATATAAAATGGCCAT | ATGGCCATTTTATATACTG | 0.0582 | 0.0272 | 0.6293 | 0.7500 | 1.0000 | 1 | 1 |
| 37 | 113 | GCAGTATATAAAATGGCCA | TGGCCATTTTATATACTGC | 0.0754 | 0.0213 | 0.5086 | 0.7500 | 1.0000 | 1 | 1 |
| 38 | 108 | TATGAGCAGTATATAAAAT | ATTTTATATACTGCTCATA | 0.2041 | 0.0085 | 0.5345 | 0.5000 | 1.0000 | 1 | 1 |
| 38 | 48 | CTCAATGAGGTTGCCAAGA | TCTTGGCAACCTCATTGAG | 0.1605 | 0.0048 | 0.5862 | 0.5000 | 1.0000 | 1 | 1 |
| 39 | 102 | GGAAAGTATGAGCAGTATA | TATACTGCTCATACTTTCC | 0.0419 | 0.0108 | 0.8276 | 1.0000 | 1.0000 | 1 | 1 |
| 40 | 105 | AAGTATGAGCAGTATATAA | TTATATACTGCTCATACTT | 0.1897 | 0.0100 | 0.6034 | 0.5000 | 1.0000 | 1 | 1 |
| 40 | 51 | AATGAGGTTGCCAAGAATT | AATTCTTGGCAACCTCATT | 0.2842 | 0.0067 | 0.5 | 0.5000 | 1.0000 | 1 | 1 |
| 41 | 101 | TGGAAAGTATGAGCAGTAT | ATACTGCTCATACTTTCCA | 0.0389 | 0.0115 | 0.8017 | 0.7500 | 1.0000 | 1 | 1 |
| 41 | 123 | AAATGGCCATGGTACATTT | AAATGTACCATGGCCATTT | 0.0446 | 0.0026 | 0.5 | 0.5000 | 1.0000 | 1 | 1 |
| 42 | 249 | TGCTGCAAATTTGATGAAG | CTTCATCAAATTTGCAGCA | 0.9330 | 0.0320 | 0.7155 | 0.5000 | 1.0000 | 1 | 1 |
| 42 | 64 | AGAATTTAAATGAATCTCT | AGAGATTCATTTAAATTCT | 0.0334 | 0.0196 | 0.5 | 0.5000 | 1.0000 | 1 | 1 |
| 42 | 172 | TAGTAATGGTGACAATTAT | ATAATTGTCACCATTACTA | 0.0432 | 0.0021 | 0.6293 | 0.5000 | 1.0000 | 1 | 1 |
| 43 | 52 | ATGAGGTTGCCAAGAATTT | AAATTCTTGGCAACCTCAT | 0.3090 | 0.0074 | 0.6552 | 0.5000 | 1.0000 | 1 | 1 |
| 43 | 115 | AGTATATAAAATGGCCATG | CATGGCCATTTTATATACT | 0.0297 | 0.0242 | 0.5517 | 0.5000 | 1.0000 | 1 | 1 |
| 43 | 23 | AAACATTCAAAAAGAAATT | AATTTCTTTTTGAATGTTT | 0.0705 | 0.0513 | 0.4914 | 0.5000 | 1.0000 | 1 | 1 |
| 44 | 83 | CATCGATCTCCAAGAACTT | AAGTTCTTGGAGATCGATG | 0.0701 | 0.0017 | 0.5172 | 0.7500 | 1.0000 | 1 | 1 |
| 44 | 239 | TTGTGGATCCTGCTGCAAA | TTTGCAGCAGGATCCACAA | 0.0645 | 0.0504 | 0.6466 | 0.5000 | 1.0000 | 1 | 1 |
| 44 | 122 | AAAATGGCCATGGTACATT | AATGTACCATGGCCATTTT | 0.5102 | 0.0043 | 0.4914 | 0.5000 | 1.0000 | 1 | 1 |
| 45 | 170 | CATAGTAATGGTGACAATT | AATTGTCACCATTACTATG | 0.0190 | 0.0056 | 0.6466 | 0.7500 | 1.0000 | 1 | 1 |
| 45 | 93 | CAAGAACTTGGAAAGTATG | CATACTTTCCAAGTTCTTG | 0.0805 | 0.0145 | 0.4914 | 0.5000 | 1.0000 | 1 | 1 |
| 46 | 171 | ATAGTAATGGTGACAATTA | TAATTGTCACCATTACTAT | 0.0414 | 0.0035 | 0.5086 | 0.5000 | 1.0000 | 1 | 1 |
| 47 | 245 | ATCCTGCTGCAAATTTGAT | ATCAAATTTGCAGCAGGAT | 0.9644 | 0.0054 | 0.5 | 0.5000 | 1.0000 | 1 | 1 |
| 47 | 147 | GGTTTTATAGCTGGCTTGA | TCAAGCCAGCTATAAAACC | 0.0161 | 0.0012 | 0.6293 | 0.7500 | 1.0000 | 1 | 1 |
| 48 | 57 | GTTGCCAAGAATTTAAATG | CATTTAAATTCTTGGCAAC | 0.4689 | 0.1923 | 0.4914 | 0.5000 | 1.0000 | 1 | 1 |
| 48 | 82 | TCATCGATCTCCAAGAACT | AGTTCTTGGAGATCGATGA | 0.0494 | 0.0012 | 0.5172 | 0.5000 | 1.0000 | 1 | 1 |
| 48 | 100 | TTGGAAAGTATGAGCAGTA | TACTGCTCATACTTTCCAA | 0.1466 | 0.0469 | 0.4655 | 0.5000 | 1.0000 | 1 | 1 |
| 49 | 149 | TTTTATAGCTGGCTTGATT | AATCAAGCCAGCTATAAAA | 0.0270 | 0.0016 | 0.4914 | 0.5000 | 1.0000 | 1 | 1 |
| 49 | 148 | GTTTTATAGCTGGCTTGAT | ATCAAGCCAGCTATAAAAC | 0.0161 | 0.0013 | 0.4655 | 0.7500 | 1.0000 | 1 | 1 |
| 49 | 146 | AGGTTTTATAGCTGGCTTG | CAAGCCAGCTATAAAACCT | 0.0176 | 0.0010 | 0.6983 | 0.5000 | 1.0000 | 1 | 1 |
| 49 | 173 | AGTAATGGTGACAATTATG | CATAATTGTCACCATTACT | 0.0144 | 0.0017 | 0.5517 | 0.5000 | 1.0000 | 1 | 1 |
