# Supplementary material for: Design of siRNA molecules for silencing of membrane glycoprotein, nucleocapsid phosphoprotein, and surface glycoprotein genes of SARS-CoV2
Source: J Genet Eng Biotechnol. 2022 Apr 28;20:65. doi: 10.1186/s43141-022-00346-z (PMC9047631; doi:10.1186/s43141-022-00346-z)
Supplement: Supplementary file 7 — Additional file 7: Supplementary Table 7. List of siRNAs predicted by siDirect for various conserved regions of the M gene. [file 43141_2022_346_MOESM7_ESM.docx]

**Supplementary Table 7:** List of siRNAs predicted by siDirect for various conserved regions of the M gene

**List of siRNAs predicted by siDirect for the ‘conserved region 6’ of the M gene**

| **target position** | **target sequence** | **RNA oligo, guide** | **passenger** | **functional siRNA selection** | **seed-duplex stability (Tm), guide** | **passenger** |
| --- | --- | --- | --- | --- | --- | --- |
| 4-26' | TACTTCATTGCTTCTTTCAGACT | UCUGAAAGAAGCAAUGAAGUA | CUUCAUUGCUUCUUUCAGACU | UR | 16.6 | 13.8 |
| 51-73 | GTCATTCAATCCAGAAACTAACA | UUAGUUUCUGGAUUGAAUGAC | CAUUCAAUCCAGAAACUAACA | UA | 14.6 | 7.2 |

**List of siRNAs predicted by siDirect for the ‘conserved region 8’ of the M gene**

| **NIL** |
| --- |
