# Supplementary material for: Design of siRNA molecules for silencing of membrane glycoprotein, nucleocapsid phosphoprotein, and surface glycoprotein genes of SARS-CoV2
Source: J Genet Eng Biotechnol. 2022 Apr 28;20:65. doi: 10.1186/s43141-022-00346-z (PMC9047631; doi:10.1186/s43141-022-00346-z)
Supplement: Supplementary file 8 — Additional file 8: Supplementary Table 8. List of siRNAs predicted by siDirect for various conserved regions of the ‘N’ gene. [file 43141_2022_346_MOESM8_ESM.docx]

**Supplementary Table 8:** List of siRNAs predicted by siDirect for various conserved regions of the ‘N’ gene

**List of siRNAs predicted by siDirect for the ‘conserved region 3’ of the N gene**

| **target position** | **target sequence** | **RNA oligo, guide** | **passenger** | **functional siRNA selection** | **seed-duplex stability (Tm), guide** | **passenger** |
| --- | --- | --- | --- | --- | --- | --- |
| 30-52 | CCCAAGGTTTACCCAATAATACT | UAUUAUUGGGUAAACCUUGGG | CAAGGUUUACCCAAUAAUACU | URA | -1.8 | 17.3 |

**List of siRNAs predicted by siDirect for the ‘conserved region 4’ of the N gene**

| **target position** | **target sequence** | **RNA oligo, guide** | **passenger** | **functional siRNA selection** | **seed-duplex stability (Tm), guide** | **passenger** |
| --- | --- | --- | --- | --- | --- | --- |
| 16-38 | TTCCAATTAACACCAATAGCAGT | UGCUAUUGGUGUUAAUUGGAA | CCAAUUAACACCAAUAGCAGU | UR | 19.7 | -1.4 |
| 92-114 | TGACGGTAAAATGAAAGATCTCA | AGAUCUUUCAUUUUACCGUCA | ACGGUAAAAUGAAAGAUCUCA | A | 14.8 | 20.6 |
| 93-115 | GACGGTAAAATGAAAGATCTCAG | GAGAUCUUUCAUUUUACCGUC | CGGUAAAAUGAAAGAUCUCAG | A | 19.1 | 14 |
| 115-137 | GTCCAAGATGGTATTTCTACTAC | AGUAGAAAUACCAUCUUGGAC | CCAAGAUGGUAUUUCUACUAC | URA | 14.6 | 18.1 |

**List of siRNAs predicted by siDirect for the ‘conserved region 7’ of the N gene**

| **NIL** |
| --- |

**List of siRNAs predicted by siDirect for the ‘conserved region 10’ of the N gene**

| **target position** | **target sequence** | **RNA oligo, guide** | **passenger** | **functional siRNA selection** | **seed-duplex stability (Tm), guide** | **passenger** |
| --- | --- | --- | --- | --- | --- | --- |
| 23-45 | GGCCAAACTGTCACTAAGAAATC | UUUCUUAGUGACAGUUUGGCC | CCAAACUGUCACUAAGAAAUC | URA | 11.7 | 16.7 |
| 24-46 | GCCAAACTGTCACTAAGAAATCT | AUUUCUUAGUGACAGUUUGGC | CAAACUGUCACUAAGAAAUCU | UA | 7.1 | 17.8 |
| 85-107 | TGCCACTAAAGCATACAATGTAA | ACAUUGUAUGCUUUAGUGGCA | CCACUAAAGCAUACAAUGUAA | URA | 13.5 | 11.8 |
| 86-108 | GCCACTAAAGCATACAATGTAAC | UACAUUGUAUGCUUUAGUGGC | CACUAAAGCAUACAAUGUAAC | UA | 19.3 | 9.8 |
| 87-109 | CCACTAAAGCATACAATGTAACA | UUACAUUGUAUGCUUUAGUGG | ACUAAAGCAUACAAUGUAACA | RA | 13.5 | 18.3 |
| 89-111 | ACTAAAGCATACAATGTAACACA | UGUUACAUUGUAUGCUUUAGU | UAAAGCAUACAAUGUAACACA | R | 14.6 | 18.5 |
| 131-153 | CCAGAACAAACCCAAGGAAATTT | AUUUCCUUGGGUUUGUUCUGG | AGAACAAACCCAAGGAAAUUU | RA | 18.7 | 14.9 |
| 132-154 | CAGAACAAACCCAAGGAAATTTT | AAUUUCCUUGGGUUUGUUCUG | GAACAAACCCAAGGAAAUUUU | URA | 18.7 | 13.3 |
| 188-210 | TACAAACATTGGCCGCAAATTGC | AAUUUGCGGCCAAUGUUUGUA | CAAACAUUGGCCGCAAAUUGC | URA | 20.6 | 5.3 |
| 198-220 | GGCCGCAAATTGCACAATTTGCC | CAAAUUGUGCAAUUUGCGGCC | CCGCAAAUUGCACAAUUUGCC | A | 5.3 | 20.6 |
| 199-221 | GCCGCAAATTGCACAATTTGCCC | GCAAAUUGUGCAAUUUGCGGC | CGCAAAUUGCACAAUUUGCCC | A | 4.2 | 14 |

**List of siRNAs predicted by siDirect for the ‘conserved region 11’ of the N gene**

| **target position** | **target sequence** | **RNA oligo, guide** | **passenger** | **functional siRNA selection** | **seed-duplex stability (Tm), guide** | **passenger** |
| --- | --- | --- | --- | --- | --- | --- |
| 25-47 | GCCATCAAATTGGATGACAAAGA | UUUGUCAUCCAAUUUGAUGGC | CAUCAAAUUGGAUGACAAAGA | UA | 20.5 | 7.2 |
| 27-49 | CATCAAATTGGATGACAAAGATC | UCUUUGUCAUCCAAUUUGAUG | UCAAAUUGGAUGACAAAGAUC | R | 19.2 | 4.2 |
| 28-50 | ATCAAATTGGATGACAAAGATCC | AUCUUUGUCAUCCAAUUUGAU | CAAAUUGGAUGACAAAGAUCC | UR | 19.2 | 11.3 |
| 35-57 | TGGATGACAAAGATCCAAATTTC | AAUUUGGAUCUUUGUCAUCCA | GAUGACAAAGAUCCAAAUUUC | UA | 20.1 | 20.5 |
| 36-58 | GGATGACAAAGATCCAAATTTCA | AAAUUUGGAUCUUUGUCAUCC | AUGACAAAGAUCCAAAUUUCA | A | 11.3 | 20.5 |
| 38-60 | ATGACAAAGATCCAAATTTCAAA | UGAAAUUUGGAUCUUUGUCAU | GACAAAGAUCCAAAUUUCAAA | UA | 0.4 | 19.2 |
| 39-61 | TGACAAAGATCCAAATTTCAAAG | UUGAAAUUUGGAUCUUUGUCA | ACAAAGAUCCAAAUUUCAAAG | R | 7.4 | 12 |
| 40-62 | GACAAAGATCCAAATTTCAAAGA | UUUGAAAUUUGGAUCUUUGUC | CAAAGAUCCAAAUUUCAAAGA | URA | 7.4 | 14.8 |
| 48-70 | TCCAAATTTCAAAGATCAAGTCA | ACUUGAUCUUUGAAAUUUGGA | CAAAUUUCAAAGAUCAAGUCA | U | 20.4 | 0.4 |
| 54-76 | TTTCAAAGATCAAGTCATTTTGC | AAAAUGACUUGAUCUUUGAAA | UCAAAGAUCAAGUCAUUUUGC | R | 14.8 | 12 |

**List of siRNAs predicted by siDirect for the ‘conserved region 14’ of the N gene**

| **target position** | **target sequence** | **RNA oligo, guide** | **passenger** | **functional siRNA selection** | **seed-duplex stability (Tm), guide** | **passenger** |
| --- | --- | --- | --- | --- | --- | --- |
| 61-83 | CTGCAGATTTGGATGATTTCTCC | AGAAAUCAUCCAAAUCUGCAG | GCAGAUUUGGAUGAUUUCUCC | UA | 16.3 | 12 |
| 70-92 | TGGATGATTTCTCCAAACAATTG | AUUGUUUGGAGAAAUCAUCCA | GAUGAUUUCUCCAAACAAUUG | UA | 12.2 | 7.2 |
