# Supplementary material for: Design of siRNA molecules for silencing of membrane glycoprotein, nucleocapsid phosphoprotein, and surface glycoprotein genes of SARS-CoV2
Source: J Genet Eng Biotechnol. 2022 Apr 28;20:65. doi: 10.1186/s43141-022-00346-z (PMC9047631; doi:10.1186/s43141-022-00346-z)
Supplement: Supplementary file 9 — Additional file 9: Supplementary Table 9. List of siRNAs predicted by siDirect for various conserved regions of the ‘S’ gene. [file 43141_2022_346_MOESM9_ESM.docx]

**Supplementary Table 9:** List of siRNAs predicted by siDirect for various conserved regions of the ‘S’ gene

**List of siRNAs predicted by siDirect for the ‘conserved region 1’ of the S gene**

| **target position** | **target sequence** | **RNA oligo, guide** | **passenger** | **functional siRNA selection** | **seed-duplex stability (Tm), guide** | **passenger** |
| --- | --- | --- | --- | --- | --- | --- |
| 21-43 | CTGCATACACTAATTCTTTCACA | UGAAAGAAUUAGUGUAUGCAG | GCAUACACUAAUUCUUUCACA | URA | 14.8 | 20.4 |
| 23-45 | GCATACACTAATTCTTTCACACG | UGUGAAAGAAUUAGUGUAUGC | AUACACUAAUUCUUUCACACG | R | 19.2 | 20.2 |
| 50-72 | GTTTATTACCCTGACAAAGTTTT | AACUUUGUCAGGGUAAUAAAC | UUAUUACCCUGACAAAGUUUU | R | 17.8 | 15.3 |
| 58-80 | CCCTGACAAAGTTTTCAGATCCT | GAUCUGAAAACUUUGUCAGGG | CUGACAAAGUUUUCAGAUCCU | A | 20.4 | 20.5 |
| 68-90 | GTTTTCAGATCCTCAGTTTTACA | UAAAACUGAGGAUCUGAAAAC | UUUCAGAUCCUCAGUUUUACA | R | 10.3 | 20.4 |

**List of siRNAs predicted by siDirect for the ‘conserved region 5’ of the S gene**

| **target position** | **target sequence** | **RNA oligo, guide** | **passenger** | **functional siRNA selection** | **seed-duplex stability (Tm), guide** | **passenger** |
| --- | --- | --- | --- | --- | --- | --- |
| 23-45 | GTTAATAACGCTACTAATGTTGT | AACAUUAGUAGCGUUAUUAAC | UAAUAACGCUACUAAUGUUGU | R | 11.6 | 6.9 |
| 30-52 | ACGCTACTAATGTTGTTATTAAA | UAAUAACAACAUUAGUAGCGU | GCUACUAAUGUUGUUAUUAAA | UA | 6.9 | 11.3 |
| 31-53 | CGCTACTAATGTTGTTATTAAAG | UUAAUAACAACAUUAGUAGCG | CUACUAAUGUUGUUAUUAAAG | URA | 1.4 | 6.3 |
| 32-54 | GCTACTAATGTTGTTATTAAAGT | UUUAAUAACAACAUUAGUAGC | UACUAAUGUUGUUAUUAAAGU | R | -7.5 | 11.6 |
| 34-56 | TACTAATGTTGTTATTAAAGTCT | ACUUUAAUAACAACAUUAGUA | CUAAUGUUGUUAUUAAAGUCU | UR | -4.3 | 6.9 |
| 46-68 | TATTAAAGTCTGTGAATTTCAAT | UGAAAUUCACAGACUUUAAUA | UUAAAGUCUGUGAAUUUCAAU | R | 10.4 | 14.6 |
| 47-69 | ATTAAAGTCTGTGAATTTCAATT | UUGAAAUUCACAGACUUUAAU | UAAAGUCUGUGAAUUUCAAUU | R | 7.4 | 17.7 |
| 53-75 | GTCTGTGAATTTCAATTTTGTAA | ACAAAAUUGAAAUUCACAGAC | CUGUGAAUUUCAAUUUUGUAA | UA | -3.3 | 20.5 |
| 55-77 | CTGTGAATTTCAATTTTGTAATG | UUACAAAAUUGAAAUUCACAG | GUGAAUUUCAAUUUUGUAAUG | UA | 7.2 | 7.4 |

**List of siRNAs predicted by siDirect for the ‘conserved region 6’ of the S gene**

| **target position** | **target sequence** | **RNA oligo, guide** | **passenger** | **functional siRNA selection** | **seed-duplex stability (Tm), guide** | **passenger** |
| --- | --- | --- | --- | --- | --- | --- |
| 3-25' | GTTTATTCTAGTGCGAATAATTG | AUUAUUCGCACUAGAAUAAAC | UUAUUCUAGUGCGAAUAAUUG | R | 9 | 8.4 |
| 14-36 | TGCGAATAATTGCACTTTTGAAT | UCAAAAGUGCAAUUAUUCGCA | CGAAUAAUUGCACUUUUGAAU | URA | 10.3 | 1.8 |
| 15-37 | GCGAATAATTGCACTTTTGAATA | UUCAAAAGUGCAAUUAUUCGC | GAAUAAUUGCACUUUUGAAUA | URA | 12.2 | -10.3 |
| 17-39 | GAATAATTGCACTTTTGAATATG | UAUUCAAAAGUGCAAUUAUUC | AUAAUUGCACUUUUGAAUAUG | R | 7.4 | 15.3 |
| 18-40 | AATAATTGCACTTTTGAATATGT | AUAUUCAAAAGUGCAAUUAUU | UAAUUGCACUUUUGAAUAUGU | R | 8.9 | 20 |
| 65-87 | TGAAGGAAAACAGGGTAATTTCA | AAAUUACCCUGUUUUCCUUCA | AAGGAAAACAGGGUAAUUUCA | A | 13.9 | 18.7 |
| 67-89 | AAGGAAAACAGGGTAATTTCAAA | UGAAAUUACCCUGUUUUCCUU | GGAAAACAGGGUAAUUUCAAA | URA | 2.1 | 14.9 |
| 68-90 | AGGAAAACAGGGTAATTTCAAAA | UUGAAAUUACCCUGUUUUCCU | GAAAACAGGGUAAUUUCAAAA | UR | 7.4 | 10.3 |
| 75-97 | CAGGGTAATTTCAAAAATCTTAG | AAGAUUUUUGAAAUUACCCUG | GGGUAAUUUCAAAAAUCUUAG | UA | 5.3 | 13.9 |
| 76-98 | AGGGTAATTTCAAAAATCTTAGG | UAAGAUUUUUGAAAUUACCCU | GGUAAUUUCAAAAAUCUUAGG | UA | 5.3 | -0.3 |
| 95-117 | TAGGGAATTTGTGTTTAAGAATA | UUCUUAAACACAAAUUCCCUA | GGGAAUUUGUGUUUAAGAAUA | URA | 7.1 | 14.2 |
| 96-118 | AGGGAATTTGTGTTTAAGAATAT | AUUCUUAAACACAAAUUCCCU | GGAAUUUGUGUUUAAGAAUAU | UA | 7.1 | 7.4 |
| 97-119 | GGGAATTTGTGTTTAAGAATATT | UAUUCUUAAACACAAAUUCCC | GAAUUUGUGUUUAAGAAUAUU | URA | 6.9 | 5.3 |
| 98-120 | GGAATTTGTGTTTAAGAATATTG | AUAUUCUUAAACACAAAUUCC | AAUUUGUGUUUAAGAAUAUUG | R | 6.9 | 12.1 |
| 108-130 | TTTAAGAATATTGATGGTTATTT | AUAACCAUCAAUAUUCUUAAA | UAAGAAUAUUGAUGGUUAUUU | R | 20 | 6.9 |
| 110-132 | TAAGAATATTGATGGTTATTTTA | AAAUAACCAUCAAUAUUCUUA | AGAAUAUUGAUGGUUAUUUUA | A | 13.9 | 1.8 |
| 111-133 | AAGAATATTGATGGTTATTTTAA | AAAAUAACCAUCAAUAUUCUU | GAAUAUUGAUGGUUAUUUUAA | URA | -0.3 | -1.8 |
| 112-134 | AGAATATTGATGGTTATTTTAAA | UAAAAUAACCAUCAAUAUUCU | AAUAUUGAUGGUUAUUUUAAA | R | -9.7 | 8.7 |
| 113-135 | GAATATTGATGGTTATTTTAAAA | UUAAAAUAACCAUCAAUAUUC | AUAUUGAUGGUUAUUUUAAAA | R | -7.5 | 8.7 |
| 114-136 | AATATTGATGGTTATTTTAAAAT | UUUAAAAUAACCAUCAAUAUU | UAUUGAUGGUUAUUUUAAAAU | R | -9.7 | 13.6 |
| 118-140 | TTGATGGTTATTTTAAAATATAT | AUAUUUUAAAAUAACCAUCAA | GAUGGUUAUUUUAAAAUAUAU | URA | -7.5 | 20 |
| 119-141 | TGATGGTTATTTTAAAATATATT | UAUAUUUUAAAAUAACCAUCA | AUGGUUAUUUUAAAAUAUAUU | R | -10.3 | 20 |
| 120-142 | GATGGTTATTTTAAAATATATTC | AUAUAUUUUAAAAUAACCAUC | UGGUUAUUUUAAAAUAUAUUC | RA | -8 | 13.9 |
| 121-143 | ATGGTTATTTTAAAATATATTCT | AAUAUAUUUUAAAAUAACCAU | GGUUAUUUUAAAAUAUAUUCU | UA | -8.6 | -0.3 |
| 125-147 | TTATTTTAAAATATATTCTAAGC | UUAGAAUAUAUUUUAAAAUAA | AUUUUAAAAUAUAUUCUAAGC | R | 8.4 | -9.1 |

**List of siRNAs predicted by siDirect for the ‘conserved region 9’ of the S gene**

| **target position** | **target sequence** | **RNA oligo, guide** | **passenger** | **functional siRNA selection** | **seed-duplex stability (Tm), guide** | **passenger** |
| --- | --- | --- | --- | --- | --- | --- |
| 58-80 | TAGGACTTTTCTATTAAAATATA | UAUUUUAAUAGAAAAGUCCUA | GGACUUUUCUAUUAAAAUAUA | URA | -9.7 | 13.3 |
| 59-81 | AGGACTTTTCTATTAAAATATAA | AUAUUUUAAUAGAAAAGUCCU | GACUUUUCUAUUAAAAUAUAA | UA | -7.5 | 13.3 |
| 60-82 | GGACTTTTCTATTAAAATATAAT | UAUAUUUUAAUAGAAAAGUCC | ACUUUUCUAUUAAAAUAUAAU | R | -10.3 | 10.3 |
| 61-83 | GACTTTTCTATTAAAATATAATG | UUAUAUUUUAAUAGAAAAGUC | CUUUUCUAUUAAAAUAUAAUG | URA | -8 | 7.1 |
| 66-88 | TTCTATTAAAATATAATGAAAAT | UUUCAUUAUAUUUUAAUAGAA | CUAUUAAAAUAUAAUGAAAAU | URA | 8.9 | -7.5 |
| 68-90 | CTATTAAAATATAATGAAAATGG | AUUUUCAUUAUAUUUUAAUAG | AUUAAAAUAUAAUGAAAAUGG | R | 7.4 | -9.7 |
| 71-93 | TTAAAATATAATGAAAATGGAAC | UCCAUUUUCAUUAUAUUUUAA | AAAAUAUAAUGAAAAUGGAAC | R | 11.3 | -8 |
| 80-102 | AATGAAAATGGAACCATTACAGA | UGUAAUGGUUCCAUUUUCAUU | UGAAAAUGGAACCAUUACAGA | R | 20 | 7.4 |

**List of siRNAs predicted by siDirect for the ‘conserved region 10’ of the S gene**

| **target position** | **target sequence** | **RNA oligo, guide** | **passenger** | **functional siRNA selection** | **seed-duplex stability (Tm), guide** | **passenger** |
| --- | --- | --- | --- | --- | --- | --- |
| 8-30' | CAGAAACAAAGTGTACGTTGAAA | UCAACGUACACUUUGUUUCUG | GAAACAAAGUGUACGUUGAAA | URA | 21 | 5.6 |
| 10-32' | GAAACAAAGTGTACGTTGAAATC | UUUCAACGUACACUUUGUUUC | AACAAAGUGUACGUUGAAAUC | A | 21.1 | 17.8 |
| 40-62 | GTAGAAAAAGGAATCTATCAAAC | UUGAUAGAUUCCUUUUUCUAC | AGAAAAAGGAAUCUAUCAAAC | R | 21.4 | 5.5 |
| 41-63 | TAGAAAAAGGAATCTATCAAACT | UUUGAUAGAUUCCUUUUUCUA | GAAAAAGGAAUCUAUCAAACU | URA | 13.4 | 9.5 |
| 44-66 | AAAAAGGAATCTATCAAACTTCT | AAGUUUGAUAGAUUCCUUUUU | AAAGGAAUCUAUCAAACUUCU | R | 19.2 | 18.7 |
| 47-69 | AAGGAATCTATCAAACTTCTAAC | UAGAAGUUUGAUAGAUUCCUU | GGAAUCUAUCAAACUUCUAAC | URA | 17.7 | 16 |
| 48-70 | AGGAATCTATCAAACTTCTAACT | UUAGAAGUUUGAUAGAUUCCU | GAAUCUAUCAAACUUCUAACU | URA | 18.9 | 6.6 |
| 52-74 | ATCTATCAAACTTCTAACTTTAG | AAAGUUAGAAGUUUGAUAGAU | CUAUCAAACUUCUAACUUUAG | URA | 9.8 | 8.9 |
| 53-75 | TCTATCAAACTTCTAACTTTAGA | UAAAGUUAGAAGUUUGAUAGA | UAUCAAACUUCUAACUUUAGA | R | 4.9 | 14.8 |
| 55-77 | TATCAAACTTCTAACTTTAGAGT | UCUAAAGUUAGAAGUUUGAUA | UCAAACUUCUAACUUUAGAGU | R | 9.8 | 10.3 |
| 82-104 | CCAACAGAATCTATTGTTAGATT | UCUAACAAUAGAUUCUGUUGG | AACAGAAUCUAUUGUUAGAUU | R | 11.8 | 19.2 |
| 84-106 | AACAGAATCTATTGTTAGATTTC | AAUCUAACAAUAGAUUCUGUU | CAGAAUCUAUUGUUAGAUUUC | UA | 14.5 | 19.1 |
| 85-107 | ACAGAATCTATTGTTAGATTTCC | AAAUCUAACAAUAGAUUCUGU | AGAAUCUAUUGUUAGAUUUCC | R | 6.9 | 16 |
| 90-112 | ATCTATTGTTAGATTTCCTAATA | UUAGGAAAUCUAACAAUAGAU | CUAUUGUUAGAUUUCCUAAUA | UA | 19.9 | 6.9 |
| 92-114 | CTATTGTTAGATTTCCTAATATT | UAUUAGGAAAUCUAACAAUAG | AUUGUUAGAUUUCCUAAUAUU | R | 19.9 | 11.8 |
| 93-115 | TATTGTTAGATTTCCTAATATTA | AUAUUAGGAAAUCUAACAAUA | UUGUUAGAUUUCCUAAUAUUA | R | 12.3 | 20.3 |
| 94-116 | ATTGTTAGATTTCCTAATATTAC | AAUAUUAGGAAAUCUAACAAU | UGUUAGAUUUCCUAAUAUUAC | R | -2.7 | 14.5 |
| 95-117 | TTGTTAGATTTCCTAATATTACA | UAAUAUUAGGAAAUCUAACAA | GUUAGAUUUCCUAAUAUUACA | URA | -8 | 6.9 |
| 97-119 | GTTAGATTTCCTAATATTACAAA | UGUAAUAUUAGGAAAUCUAAC | UAGAUUUCCUAAUAUUACAAA | R | 1.1 | 14.8 |
| 99-121 | TAGATTTCCTAATATTACAAACT | UUUGUAAUAUUAGGAAAUCUA | GAUUUCCUAAUAUUACAAACU | UA | 6.9 | 18.7 |

**List of siRNAs predicted by siDirect for the ‘conserved region 12’ of the S gene**

| **target position** | **target sequence** | **RNA oligo, guide** | **passenger** | **functional siRNA selection** | **seed-duplex stability (Tm), guide** | **passenger** |
| --- | --- | --- | --- | --- | --- | --- |
| 50-72 | GCTGATTATTCTGTCCTATATAA | AUAUAGGACAGAAUAAUCAGC | UGAUUAUUCUGUCCUAUAUAA | R | 21 | 1.8 |
| 51-73 | CTGATTATTCTGTCCTATATAAT | UAUAUAGGACAGAAUAAUCAG | GAUUAUUCUGUCCUAUAUAAU | URA | 12.2 | 1.8 |
| 52-74 | TGATTATTCTGTCCTATATAATT | UUAUAUAGGACAGAAUAAUCA | AUUAUUCUGUCCUAUAUAAUU | R | -0.8 | 6.9 |
| 53-75 | GATTATTCTGTCCTATATAATTC | AUUAUAUAGGACAGAAUAAUC | UUAUUCUGUCCUAUAUAAUUC | R | -5.9 | 13.4 |
| 75-97 | CCGCATCATTTTCCACTTTTAAG | UAAAAGUGGAAAAUGAUGCGG | GCAUCAUUUUCCACUUUUAAG | URA | 10.3 | 13.6 |
| 76-98 | CGCATCATTTTCCACTTTTAAGT | UUAAAAGUGGAAAAUGAUGCG | CAUCAUUUUCCACUUUUAAGU | URA | 4.9 | 7.2 |
| 86-108 | TCCACTTTTAAGTGTTATGGAGT | UCCAUAACACUUAAAAGUGGA | CACUUUUAAGUGUUAUGGAGU | U | 20 | 4.9 |

**List of siRNAs predicted by siDirect for the ‘conserved region 13’ of the S gene**

| **target position** | **target sequence** | **RNA oligo, guide** | **passenger** | **functional siRNA selection** | **seed-duplex stability (Tm), guide** | **passenger** |
| --- | --- | --- | --- | --- | --- | --- |
| 3-25' | TCCTACTAAATTAAATGATCTCT | AGAUCAUUUAAUUUAGUAGGA | CUACUAAAUUAAAUGAUCUCU | UA | 16.2 | 6.6 |
| 14-36 | TAAATGATCTCTGCTTTACTAAT | UAGUAAAGCAGAGAUCAUUUA | AAUGAUCUCUGCUUUACUAAU | R | 9.8 | 20.4 |
| 22-44 | CTCTGCTTTACTAATGTCTATGC | AUAGACAUUAGUAAAGCAGAG | CUGCUUUACUAAUGUCUAUGC | UA | 20.3 | 19.7 |
| 31-53 | ACTAATGTCTATGCAGATTCATT | UGAAUCUGCAUAGACAUUAGU | UAAUGUCUAUGCAGAUUCAUU | R | 20.4 | 19.2 |
| 41-63 | ATGCAGATTCATTTGTAATTAGA | UAAUUACAAAUGAAUCUGCAU | GCAGAUUCAUUUGUAAUUAGA | URA | 6.9 | 20.4 |
| 43-65 | GCAGATTCATTTGTAATTAGAGG | UCUAAUUACAAAUGAAUCUGC | AGAUUCAUUUGUAAUUAGAGG | R | -2.3 | 16.2 |

**List of siRNAs predicted by siDirect for the ‘conserved region 14’ of the S gene**

| **target position** | **target sequence** | **RNA oligo, guide** | **passenger** | **functional siRNA selection** | **seed-duplex stability (Tm), guide** | **passenger** |
| --- | --- | --- | --- | --- | --- | --- |
| 2-24' | TTGCTGATTATAATTATAAATTA | AUUUAUAAUUAUAAUCAGCAA | GCUGAUUAUAAUUAUAAAUUA | UA | -7.5 | 13.4 |
| 3-25' | TGCTGATTATAATTATAAATTAC | AAUUUAUAAUUAUAAUCAGCA | CUGAUUAUAAUUAUAAAUUAC | UA | -8 | 8.7 |
| 4-26' | GCTGATTATAATTATAAATTACC | UAAUUUAUAAUUAUAAUCAGC | UGAUUAUAAUUAUAAAUUACC | R | -10.3 | 3.5 |
| 7-29' | GATTATAATTATAAATTACCAGA | UGGUAAUUUAUAAUUAUAAUC | UUAUAAUUAUAAAUUACCAGA | R | 13.9 | -8 |
| 16-38 | TATAAATTACCAGATGATTTTAC | AAAAUCAUCUGGUAAUUUAUA | UAAAUUACCAGAUGAUUUUAC | R | 7.2 | -0.3 |
| 17-39 | ATAAATTACCAGATGATTTTACA | UAAAAUCAUCUGGUAAUUUAU | AAAUUACCAGAUGAUUUUACA | R | 7.4 | 13.9 |
| 42-64 | CTGCGTTATAGCTTGGAATTCTA | GAAUUCCAAGCUAUAACGCAG | GCGUUAUAGCUUGGAAUUCUA | A | 20.1 | 8.5 |
| 44-66 | GCGTTATAGCTTGGAATTCTAAC | UAGAAUUCCAAGCUAUAACGC | GUUAUAGCUUGGAAUUCUAAC | URA | 14.8 | 14.9 |
| 45-67 | CGTTATAGCTTGGAATTCTAACA | UUAGAAUUCCAAGCUAUAACG | UUAUAGCUUGGAAUUCUAACA | R | 6.9 | 19.5 |
| 51-73 | AGCTTGGAATTCTAACAATCTTG | AGAUUGUUAGAAUUCCAAGCU | CUUGGAAUUCUAACAAUCUUG | UA | 14.8 | 20.1 |
| 54-76 | TTGGAATTCTAACAATCTTGATT | UCAAGAUUGUUAGAAUUCCAA | GGAAUUCUAACAAUCUUGAUU | UA | 12 | 14.8 |
| 55-77 | TGGAATTCTAACAATCTTGATTC | AUCAAGAUUGUUAGAAUUCCA | GAAUUCUAACAAUCUUGAUUC | U | 20.4 | 6.9 |
| 60-82 | TTCTAACAATCTTGATTCTAAGG | UUAGAAUCAAGAUUGUUAGAA | CUAACAAUCUUGAUUCUAAGG | URA | 16 | 6.9 |
| 75-97 | TTCTAAGGTTGGTGGTAATTATA | UAAUUACCACCAACCUUAGAA | CUAAGGUUGGUGGUAAUUAUA | URA | 13.9 | 18.6 |

**List of siRNAs predicted by siDirect for the ‘conserved region 15’ of the S gene**

| **target position** | **target sequence** | **RNA oligo, guide** | **passenger** | **functional siRNA selection** | **seed-duplex stability (Tm), guide** | **passenger** |
| --- | --- | --- | --- | --- | --- | --- |
| 04-26 | TAGATTGTTTAGGAAGTCTAATC | UUAGACUUCCUAAACAAUCUA | GAUUGUUUAGGAAGUCUAAUC | UA | 18.9 | 5.3 |
| 16-38 | GAAGTCTAATCTCAAACCTTTTG | AAAGGUUUGAGAUUAGACUUC | AGUCUAAUCUCAAACCUUUUG | A | 17.3 | 14.5 |
| 17-39 | AAGTCTAATCTCAAACCTTTTGA | AAAAGGUUUGAGAUUAGACUU | GUCUAAUCUCAAACCUUUUGA | UA | 17.3 | 16 |
| 19-41 | GTCTAATCTCAAACCTTTTGAGA | UCAAAAGGUUUGAGAUUAGAC | CUAAUCUCAAACCUUUUGAGA | UR | 16.1 | 16 |
| 29-51 | AAACCTTTTGAGAGAGATATTTC | AAUAUCUCUCUCAAAAGGUUU | ACCUUUUGAGAGAGAUAUUUC | A | 15.9 | 16.1 |
| 30-52 | AACCTTTTGAGAGAGATATTTCA | AAAUAUCUCUCUCAAAAGGUU | CCUUUUGAGAGAGAUAUUUCA | UA | 6.6 | 12.2 |
| 39-61 | AGAGAGATATTTCAACTGAAATC | UUUCAGUUGAAAUAUCUCUCU | AGAGAUAUUUCAACUGAAAUC | R | 19.2 | 15.9 |
| 40-62 | GAGAGATATTTCAACTGAAATCT | AUUUCAGUUGAAAUAUCUCUC | GAGAUAUUUCAACUGAAAUCU | UA | 19.2 | 6.6 |
| 42-64 | GAGATATTTCAACTGAAATCTAT | AGAUUUCAGUUGAAAUAUCUC | GAUAUUUCAACUGAAAUCUAU | U | 16.3 | 1.8 |
| 43-65 | AGATATTTCAACTGAAATCTATC | UAGAUUUCAGUUGAAAUAUCU | AUAUUUCAACUGAAAUCUAUC | R | 14.8 | 8.9 |

**List of siRNAs predicted by siDirect for the ‘conserved region 19’ of the S gene**

| **target position** | **target sequence** | **RNA oligo, guide** | **passenger** | **functional siRNA selection** | **seed-duplex stability (Tm), guide** | **passenger** |
| --- | --- | --- | --- | --- | --- | --- |
| 28-50 | CAACTGTTTGTGGACCTAAAAAG | UUUUAGGUCCACAAACAGUUG | ACUGUUUGUGGACCUAAAAAG | R | 18.6 | 16.7 |
| 29-51 | AACTGTTTGTGGACCTAAAAAGT | UUUUUAGGUCCACAAACAGUU | CUGUUUGUGGACCUAAAAAGU | URA | 11 | 19.3 |
| 38-60 | TGGACCTAAAAAGTCTACTAATT | UUAGUAGACUUUUUAGGUCCA | GACCUAAAAAGUCUACUAAUU | UA | 20.1 | 18.6 |
| 39-61 | GGACCTAAAAAGTCTACTAATTT | AUUAGUAGACUUUUUAGGUCC | ACCUAAAAAGUCUACUAAUUU | A | 11.3 | 11 |
| 40-62 | GACCTAAAAAGTCTACTAATTTG | AAUUAGUAGACUUUUUAGGUC | CCUAAAAAGUCUACUAAUUUG | URA | 6.3 | -3.8 |
| 41-63 | ACCTAAAAAGTCTACTAATTTGG | AAAUUAGUAGACUUUUUAGGU | CUAAAAAGUCUACUAAUUUGG | URA | 4.6 | -3.8 |
| 48-70 | AAGTCTACTAATTTGGTTAAAAA | UUUAACCAAAUUAGUAGACUU | GUCUACUAAUUUGGUUAAAAA | URA | 20 | 20.1 |
| 49-71 | AGTCTACTAATTTGGTTAAAAAC | UUUUAACCAAAUUAGUAGACU | UCUACUAAUUUGGUUAAAAAC | R | 14 | 11.3 |
| 50-72 | GTCTACTAATTTGGTTAAAAACA | UUUUUAACCAAAUUAGUAGAC | CUACUAAUUUGGUUAAAAACA | URA | 0 | 6.3 |
| 53-75 | TACTAATTTGGTTAAAAACAAAT | UUGUUUUUAACCAAAUUAGUA | CUAAUUUGGUUAAAAACAAAU | UR | 5.6 | -1.4 |
| 54-76 | ACTAATTTGGTTAAAAACAAATG | UUUGUUUUUAACCAAAUUAGU | UAAUUUGGUUAAAAACAAAUG | R | 5.6 | 11.3 |
| 65-87 | TAAAAACAAATGTGTCAATTTCA | AAAUUGACACAUUUGUUUUUA | AAAACAAAUGUGUCAAUUUCA | R | 14.8 | 5.6 |
| 68-90 | AAACAAATGTGTCAATTTCAACT | UUGAAAUUGACACAUUUGUUU | ACAAAUGUGUCAAUUUCAACU | R | 7.4 | 12.1 |
| 74-96 | ATGTGTCAATTTCAACTTCAATG | UUGAAGUUGAAAUUGACACAU | GUGUCAAUUUCAACUUCAAUG | URA | 19.2 | 20.5 |
| 84-106 | TTCAACTTCAATGGTTTAACAGG | UGUUAAACCAUUGAAGUUGAA | CAACUUCAAUGGUUUAACAGG | UR | 8.2 | 19.2 |

**List of siRNAs predicted by siDirect for the ‘conserved region 22’ of the S gene**

| **target position** | **target sequence** | **RNA oligo, guide** | **passenger** | **functional siRNA selection** | **seed-duplex stability (Tm), guide** | **passenger** |
| --- | --- | --- | --- | --- | --- | --- |
| 5-27 | TTGACATTACACCATGTTCTTTT | AAGAACAUGGUGUAAUGUCAA | GACAUUACACCAUGUUCUUUU | UA | 19.2 | 14.6 |
| 6-28 | TGACATTACACCATGTTCTTTTG | AAAGAACAUGGUGUAAUGUCA | ACAUUACACCAUGUUCUUUUG | A | 19.2 | 13.5 |
| 7-29 | GACATTACACCATGTTCTTTTGG | AAAAGAACAUGGUGUAAUGUC | CAUUACACCAUGUUCUUUUGG | UA | 13.3 | 14.6 |

**List of siRNAs predicted by siDirect for the ‘conserved region 23’ of the S gene**

| **target position** | **target sequence** | **RNA oligo, guide** | **passenger** | **functional siRNA selection** | **seed-duplex stability (Tm), guide** | **passenger** |
| --- | --- | --- | --- | --- | --- | --- |
| 48-70 | CTCCTACTTGGCGTGTTTATTCT | AAUAAACACGCCAAGUAGGAG | CCUACUUGGCGUGUUUAUUCU | UA | 6.9 | 16.4 |
| 72-94 | CAGGTTCTAATGTTTTTCAAACA | UUUGAAAAACAUUAGAACCUG | GGUUCUAAUGUUUUUCAAACA | UA | 7.7 | 14.6 |
| 74-96 | GGTTCTAATGTTTTTCAAACACG | UGUUUGAAAAACAUUAGAACC | UUCUAAUGUUUUUCAAACACG | R | 14.9 | 13.4 |
| 123-145 | ATGTCAACAACTCATATGAGTGT | ACUCAUAUGAGUUGUUGACAU | GUCAACAACUCAUAUGAGUGU | UA | 13.3 | 20.5 |
| 161-183 | GCAGGTATATGCGCTAGTTATCA | AUAACUAGCGCAUAUACCUGC | AGGUAUAUGCGCUAGUUAUCA | A | 11.3 | 15.2 |
| 162-184 | CAGGTATATGCGCTAGTTATCAG | GAUAACUAGCGCAUAUACCUG | GGUAUAUGCGCUAGUUAUCAG | A | 6.3 | 8.2 |
| 163-185 | AGGTATATGCGCTAGTTATCAGA | UGAUAACUAGCGCAUAUACCU | GUAUAUGCGCUAGUUAUCAGA | UR | 14.5 | 16.5 |

**List of siRNAs predicted by siDirect for the ‘conserved region 28’ of the S gene**

| **target position** | **target sequence** | **RNA oligo, guide** | **passenger** | **functional siRNA selection** | **seed-duplex stability (Tm), guide** | **passenger** |
| --- | --- | --- | --- | --- | --- | --- |
| 4-26 | TACATTTGTGGTGATTCAACTGA | AGUUGAAUCACCACAAAUGUA | CAUUUGUGGUGAUUCAACUGA | U | 14.8 | 12.1 |
| 20-42 | CAACTGAATGCAGCAATCTTTTG | AAAGAUUGCUGCAUUCAGUUG | ACUGAAUGCAGCAAUCUUUUG | A | 12 | 18.1 |
| 30-52 | CAGCAATCTTTTGTTGCAATATG | UAUUGCAACAAAAGAUUGCUG | GCAAUCUUUUGUUGCAAUAUG | URA | 20 | 12 |
| 31-53 | AGCAATCTTTTGTTGCAATATGG | AUAUUGCAACAAAAGAUUGCU | CAAUCUUUUGUUGCAAUAUGG | UA | 21.1 | 5.3 |
| 43-65 | TTGCAATATGGCAGTTTTTGTAC | ACAAAAACUGCCAUAUUGCAA | GCAAUAUGGCAGUUUUUGUAC | URA | 5.6 | 5.6 |
| 44-66 | TGCAATATGGCAGTTTTTGTACA | UACAAAAACUGCCAUAUUGCA | CAAUAUGGCAGUUUUUGUACA | UR | 5.6 | 12.6 |
| 51-73 | TGGCAGTTTTTGTACACAATTAA | AAUUGUGUACAAAAACUGCCA | GCAGUUUUUGUACACAAUUAA | UA | 19.3 | 10.3 |
| 52-74 | GGCAGTTTTTGTACACAATTAAA | UAAUUGUGUACAAAAACUGCC | CAGUUUUUGUACACAAUUAAA | URA | 12.1 | 3.2 |
| 53-75 | GCAGTTTTTGTACACAATTAAAC | UUAAUUGUGUACAAAAACUGC | AGUUUUUGUACACAAUUAAAC | R | 6.9 | 5.6 |
| 54-76 | CAGTTTTTGTACACAATTAAACC | UUUAAUUGUGUACAAAAACUG | GUUUUUGUACACAAUUAAACC | URA | -1.4 | 5.6 |
| 65-87 | CACAATTAAACCGTGCTTTAACT | UUAAAGCACGGUUUAAUUGUG | CAAUUAAACCGUGCUUUAACU | URA | 19.7 | -9.7 |
| 85-107 | ACTGGAATAGCTGTTGAACAAGA | UUGUUCAACAGCUAUUCCAGU | UGGAAUAGCUGUUGAACAAGA | R | 20.5 | 19.9 |
| 87-109 | TGGAATAGCTGTTGAACAAGACA | UCUUGUUCAACAGCUAUUCCA | GAAUAGCUGUUGAACAAGACA | UR | 19.2 | 18.3 |
| 93-115 | AGCTGTTGAACAAGACAAAAACA | UUUUUGUCUUGUUCAACAGCU | CUGUUGAACAAGACAAAAACA | URA | 14.9 | 20.5 |
| 95-117 | CTGTTGAACAAGACAAAAACACC | UGUUUUUGUCUUGUUCAACAG | GUUGAACAAGACAAAAACACC | UA | 5.6 | 20.5 |
| 106-128 | GACAAAAACACCCAAGAAGTTTT | AACUUCUUGGGUGUUUUUGUC | CAAAAACACCCAAGAAGUUUU | URA | 17.7 | 5.6 |
| 131-153 | CACAAGTCAAACAAATTTACAAA | UGUAAAUUUGUUUGACUUGUG | CAAGUCAAACAAAUUUACAAA | UR | -0.3 | 19.2 |
| 134-156 | AAGTCAAACAAATTTACAAAACA | UUUUGUAAAUUUGUUUGACUU | GUCAAACAAAUUUACAAAACA | UA | 7.2 | 20.5 |
| 136-158 | GTCAAACAAATTTACAAAACACC | UGUUUUGUAAAUUUGUUUGAC | CAAACAAAUUUACAAAACACC | URA | 13.3 | 5.6 |
| 146-168 | TTTACAAAACACCACCAATTAAA | UAAUUGGUGGUGUUUUGUAAA | UACAAAACACCACCAAUUAAA | R | 18.8 | 13.3 |
| 147-169 | TTACAAAACACCACCAATTAAAG | UUAAUUGGUGGUGUUUUGUAA | ACAAAACACCACCAAUUAAAG | RA | 12.8 | 12.2 |
| 148-170 | TACAAAACACCACCAATTAAAGA | UUUAAUUGGUGGUGUUUUGUA | CAAAACACCACCAAUUAAAGA | URA | -1.4 | 13.3 |
| 164-186 | TTAAAGATTTTGGTGGTTTTAAT | UAAAACCACCAAAAUCUUUAA | AAAGAUUUUGGUGGUUUUAAU | R | 18.8 | 5.3 |
| 166-188 | AAAGATTTTGGTGGTTTTAATTT | AUUAAAACCACCAAAAUCUUU | AGAUUUUGGUGGUUUUAAUUU | R | 0 | 7.4 |
| 167-189 | AAGATTTTGGTGGTTTTAATTTT | AAUUAAAACCACCAAAAUCUU | GAUUUUGGUGGUUUUAAUUUU | UA | -9.7 | 11.3 |
| 174-196 | TGGTGGTTTTAATTTTTCACAAA | UGUGAAAAAUUAAAACCACCA | GUGGUUUUAAUUUUUCACAAA | UA | 14.9 | 18.8 |
| 176-198 | GTGGTTTTAATTTTTCACAAATA | UUUGUGAAAAAUUAAAACCAC | GGUUUUAAUUUUUCACAAAUA | URA | 20.5 | 0 |
| 177-199 | TGGTTTTAATTTTTCACAAATAT | AUUUGUGAAAAAUUAAAACCA | GUUUUAAUUUUUCACAAAUAU | UA | 20.5 | -9.7 |
| 178-200 | GGTTTTAATTTTTCACAAATATT | UAUUUGUGAAAAAUUAAAACC | UUUUAAUUUUUCACAAAUAUU | R | 12.1 | -9.7 |
| 179-201 | GTTTTAATTTTTCACAAATATTA | AUAUUUGUGAAAAAUUAAAAC | UUUAAUUUUUCACAAAUAUUA | R | 6.9 | -9.7 |
| 180-202 | TTTTAATTTTTCACAAATATTAC | AAUAUUUGUGAAAAAUUAAAA | UUAAUUUUUCACAAAUAUUAC | R | -1.8 | -9.7 |
| 181-203 | TTTAATTTTTCACAAATATTACC | UAAUAUUUGUGAAAAAUUAAA | UAAUUUUUCACAAAUAUUACC | R | -10.3 | -12 |

**List of siRNAs predicted by siDirect for the ‘conserved region 29’ of the S gene**

| **target position** | **target sequence** | **RNA oligo, guide** | **passenger** | **functional siRNA selection** | **seed-duplex stability (Tm), guide** | **passenger** |
| --- | --- | --- | --- | --- | --- | --- |
| 8-30 | AGGTCATTTATTGAAGATCTACT | UAGAUCUUCAAUAAAUGACCU | GUCAUUUAUUGAAGAUCUACU | URA | 19.1 | 8.9 |
| 16-38 | TATTGAAGATCTACTTTTCAACA | UUGAAAAGUAGAUCUUCAAUA | UUGAAGAUCUACUUUUCAACA | R | 12.2 | 20.4 |
| 18-40 | TTGAAGATCTACTTTTCAACAAA | UGUUGAAAAGUAGAUCUUCAA | GAAGAUCUACUUUUCAACAAA | U | 14.9 | 19.1 |
| 19-41 | TGAAGATCTACTTTTCAACAAAG | UUGUUGAAAAGUAGAUCUUCA | AAGAUCUACUUUUCAACAAAG | R | 20.5 | 20.2 |
| 24-46 | ATCTACTTTTCAACAAAGTGACA | UCACUUUGUUGAAAAGUAGAU | CUACUUUUCAACAAAGUGACA | U | 16.7 | 4.9 |

**List of siRNAs predicted by siDirect for the ‘conserved region 30’ of the S gene**

| **target position** | **target sequence** | **RNA oligo, guide** | **passenger** | **functional siRNA selection** | **seed-duplex stability (Tm), guide** | **passenger** |
| --- | --- | --- | --- | --- | --- | --- |
| 10-32 | ACCTCATTTGTGCACAAAAGTTT | ACUUUUGUGCACAAAUGAGGU | CUCAUUUGUGCACAAAAGUUU | UA | 10.3 | 13.8 |
| 12-34 | CTCATTTGTGCACAAAAGTTTAA | AAACUUUUGUGCACAAAUGAG | CAUUUGUGCACAAAAGUUUAA | UA | 3.2 | 12.1 |
| 27-49 | AAGTTTAACGGCCTTACTGTTTT | AACAGUAAGGCCGUUAAACUU | GUUUAACGGCCUUACUGUUUU | UA | 19 | 7.1 |
| 67-89 | ATGAAATGATTGCTCAATACACT | UGUAUUGAGCAAUCAUUUCAU | GAAAUGAUUGCUCAAUACACU | UR | 16.1 | 7.2 |

**List of siRNAs predicted by siDirect for the ‘conserved region 31’ of the S gene**

| **target position** | **target sequence** | **RNA oligo, guide** | **passenger** | **functional siRNA selection** | **seed-duplex stability (Tm), guide** | **passenger** |
| --- | --- | --- | --- | --- | --- | --- |
| 11-33 | TGCTGCATTACAAATACCATTTG | AAUGGUAUUUGUAAUGCAGCA | CUGCAUUACAAAUACCAUUUG | UA | 20 | 21.1 |
| 32-54 | TGCTATGCAAATGGCTTATAGGT | CUAUAAGCCAUUUGCAUAGCA | CUAUGCAAAUGGCUUAUAGGU | R | 14.9 | 21.1 |
| 36-58 | ATGCAAATGGCTTATAGGTTTAA | AAACCUAUAAGCCAUUUGCAU | GCAAAUGGCUUAUAGGUUUAA | URA | 18.5 | 17.7 |
| 42-64 | ATGGCTTATAGGTTTAATGGTAT | ACCAUUAAACCUAUAAGCCAU | GGCUUAUAGGUUUAAUGGUAU | UA | 12.8 | 14.9 |
| 43-65 | TGGCTTATAGGTTTAATGGTATT | UACCAUUAAACCUAUAAGCCA | GCUUAUAGGUUUAAUGGUAUU | UA | 20 | 2.8 |
| 44-66 | GGCTTATAGGTTTAATGGTATTG | AUACCAUUAAACCUAUAAGCC | CUUAUAGGUUUAAUGGUAUUG | UA | 20 | 12.3 |
| 45-67 | GCTTATAGGTTTAATGGTATTGG | AAUACCAUUAAACCUAUAAGC | UUAUAGGUUUAAUGGUAUUGG | R | 20 | 19.8 |
| 54-76 | TTTAATGGTATTGGAGTTACACA | UGUAACUCCAAUACCAUUAAA | UAAUGGUAUUGGAGUUACACA | R | 21.4 | 20 |
| 65-87 | TGGAGTTACACAGAATGTTCTCT | AGAACAUUCUGUGUAACUCCA | GAGUUACACAGAAUGUUCUCU | UA | 14.8 | 19 |
| 71-93 | TACACAGAATGTTCTCTATGAGA | UCAUAGAGAACAUUCUGUGUA | CACAGAAUGUUCUCUAUGAGA | URA | 17.8 | 19.2 |
| 73-95 | CACAGAATGTTCTCTATGAGAAC | UCUCAUAGAGAACAUUCUGUG | CAGAAUGUUCUCUAUGAGAAC | UA | 17.8 | 19.2 |
| 74-96 | ACAGAATGTTCTCTATGAGAACC | UUCUCAUAGAGAACAUUCUGU | AGAAUGUUCUCUAUGAGAACC | R | 21.4 | 14.8 |
| 81-103 | GTTCTCTATGAGAACCAAAAATT | UUUUUGGUUCUCAUAGAGAAC | UCUCUAUGAGAACCAAAAAUU | R | 18.8 | 17.8 |
| 82-104 | TTCTCTATGAGAACCAAAAATTG | AUUUUUGGUUCUCAUAGAGAA | CUCUAUGAGAACCAAAAAUUG | UA | 11.5 | 21.4 |

**List of siRNAs predicted by siDirect for the ‘conserved region 33’ of the S gene**

| **target position** | **target sequence** | **RNA oligo, guide** | **passenger** | **functional siRNA selection** | **seed-duplex stability (Tm), guide** | **passenger** |
| --- | --- | --- | --- | --- | --- | --- |
| 1-23 | AGCTTTAAACACGCTTGTTAAAC | UUAACAAGCGUGUUUAAAGCU | CUUUAAACACGCUUGUUAAAC | UA | 11.8 | 0 |
| 2-24 | GCTTTAAACACGCTTGTTAAACA | UUUAACAAGCGUGUUUAAAGC | UUUAAACACGCUUGUUAAACA | R | 7.2 | 7.2 |
| 4-26 | TTTAAACACGCTTGTTAAACAAC | UGUUUAACAAGCGUGUUUAAA | UAAACACGCUUGUUAAACAAC | R | 8.2 | 19.8 |
| 20-42 | AAACAACTTAGCTCCAATTTTGG | AAAAUUGGAGCUAAGUUGUUU | ACAACUUAGCUCCAAUUUUGG | R | 11.3 | 11.8 |
| 21-43 | AACAACTTAGCTCCAATTTTGGT | CAAAAUUGGAGCUAAGUUGUU | CAACUUAGCUCCAAUUUUGGU | R | -3.3 | 9.8 |
| 32-54 | TCCAATTTTGGTGCAATTTCAAG | UGAAAUUGCACCAAAAUUGGA | CAAUUUUGGUGCAAUUUCAAG | UR | 7.4 | -3.3 |
| 40-62 | TGGTGCAATTTCAAGTGTTTTAA | AAAACACUUGAAAUUGCACCA | GUGCAAUUUCAAGUGUUUUAA | UA | 17.8 | 20 |
| 41-63 | GGTGCAATTTCAAGTGTTTTAAA | UAAAACACUUGAAAUUGCACC | UGCAAUUUCAAGUGUUUUAAA | R | 13.3 | 14 |
| 42-64 | GTGCAATTTCAAGTGTTTTAAAT | UUAAAACACUUGAAAUUGCAC | GCAAUUUCAAGUGUUUUAAAU | URA | 7.2 | 7.4 |
| 43-65 | TGCAATTTCAAGTGTTTTAAATG | UUUAAAACACUUGAAAUUGCA | CAAUUUCAAGUGUUUUAAAUG | URA | 0 | 7.4 |
| 44-66 | GCAATTTCAAGTGTTTTAAATGA | AUUUAAAACACUUGAAAUUGC | AAUUUCAAGUGUUUUAAAUGA | R | -9.1 | 7.4 |
| 46-68 | AATTTCAAGTGTTTTAAATGATA | UCAUUUAAAACACUUGAAAUU | UUUCAAGUGUUUUAAAUGAUA | R | -1.4 | 19.2 |
| 49-71 | TTCAAGTGTTTTAAATGATATCC | AUAUCAUUUAAAACACUUGAA | CAAGUGUUUUAAAUGAUAUCC | URA | 8.7 | 17.8 |

**List of siRNAs predicted by siDirect for the ‘conserved region 34’ of the S gene**

| **target position** | **target sequence** | **RNA oligo, guide** | **passenger** | **functional siRNA selection** | **seed-duplex stability (Tm), guide** | **passenger** |
| --- | --- | --- | --- | --- | --- | --- |
| 71-93 | GACATATGTGACTCAACAATTAA | AAUUGUUGAGUCACAUAUGUC | CAUAUGUGACUCAACAAUUAA | UA | 12.1 | 13.3 |
| 72-94 | ACATATGTGACTCAACAATTAAT | UAAUUGUUGAGUCACAUAUGU | AUAUGUGACUCAACAAUUAAU | R | 5.3 | 21.5 |
| 120-142 | GCTAATCTTGCTGCTACTAAAAT | UUUAGUAGCAGCAAGAUUAGC | UAAUCUUGCUGCUACUAAAAU | R | 11.3 | 12 |
| 149-171 | GTGTGTACTTGGACAATCAAAAA | UUUGAUUGUCCAAGUACACAC | GUGUACUUGGACAAUCAAAAA | URA | 13.8 | 19 |
| 160-182 | GACAATCAAAAAGAGTTGATTTT | AAUCAACUCUUUUUGAUUGUC | CAAUCAAAAAGAGUUGAUUUU | UA | 19.2 | 7.4 |
| 162-184 | CAATCAAAAAGAGTTGATTTTTG | AAAAUCAACUCUUUUUGAUUG | AUCAAAAAGAGUUGAUUUUUG | A | 7.4 | 7.7 |
| 165-187 | TCAAAAAGAGTTGATTTTTGTGG | ACAAAAAUCAACUCUUUUUGA | AAAAAGAGUUGAUUUUUGUGG | R | -3.3 | 10.3 |

**List of siRNAs predicted by siDirect for the ‘conserved region 40’ of the S gene**

| **target position** | **target sequence** | **RNA oligo, guide** | **passenger** | **functional siRNA selection** | **seed-duplex stability (Tm), guide** | **passenger** |
| --- | --- | --- | --- | --- | --- | --- |
| 16-38 | CTGGTAACTGTGATGTTGTAATA | UUACAACAUCACAGUUACCAG | GGUAACUGUGAUGUUGUAAUA | UA | 20.4 | 19 |
| 17-39 | TGGTAACTGTGATGTTGTAATAG | AUUACAACAUCACAGUUACCA | GUAACUGUGAUGUUGUAAUAG | UA | 14.7 | 19 |
| 23-45 | CTGTGATGTTGTAATAGGAATTG | AUUCCUAUUACAACAUCACAG | GUGAUGUUGUAAUAGGAAUUG | UA | 19.9 | 20.5 |
| 28-50 | ATGTTGTAATAGGAATTGTCAAC | UGACAAUUCCUAUUACAACAU | GUUGUAAUAGGAAUUGUCAAC | UA | 14.8 | 6.9 |
| 44-66 | TGTCAACAACACAGTTTATGATC | UCAUAAACUGUGUUGUUGACA | UCAACAACACAGUUUAUGAUC | R | 6.9 | 19.3 |
| 45-67 | GTCAACAACACAGTTTATGATCC | AUCAUAAACUGUGUUGUUGAC | CAACAACACAGUUUAUGAUCC | UA | 8.9 | 19.3 |
| 65-87 | TCCTTTGCAACCTGAATTAGACT | UCUAAUUCAGGUUGCAAAGGA | CUUUGCAACCUGAAUUAGACU | UR | 6.9 | 20 |
| 73-95 | AACCTGAATTAGACTCATTCAAG | UGAAUGAGUCUAAUUCAGGUU | CCUGAAUUAGACUCAUUCAAG | URA | 20.4 | 12 |
| 86-108 | CTCATTCAAGGAGGAGTTAGATA | UCUAACUCCUCCUUGAAUGAG | CAUUCAAGGAGGAGUUAGAUA | URA | 18.9 | 12 |
| 96-118 | GAGGAGTTAGATAAATATTTTAA | AAAAUAUUUAUCUAACUCCUC | GGAGUUAGAUAAAUAUUUUAA | URA | -10.3 | 18.9 |
| 97-119 | AGGAGTTAGATAAATATTTTAAG | UAAAAUAUUUAUCUAACUCCU | GAGUUAGAUAAAUAUUUUAAG | URA | -10.3 | 18.9 |
| 98-120 | GGAGTTAGATAAATATTTTAAGA | UUAAAAUAUUUAUCUAACUCC | AGUUAGAUAAAUAUUUUAAGA | R | -7.5 | 14.5 |
| 100-122 | AGTTAGATAAATATTTTAAGAAT | UCUUAAAAUAUUUAUCUAACU | UUAGAUAAAUAUUUUAAGAAU | R | -3.8 | 8.4 |
| 102-124 | TTAGATAAATATTTTAAGAATCA | AUUCUUAAAAUAUUUAUCUAA | AGAUAAAUAUUUUAAGAAUCA | RA | 7.1 | 1.8 |
| 104-126 | AGATAAATATTTTAAGAATCATA | UGAUUCUUAAAAUAUUUAUCU | AUAAAUAUUUUAAGAAUCAUA | R | 14.8 | -8 |
| 106-128 | ATAAATATTTTAAGAATCATACA | UAUGAUUCUUAAAAUAUUUAU | AAAUAUUUUAAGAAUCAUACA | R | 16.2 | -10.3 |
| 108-130 | AAATATTTTAAGAATCATACATC | UGUAUGAUUCUUAAAAUAUUU | AUAUUUUAAGAAUCAUACAUC | R | 16 | -7.5 |
| 122-144 | TCATACATCACCAGATGTTGATT | UCAACAUCUGGUGAUGUAUGA | AUACAUCACCAGAUGUUGAUU | R | 20.5 | 21.5 |

**List of siRNAs predicted by siDirect for the ‘conserved region 41’ of the S gene**

| **target position** | **target sequence** | **RNA oligo, guide** | **passenger** | **functional siRNA selection** | **seed-duplex stability (Tm), guide** | **passenger** |
| --- | --- | --- | --- | --- | --- | --- |
| 1-23 | TTGTAAACATTCAAAAAGAAATT | UUUCUUUUUGAAUGUUUACAA | GUAAACAUUCAAAAAGAAAUU | URA | 5.5 | 6.9 |
| 3-25 | GTAAACATTCAAAAAGAAATTGA | AAUUUCUUUUUGAAUGUUUAC | AAACAUUCAAAAAGAAAUUGA | R | 5.3 | 14.8 |
| 5-27 | AAACATTCAAAAAGAAATTGACC | UCAAUUUCUUUUUGAAUGUUU | ACAUUCAAAAAGAAAUUGACC | R | 7.4 | 13.8 |
| 17-39 | AGAAATTGACCGCCTCAATGAGG | UCAUUGAGGCGGUCAAUUUCU | AAAUUGACCGCCUCAAUGAGG | R | 18.1 | 14.8 |
| 41-63 | TGCCAAGAATTTAAATGAATCTC | GAUUCAUUUAAAUUCUUGGCA | CCAAGAAUUUAAAUGAAUCUC | A | 7.2 | 12 |
| 42-64 | GCCAAGAATTTAAATGAATCTCT | AGAUUCAUUUAAAUUCUUGGC | CAAGAAUUUAAAUGAAUCUCU | UA | 16.2 | 5.3 |
| 72-94 | CTCCAAGAACTTGGAAAGTATGA | AUACUUUCCAAGUUCUUGGAG | CCAAGAACUUGGAAAGUAUGA | URA | 14.6 | 19.2 |
| 83-105 | TGGAAAGTATGAGCAGTATATAA | AUAUACUGCUCAUACUUUCCA | GAAAGUAUGAGCAGUAUAUAA | URA | 13.1 | 4.6 |
| 84-106 | GGAAAGTATGAGCAGTATATAAA | UAUAUACUGCUCAUACUUUCC | AAAGUAUGAGCAGUAUAUAAA | R | 6.1 | 11.6 |
| 85-107 | GAAAGTATGAGCAGTATATAAAA | UUAUAUACUGCUCAUACUUUC | AAGUAUGAGCAGUAUAUAAAA | R | 2.8 | 20.3 |
| 86-108 | AAAGTATGAGCAGTATATAAAAT | UUUAUAUACUGCUCAUACUUU | AGUAUGAGCAGUAUAUAAAAU | R | -5.9 | 20.3 |
| 114-136 | TGGTACATTTGGCTAGGTTTTAT | AAAACCUAGCCAAAUGUACCA | GUACAUUUGGCUAGGUUUUAU | URA | 18.6 | 6.9 |
| 116-138 | GTACATTTGGCTAGGTTTTATAG | AUAAAACCUAGCCAAAUGUAC | ACAUUUGGCUAGGUUUUAUAG | R | 14 | 17.7 |
| 140-162 | TGGCTTGATTGCCATAGTAATGG | AUUACUAUGGCAAUCAAGCCA | GCUUGAUUGCCAUAGUAAUGG | UA | 6.3 | 12 |
| 150-172 | GCCATAGTAATGGTGACAATTAT | AAUUGUCACCAUUACUAUGGC | CAUAGUAAUGGUGACAAUUAU | UA | 20.5 | 6.3 |
| 151-173 | CCATAGTAATGGTGACAATTATG | UAAUUGUCACCAUUACUAUGG | AUAGUAAUGGUGACAAUUAUG | R | 14.8 | 6.3 |
| 152-174 | CATAGTAATGGTGACAATTATGC | AUAAUUGUCACCAUUACUAUG | UAGUAAUGGUGACAAUUAUGC | R | 6.9 | 11.6 |
| 166-188 | CAATTATGCTTTGCTGTATGACC | UCAUACAGCAAAGCAUAAUUG | AUUAUGCUUUGCUGUAUGACC | R | 17.9 | 19.7 |
