# Supplementary material for: Design of siRNA molecules for silencing of membrane glycoprotein, nucleocapsid phosphoprotein, and surface glycoprotein genes of SARS-CoV2
Source: J Genet Eng Biotechnol. 2022 Apr 28;20:65. doi: 10.1186/s43141-022-00346-z (PMC9047631; doi:10.1186/s43141-022-00346-z)

**Supplementary Table 13: List of siRNAs predicted by OligoWalk for various conserved regions of the ‘M’ gene**

List of siRNAs predicted by OligoWalk for the ‘conserved region 6’ of the M gene


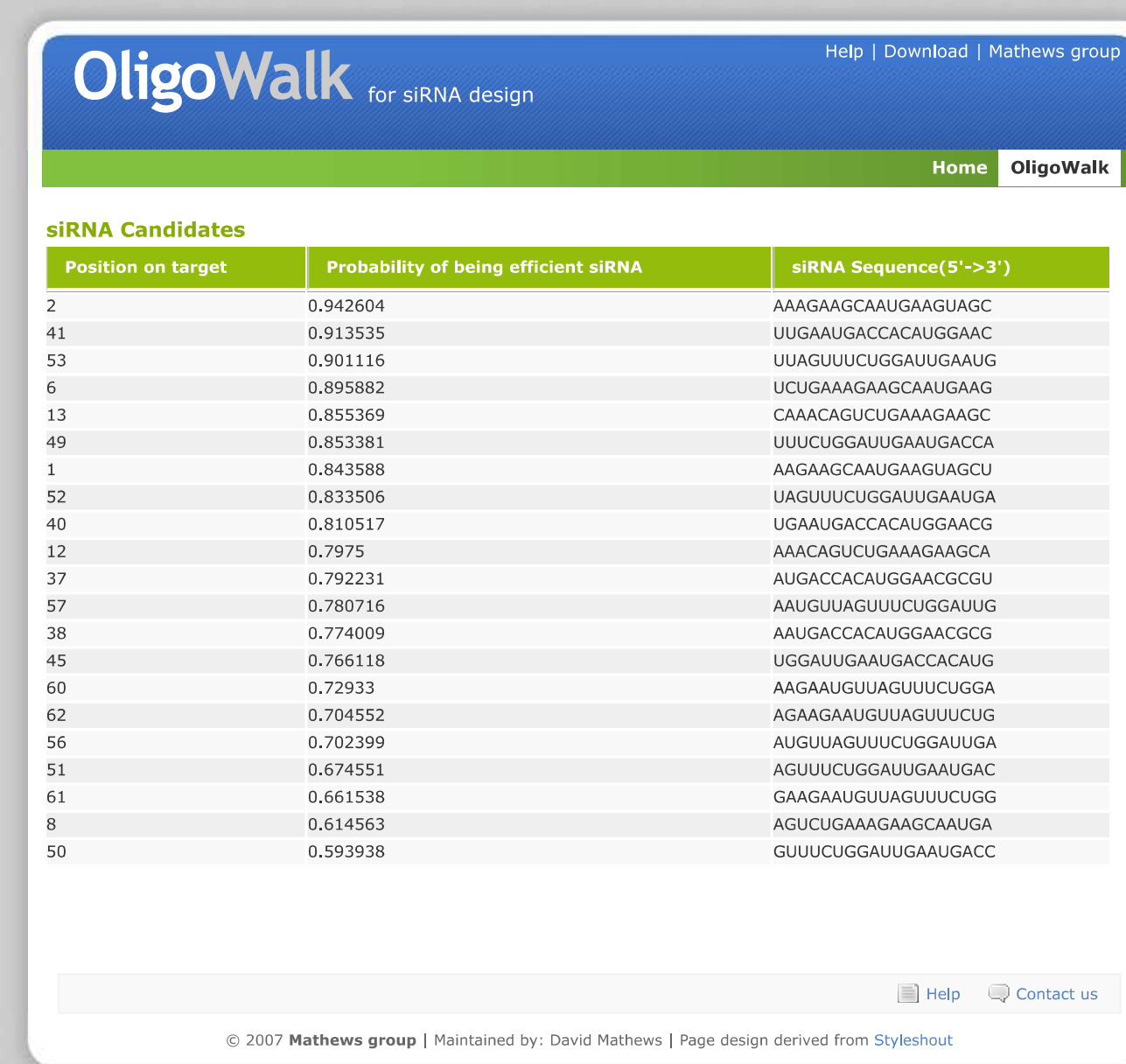


List of siRNAs predicted by OligoWalk for the ‘conserved region 8’ of the M gene


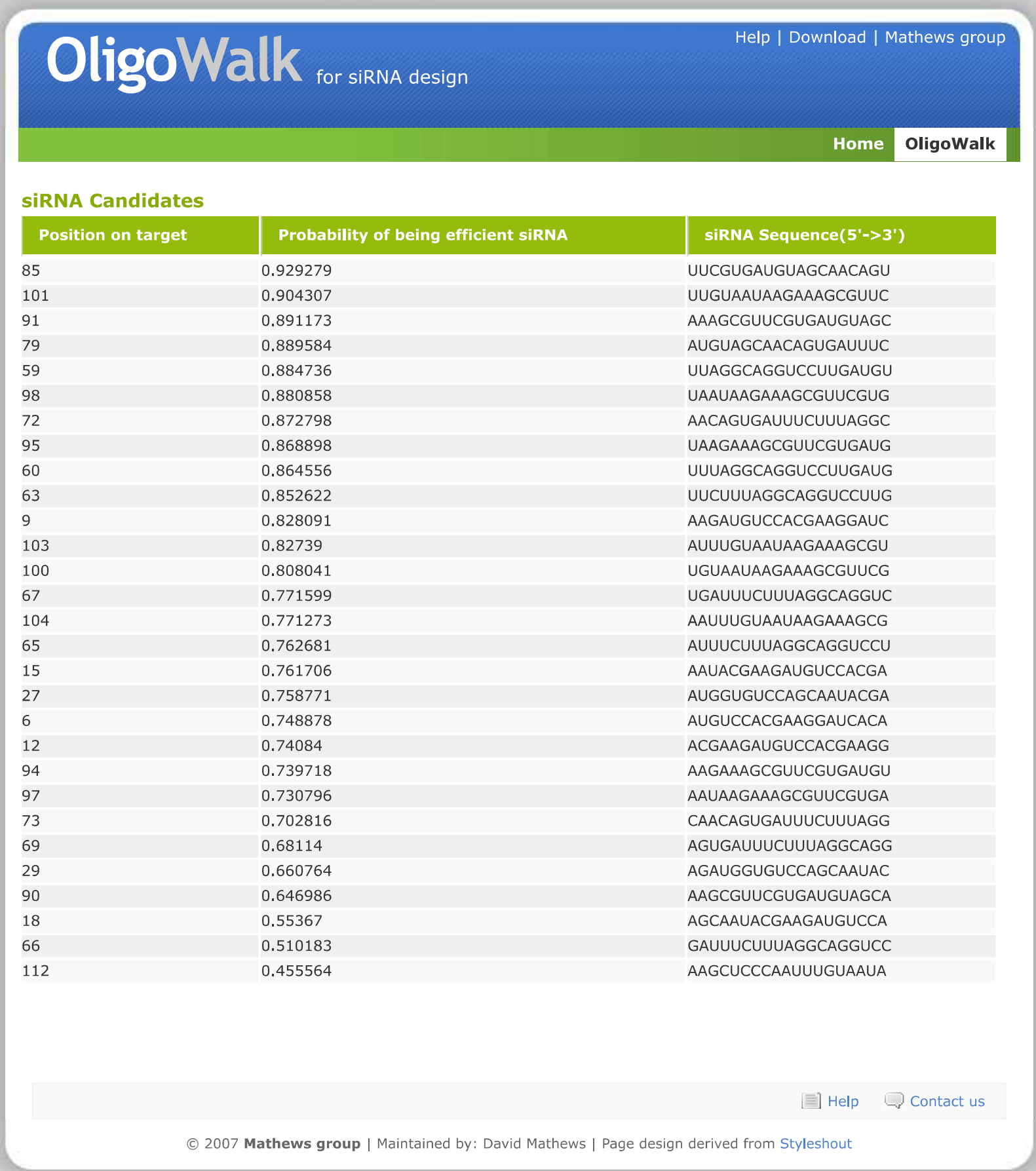

Supplement: Supplementary file 13 — Additional file 13: Supplementary Table 13. List of siRNAs predicted by OligoWalk for various conserved regions of the ‘M’ gene. [file 43141_2022_346_MOESM13_ESM.docx]
