# Supplementary material for: Design of siRNA molecules for silencing of membrane glycoprotein, nucleocapsid phosphoprotein, and surface glycoprotein genes of SARS-CoV2
Source: J Genet Eng Biotechnol. 2022 Apr 28;20:65. doi: 10.1186/s43141-022-00346-z (PMC9047631; doi:10.1186/s43141-022-00346-z)

**Supplementary Table 14: List of siRNAs predicted by OligoWalk for various conserved regions of the ‘N’ gene**

List of siRNAs predicted by OligoWalk for the ‘conserved region 3’ of the N gene


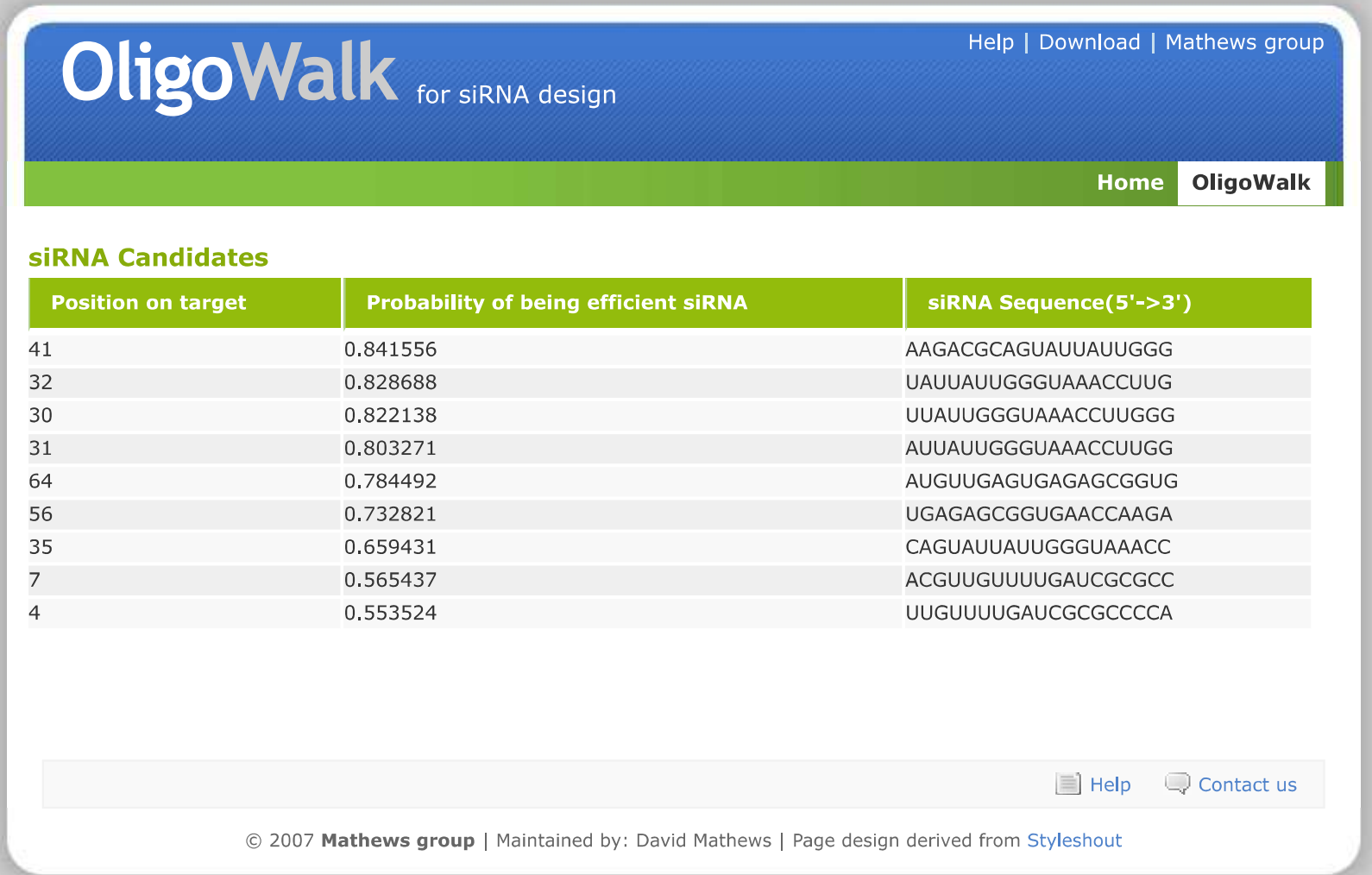


List of siRNAs predicted by OligoWalk for the ‘conserved region 4’ of the N gene


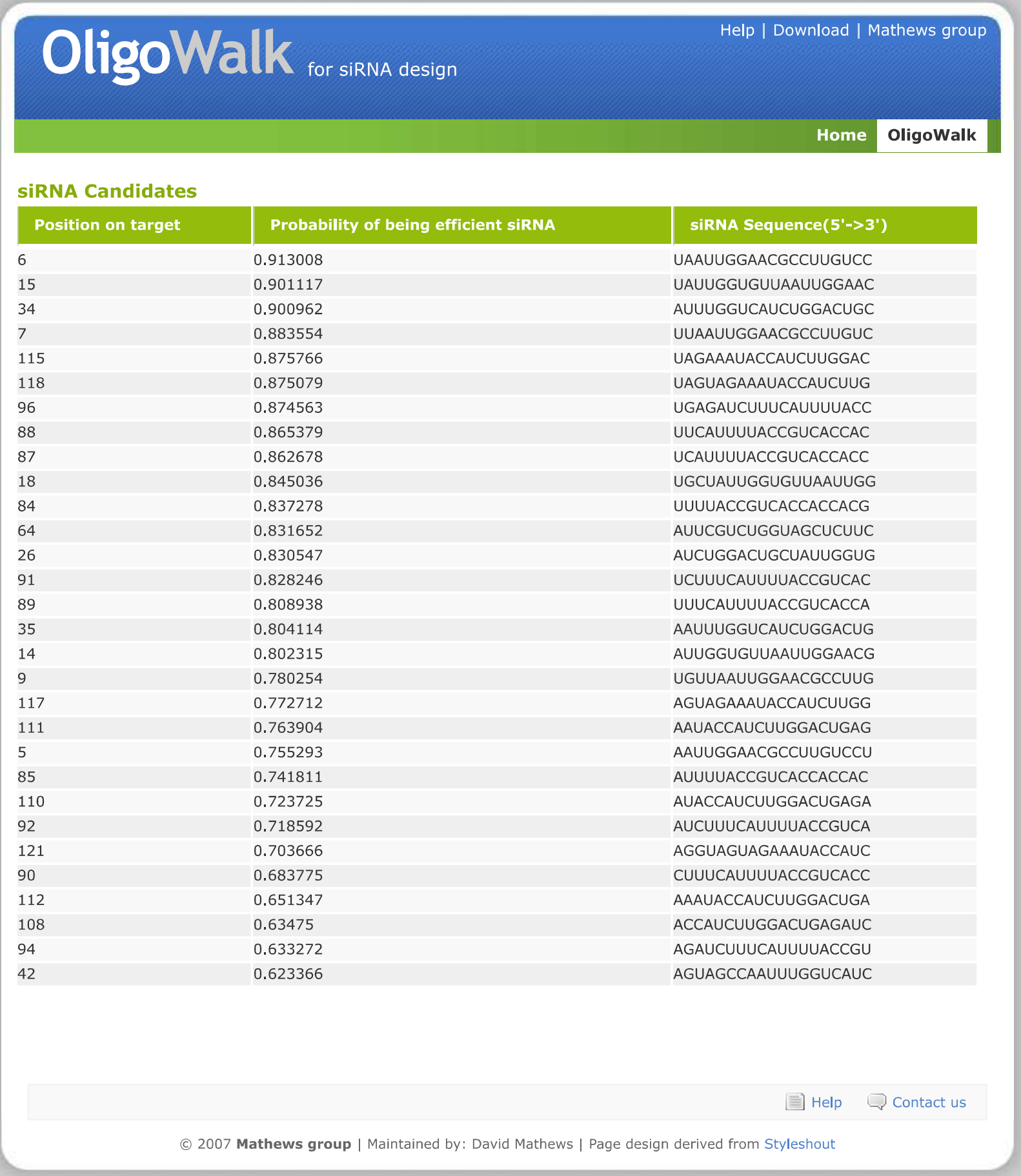


List of siRNAs predicted by OligoWalk for the ‘conserved region 7’ of the N gene


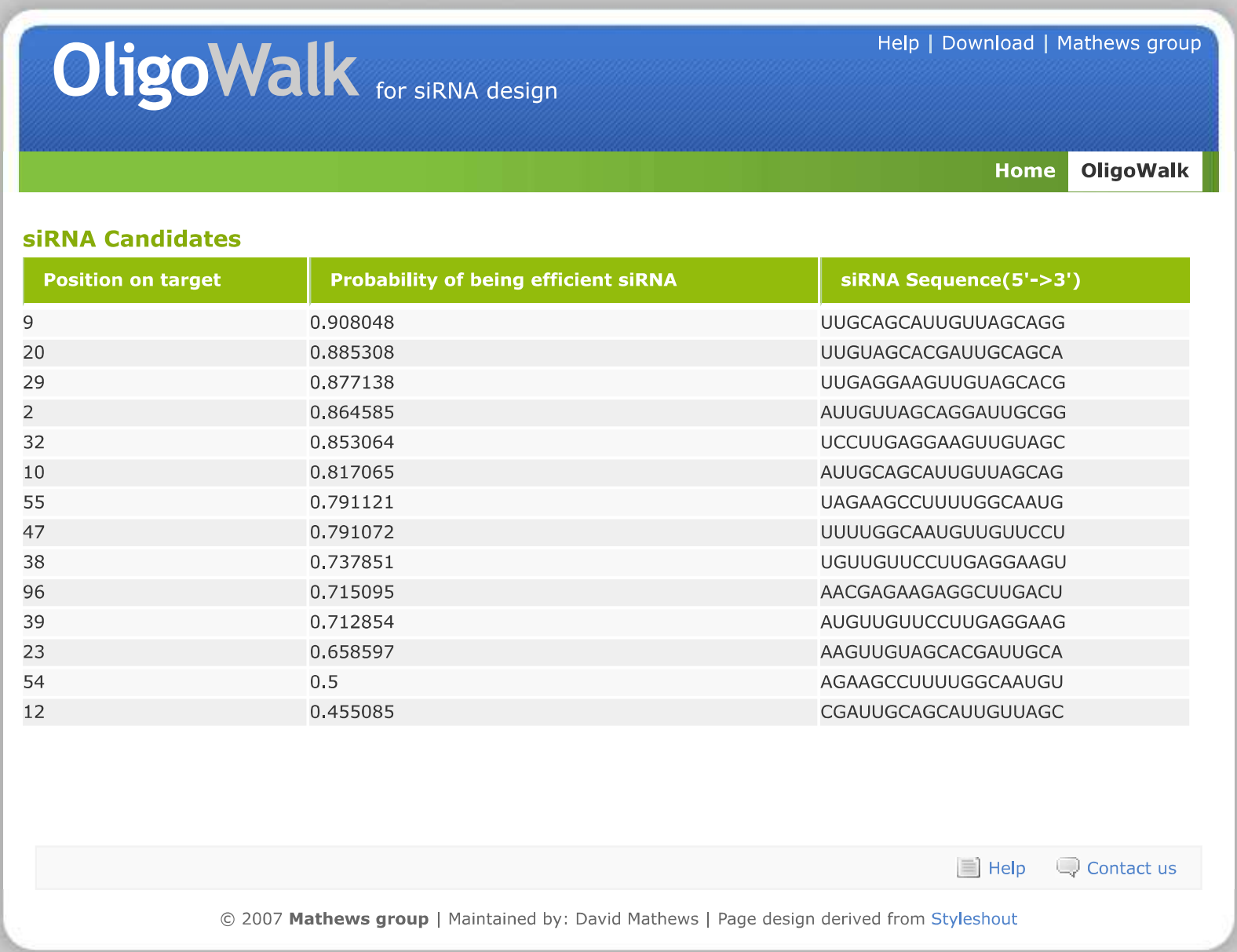


List of siRNAs predicted by OligoWalk for the ‘conserved region 10’ of the N gene


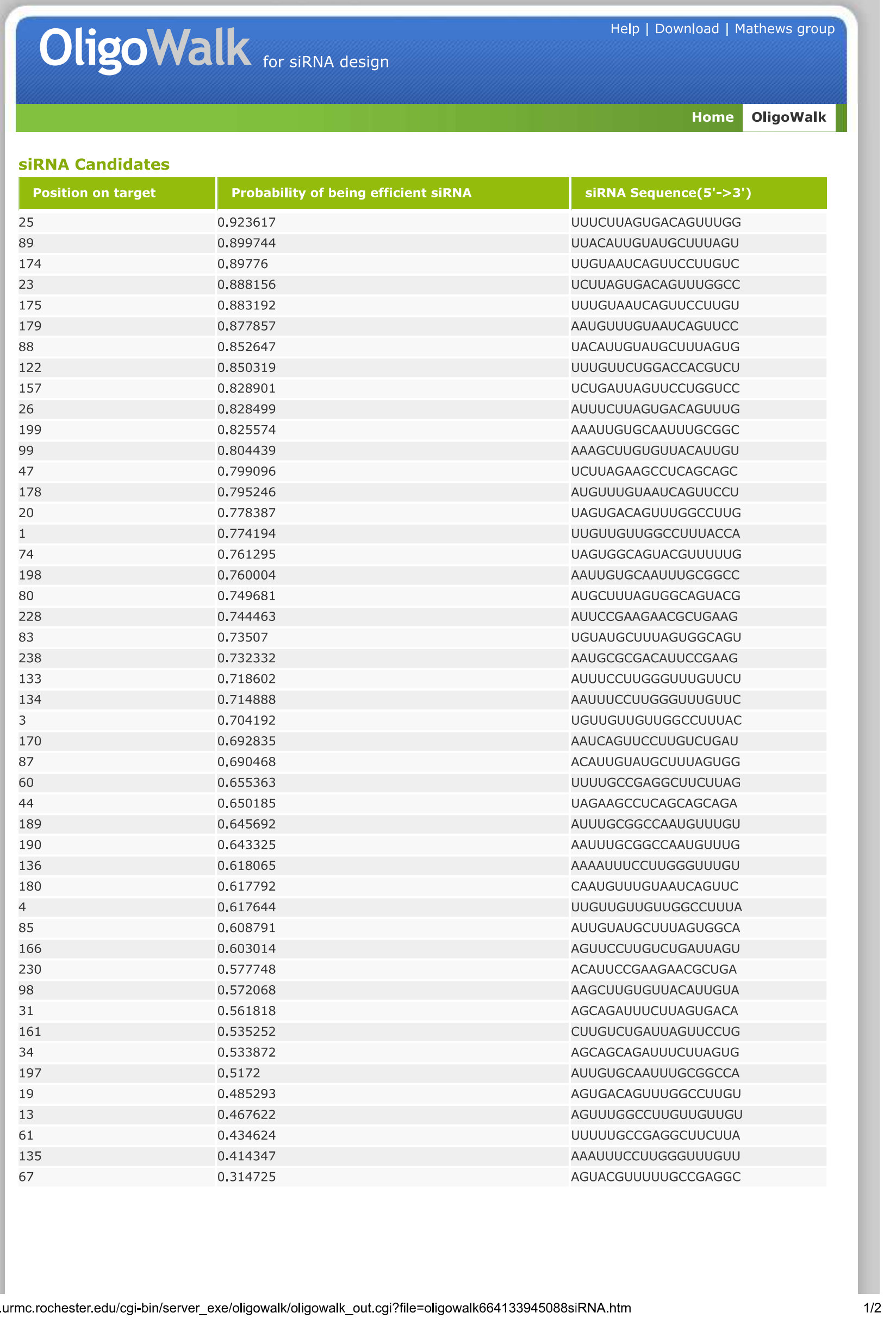


List of siRNAs predicted by OligoWalk for the ‘conserved region 11’ of the N gene


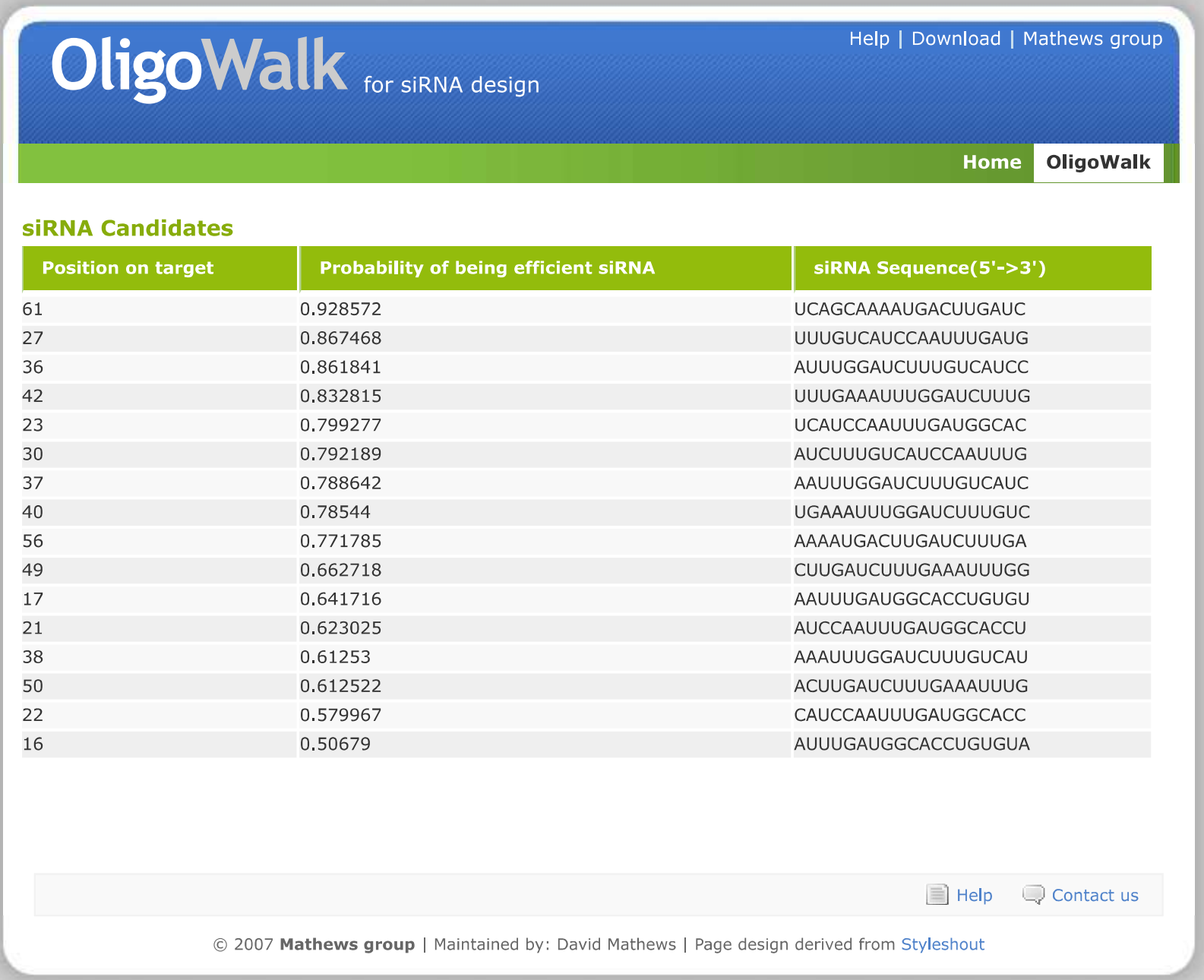


List of siRNAs predicted by OligoWalk for the ‘conserved region 14’ of the N gene


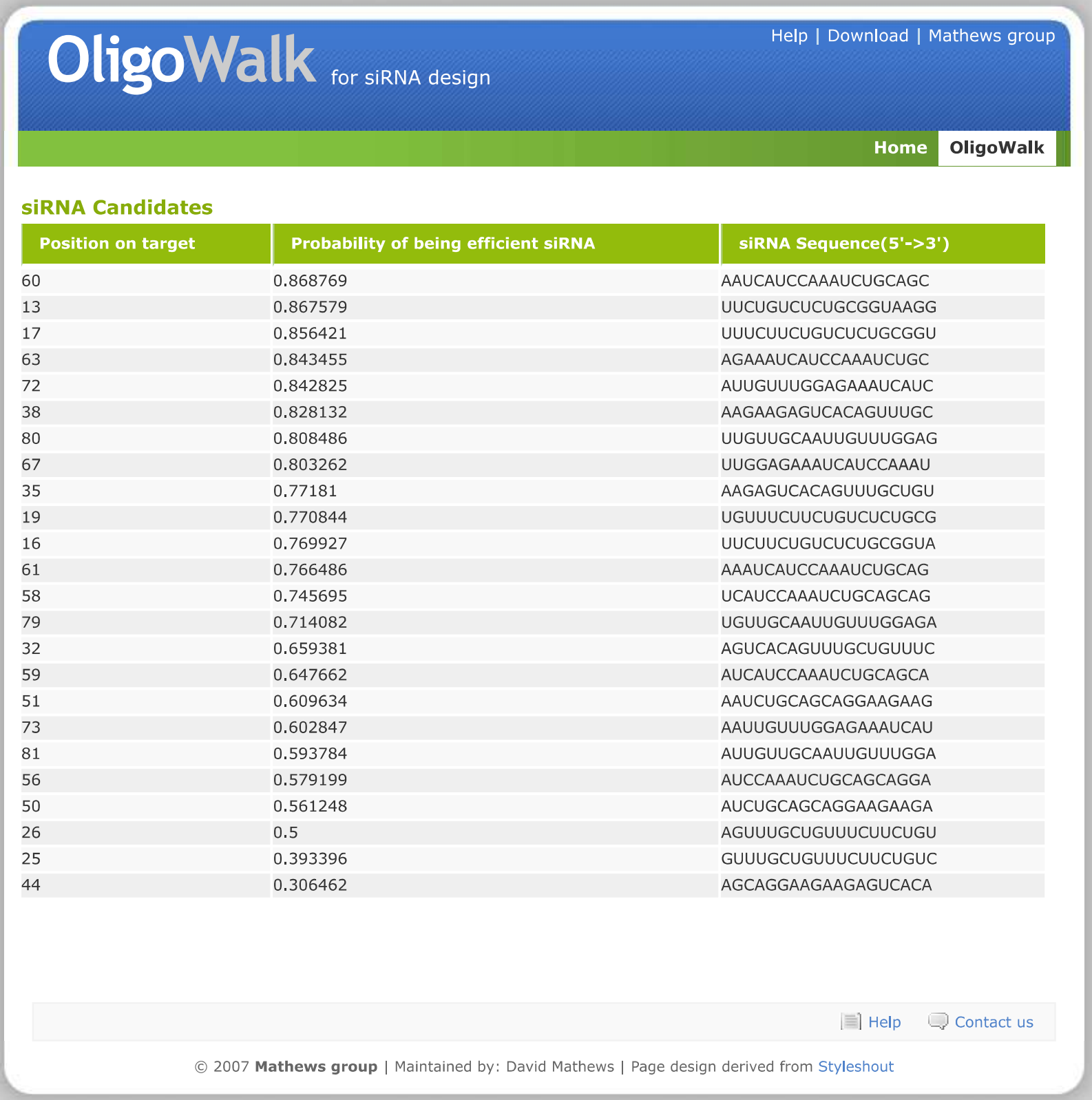

Supplement: Supplementary file 14 — Additional file 14: Supplementary Table 14. List of siRNAs predicted by OligoWalk for various conserved regions of the ‘N’ gene. [file 43141_2022_346_MOESM14_ESM.docx]
