# Supplementary material for: Design of siRNA molecules for silencing of membrane glycoprotein, nucleocapsid phosphoprotein, and surface glycoprotein genes of SARS-CoV2
Source: J Genet Eng Biotechnol. 2022 Apr 28;20:65. doi: 10.1186/s43141-022-00346-z (PMC9047631; doi:10.1186/s43141-022-00346-z)

**Supplementary Table 15: List of siRNAs predicted by OligoWalk for various conserved regions of the ‘S’ gene**

List of siRNAs predicted by OligoWalk for the ‘conserved region 1’ of the S gene


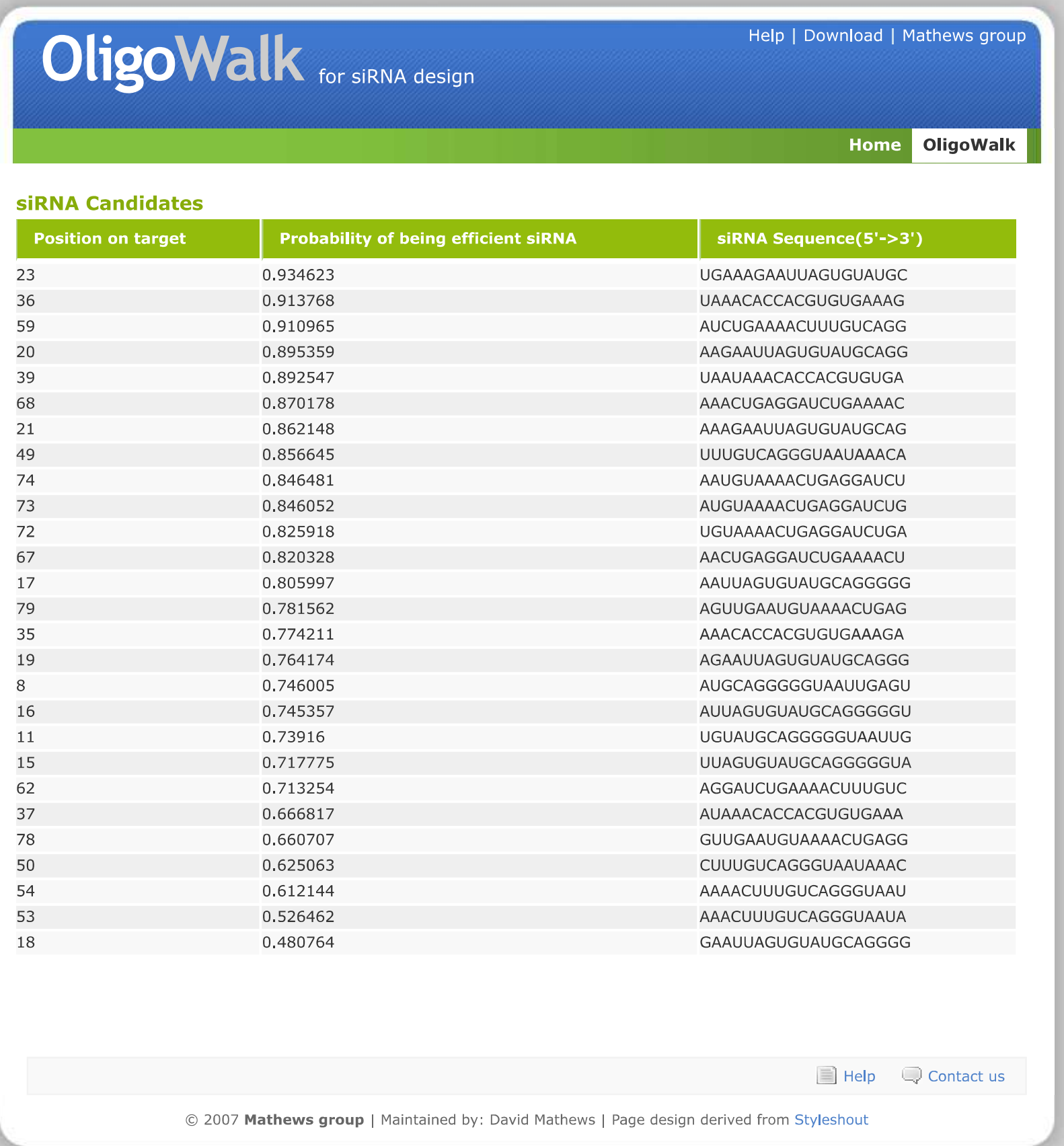


List of siRNAs predicted by OligoWalk for the ‘conserved region 5’ of the S gene


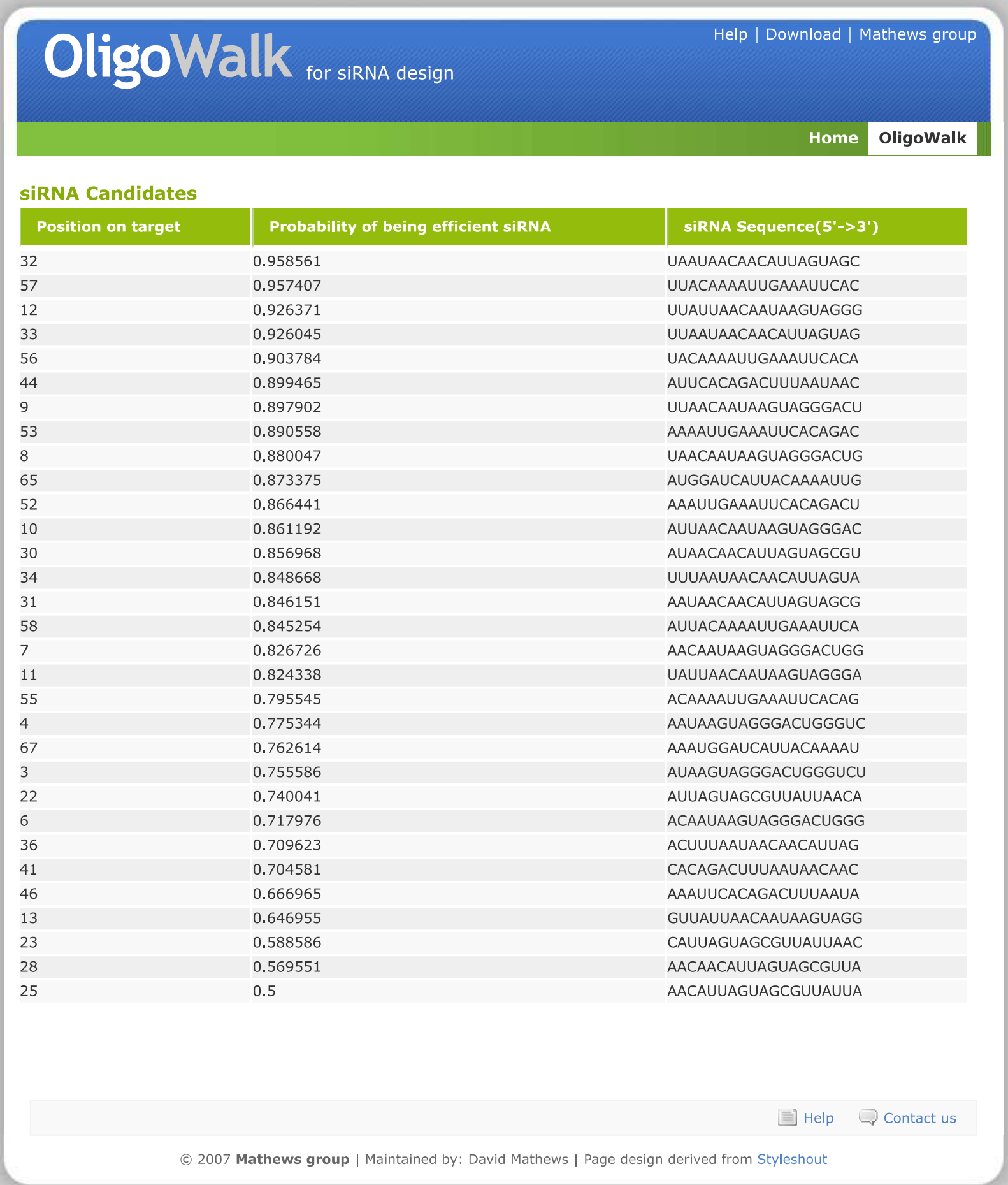


List of siRNAs predicted by OligoWalk for the ‘conserved region 6’ of the S gene


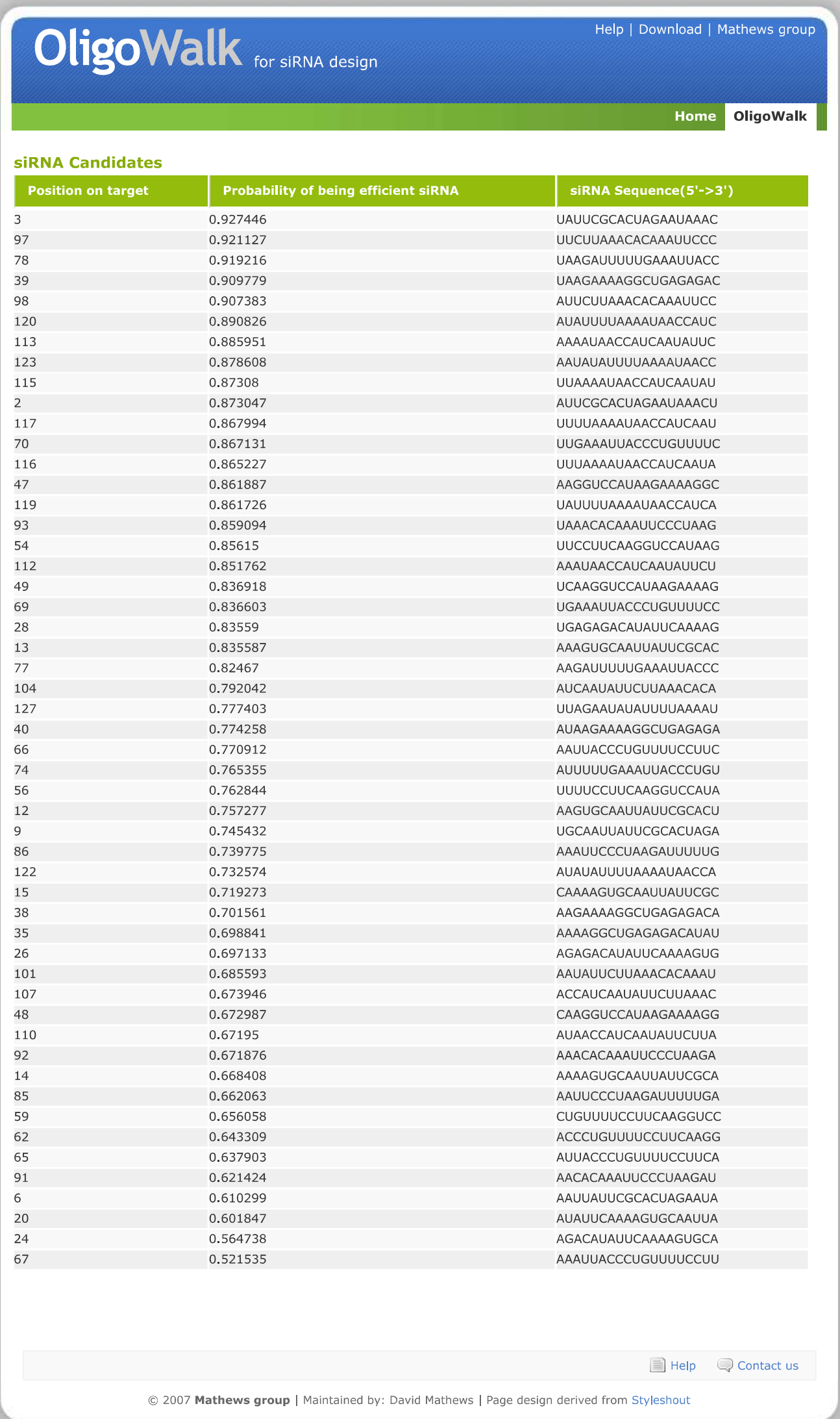


List of siRNAs predicted by OligoWalk for the ‘conserved region 9’ of the S gene


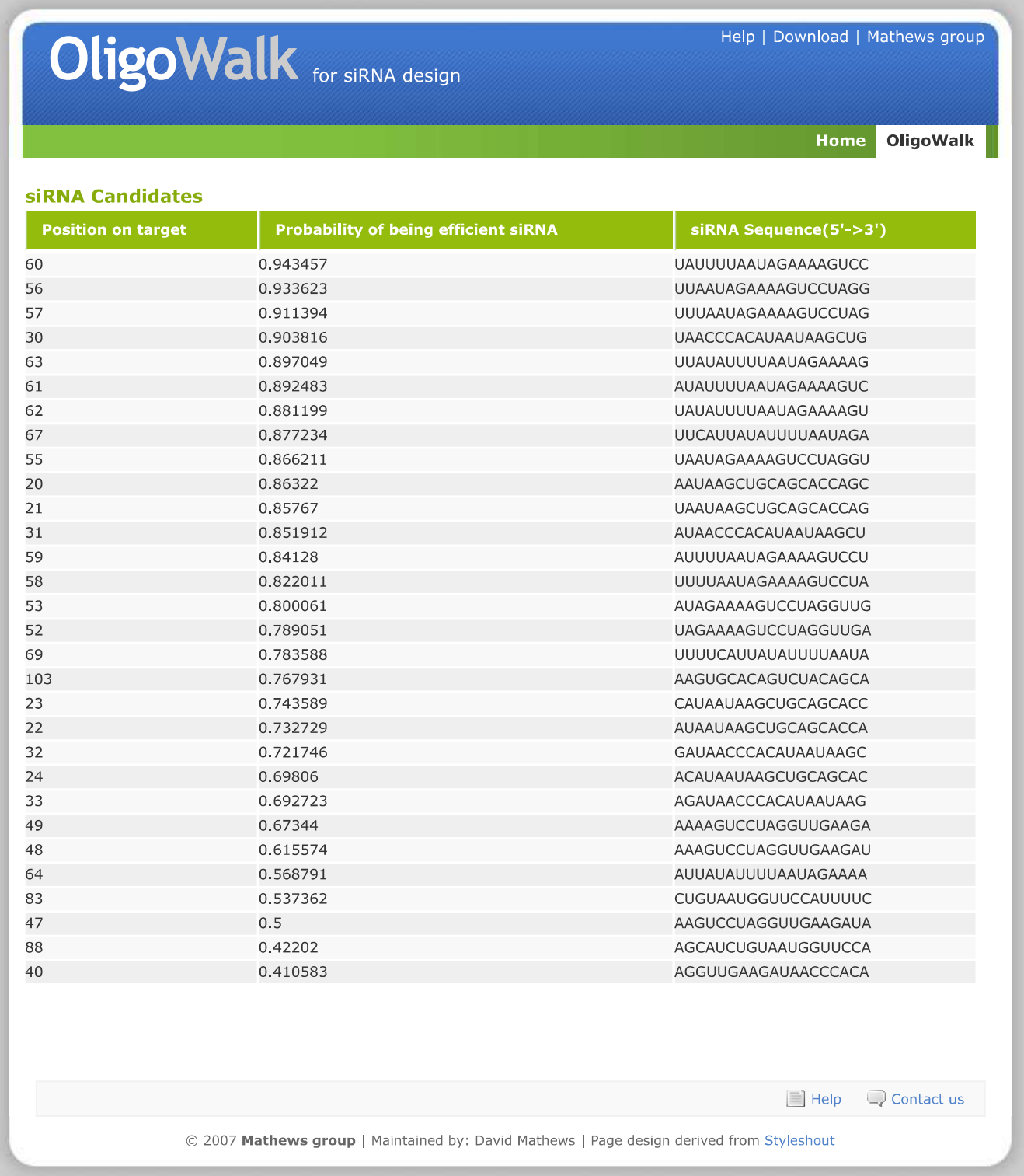


List of siRNAs predicted by OligoWalk for the ‘conserved region 10’ of the S gene


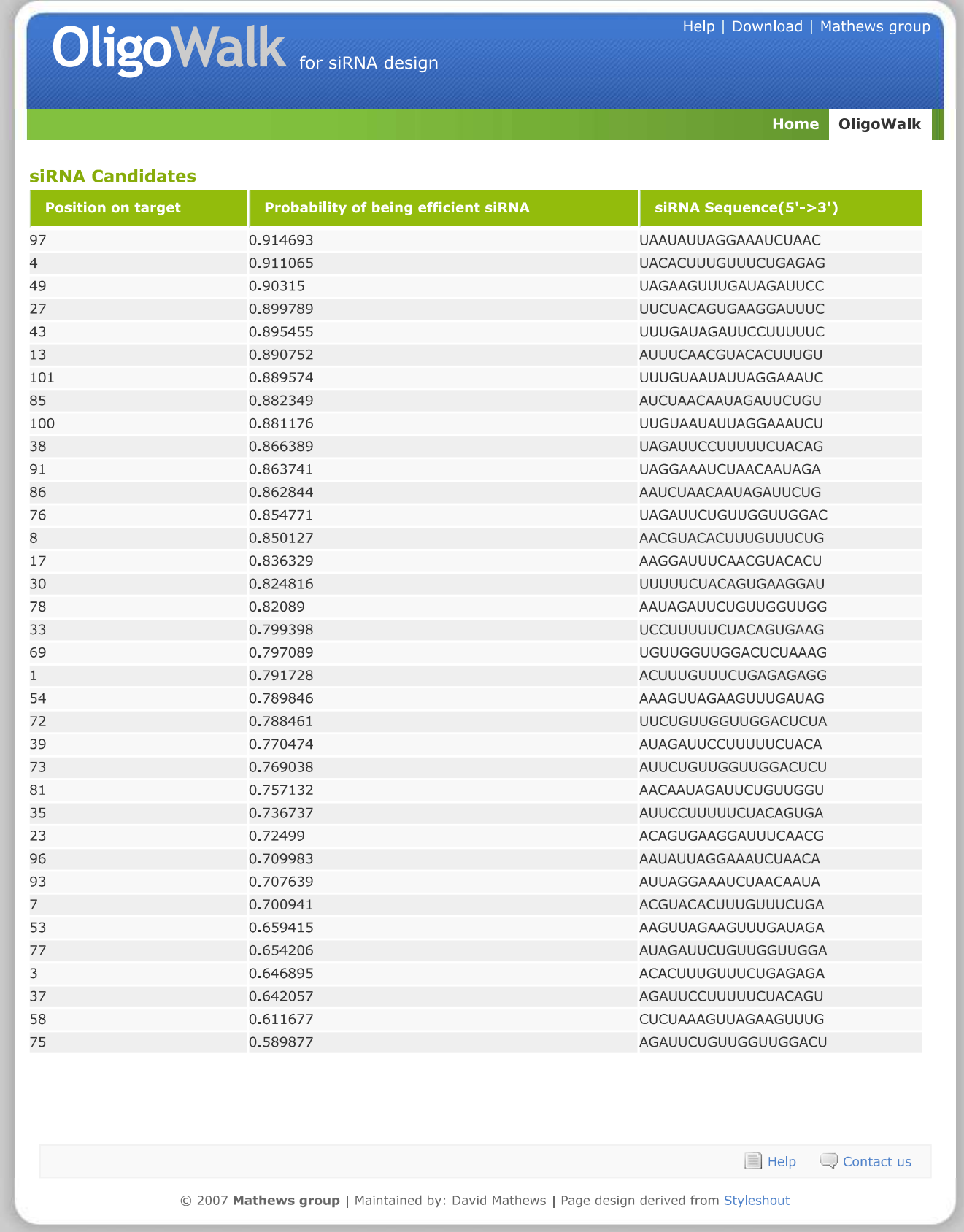


List of siRNAs predicted by OligoWalk for the ‘conserved region 12’ of the S gene


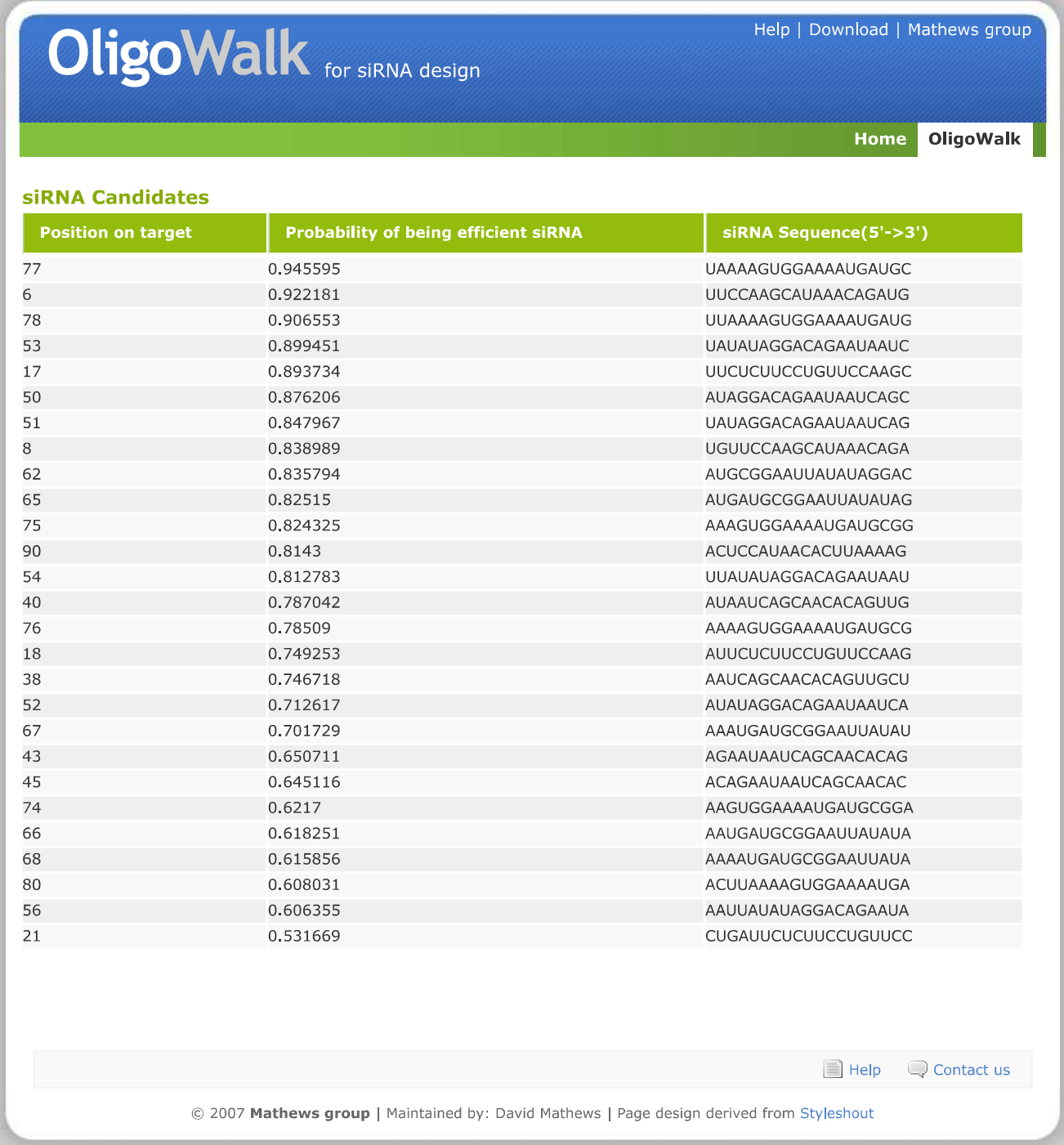


List of siRNAs predicted by OligoWalk for the ‘conserved region 13’ of the S gene


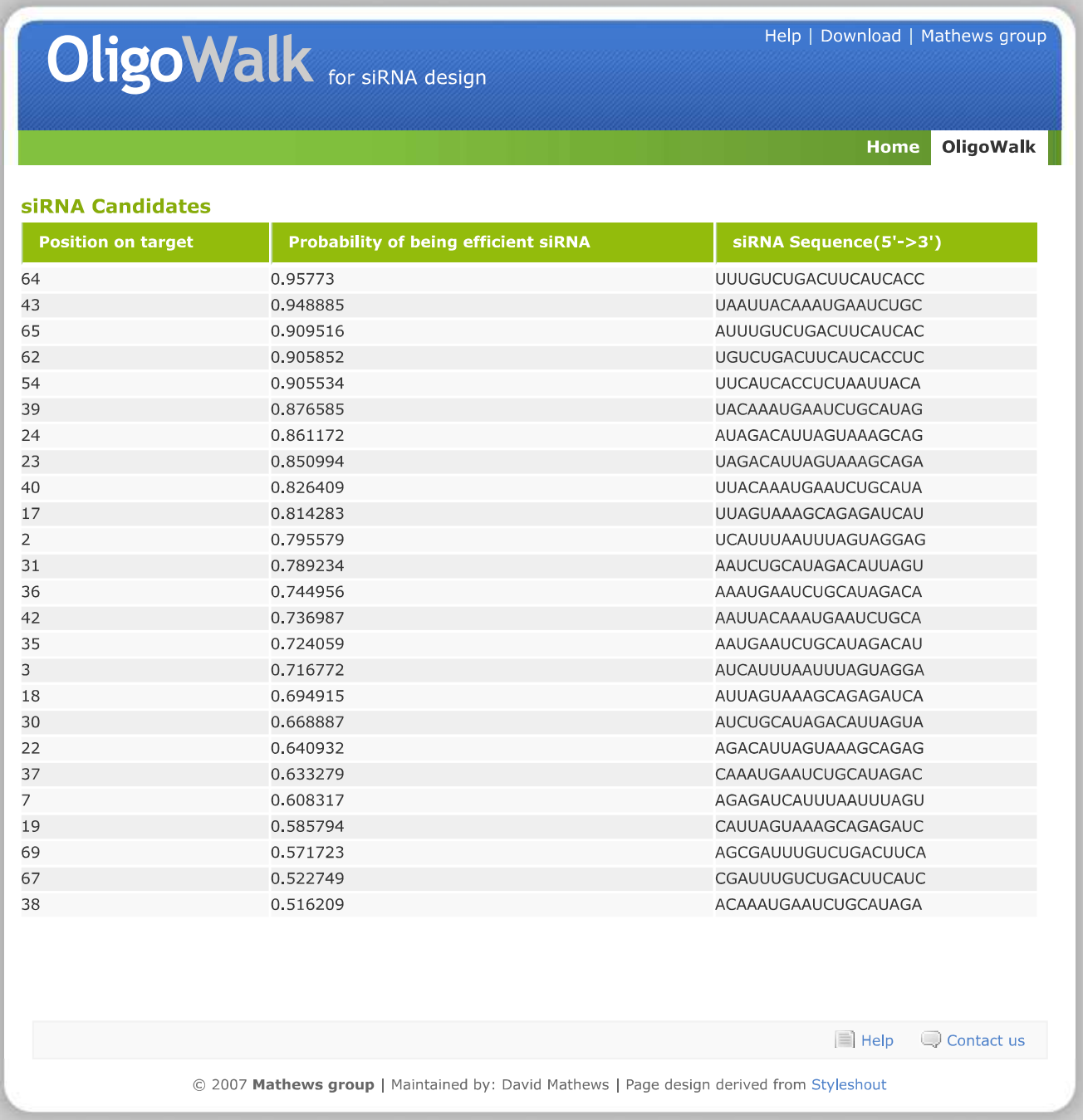


List of siRNAs predicted by OligoWalk for the ‘conserved region 14’ of the S gene


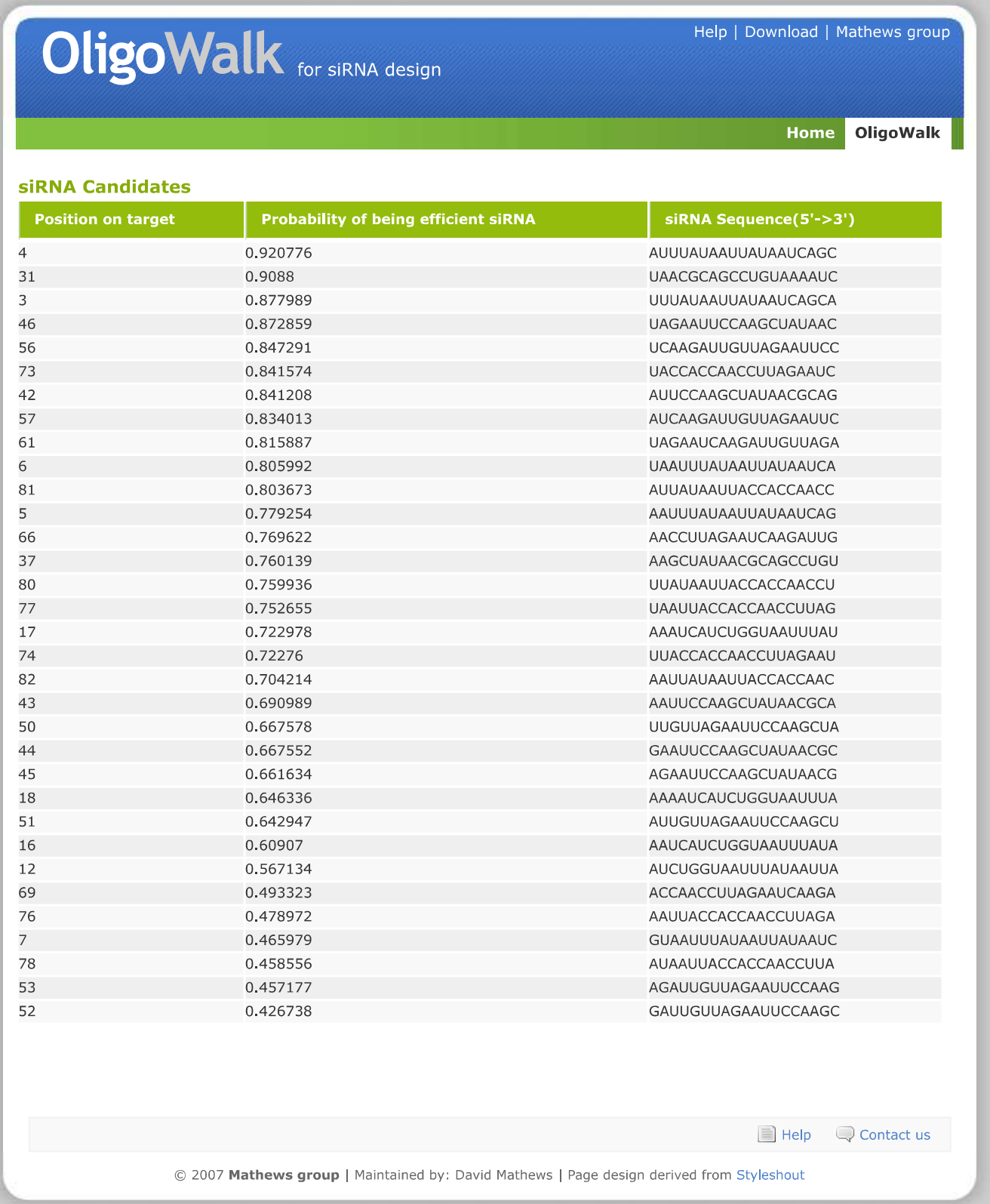


List of siRNAs predicted by OligoWalk for the ‘conserved region 19’ of the S gene


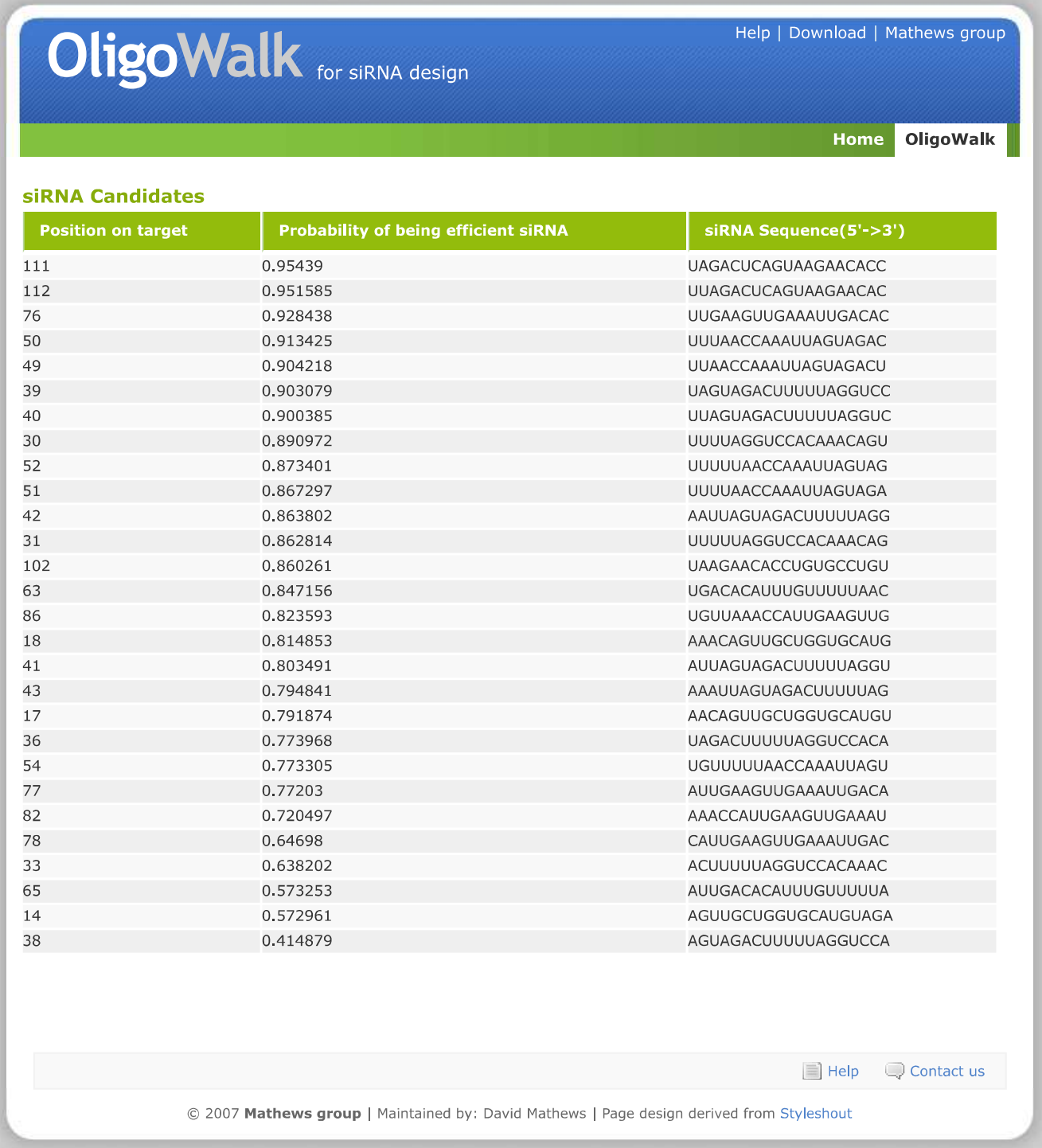


List of siRNAs predicted by OligoWalk for the ‘conserved region 22’ of the S gene


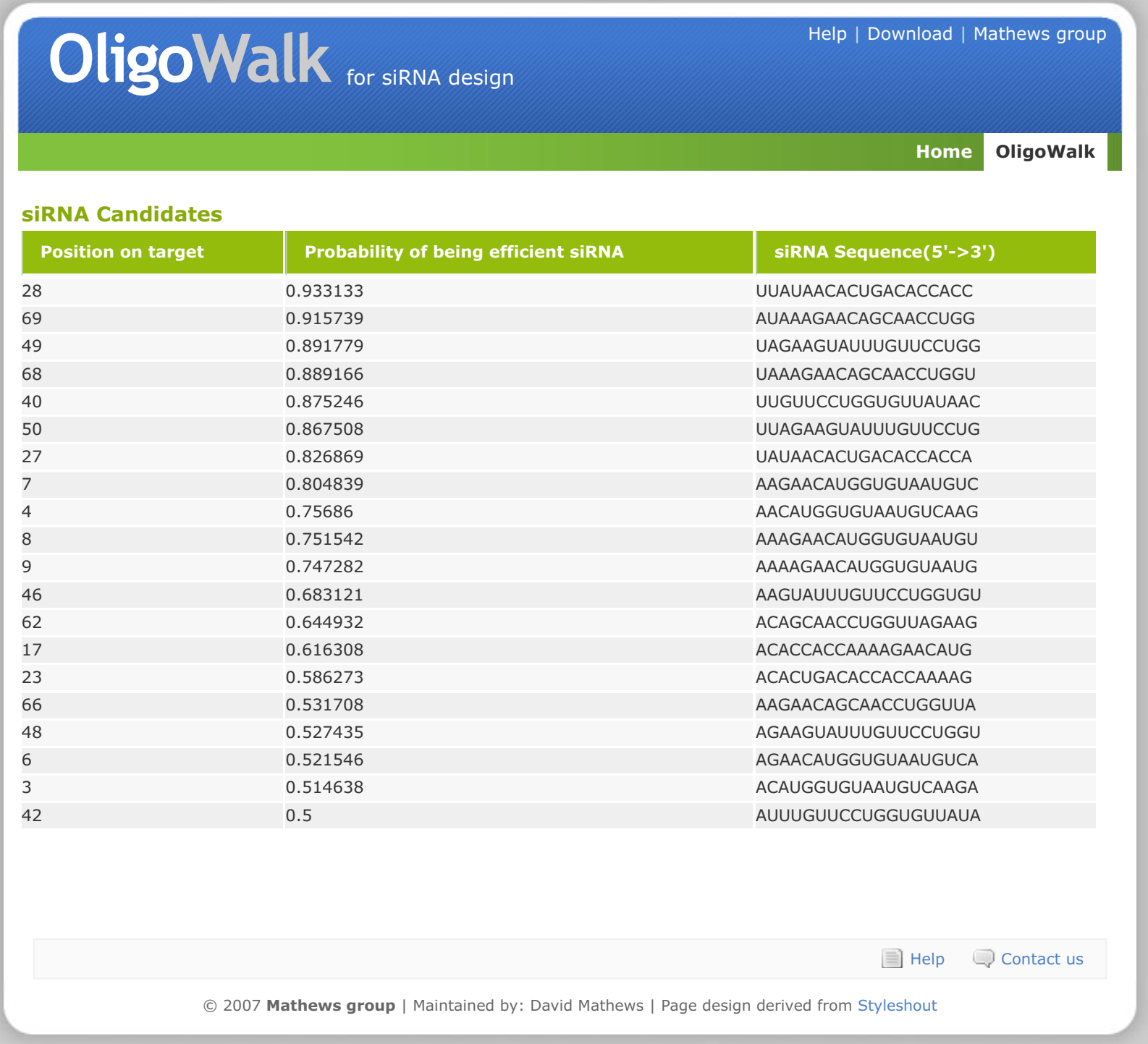


List of siRNAs predicted by OligoWalk for the ‘conserved region 23’ of the S gene


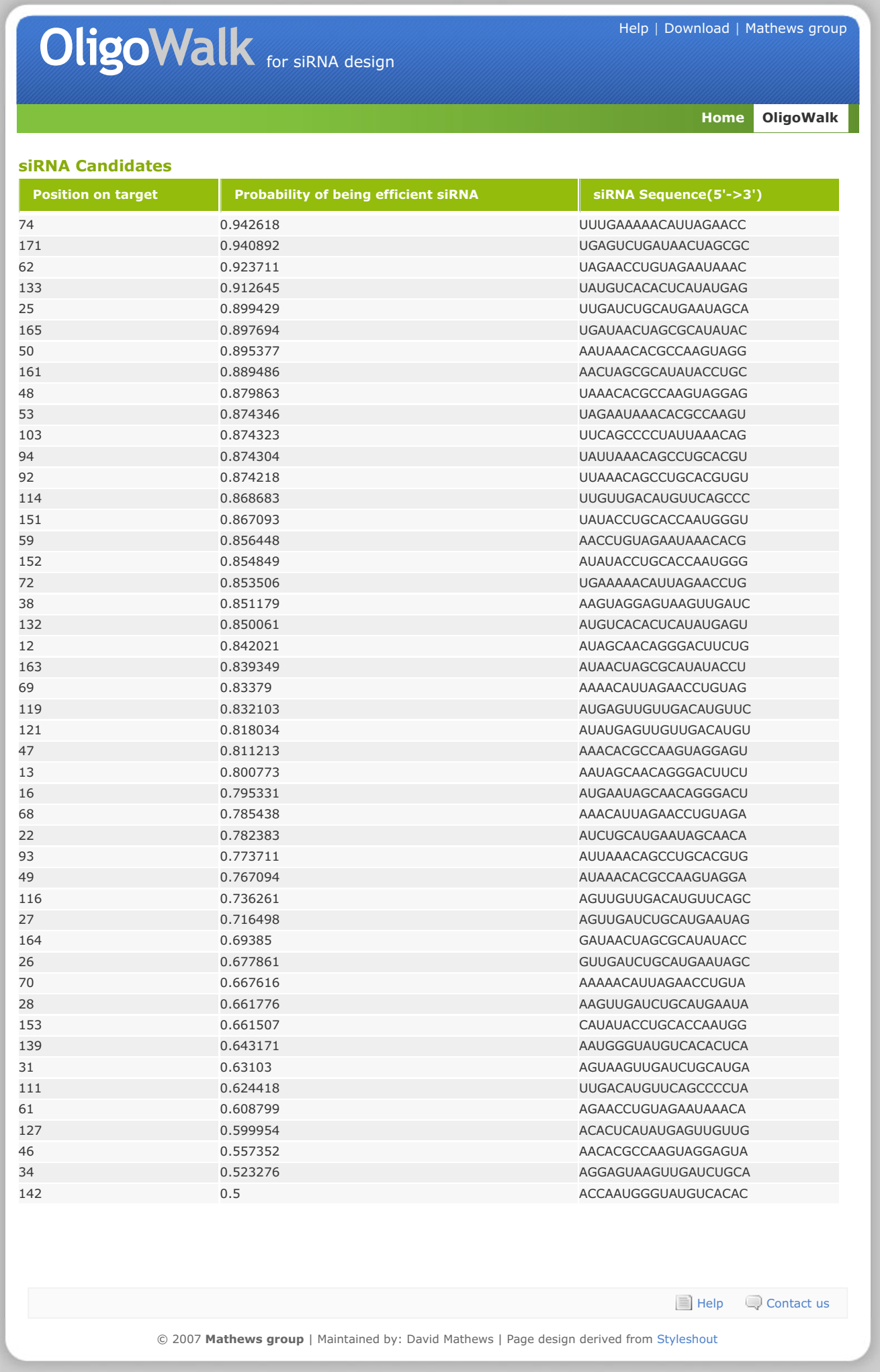


List of siRNAs predicted by OligoWalk for the ‘conserved region 28’ of the S gene


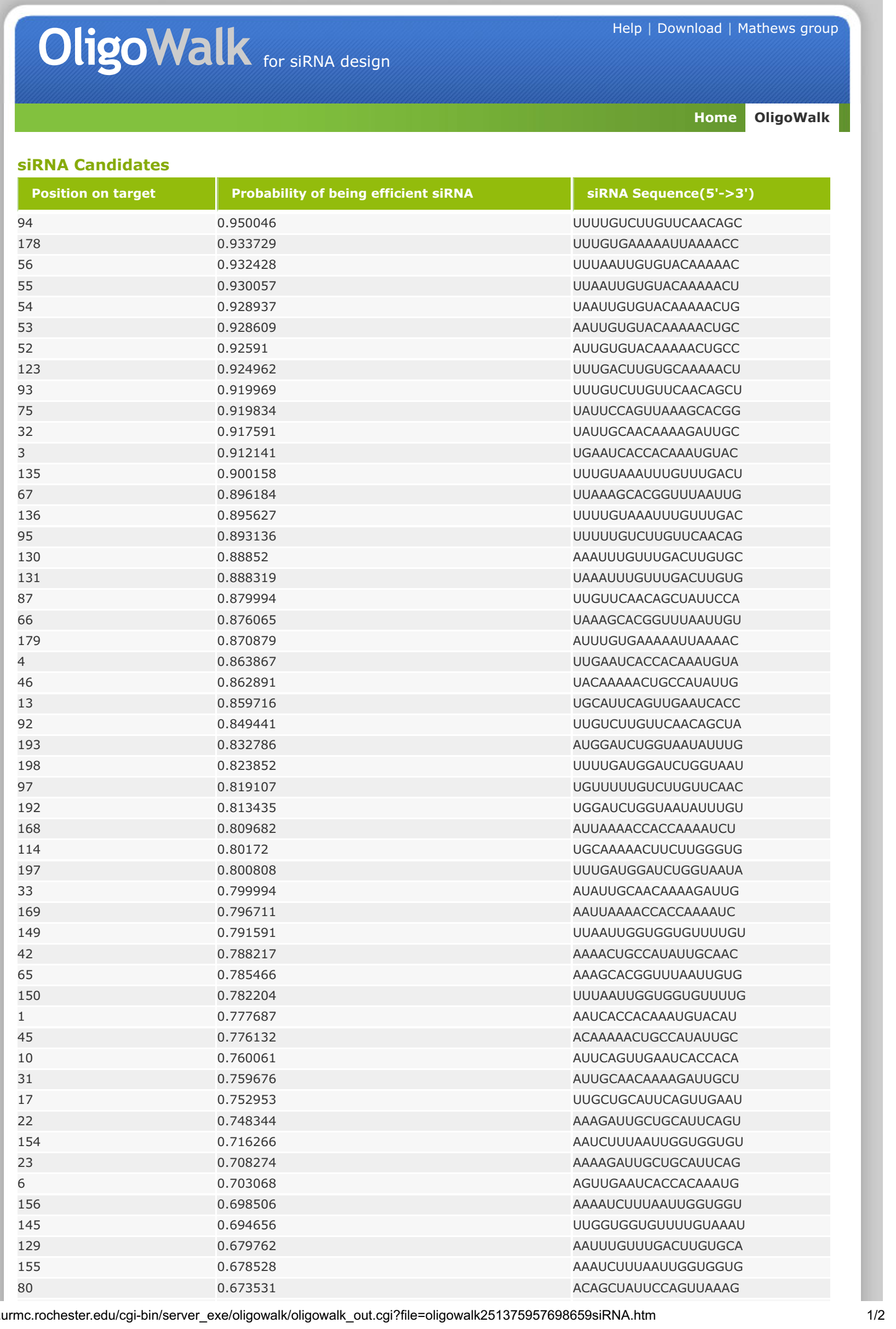


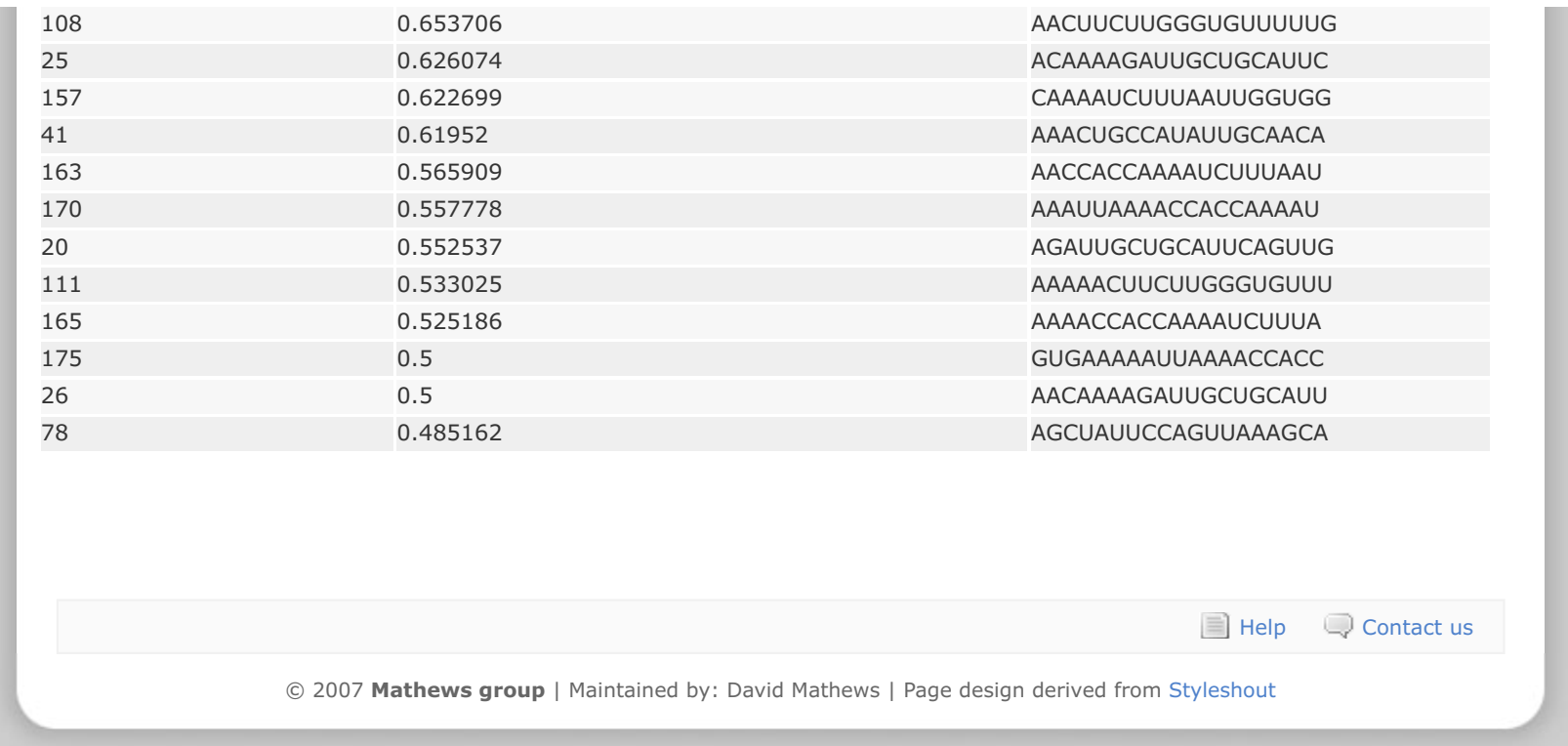


List of siRNAs predicted by OligoWalk for the ‘conserved region 30’ of the S gene


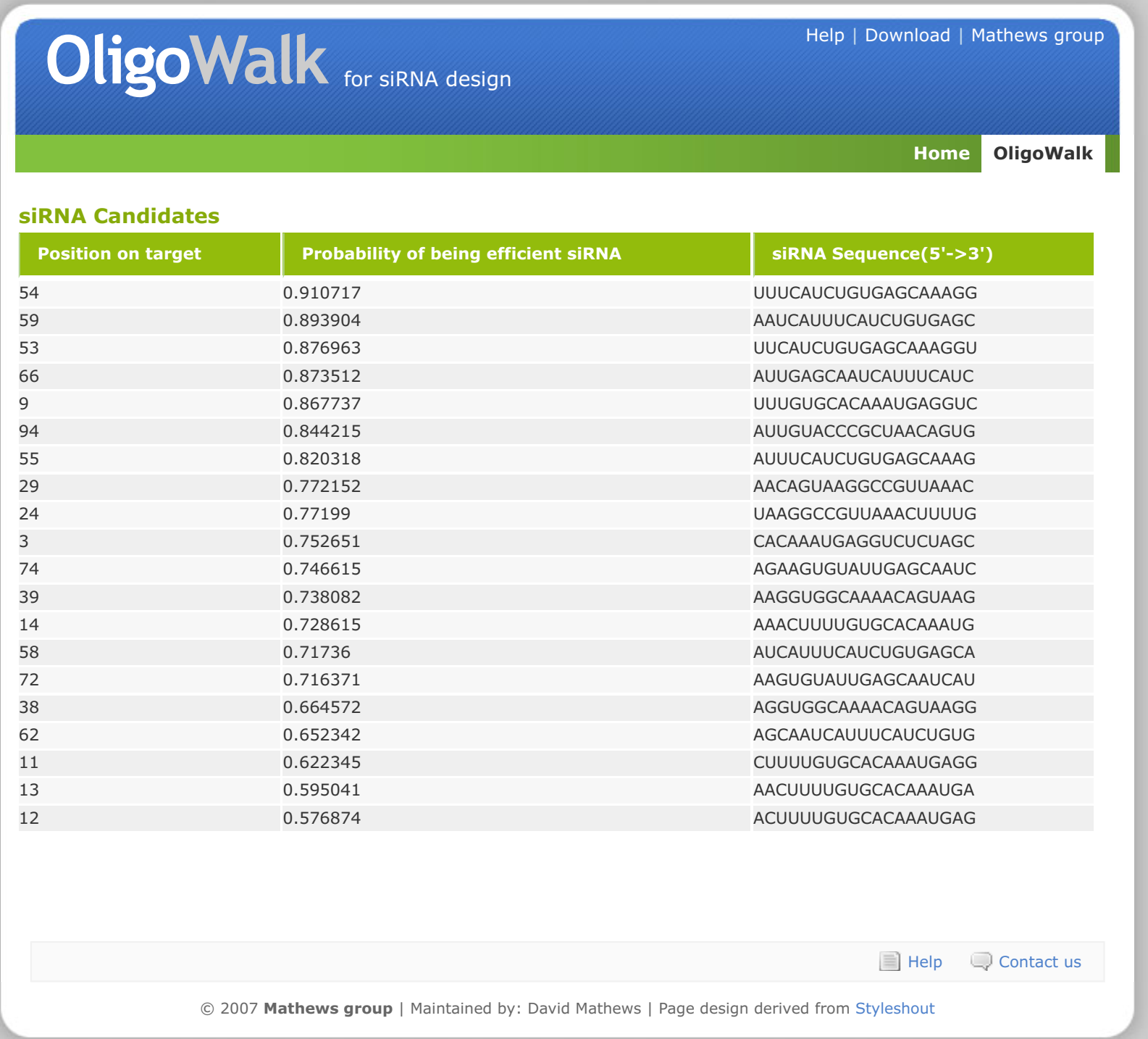


List of siRNAs predicted by OligoWalk for the ‘conserved region 31’ of the S gene


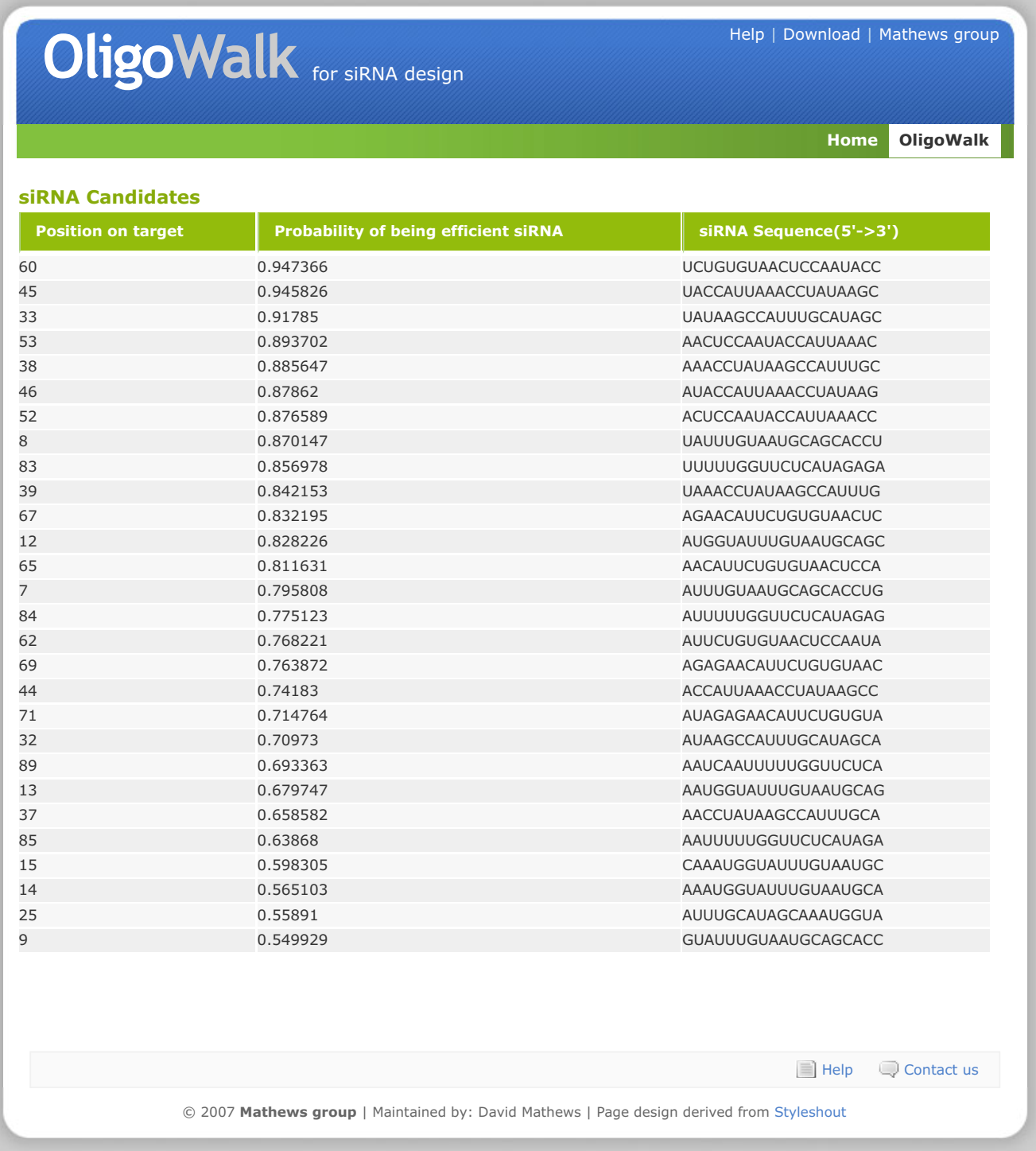


List of siRNAs predicted by OligoWalk for the ‘conserved region 33’ of the S gene


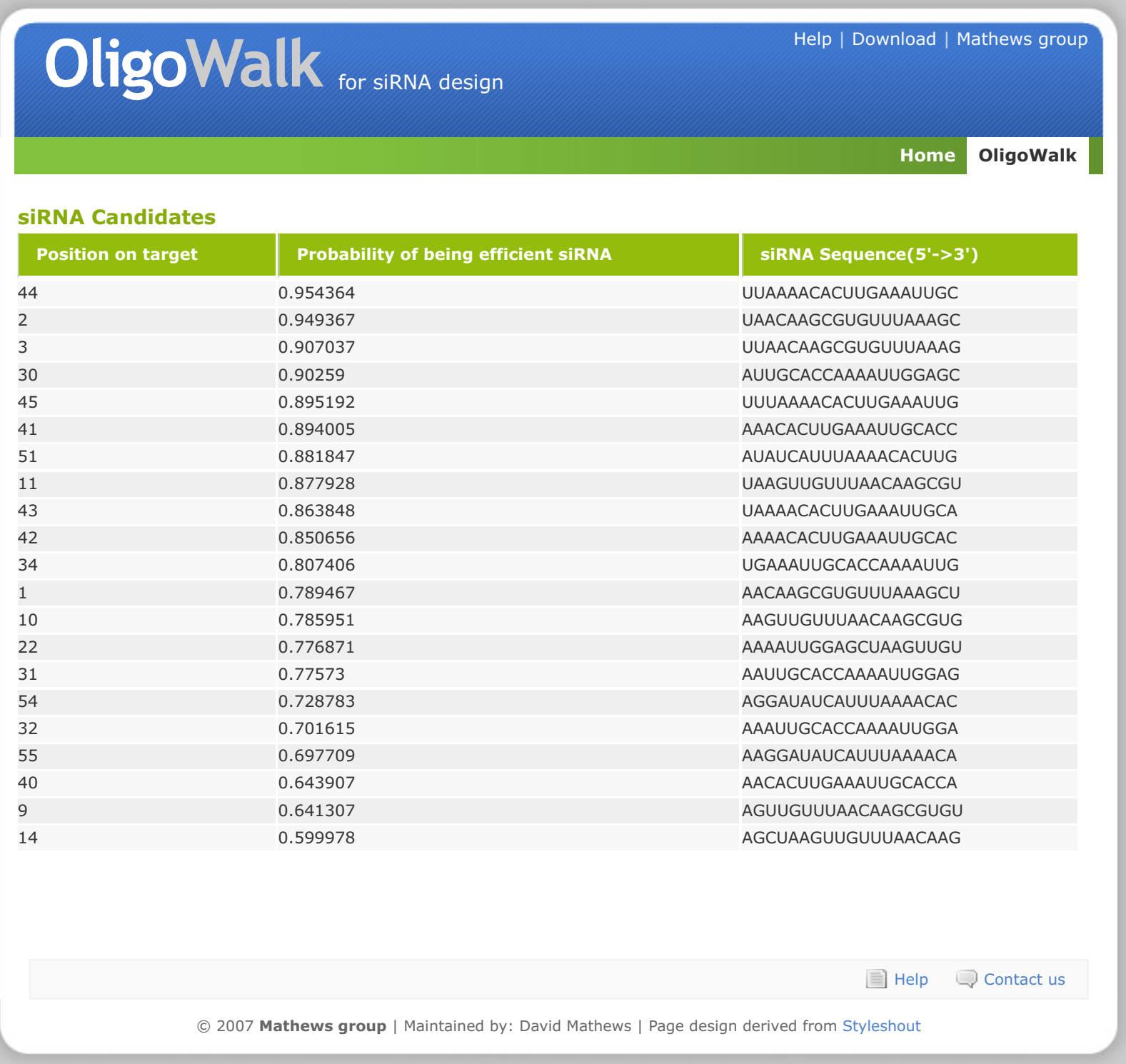


List of siRNAs predicted by OligoWalk for the ‘conserved region 34’ of the S gene


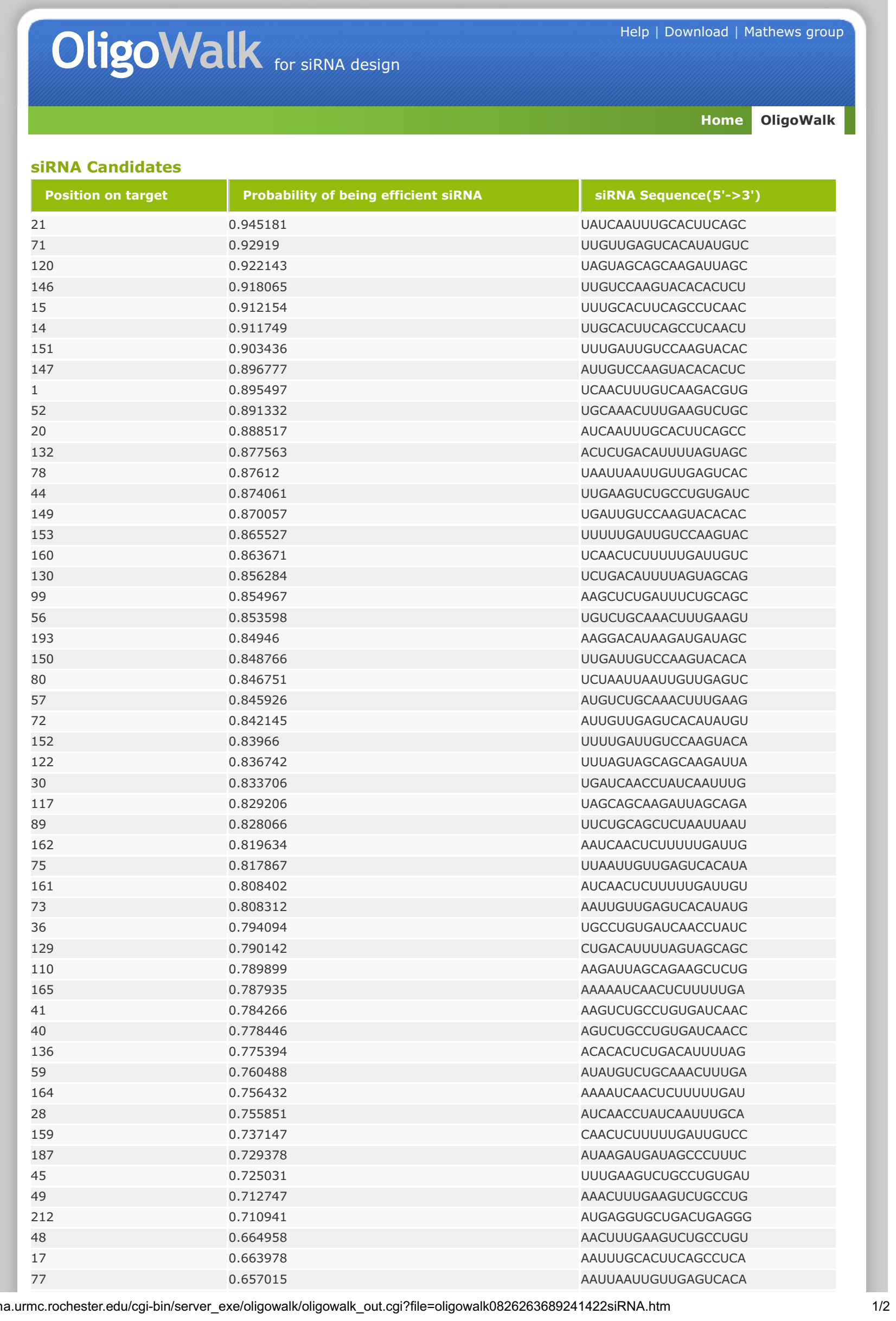


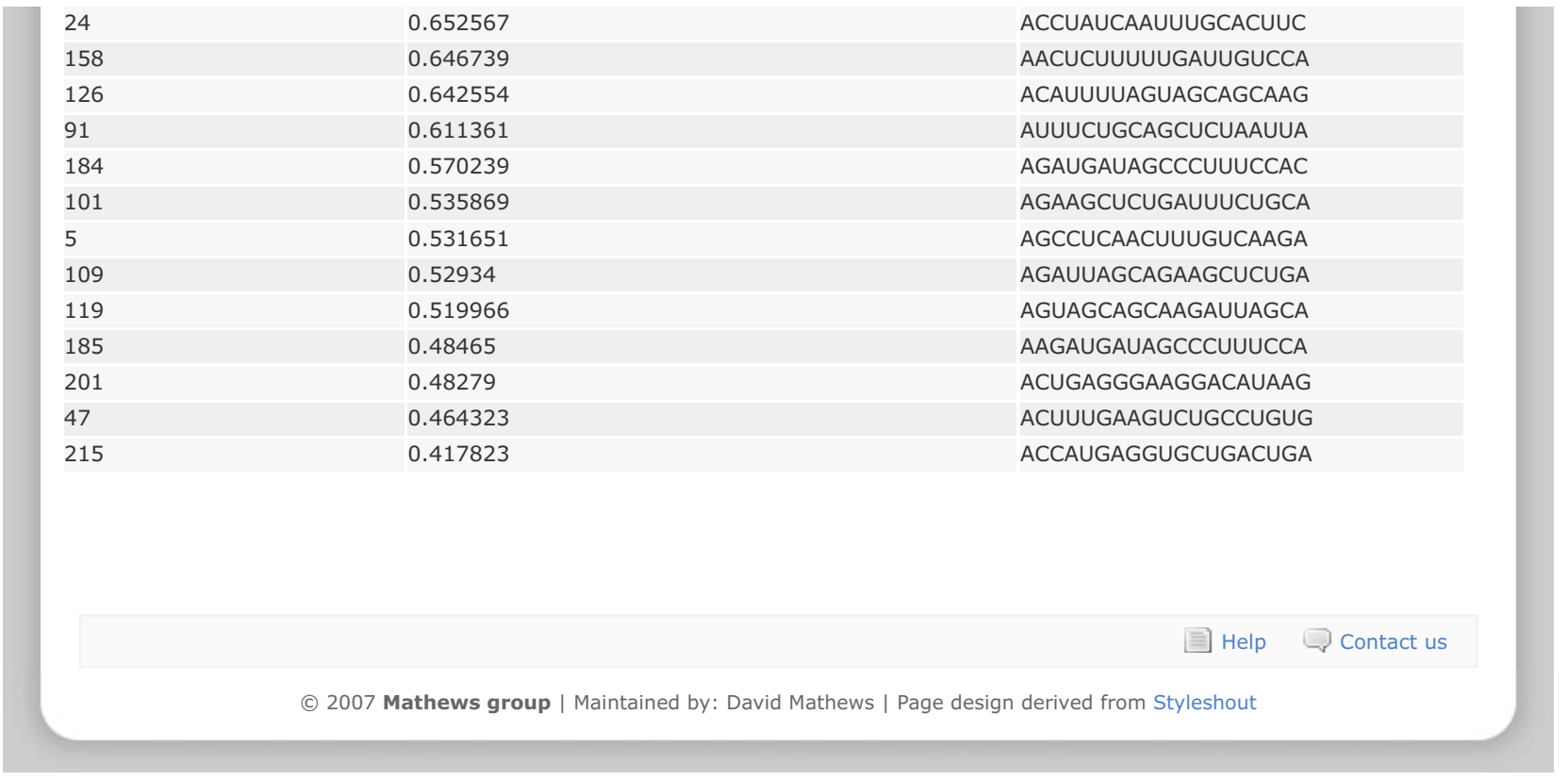


List of siRNAs predicted by OligoWalk for the ‘conserved region 40’ of the S gene


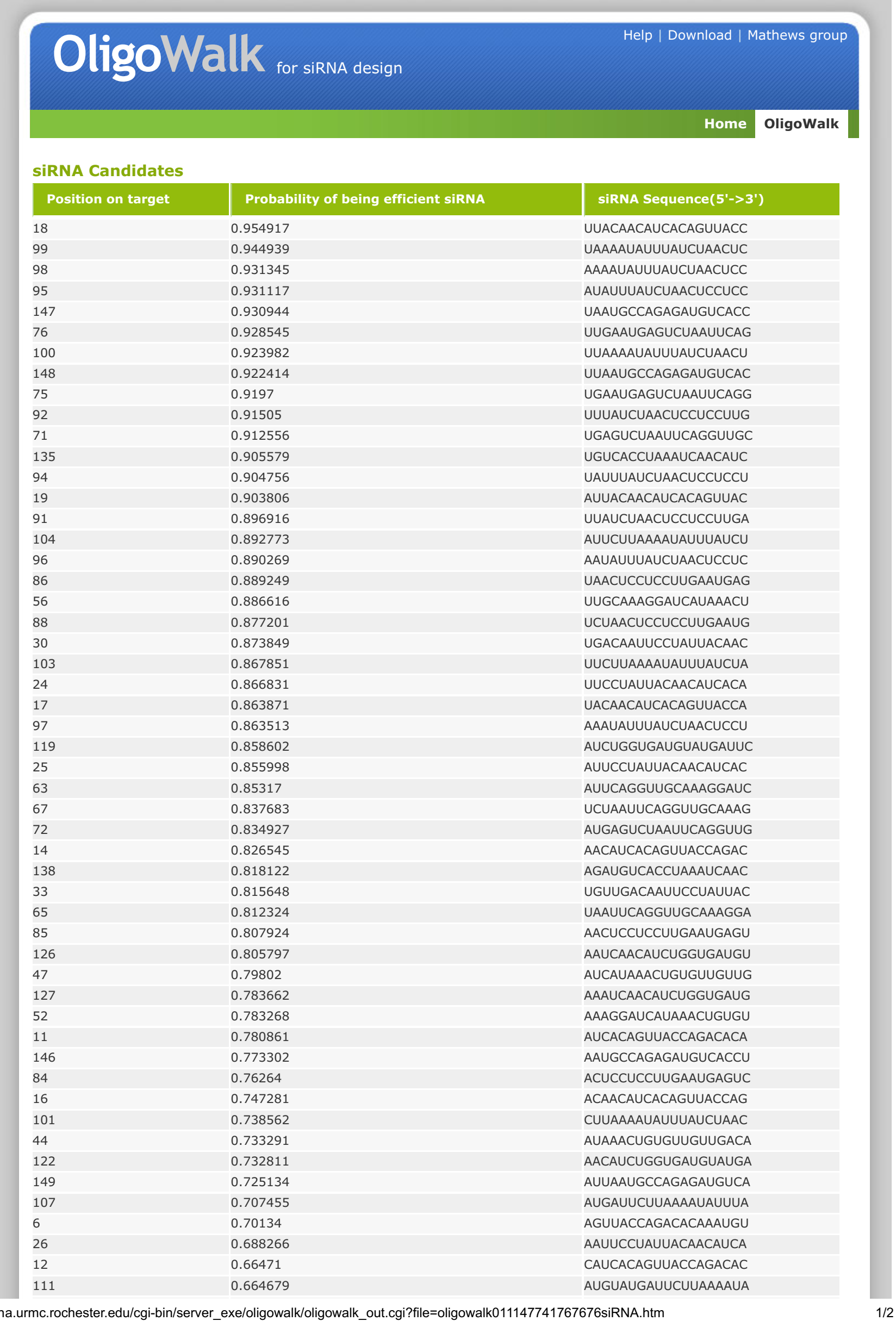


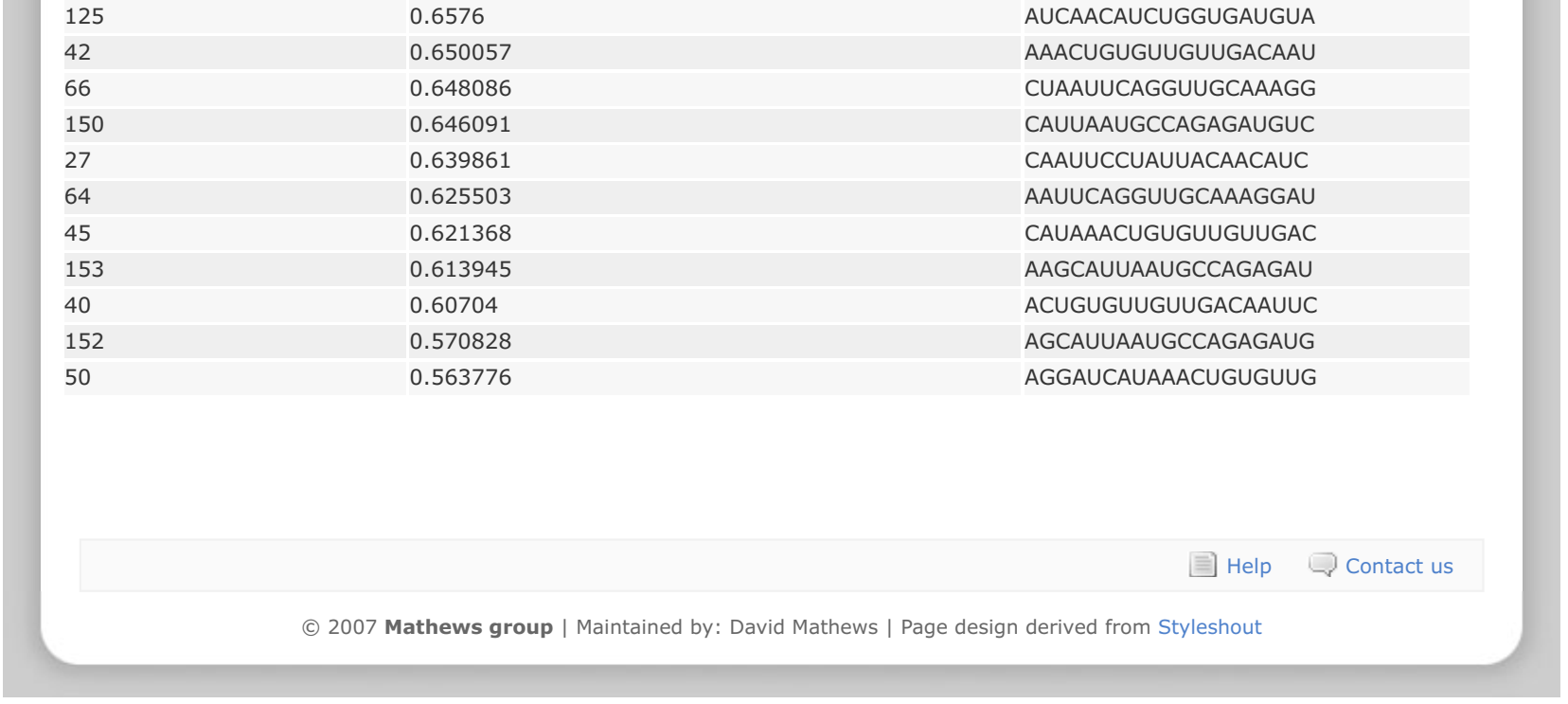


List of siRNAs predicted by OligoWalk for the ‘conserved region 41’ of the S gene


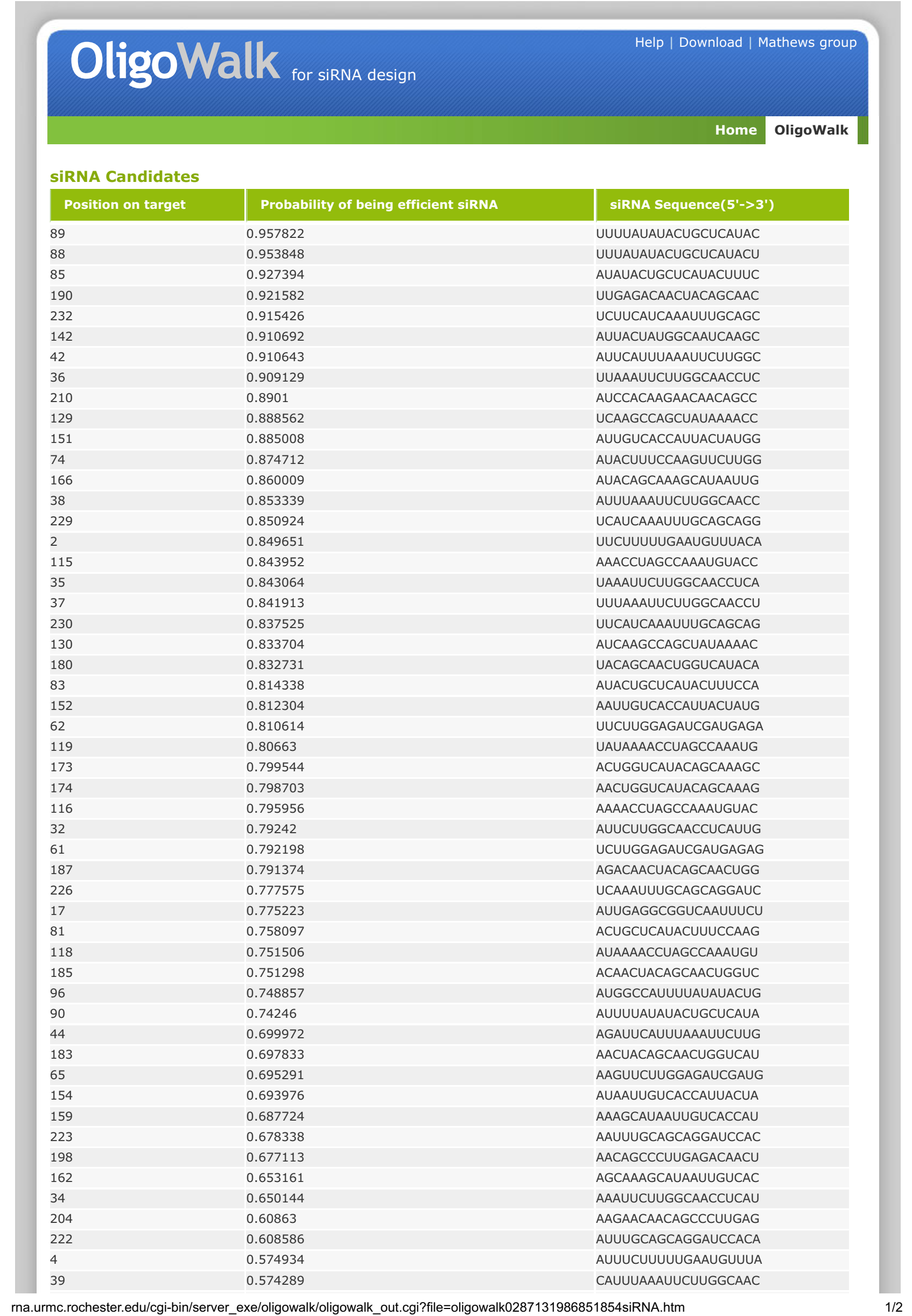


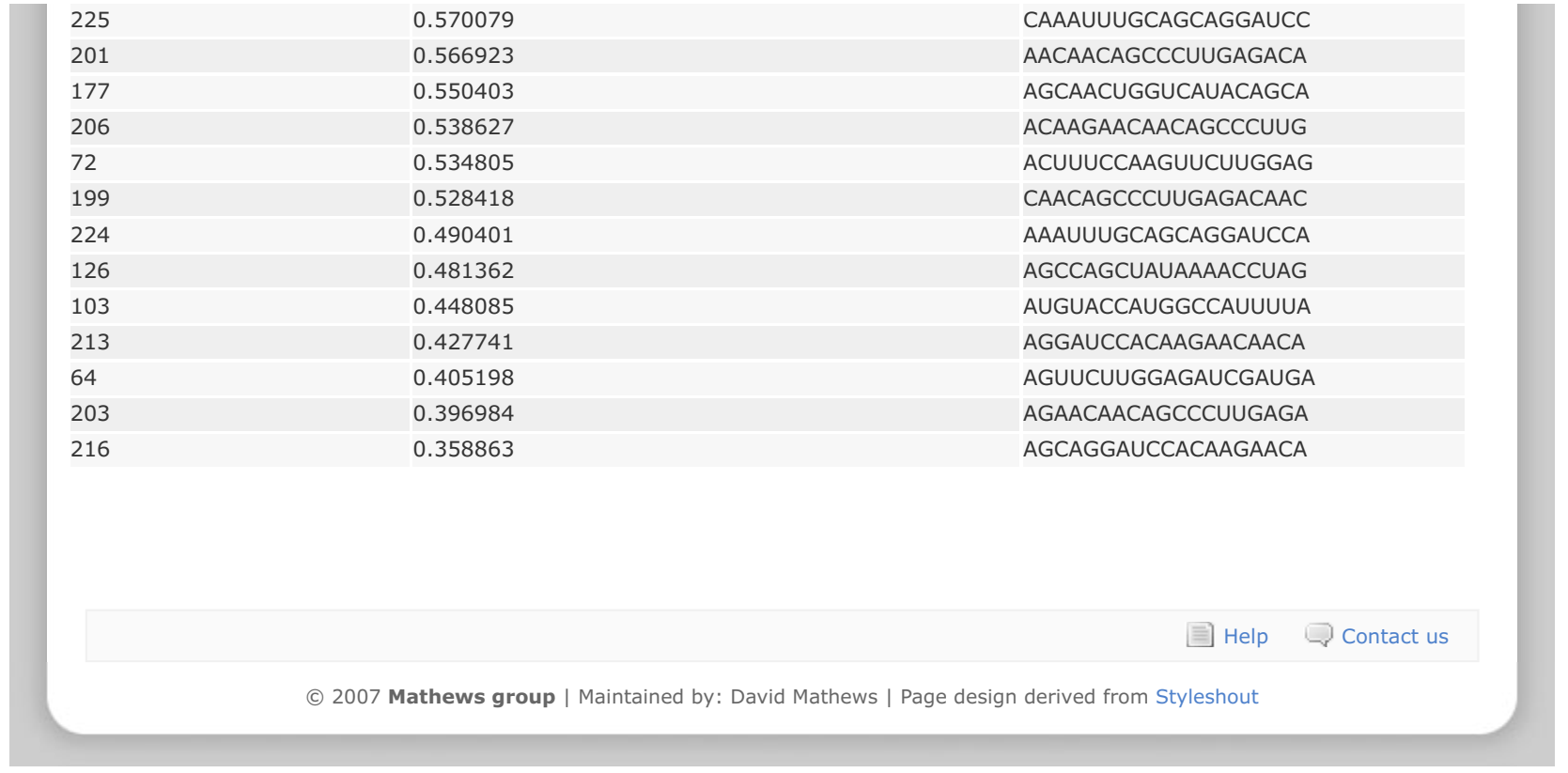

Supplement: Supplementary file 15 — Additional file 15: Supplementary Table 15. List of siRNAs predicted by OligoWalk for various conserved regions of the ‘S’ gene. [file 43141_2022_346_MOESM15_ESM.docx]
