# Supplementary material for: Design of siRNA molecules for silencing of membrane glycoprotein, nucleocapsid phosphoprotein, and surface glycoprotein genes of SARS-CoV2
Source: J Genet Eng Biotechnol. 2022 Apr 28;20:65. doi: 10.1186/s43141-022-00346-z (PMC9047631; doi:10.1186/s43141-022-00346-z)
Supplement: Supplementary file 20 — Additional file 20: Supplementary Table 20. siRNAs predicted for M gene at Step 5/ 6 and their parameters. [file 43141_2022_346_MOESM20_ESM.docx]

**Supplementary Table 20: siRNAs predicted for M gene at Step 5/ 6 and their parameters**

| **siRNA** | **Conserved Region ID** | **Target Sequence (21 + 2 nt)** | **Antisense (5’ – 3’) 21 nt** | **Sense (5’-3’) 19 nt** | **SMEpred (Efficacy)** | **Free Energy of Binding** | **Free Energy of Folding** | **Whole dG (kcal/mol)** | **% GC Content** | **siRNA Scales** | **RNAxs (Position)** | **OligoWalk (Probability value)** | **Guide (T*_m_*)** | **Passenger (T*_m_*)** | **siDirect (Position)** | ***i-Score*** |
| --- | --- | --- | --- | --- | --- | --- | --- | --- | --- | --- | --- | --- | --- | --- | --- | --- |
| M8.3 | 8 | ATCACGAACGCTTTCTTATTACA | UAAUAAGAAAGCGUUCGUGau | CACGAACGCUUUCUUAUUA | 93.3 | -30.8 | 1.7 | -31.9 | 36.8 | 8 | 116 | 0.880858 | - | - | - | 70.3 |
| M8.4 | 8 | TACATCACGAACGCTTTCTTATT | UAAGAAAGCGUUCGUGAUGua | CAUCACGAACGCUUUCUUA | 88.5 | -33.2 | 1.6 | -34.2 | 42.1 | 9 | 113 | 0.868898 | - | - | - | 67.7 |
| M8.5 | 8 | CACGAACGCTTTCTTATTACAAA | UGUAAUAAGAAAGCGUUCGug | CGAACGCUUUCUUAUUACA | 99.5 | -32.4 | 1.7 | -31.9 | 36.8 | 4 | 118 | 0.808041 | - | - | - | 75.2 |
| M8.6 | 8 | ACGAACGCTTTCTTATTACAAAT | UUGUAAUAAGAAAGCGUUCgu | GAACGCUUUCUUAUUACAA | 93.2 | -30.7 | 1.7 | -30.4 | 31.6 | 8 | 119 | 0.904307 | - | - | - | 72.2 |
| M8.9 | 8 | CTGCCTAAAGAAATCACTGTTGC | AACAGUGAUUUCUUUAGGCag | GCCUAAAGAAAUCACUGUU | 89.1 | -33.6 | 1.7 | -33.3 | 36.8 | 16 | 90 | 0.872798 | - | - | - | 66.9 |
