# Supplementary material for: Design of siRNA molecules for silencing of membrane glycoprotein, nucleocapsid phosphoprotein, and surface glycoprotein genes of SARS-CoV2
Source: J Genet Eng Biotechnol. 2022 Apr 28;20:65. doi: 10.1186/s43141-022-00346-z (PMC9047631; doi:10.1186/s43141-022-00346-z)
Supplement: Supplementary file 21 — Additional file 21: Supplementary Table 21. siRNAs predicted for N gene at Step 5/ 6 and their parameters. [file 43141_2022_346_MOESM21_ESM.docx]

**Supplementary Table 21: siRNAs predicted for N gene at Step 5/ 6 and their parameters**

| **siRNA ID** | **Conserved Region ID** | **Target Sequence (21 + 2 nt)** | **siRNA sequence (Antisense/ Guide) 21 nt** | **Sense/ Passenger (19 nt)** | **SMEpred (Efficacy)** | **Free Energy of Binding** | **Free Energy of Folding** | **Whole dG (kcal/mol)** | **% GC Content** | **siRNA Scales** | **RNAxs (Position)** | **OligoWalk (Probability value)** | **Guide (T*_m_*)** | **Passenger (T*_m_*)** | **siDirect (Position)** | ***i-Score*** |
| --- | --- | --- | --- | --- | --- | --- | --- | --- | --- | --- | --- | --- | --- | --- | --- | --- |
| N10.1 | 10 | GGCCAAACTGTCACTAAGAAATC | UUUCUUAGUGACAGUUUGGcc | CCAAACUGUCACUAAGAAA | 87.1 | -35.8 | 1.8 | -33.1 | 36.8 | 17 | 43 | 0.923617 | 11.7 | 16.7 | 23-45 | 74.5 |
| N10.3 | 10 | GCCAAACTGTCACTAAGAAATCT | AUUUCUUAGUGACAGUUUGgc | CAAACUGUCACUAAGAAAU | 85.7 | -33.1 | 1.8 | -30.9 | 31.6 | 15 | 44 | 0.828499 | 7.1 | 17.8 | 24-46 | 65.5 |
| N11.2 | 11 | ATGACAAAGATCCAAATTTCAAA | UGAAAUUUGGAUCUUUGUCau | GACAAAGAUCCAAAUUUCA | 94.5 | -30 | 1.7 | -31.1 | 31.6 | 15 | 58 | 0.78544 | 0.4 | 19.2 | 38-60 | 73.6 |
