# Supplementary material for: Design of siRNA molecules for silencing of membrane glycoprotein, nucleocapsid phosphoprotein, and surface glycoprotein genes of SARS-CoV2
Source: J Genet Eng Biotechnol. 2022 Apr 28;20:65. doi: 10.1186/s43141-022-00346-z (PMC9047631; doi:10.1186/s43141-022-00346-z)
Supplement: Supplementary file 22 — Additional file 22: Supplementary Table 22. siRNAs predicted for S gene at Step 5/ 6 and their parameters. [file 43141_2022_346_MOESM22_ESM.docx]

**Supplementary Table 22: siRNAs predicted for S gene at Step 5/ 6 and their parameters**

| **siRNA ID** | **Conserved Region ID** | **Target Sequence (21 + 2 nt)** | **siRNA sequence (Antisense/ Guide) 21 nt** | **Sense/ Passenger (19 nt)** | **SMEpred (Efficacy)** | **Free Energy of Binding** | **Free Energy of Folding** | **Whole dG (kcal/mol)** | **% GC Content** | **siRNA Scales** | **RNAxs (Position)** | **OligoWalk (Probability value)** | **Guide (T*_m_*)** | **Passenger (T*_m_*)** | **siDirect (Position)** | ***i-Score*** |
| --- | --- | --- | --- | --- | --- | --- | --- | --- | --- | --- | --- | --- | --- | --- | --- | --- |
| S1.1 | 1 | CTGCATACACTAATTCTTTCACA | UGAAAGAAUUAGUGUAUGCag | GCAUACACUAAUUCUUUCA | 85.9 | -31.6 | 1.8 | -31.5 | 31.6 | 13 | 41 | 0.934623 | 14.8 | 20.4 | 21-43 | 69.9 |
| S6.6 | 6 | AAGGAAAACAGGGTAATTTCAAA | UGAAAUUACCCUGUUUUCCuu | GGAAAACAGGGUAAUUUCA | 91.3 | -31.9 | 1.5 | -33.2 | 36.8 | 10 | 87 | 0.836603 | 2.1 | 14.9 | 67-89 | 76.9 |
| S10.3 | 10 | AAGGAATCTATCAAACTTCTAAC | UAGAAGUUUGAUAGAUUCCuu | GGAAUCUAUCAAACUUCUA | 100.7 | -30.6 | 1.5 | -31.9 | 31.6 | 7 | 67 | 0.90315 | 17.7 | 16 | 47-69 | 79 |
| S14.3 | 14 | TTGGAATTCTAACAATCTTGATT | UCAAGAUUGUUAGAAUUCCaa | GGAAUUCUAACAAUCUUGA | 87.4 | -30 | 1.6 | -31.5 | 31.6 | 13 | 74 | 0.847291 | 12 | 14.8 | 54-76 | 72.3 |
| S28.5 | 28 | AGCTGTTGAACAAGACAAAAACA | UUUUUGUCUUGUUCAACAGcu | CUGUUGAACAAGACAAAAA | 97.3 | -31.2 | 1.6 | -30.3 | 31.6 | 11 | 113 | 0.893136 | 14.9 | 20.5 | 93-115 | 72.7 |
| S28.9 | 28 | ACTGGAATAGCTGTTGAACAAGA | UUGUUCAACAGCUAUUCCAgu | UGGAAUAGCUGUUGAACAA | 95.9 | -34.5 | 1.7 | -34.5 | 36.8 | 12 | 105 | 0.879994 | 20.5 | 19.9 | 85-107 | 70.2 |
| S28.12 | 28 | TGGCAGTTTTTGTACACAATTAA | AAUUGUGUACAAAAACUGCca | GCAGUUUUUGUACACAAUU | 89.3 | -31.5 | 1.7 | -30.5 | 31.6 | 18 | 71 | 0.928609 | 19.3 | 10.3 | 51-73 | 69.7 |
| S30.1 | 30 | CTCATTTGTGCACAAAAGTTTAA | AAACUUUUGUGCACAAAUGag | CAUUUGUGCACAAAAGUUU | 90.1 | -30.6 | 1.6 | -30 | 31.6 | 9 | 32 | 0.728615 | 3.2 | 12.1 | 12-34' | 68.5 |
| S31.1 | 31 | TGGCTTATAGGTTTAATGGTATT | UACCAUUAAACCUAUAAGCca | GCUUAUAGGUUUAAUGGUA | 93.3 | -32.6 | 1.8 | -31.7 | 31.6 | 4 | 63 | 0.945826 | 20 | 2.8 | 43-65 | 79.2 |
| S31.2 | 31 | ATGCAAATGGCTTATAGGTTTAA | AAACCUAUAAGCCAUUUGCau | GCAAAUGGCUUAUAGGUUU | 95.4 | -32.1 | 1.9 | -33.3 | 36.8 | 11 | 56 | 0.885647 | 18.5 | 17.7 | 36-58 | 71.9 |
| S33.4 | 33 | AGCTTTAAACACGCTTGTTAAAC | UUAACAAGCGUGUUUAAAGcu | CUUUAAACACGCUUGUUAA | 93.8 | -30.9 | 1.7 | -29.7 | 31.6 | 10 | 21 | 0.907037 | 11.8 | 0 | 1-23' | 74.6 |
| S34.1 | 34 | GCTAATCTTGCTGCTACTAAAAT | UUUAGUAGCAGCAAGAUUAgc | UAAUCUUGCUGCUACUAAA | 92.1 | -34.3 | 1.8 | -32.6 | 31.6 | 13 | 140 | 0.836742 | 11.3 | 12 | 120-142 | 71.1 |
| S34.2 | 34 | GACATATGTGACTCAACAATTAA | AAUUGUUGAGUCACAUAUGuc | CAUAUGUGACUCAACAAUU | 86.3 | -32 | 1.7 | -31.3 | 31.6 | 16 | 91 | 0.808312 | 12.1 | 13.3 | 71-93 | 67.4 |
