# Supplementary material for: Design of siRNA molecules for silencing of membrane glycoprotein, nucleocapsid phosphoprotein, and surface glycoprotein genes of SARS-CoV2
Source: J Genet Eng Biotechnol. 2022 Apr 28;20:65. doi: 10.1186/s43141-022-00346-z (PMC9047631; doi:10.1186/s43141-022-00346-z)
Supplement: Supplementary file 23 — Additional file 23: Supplementary Table 23. List of interacting residues of human AGO2 protein with the nucleotides of the guide strands of siRNAs of M, N & S genes within 5.0 Å. [file 43141_2022_346_MOESM23_ESM.docx]

**Supplementary Table 23:** List of interacting residues of human AGO2 protein with the nucleotides of the guide strands of siRNAs of M, N & S genes within 5.0 Å

(Note: Interacting nucleotides are not given in the table)

| **Sr. No.** | **siRNA ID** | **Docking score** | **Interacting residues** |
| --- | --- | --- | --- |
| 1. | S10.3 | -326.15 | LYS67, ARG70, ARG99, PHE158, GLN162, PRO178, VAL179, GLY180, ARG181, ARG353, ILE355, PRO604, ALA605, GLY606, GLY608, LYS 609, LYS610, PRO611, ARG632, VAL633, GLN634, GLN635, HIS636, ARG637, GLN642, ASP643, LEU644, ALA645, ALA646, MET647, GLY676, GLN677, GLN679, GLN680, HIS684, LEU687, ALA688, GLU691, ARG839, HIS841, ALA845 |
| 2. | S28.5 | -356.13 | TYR23, GLU60, GLU66, LYS67, CYS68, PRO69, ARG70, ARG71, VAL72, ASN73, GLU75, ARG99, LYS100, ASN101, ARG128, GLN162, LYS268, ILE271, HIS273, CYS274, GLN276, MET277, LYS278, ARG279, LYS280, TYR281, ARG282, PHE296, PRO297, TYR313, PHE314, ARG317, HIS318, GLN334, GLU335, HIS338, THR339, TYR340, LEU341, GLN638, GLN642, GLY676, GLN679, GLN680, HIS683, HIS684, LEU687, SER833 |
| 1. | N11.2 | -347.70 | SER182, HIS205, ASP220, VAL221, SER222, ALA223, LYS357, ASP360, ASN361, THR363, SER364, MET366, ILE367, THR370, ALA371, ARG372, ARG377, GLY526, LYS527, GLN550, LYS552, THR558, GLN560, THR561, ASN564, THR601, HIS602, PRO603, PRO604, LYS711, ARG712, HIS714, ARG716, HIS755, ALA756, GLY757, ILE758, GLN759, GLY760, THR761, SER762, ARG763, CYS795, ARG797, SER798, HIS809, PHE813, ARG816, TYR817 |
| 2. | N10.1 | -314.82 | GLU124, GLY125, LYS126, LYS265, HIS273, CYS274, MET277, ARG279, TYR281, CYS284, TYR313, ARG317, HIS318, GLN334, GLU335, GLN336, LYS337, ILE355, PRO604, ALA605, GLY606, ASP607, GLY608, LYS609, ARG632, GLN635, HIS636, ARG637, GLN638, GLU639, ILE640, GLN642, GLN677, GLN680, HIS684, HIS841, GLN842, ALA843, ALA845, LYS846, VAL848, GLN849 |
| 1. | M8.5 | -309.84 | SER364, ILE367, VAL436, TRP437, ASP438, ARG440, GLN550, MET551, LYS552, ASN553, GLN555, ARG556, THR558, PRO559, GLN560, THR561, ASN564, LEU565, LYS568, ILE758, TYR792, ARG794, CYS795, THR796, ARG797, TYR806, LEU810, PHE813 |
| 2. | M8.3 | -350.68 | LYS357, LEU358, ASP360, THR363, VAL436, TRP437, ARG440, GLN475, LYS478, ILE479, ASP482, ARG556, THR557, THR558, PRO559, GLN560, THR561, ASN564, ASP599, VAL600, THR601, HIS602, ASP671, GLY672, GLN710, LYS711, ARG712, HIS713, ILE758, ARG763, TYR792, ARG794, CYS795, THR796, ARG797, SER798, VAL799, SER800, TYR806, HIS809, LEU810, PHE813, ARG816 |
