# Supplementary material for: Design of siRNA molecules for silencing of membrane glycoprotein, nucleocapsid phosphoprotein, and surface glycoprotein genes of SARS-CoV2
Source: J Genet Eng Biotechnol. 2022 Apr 28;20:65. doi: 10.1186/s43141-022-00346-z (PMC9047631; doi:10.1186/s43141-022-00346-z)

**Supplementary Figures 1a-1c**

**Structures of guide strands of siRNAs of M, N & S genes and their energy values**

*******

**Supplementary Figures 1a: Structures of guide strands of siRNAs of M gene and their energy values**


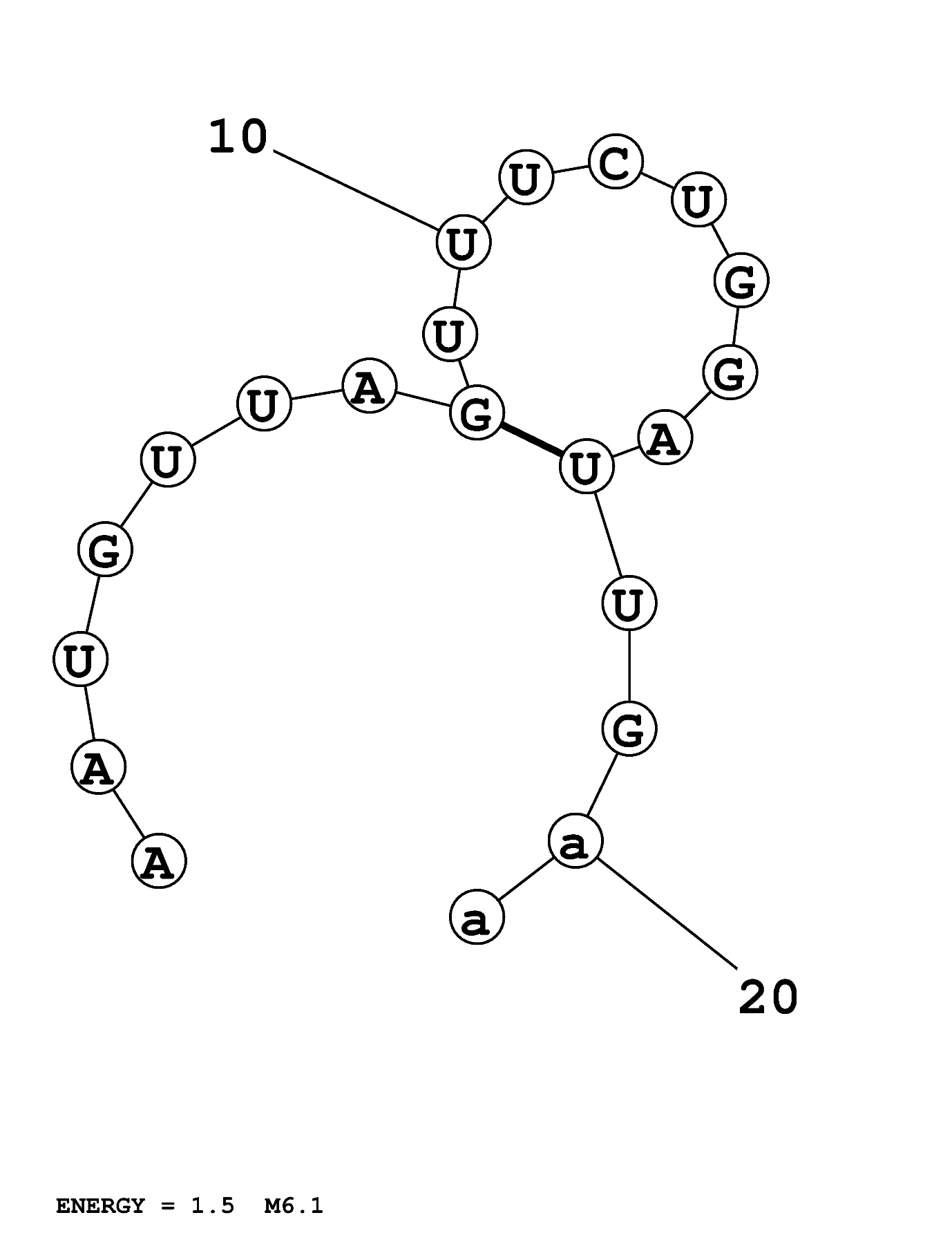


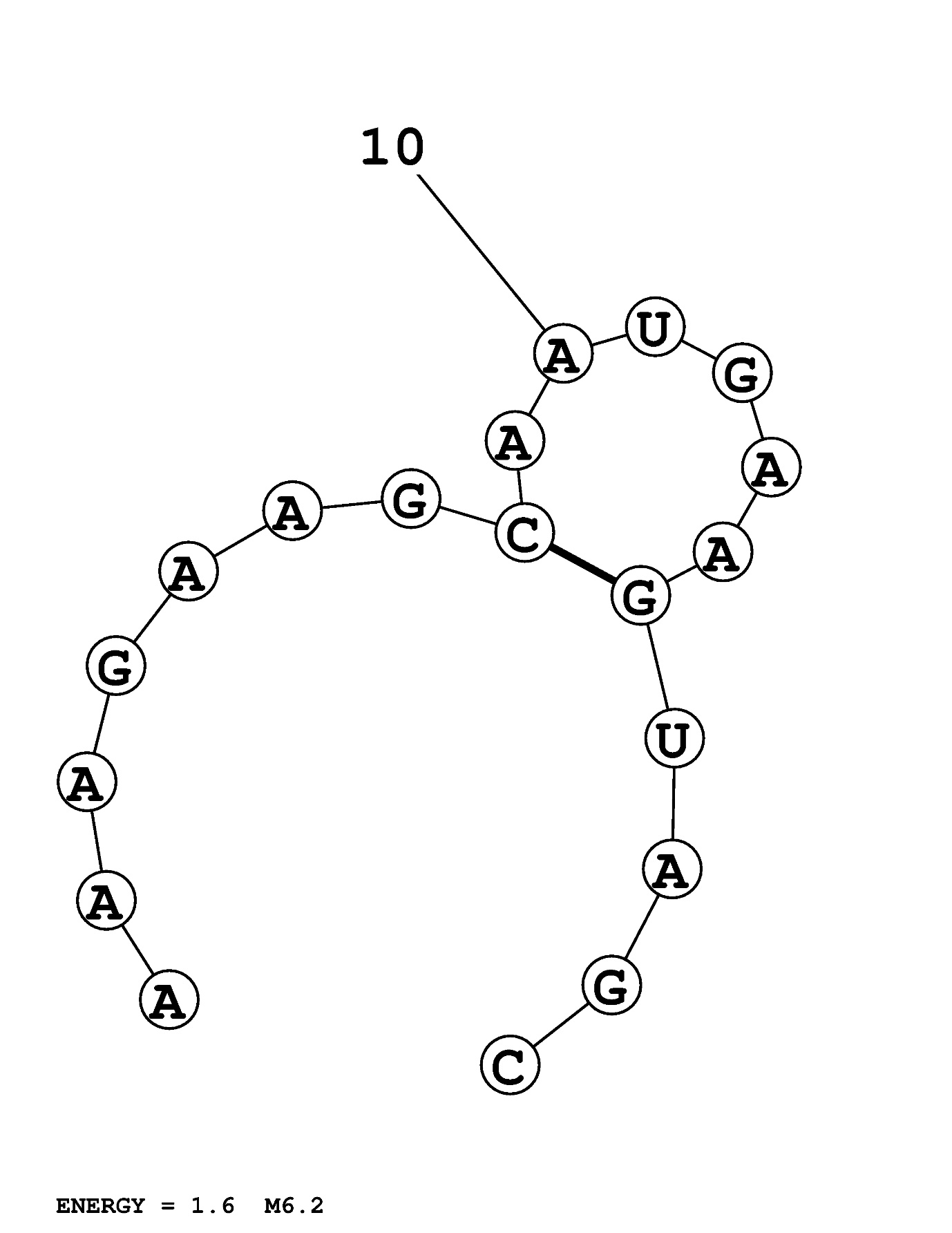


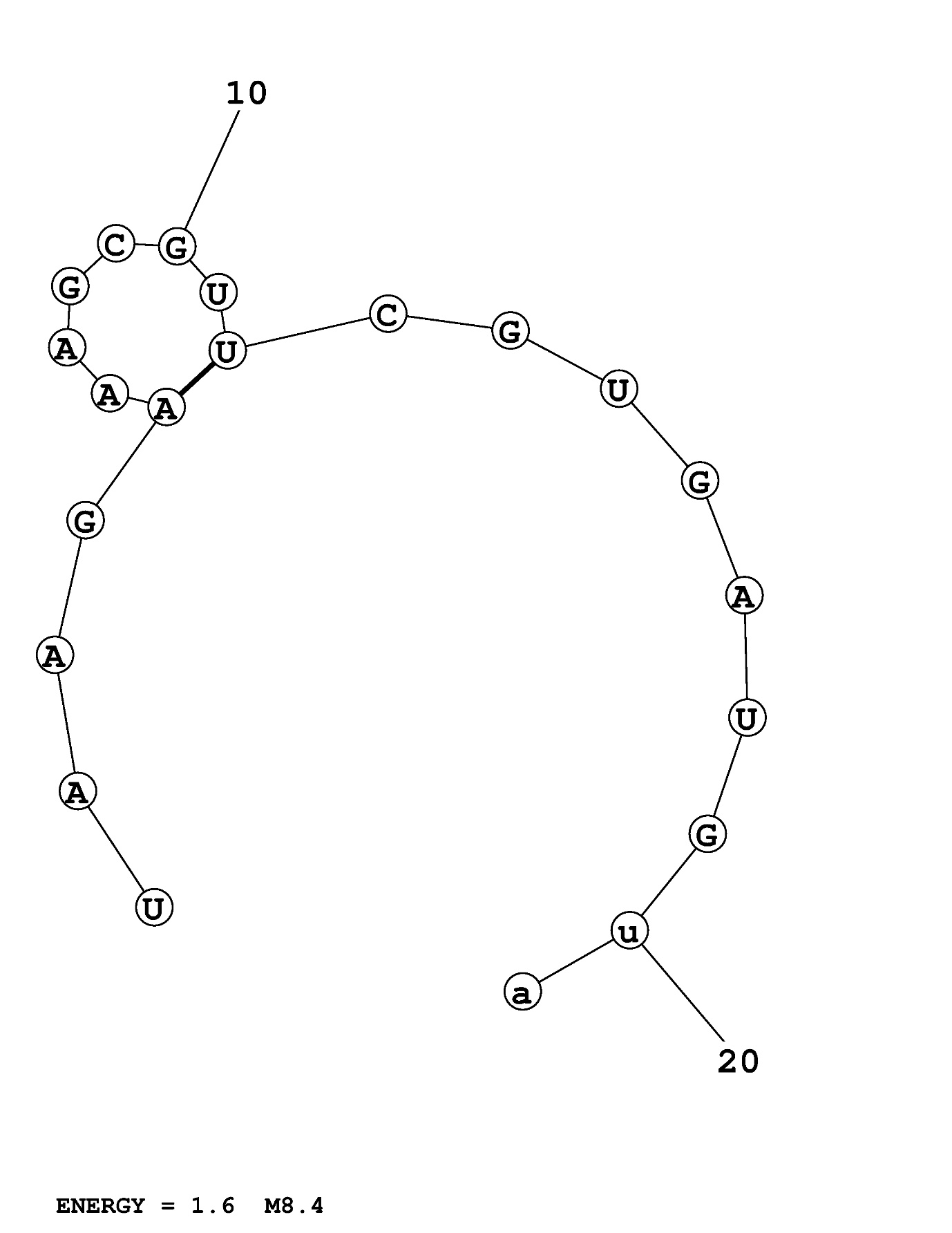


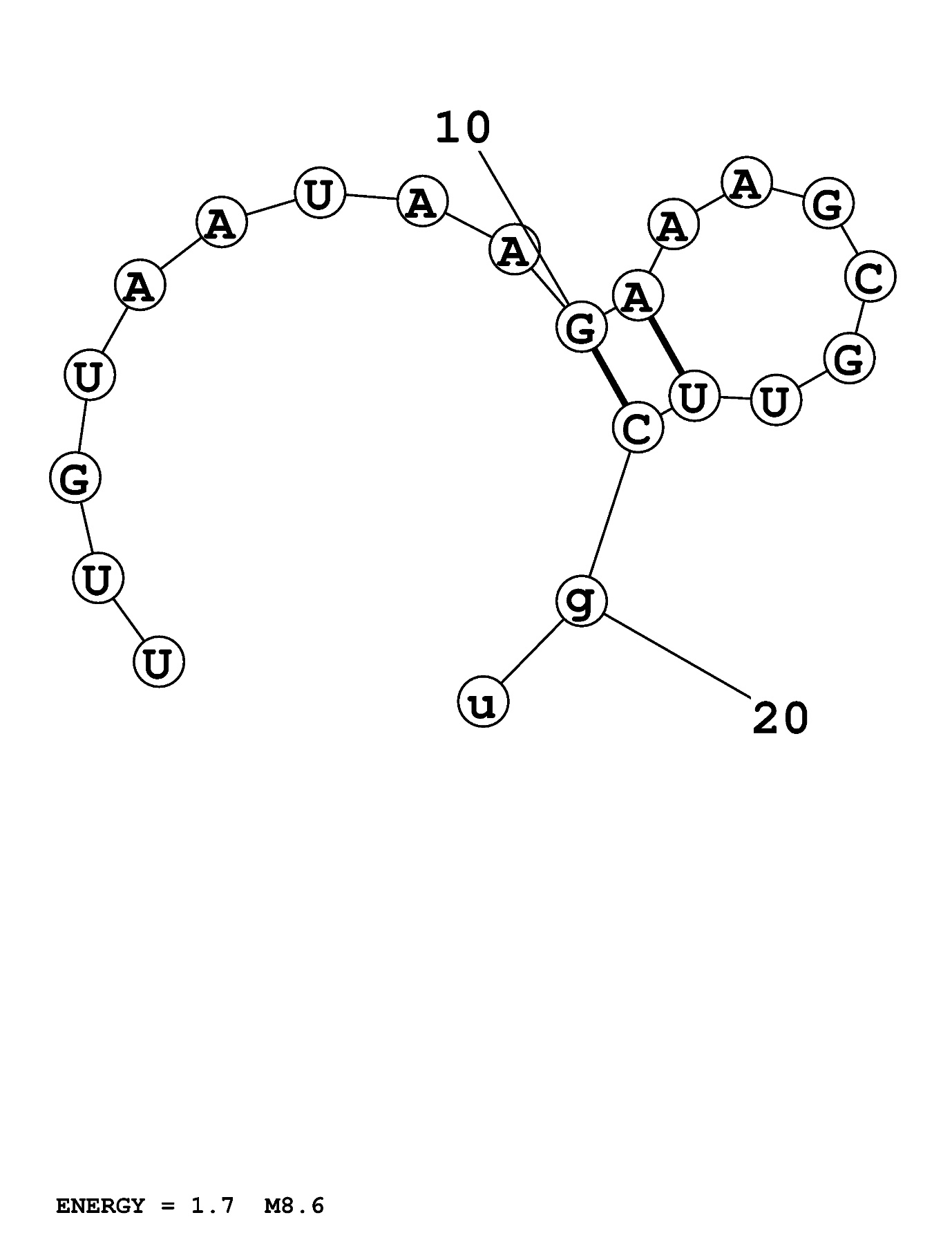


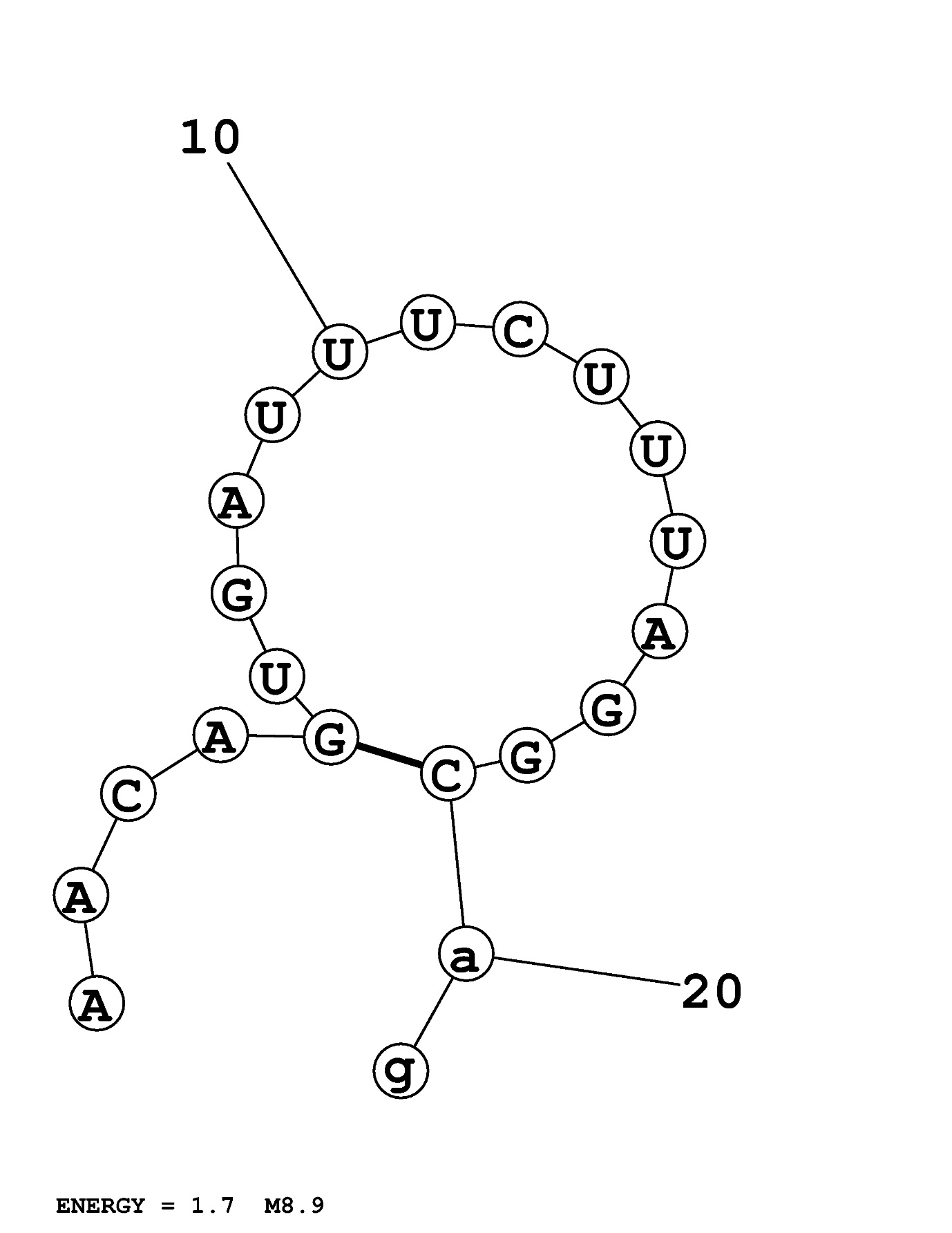


**Supplementary Figures 1b: Structures of guide strands of siRNAs of N gene and their energy values**


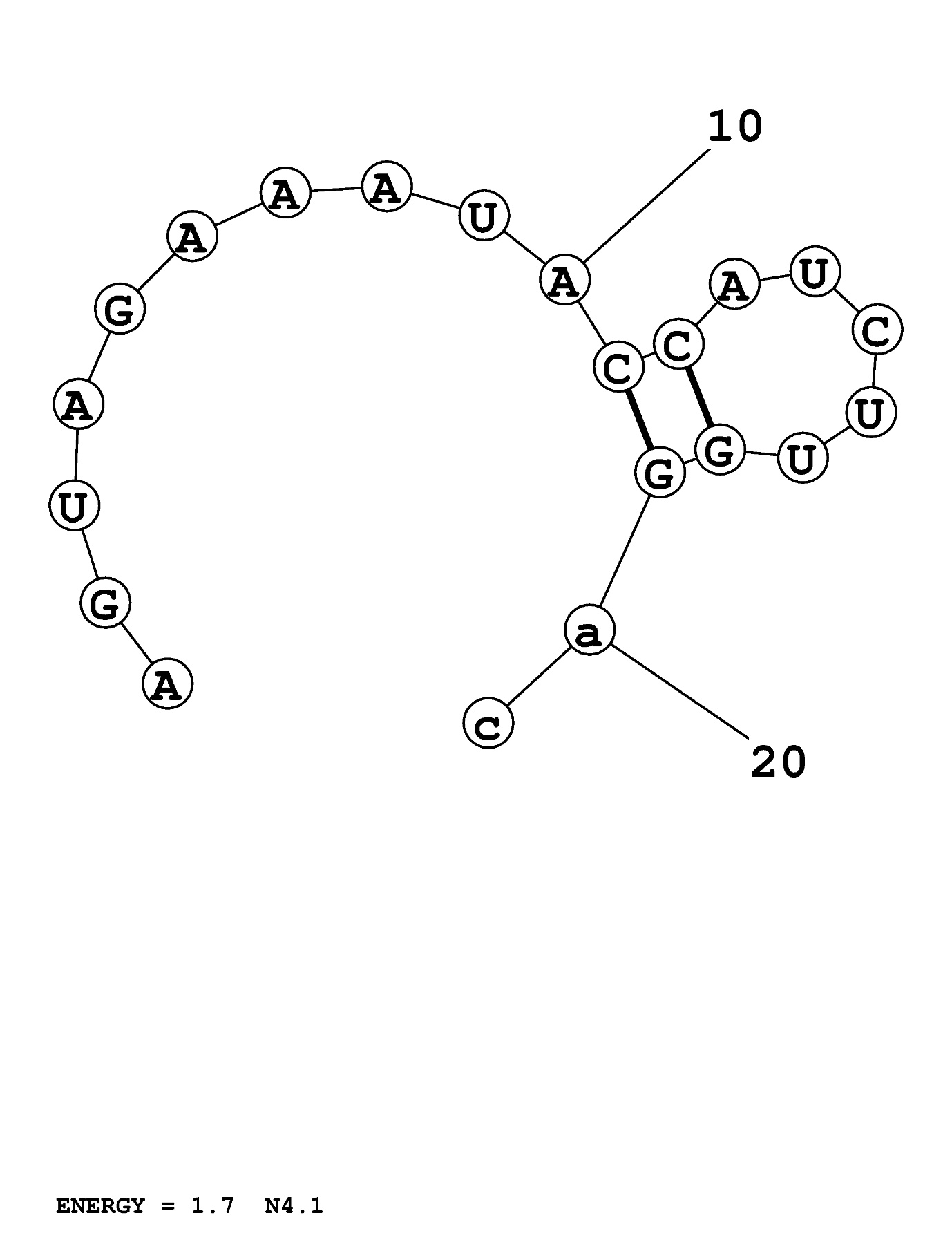


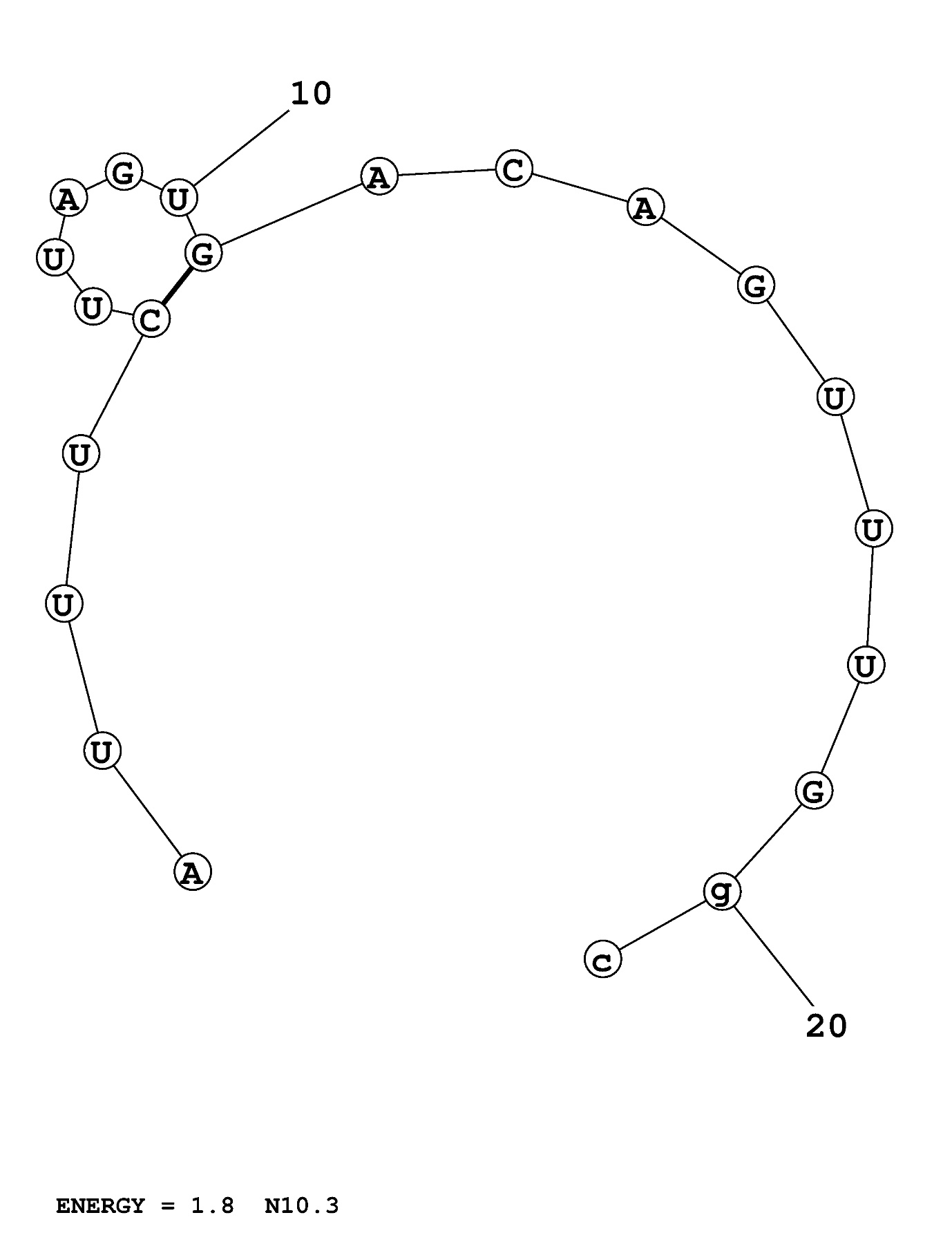


**Supplementary Figures 1c: Structures of guide strands of siRNAs of S gene and their energy values**


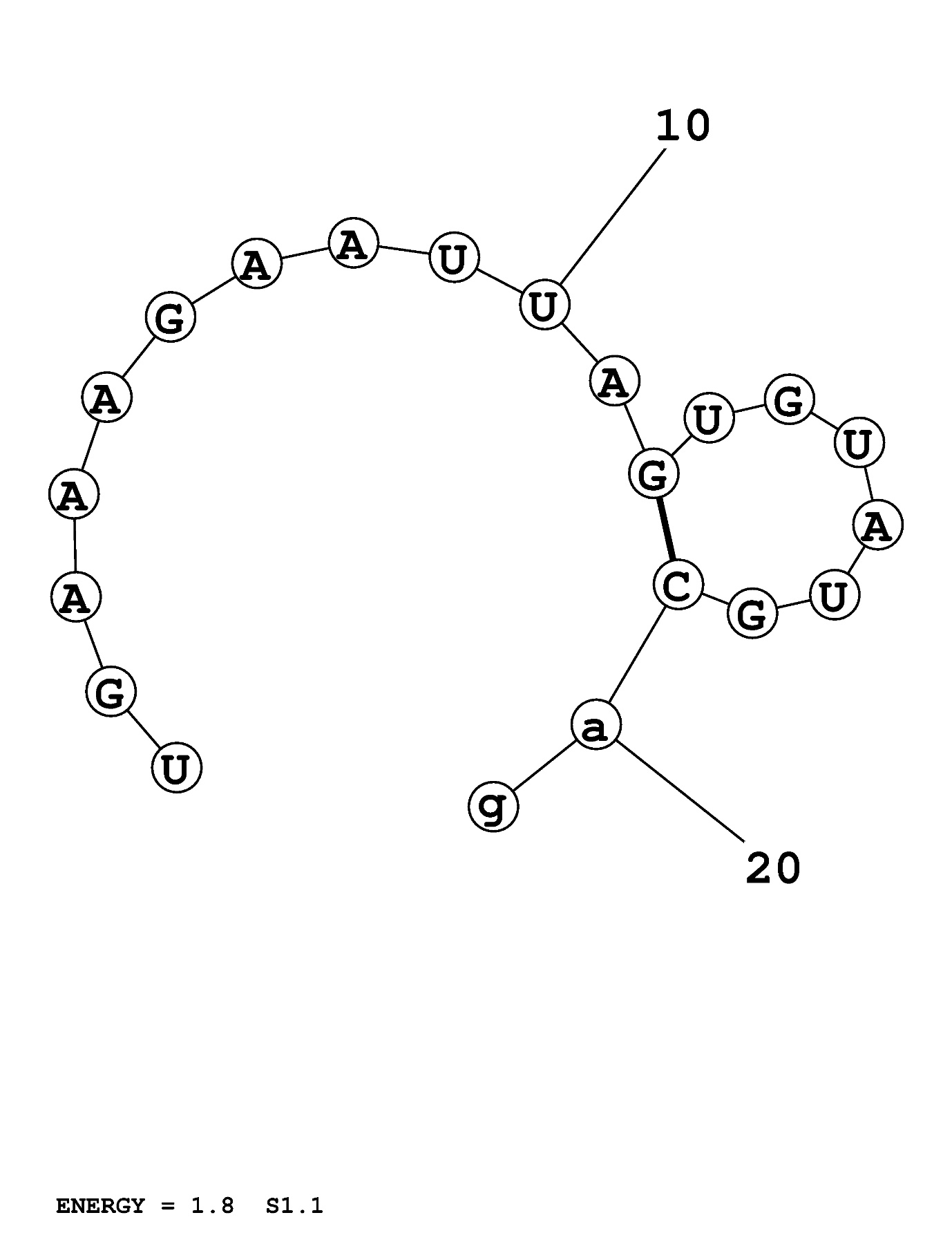


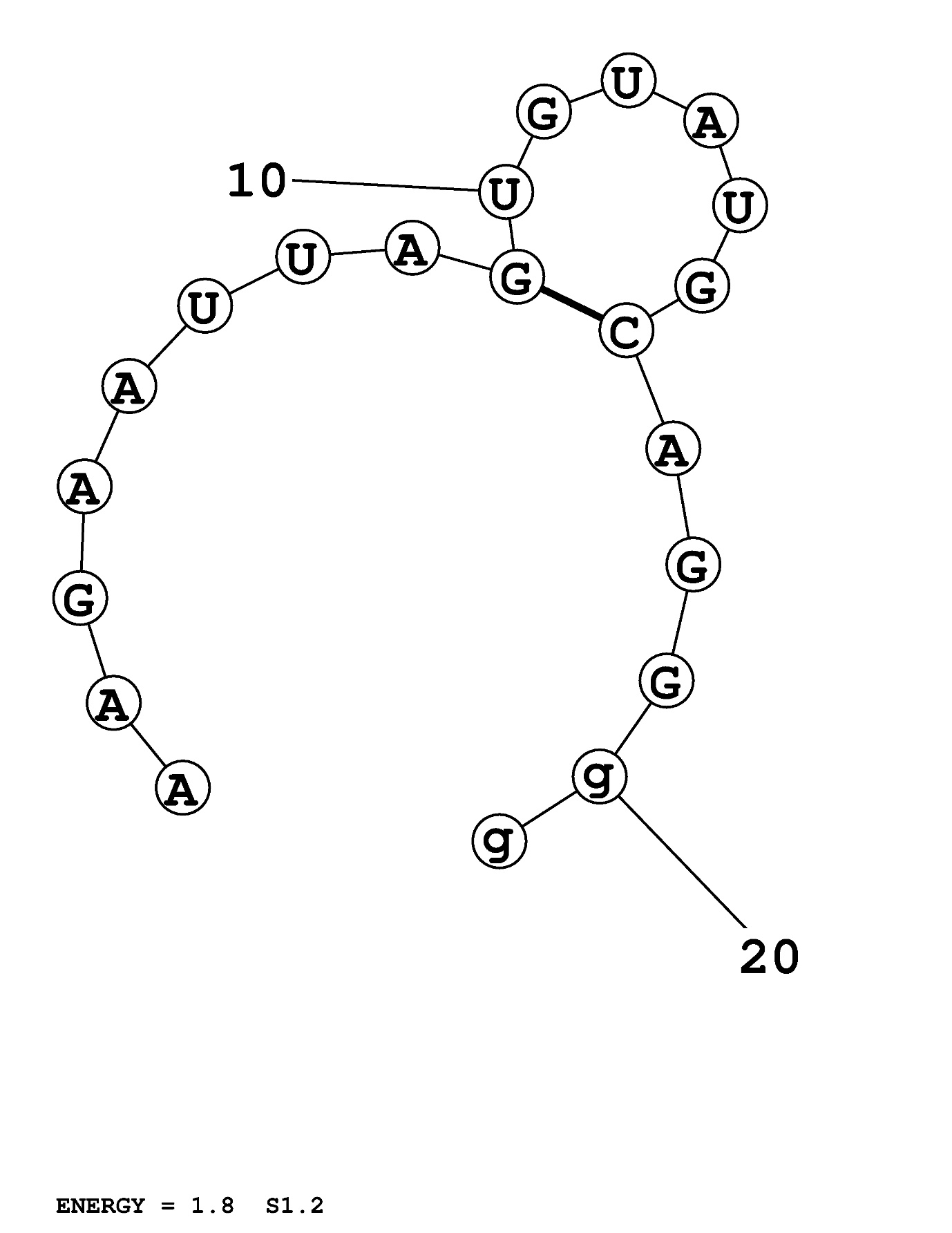


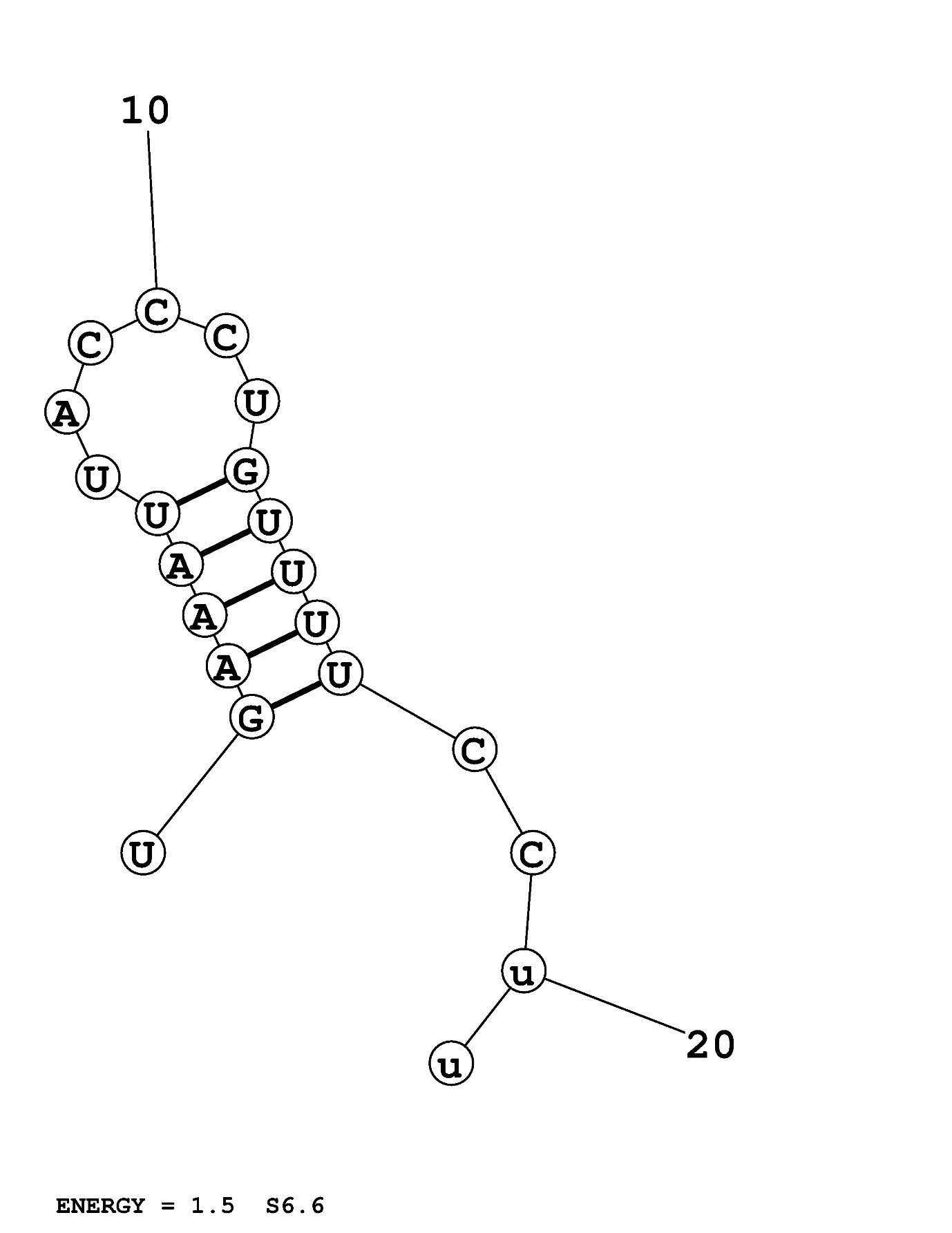


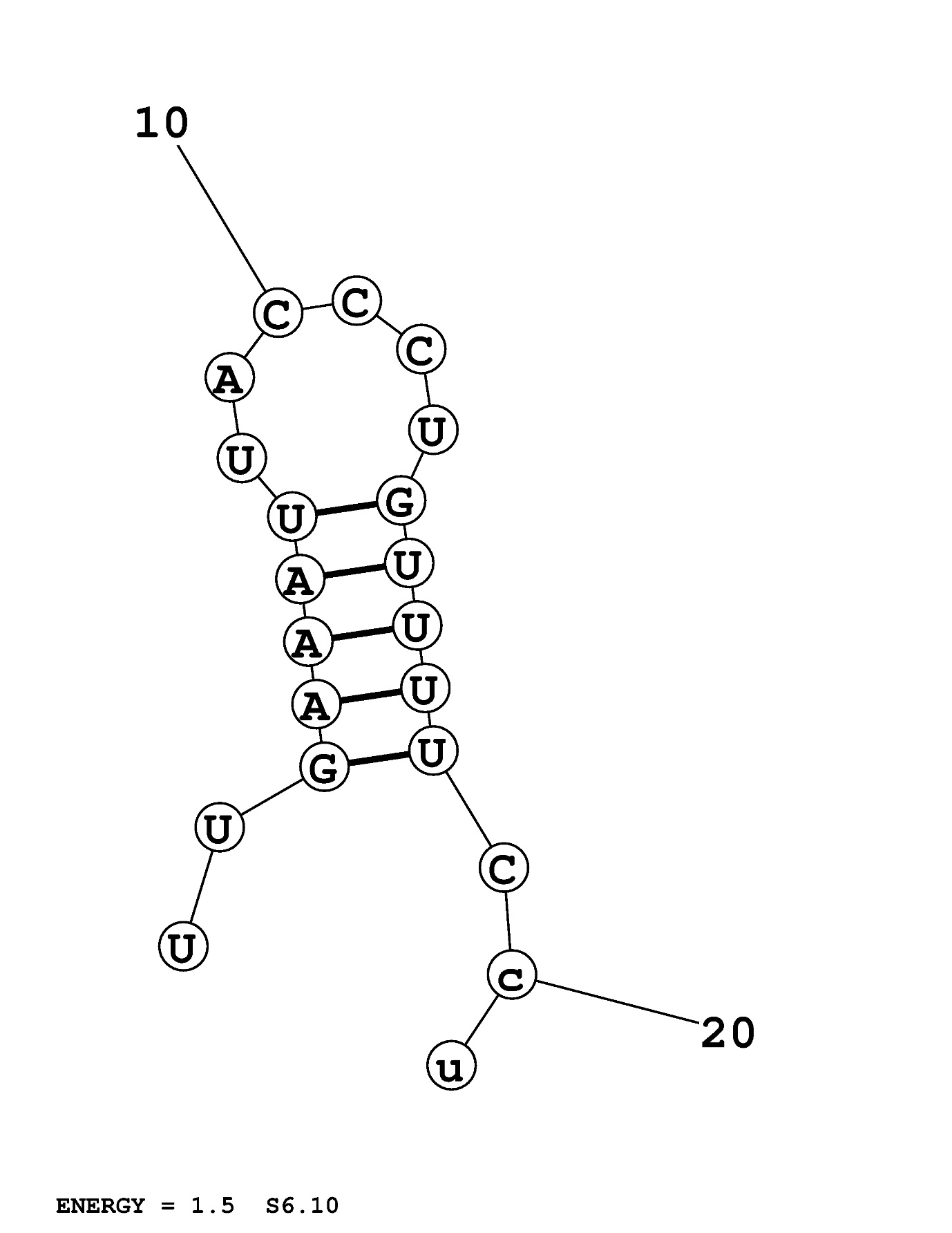


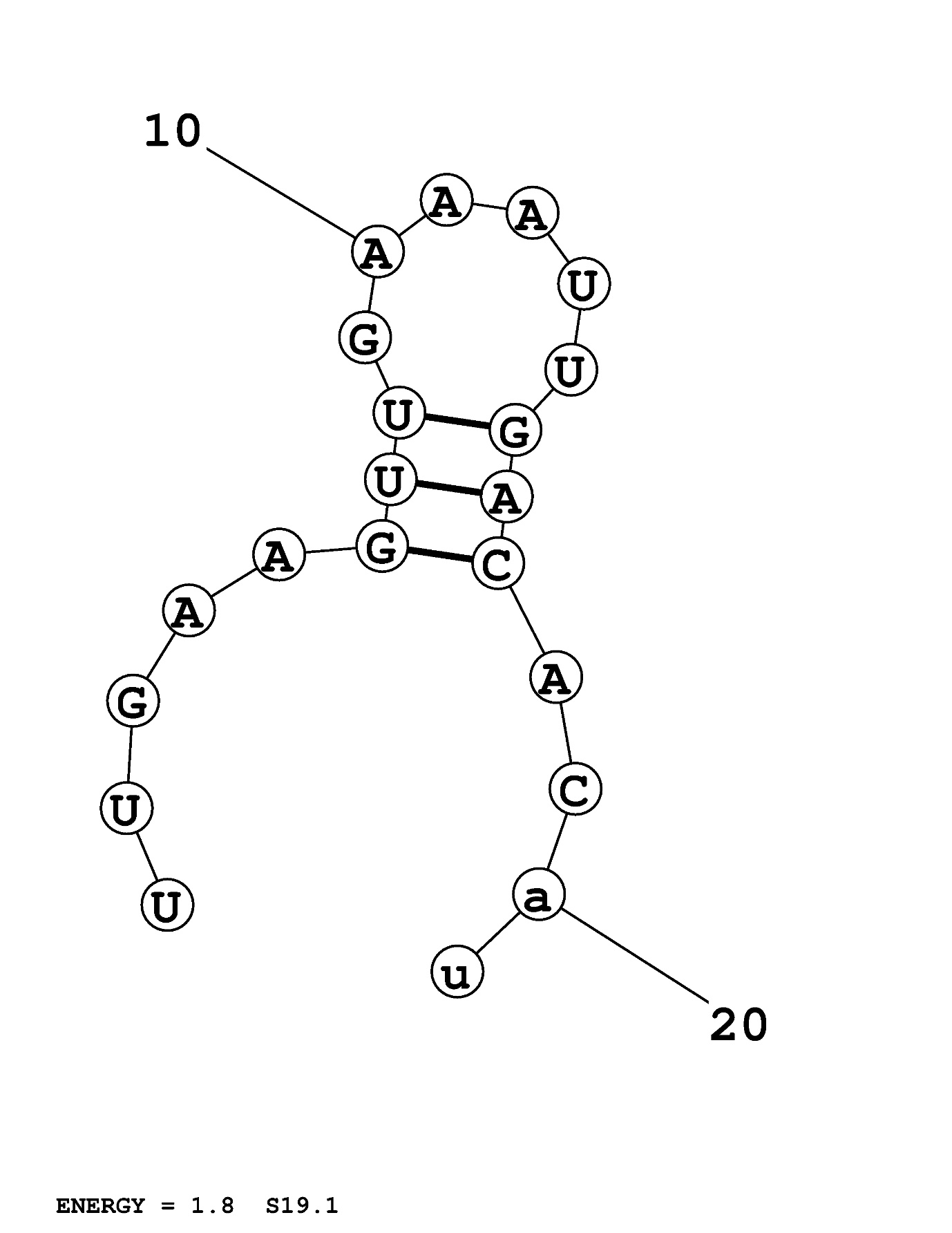


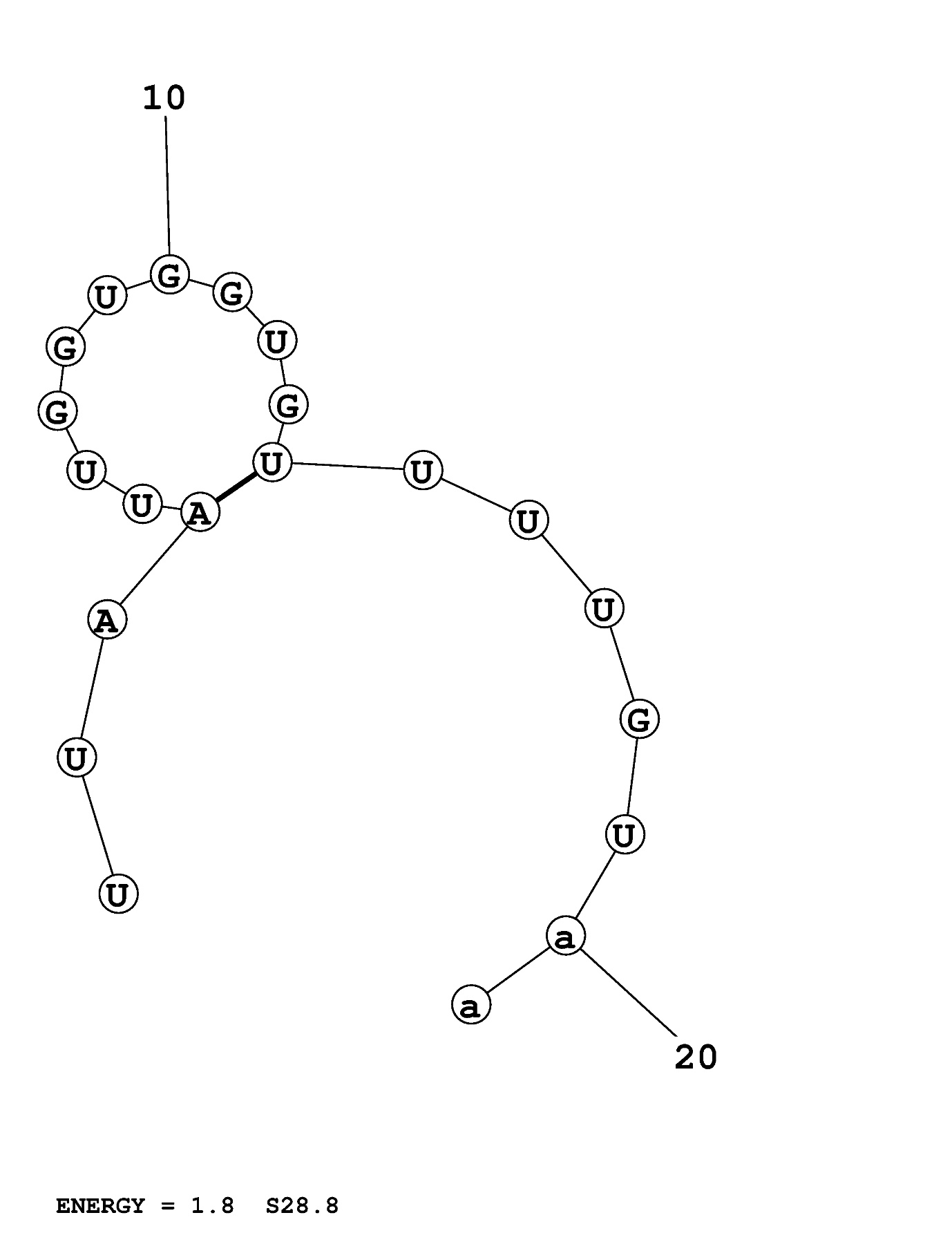


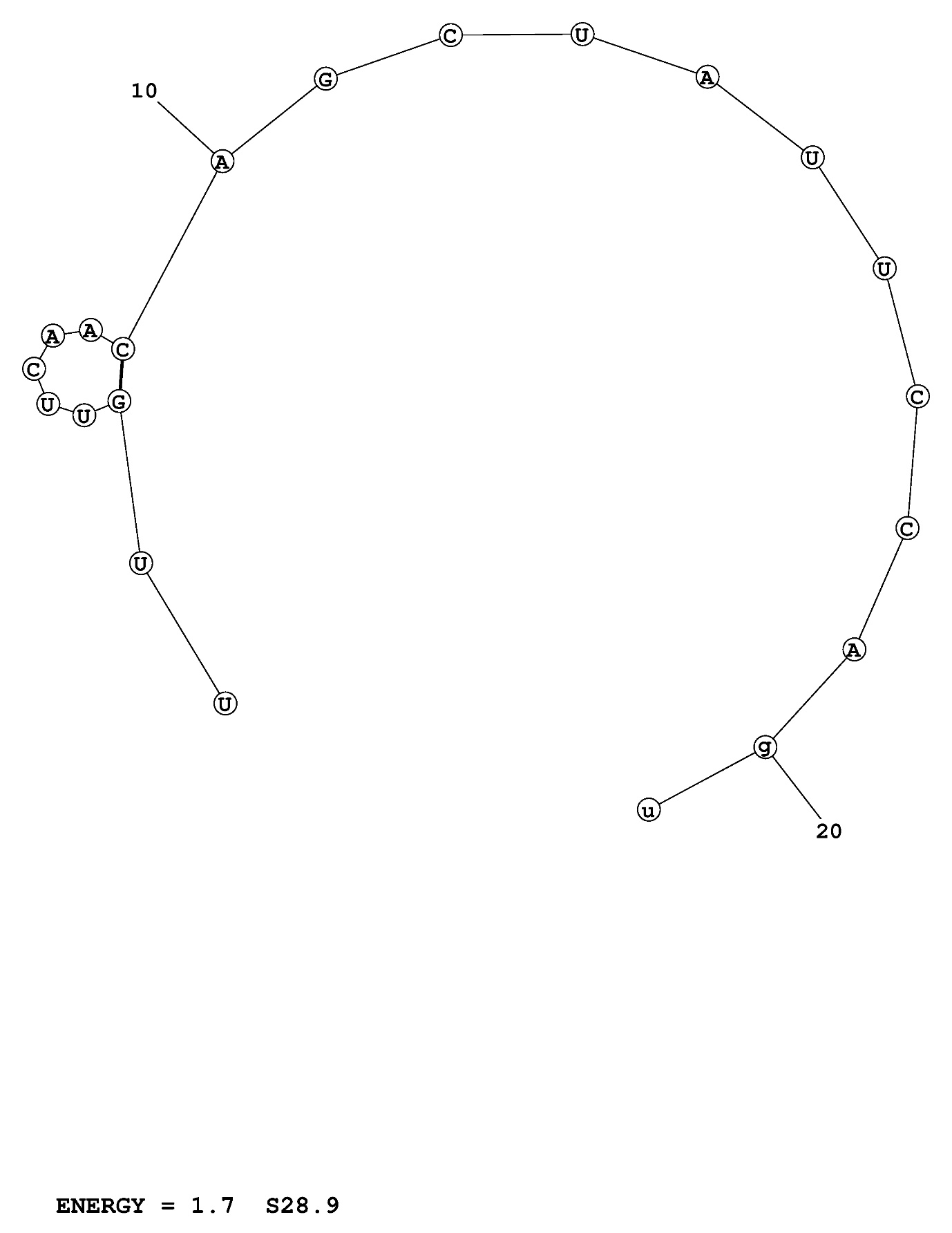


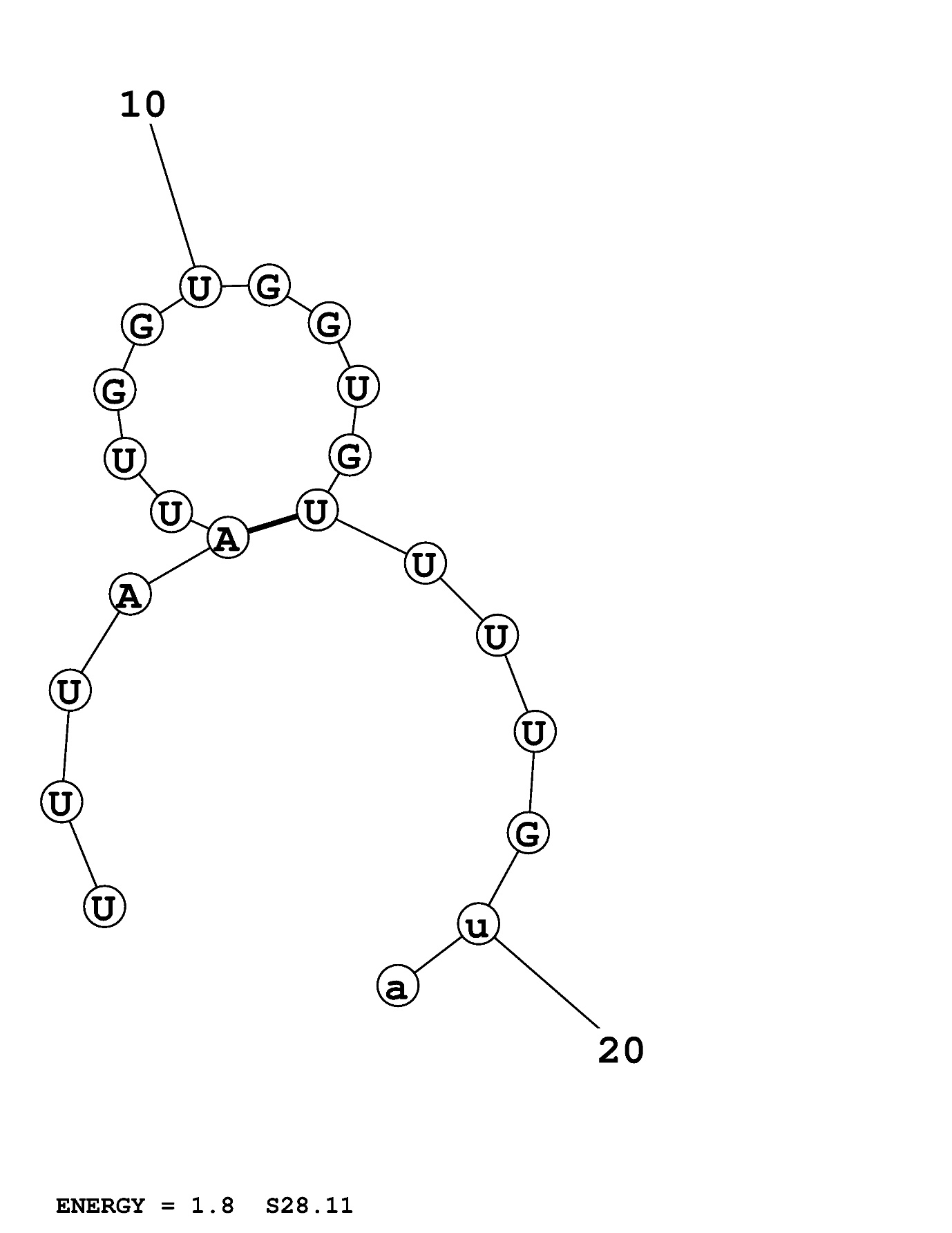


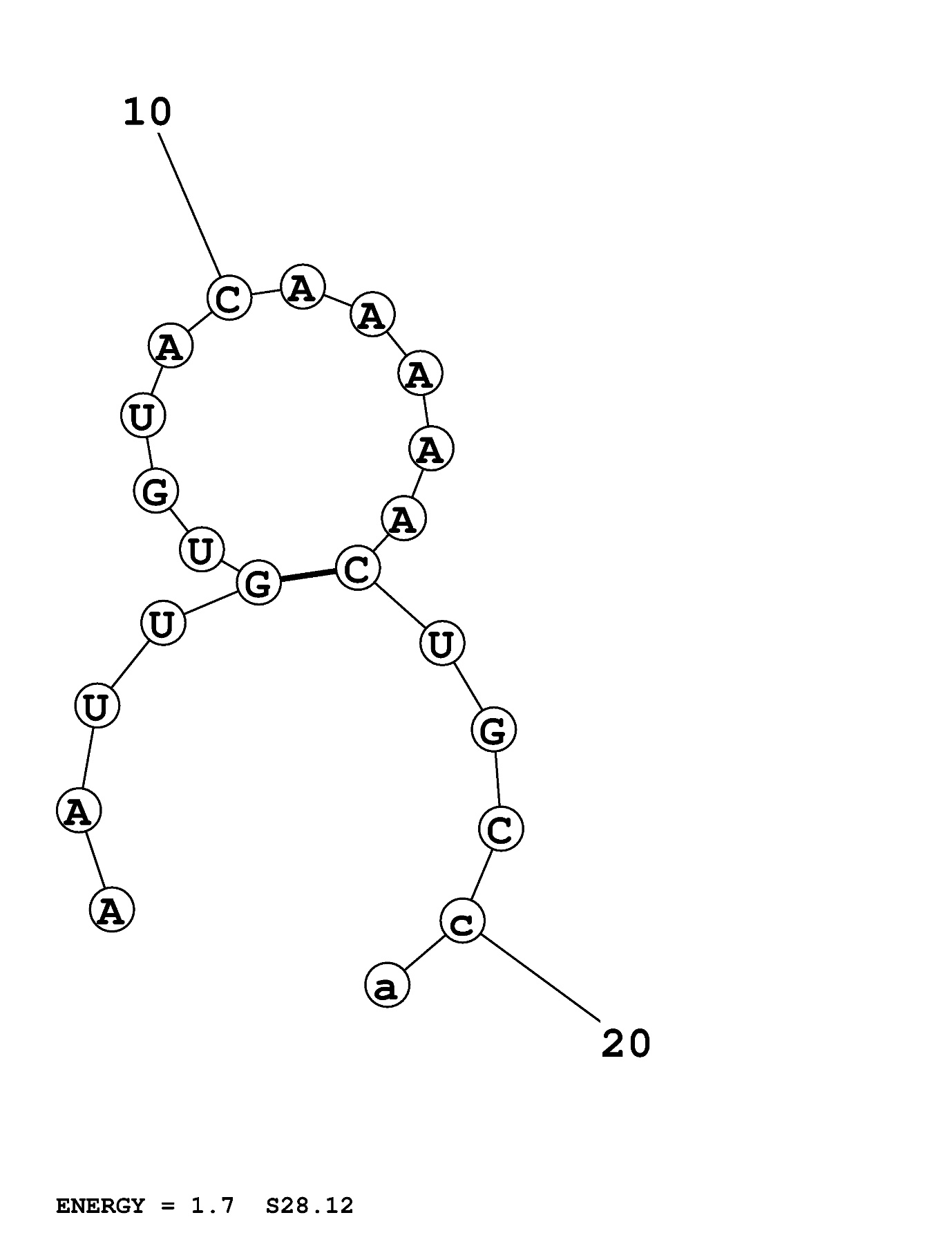


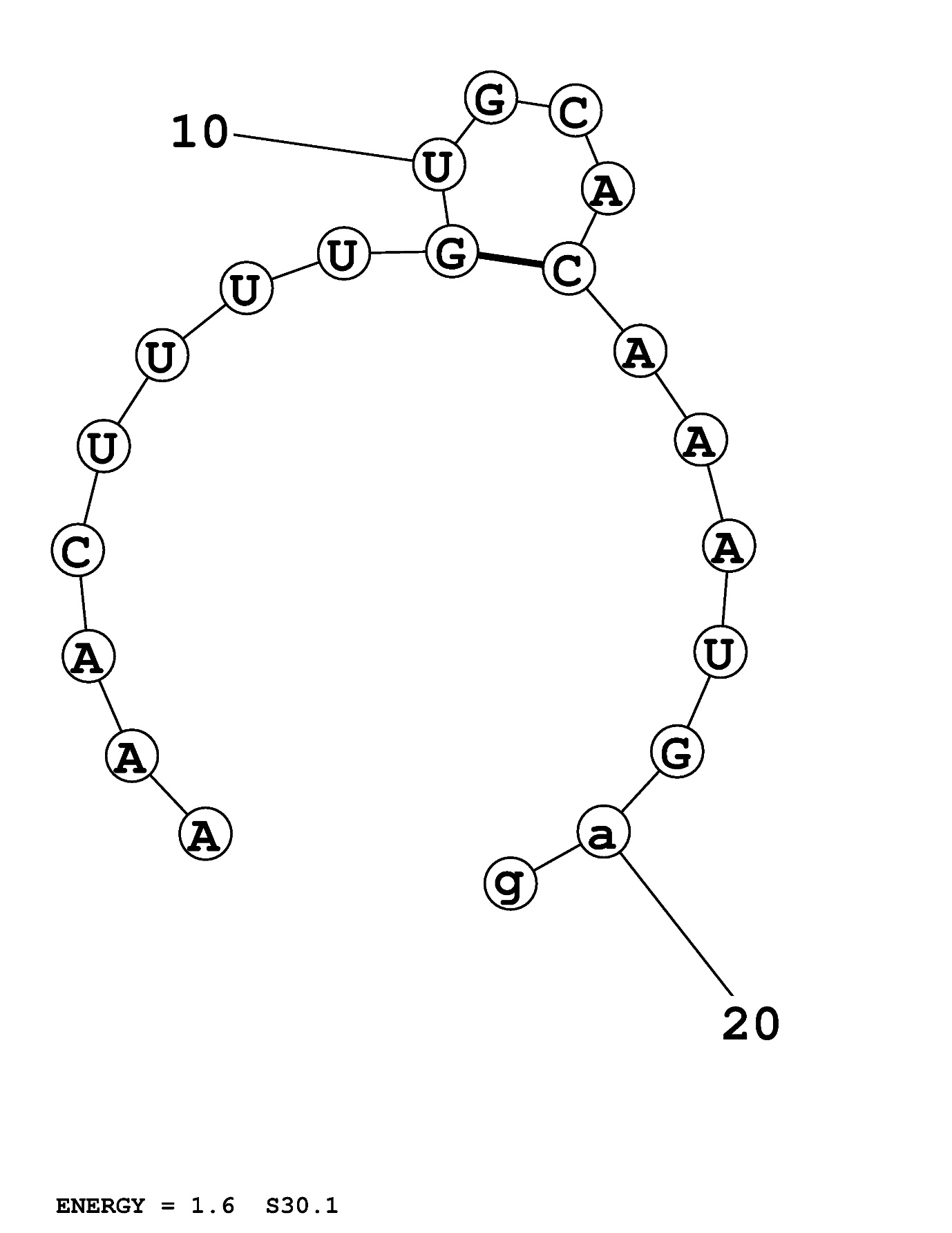


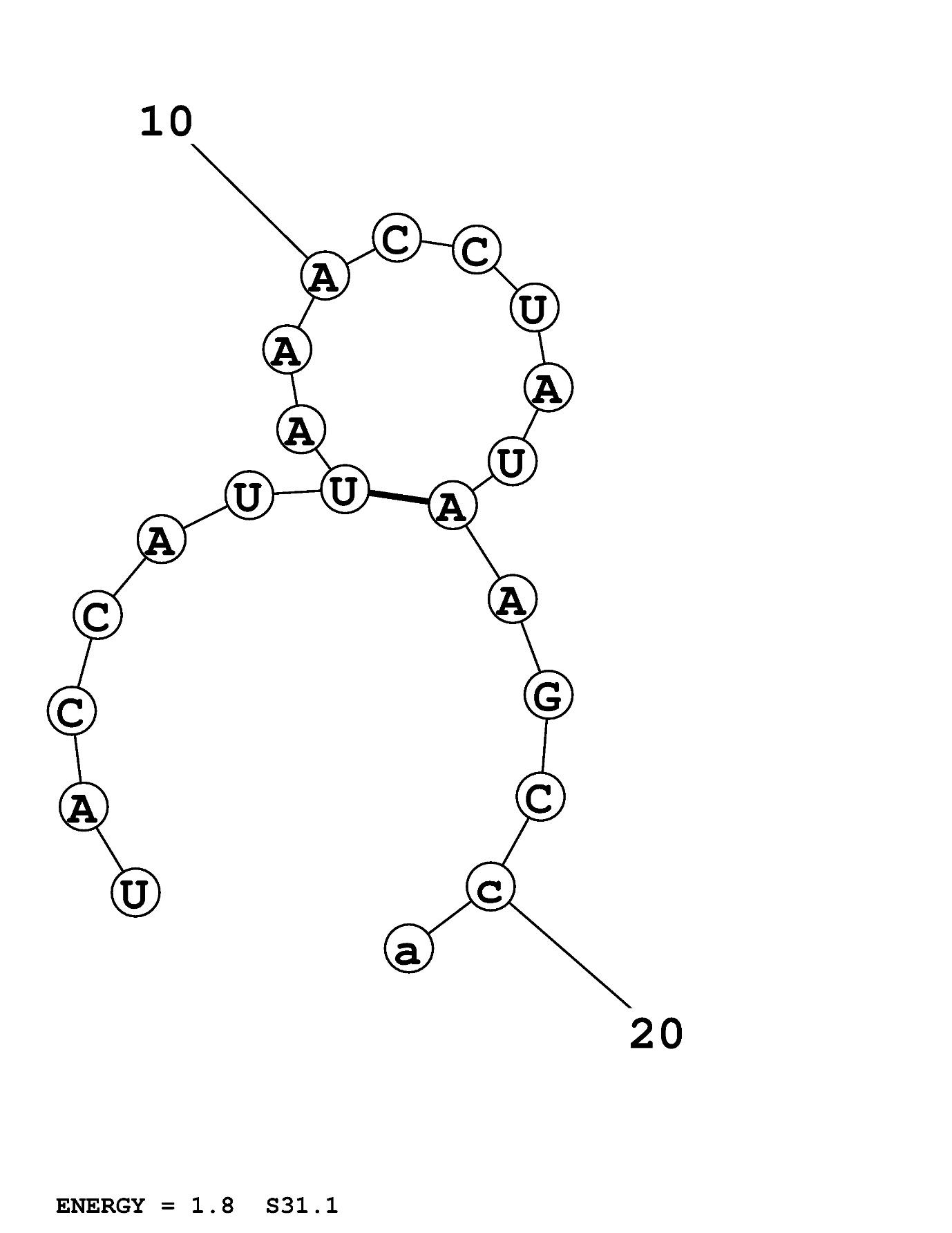


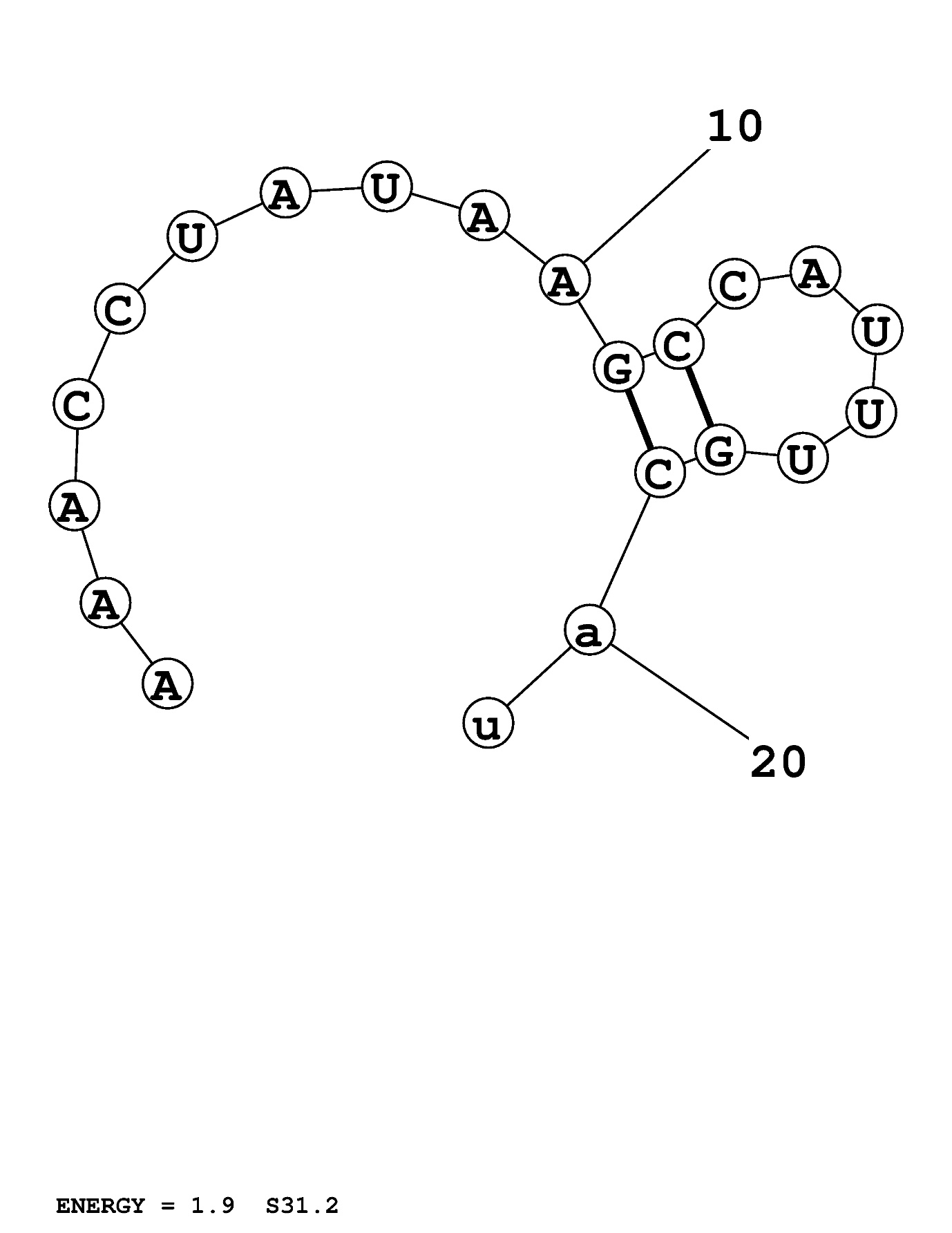


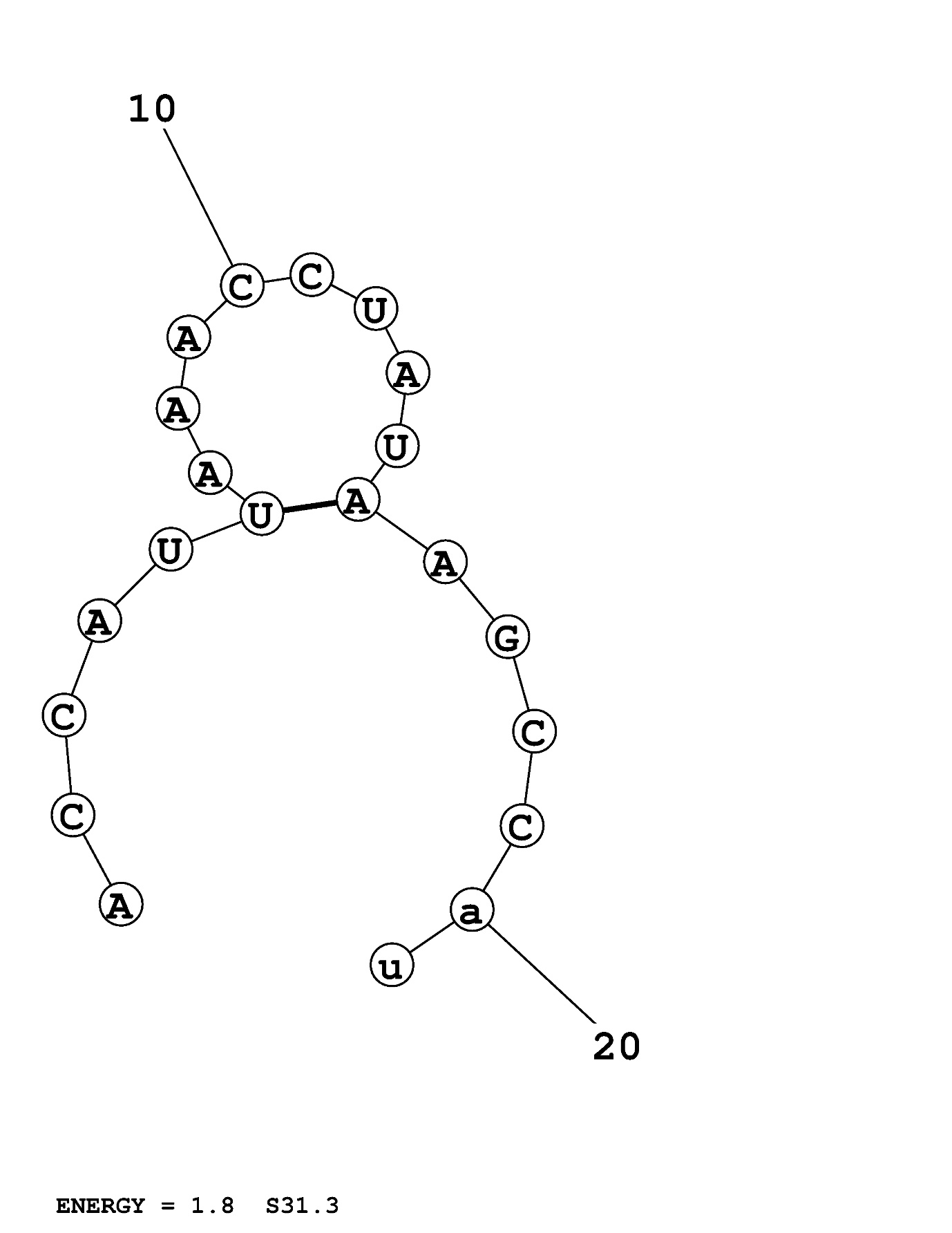


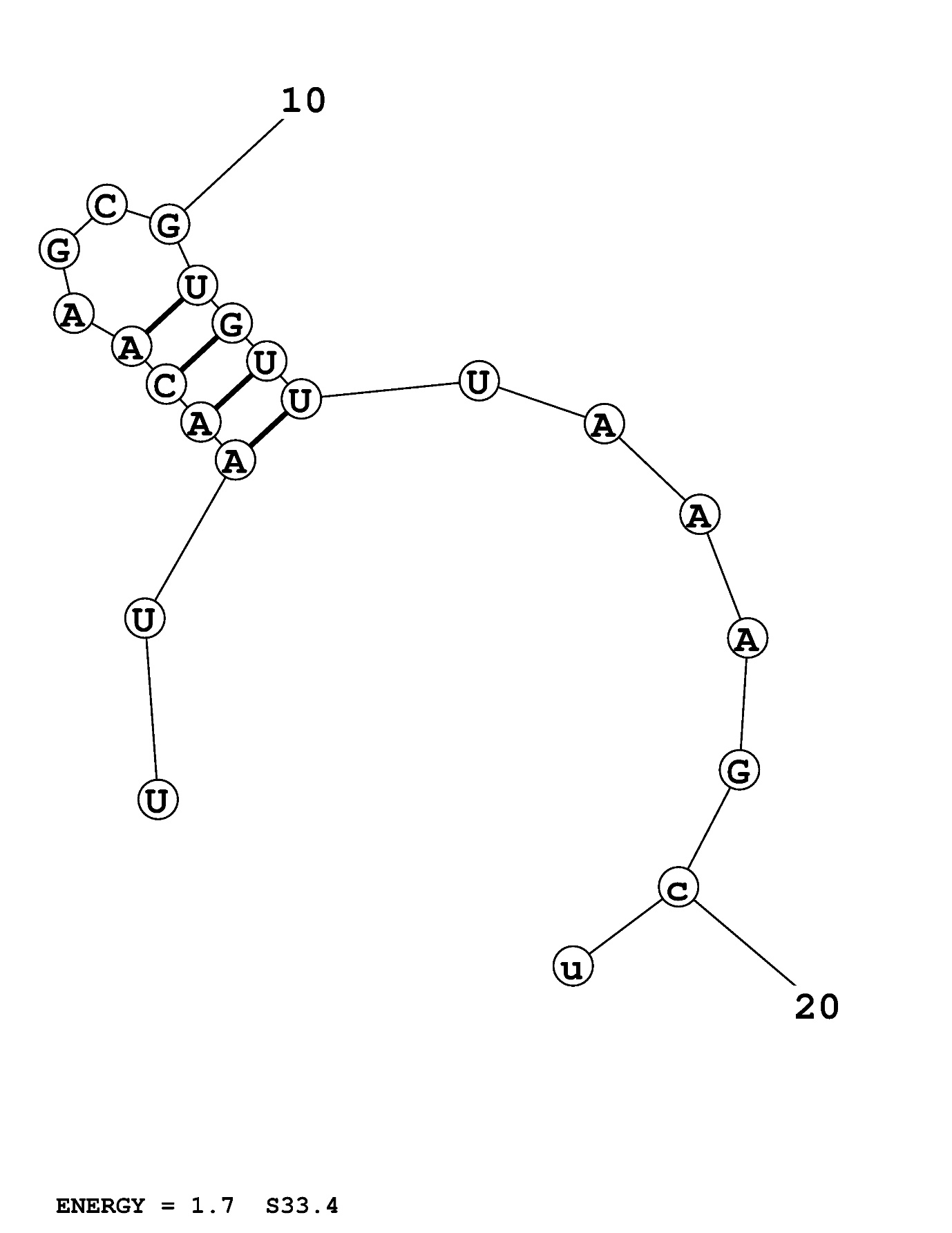


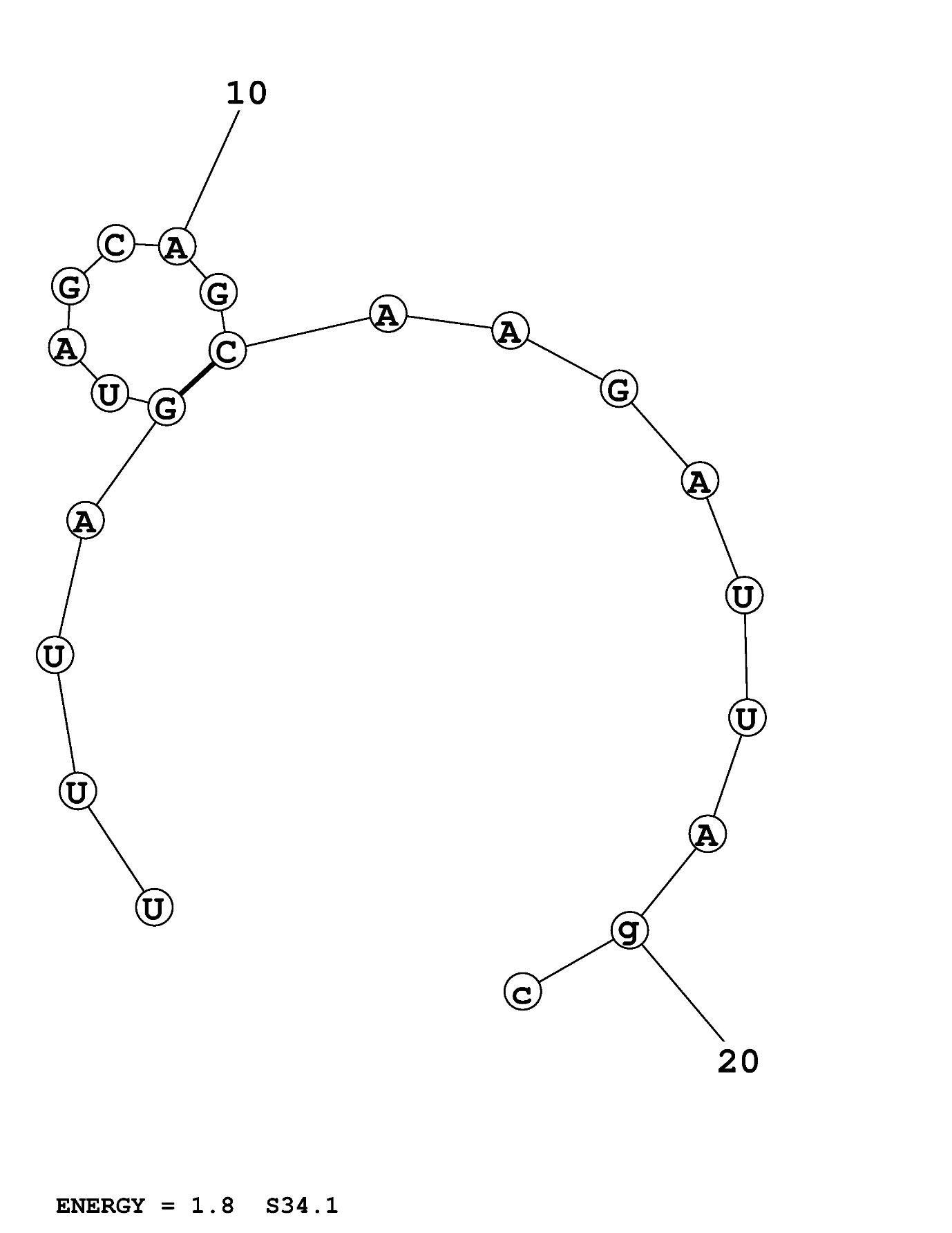


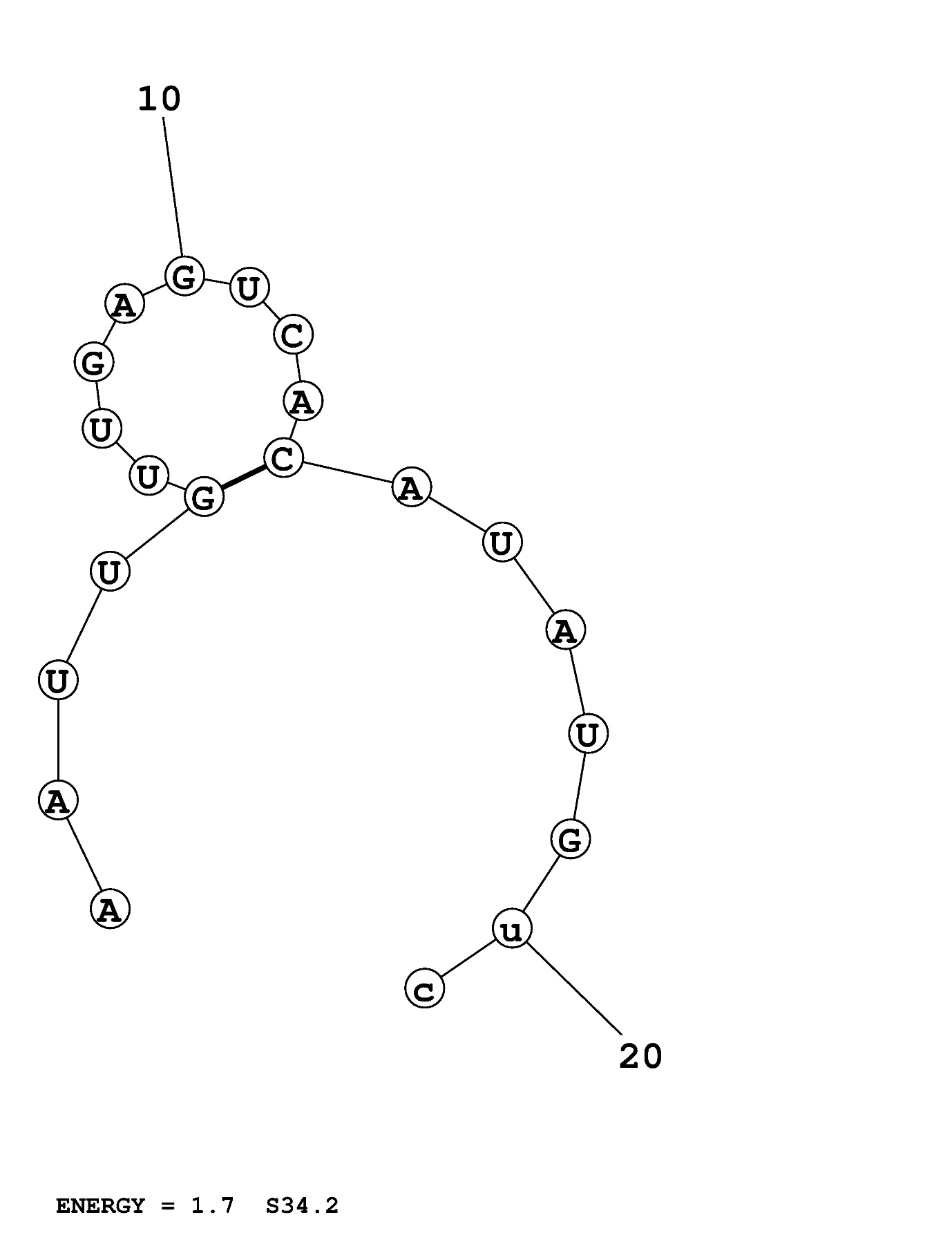


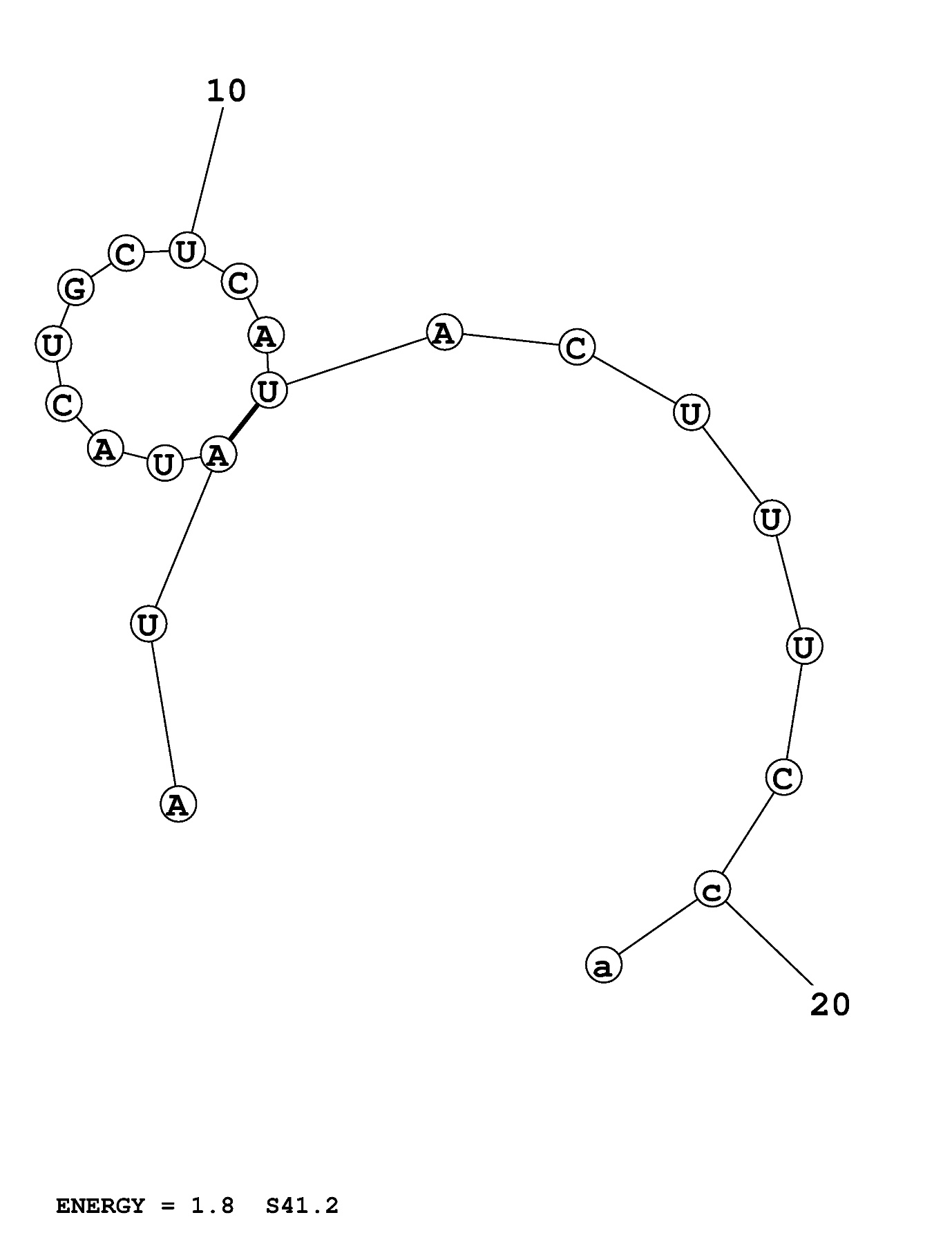

Supplement: Supplementary file 24 — Additional file 24: Supplementary Fig. S1 a–c. Structures of guide strands of siRNAs of M, N & S genes and their energy values. [file 43141_2022_346_MOESM24_ESM.docx]
