# Supplementary material for: Design of siRNA molecules for silencing of membrane glycoprotein, nucleocapsid phosphoprotein, and surface glycoprotein genes of SARS-CoV2
Source: J Genet Eng Biotechnol. 2022 Apr 28;20:65. doi: 10.1186/s43141-022-00346-z (PMC9047631; doi:10.1186/s43141-022-00346-z)

**Supplementary Figures S2a-S2c**

**Lowest free energy structures of guide strands of siRNAs of M, N & S genes and their corresponding target regions and their energy values**

*******

**Supplementary Figures S2a: Lowest free energy structures of guide strands of siRNAs of M gene and their corresponding target regions and their energy values**


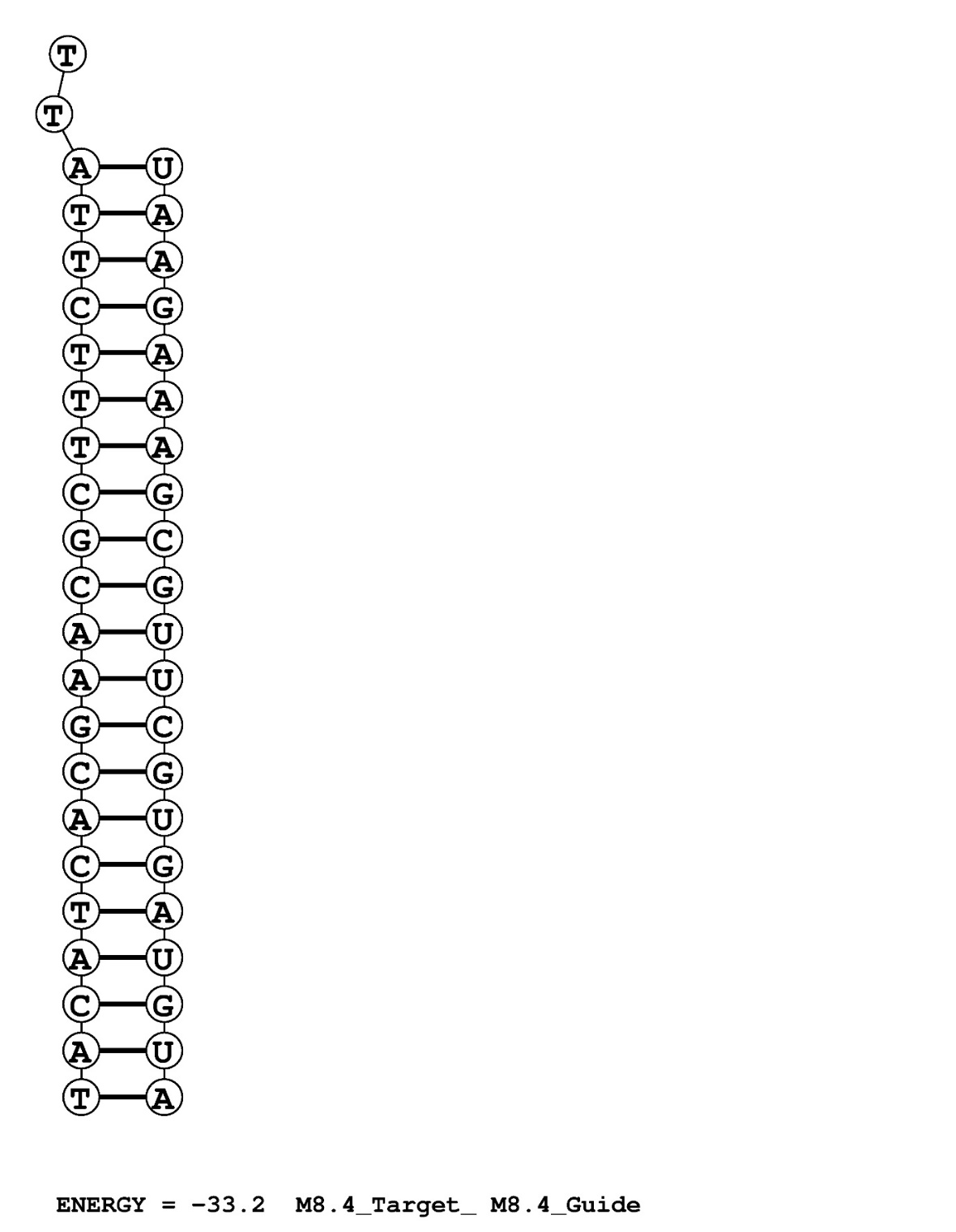


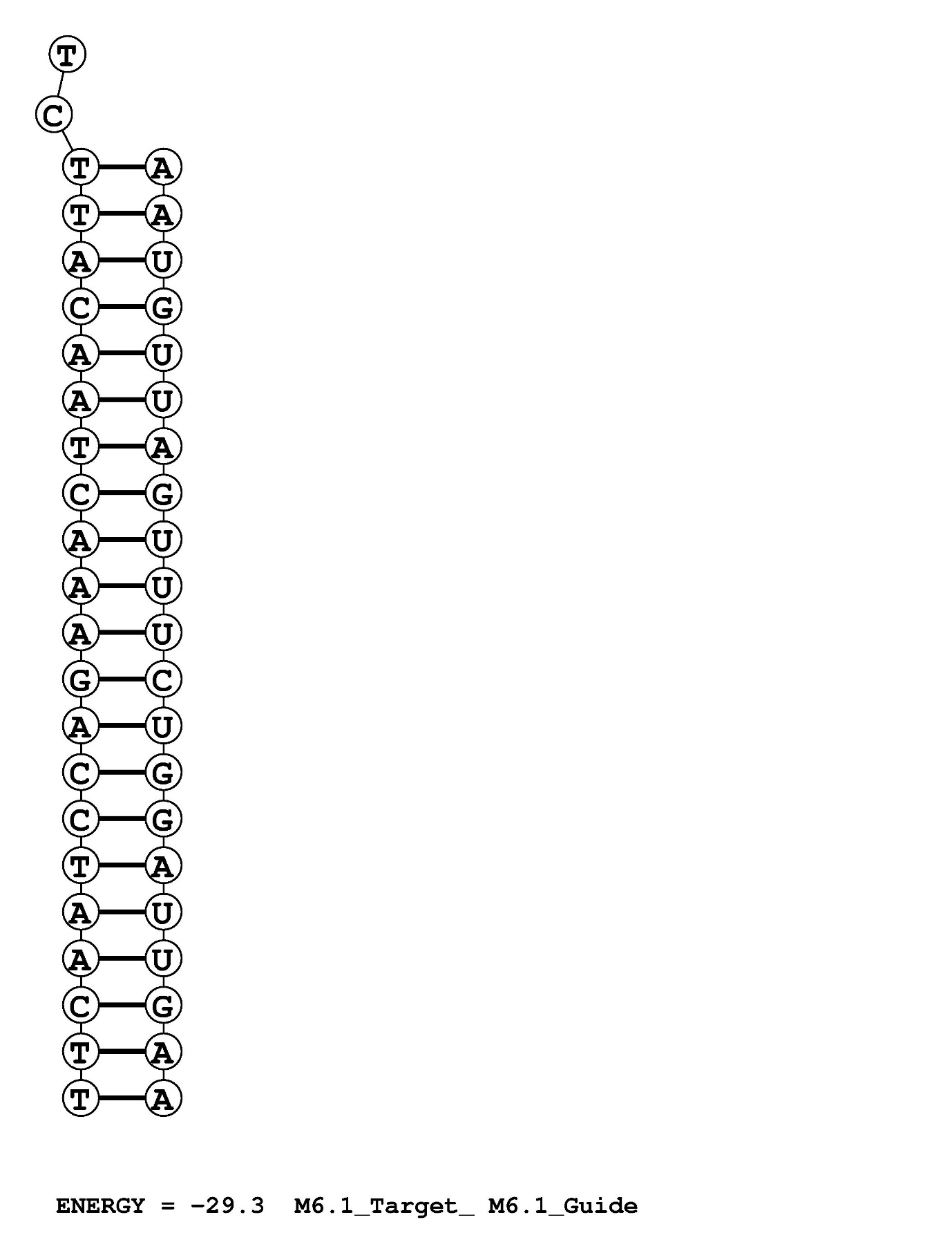


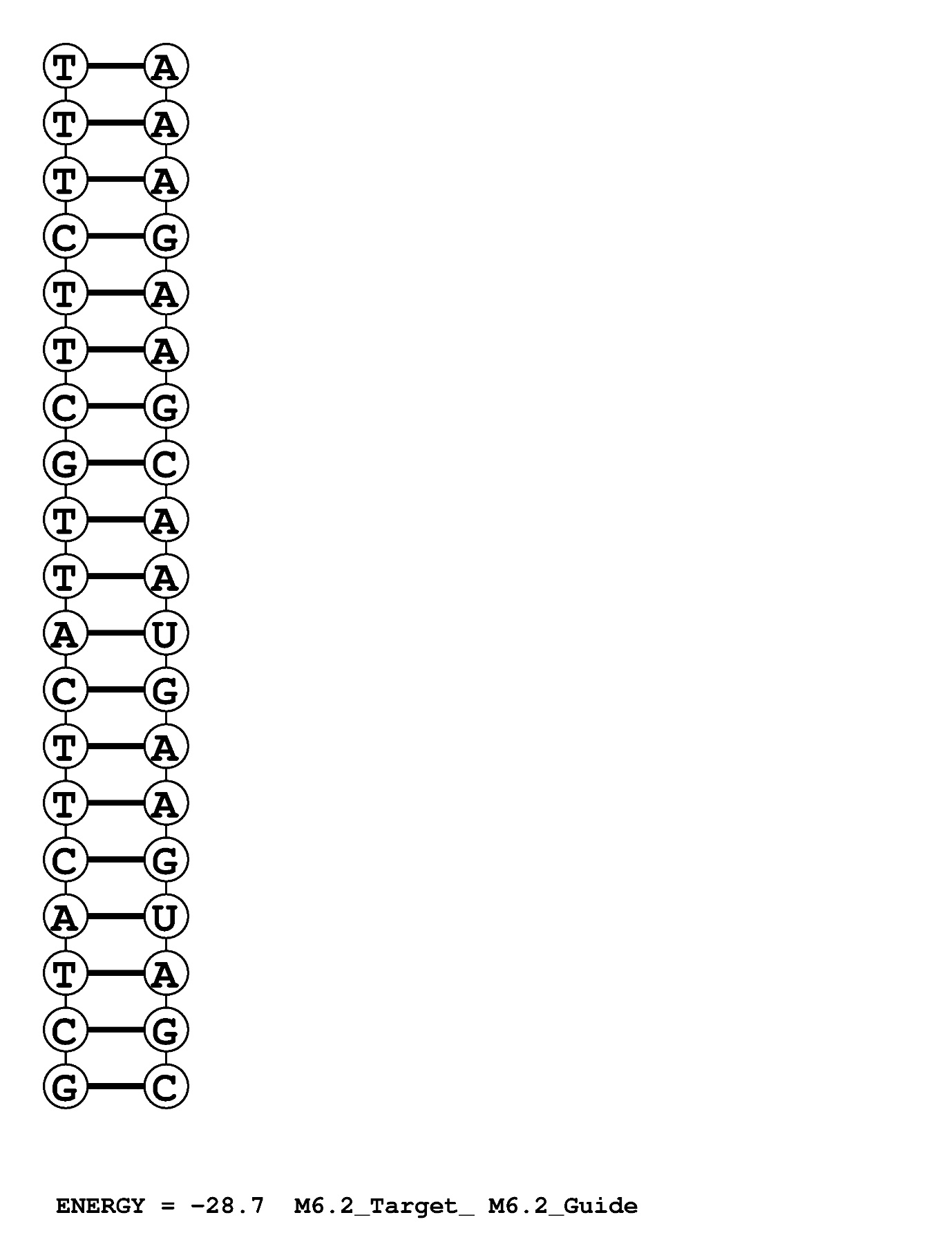


**Supplementary Figures S2b: Lowest free energy structures of guide strands of siRNAs of N gene and their corresponding target regions and their energy values**


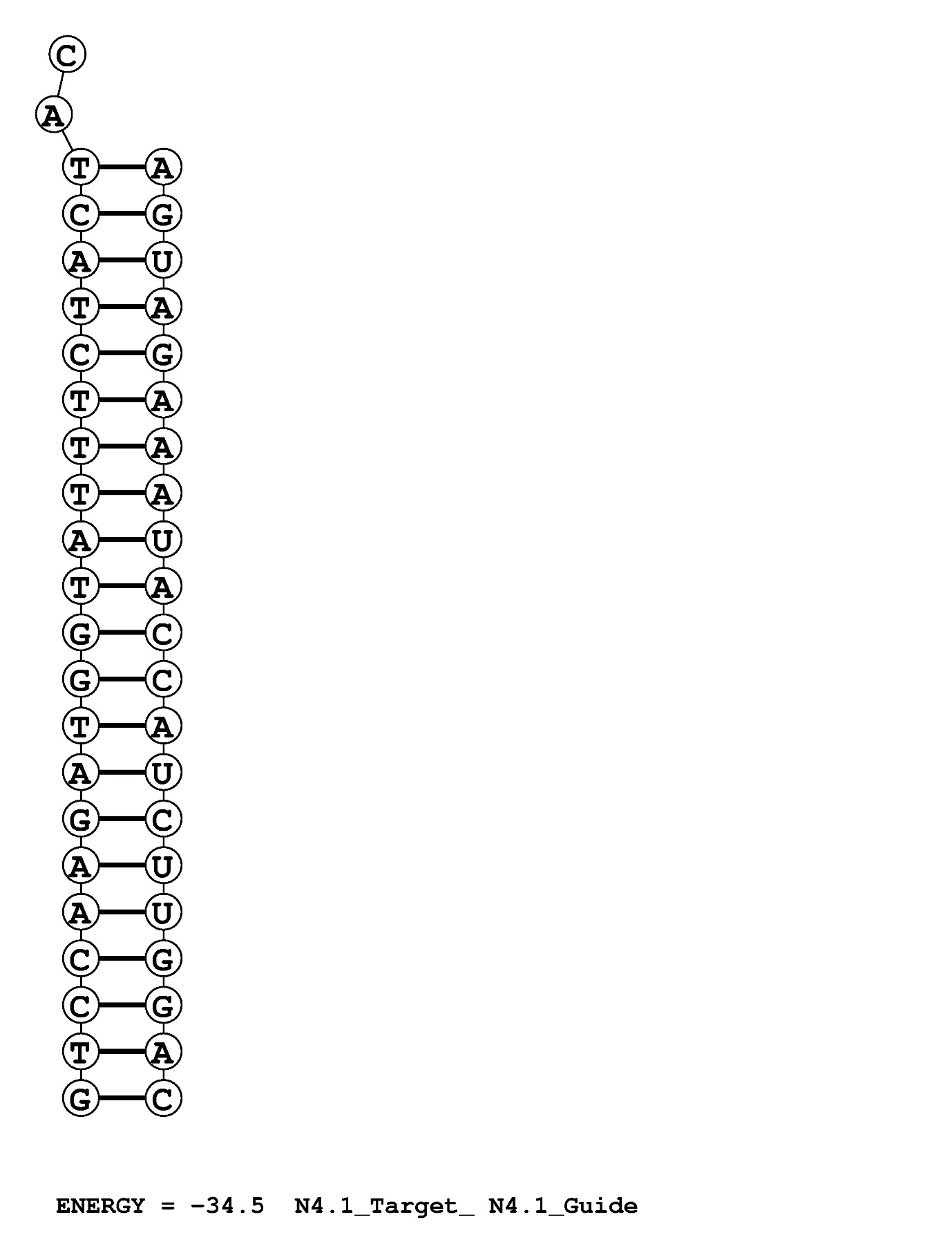


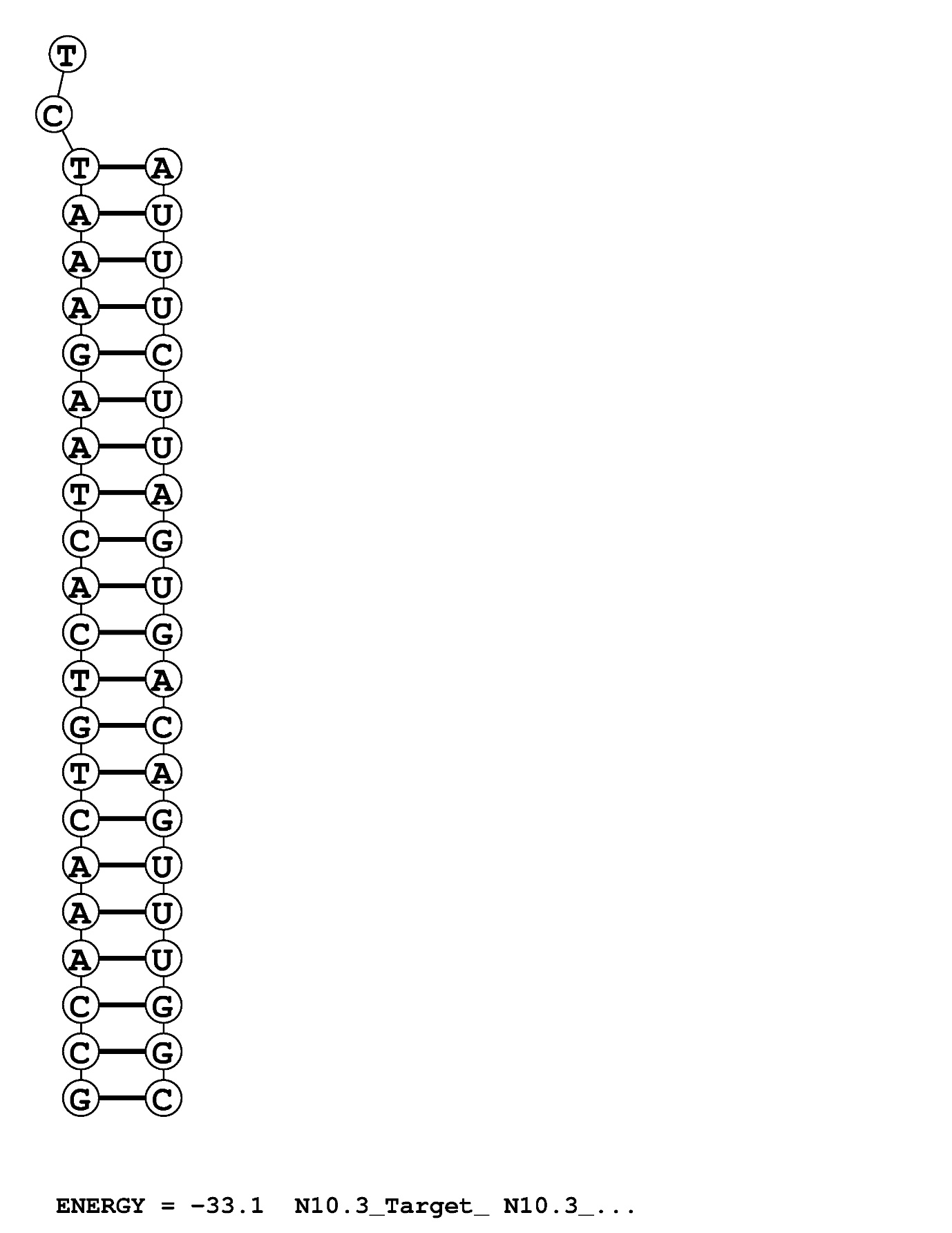


**Supplementary Figures S2c: Lowest free energy structures of guide strands of siRNAs of S gene and their corresponding target regions and their energy values**


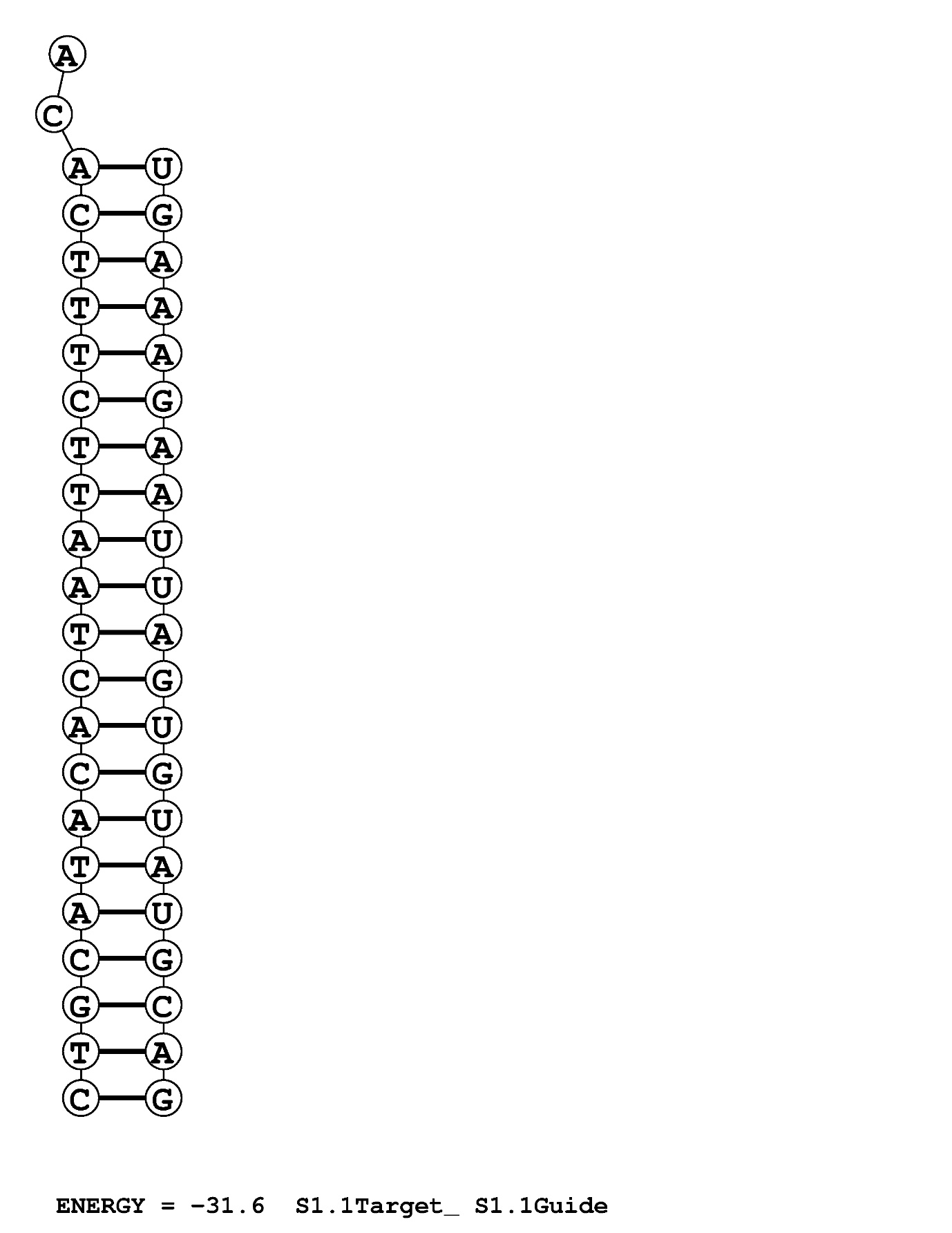


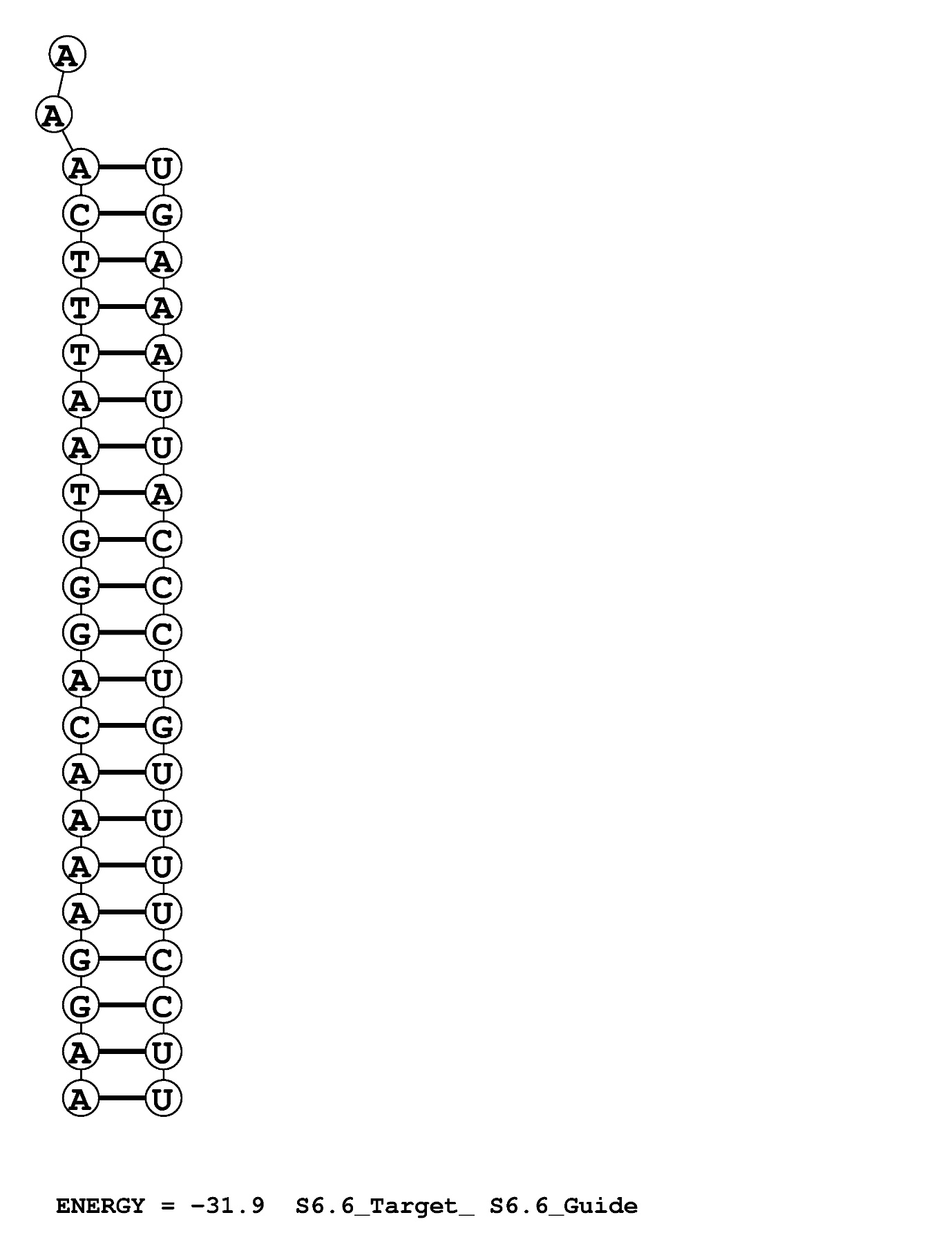


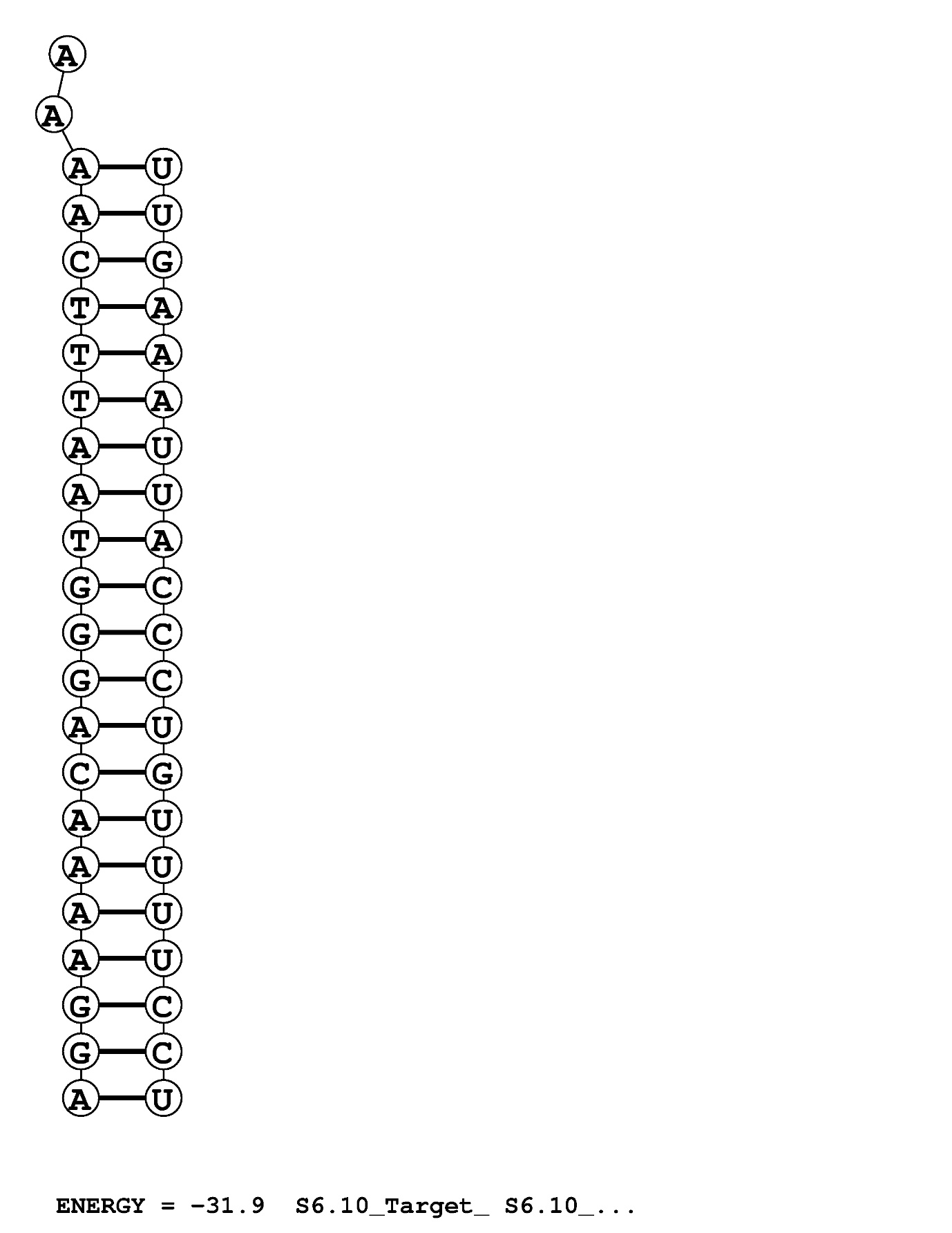


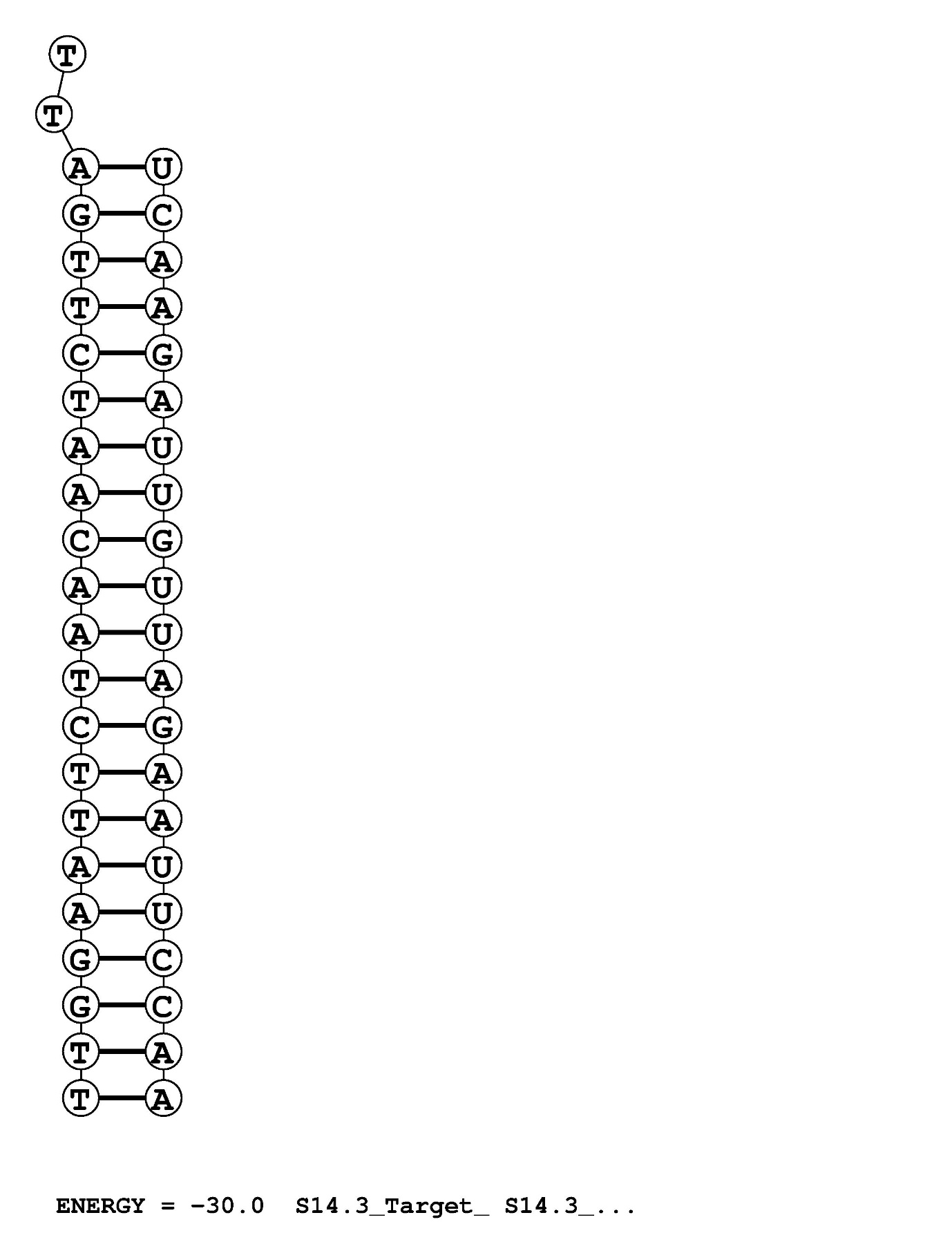


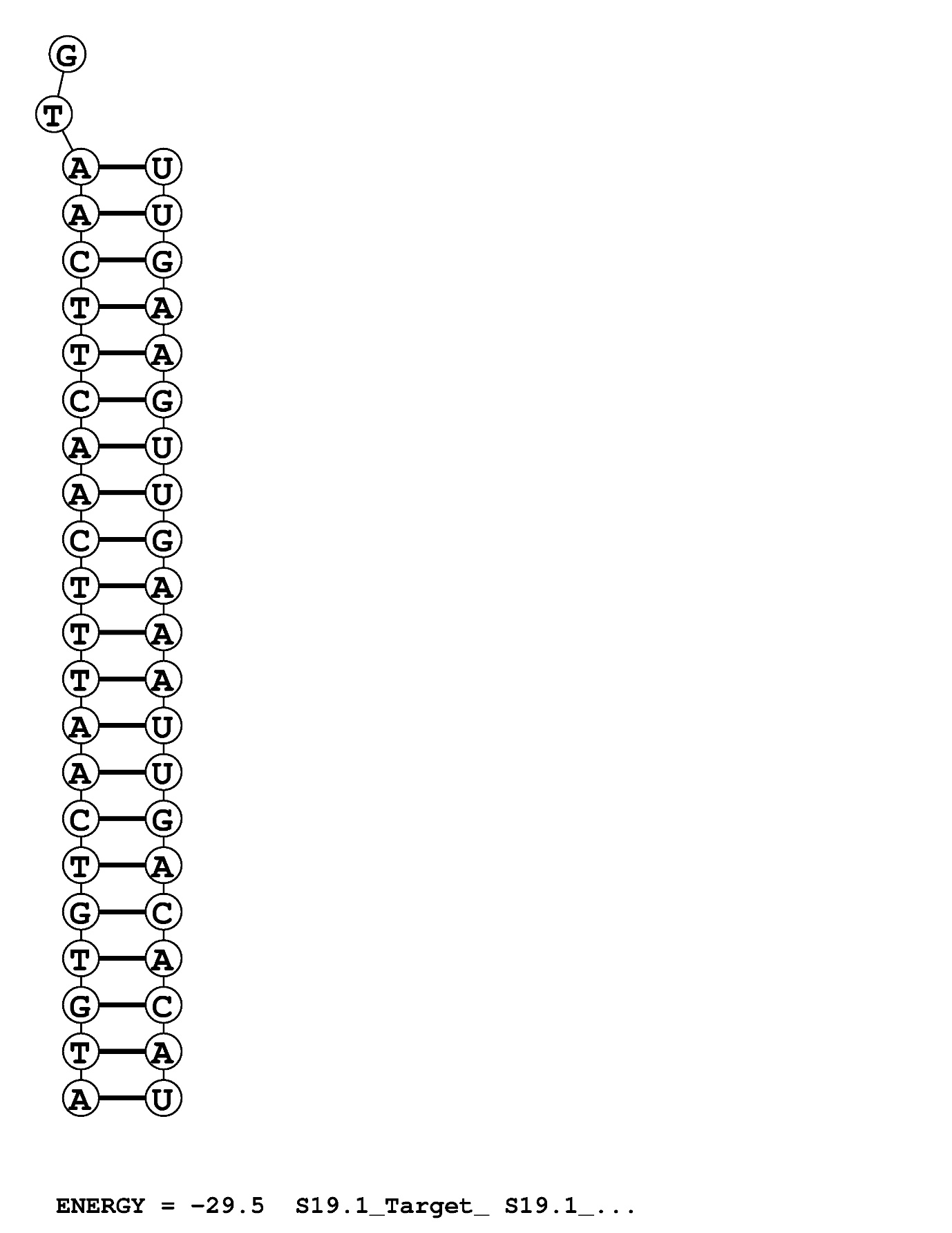


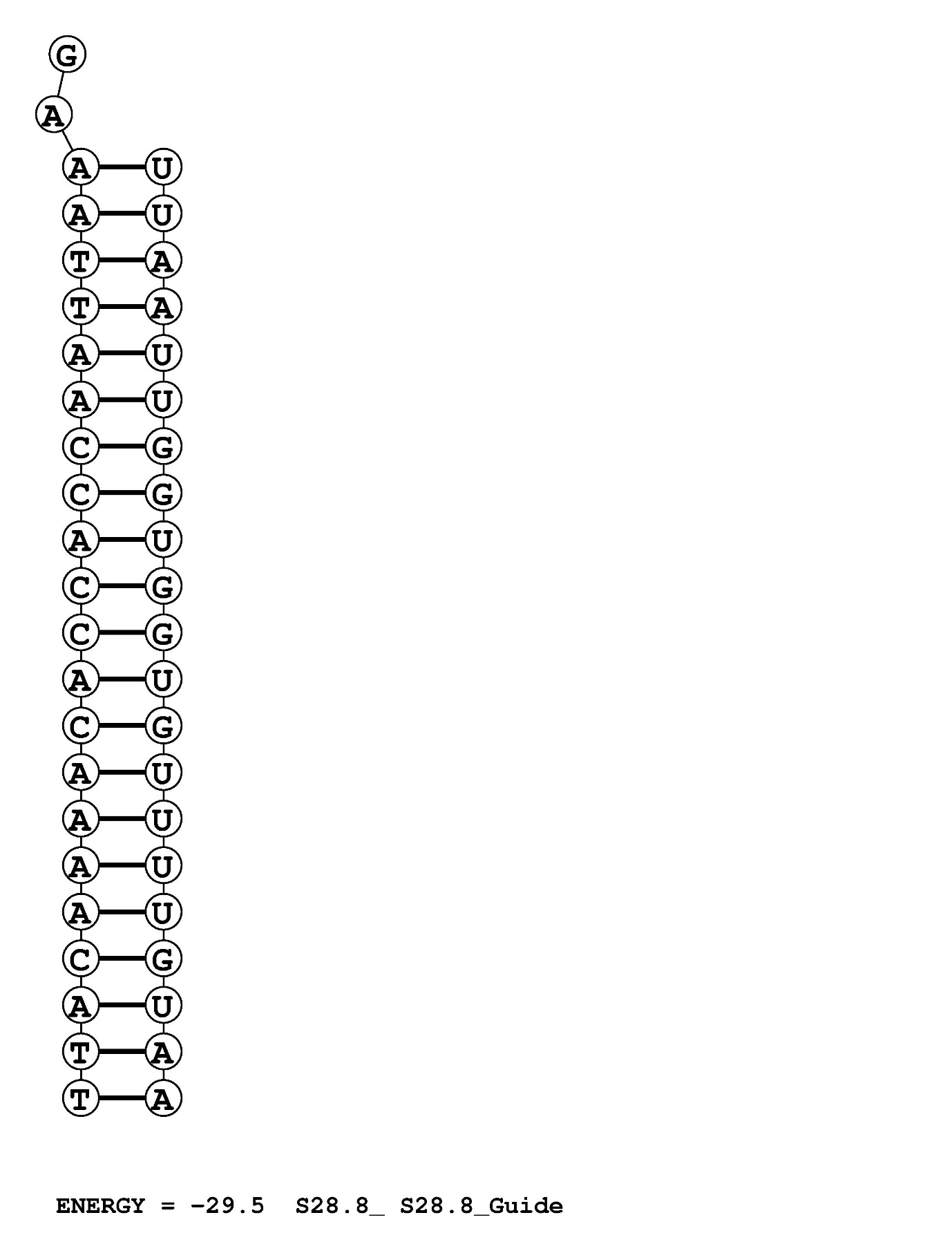


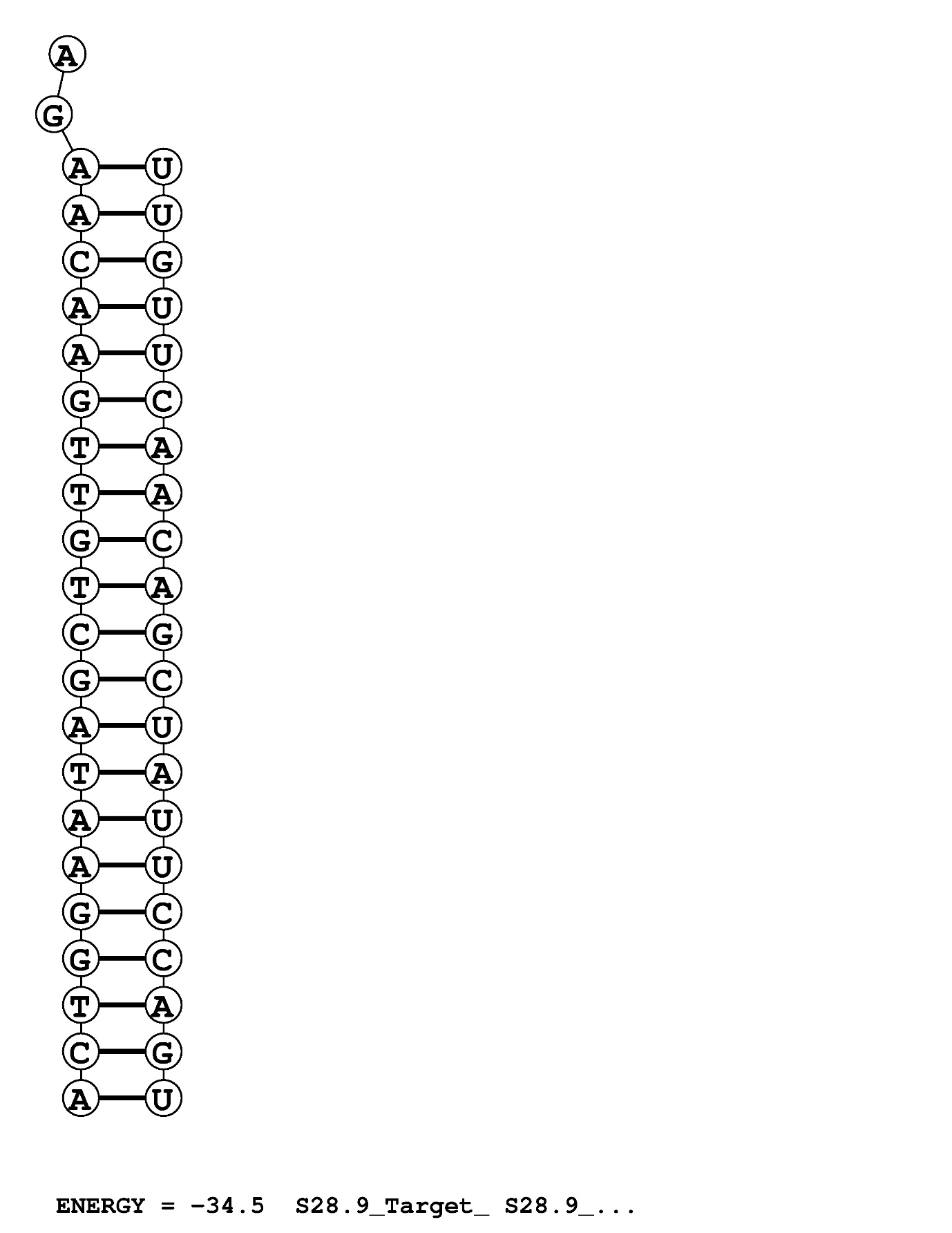


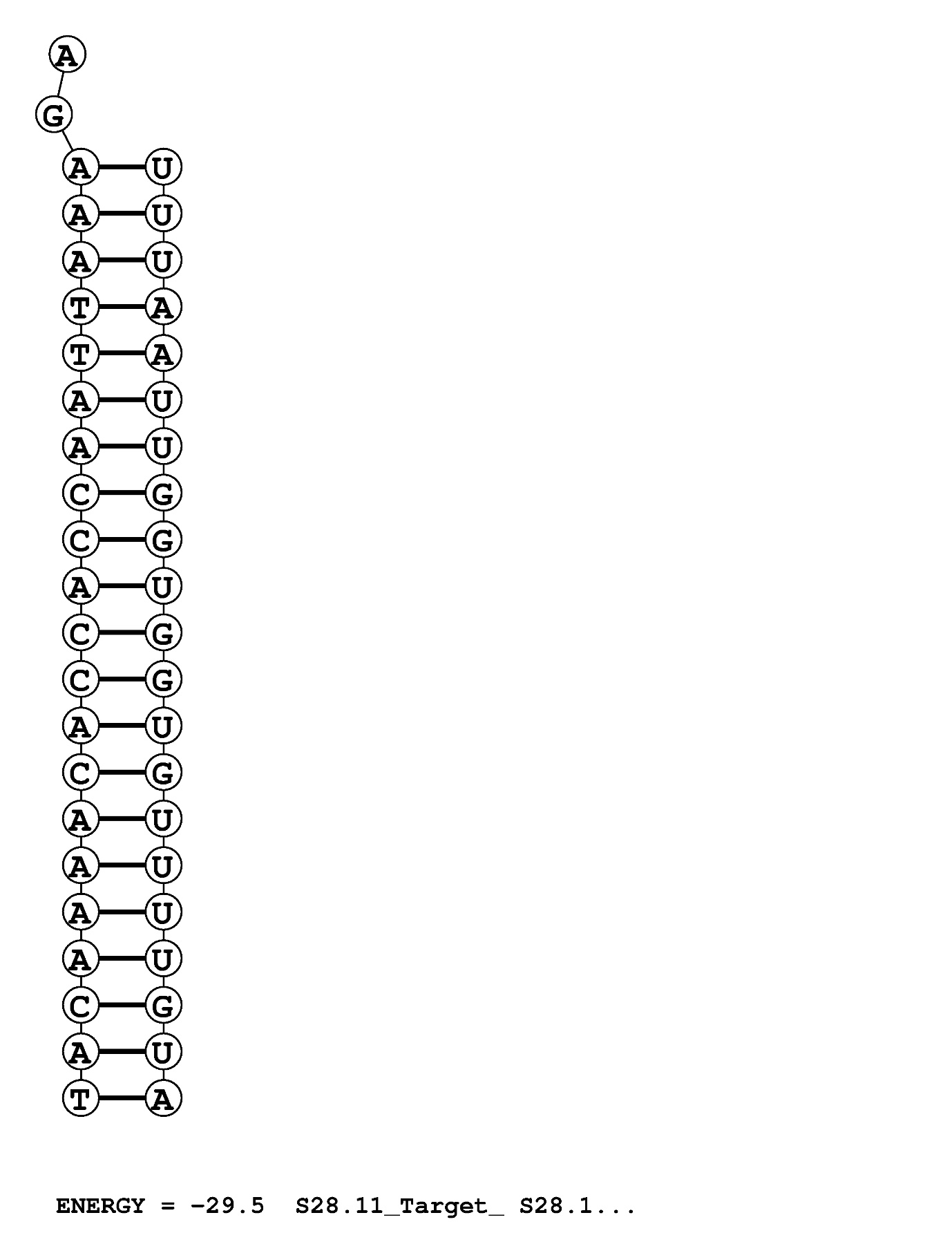


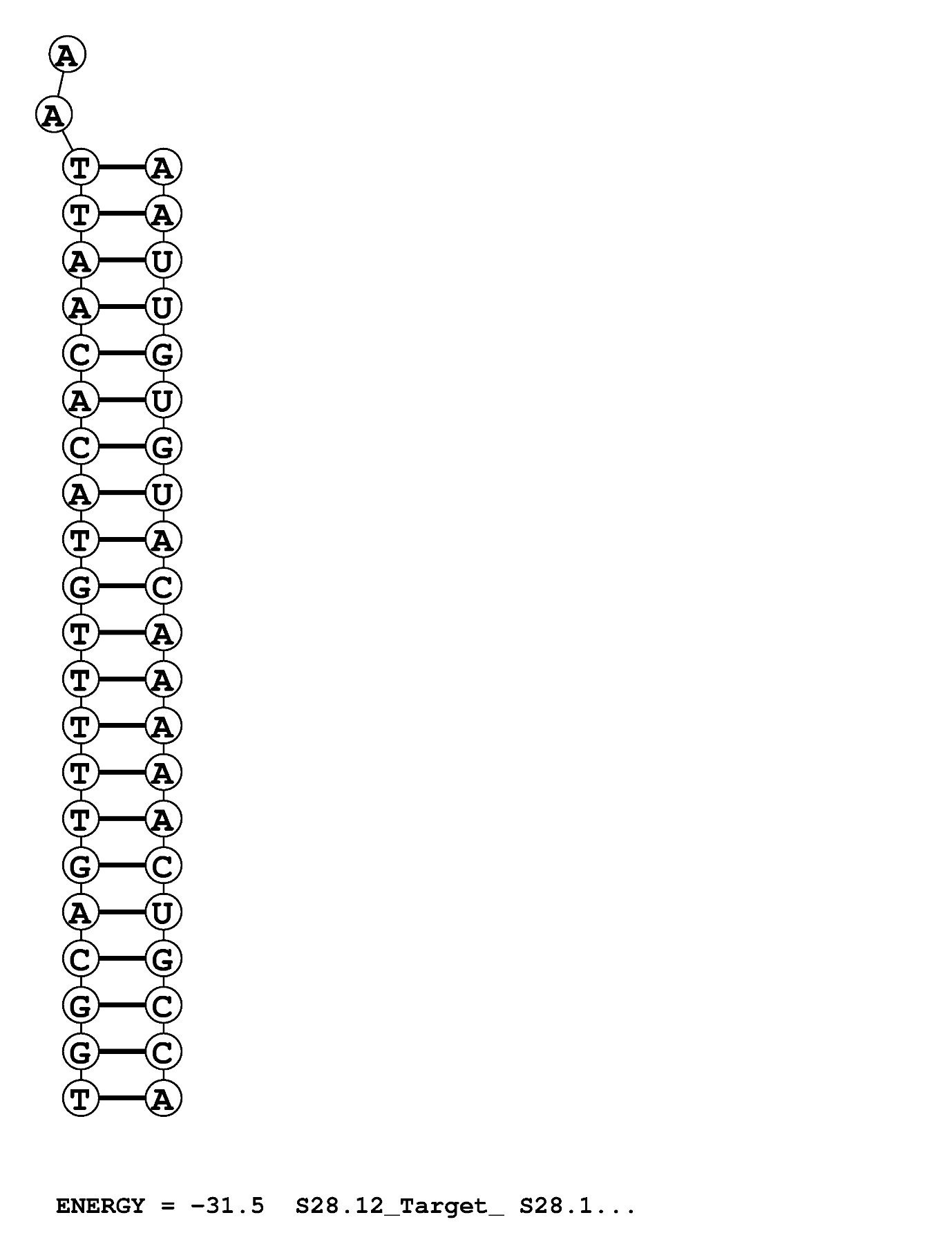


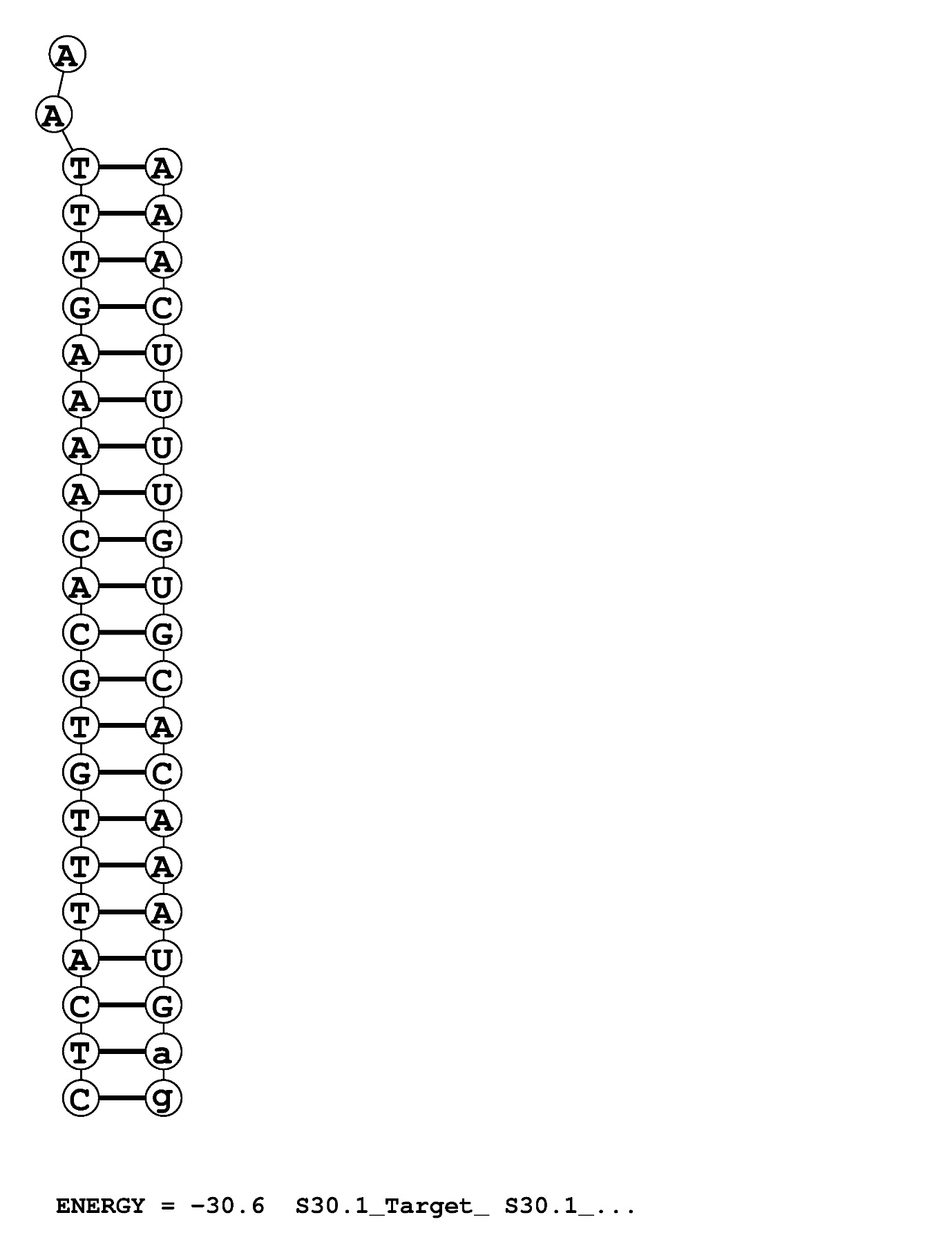


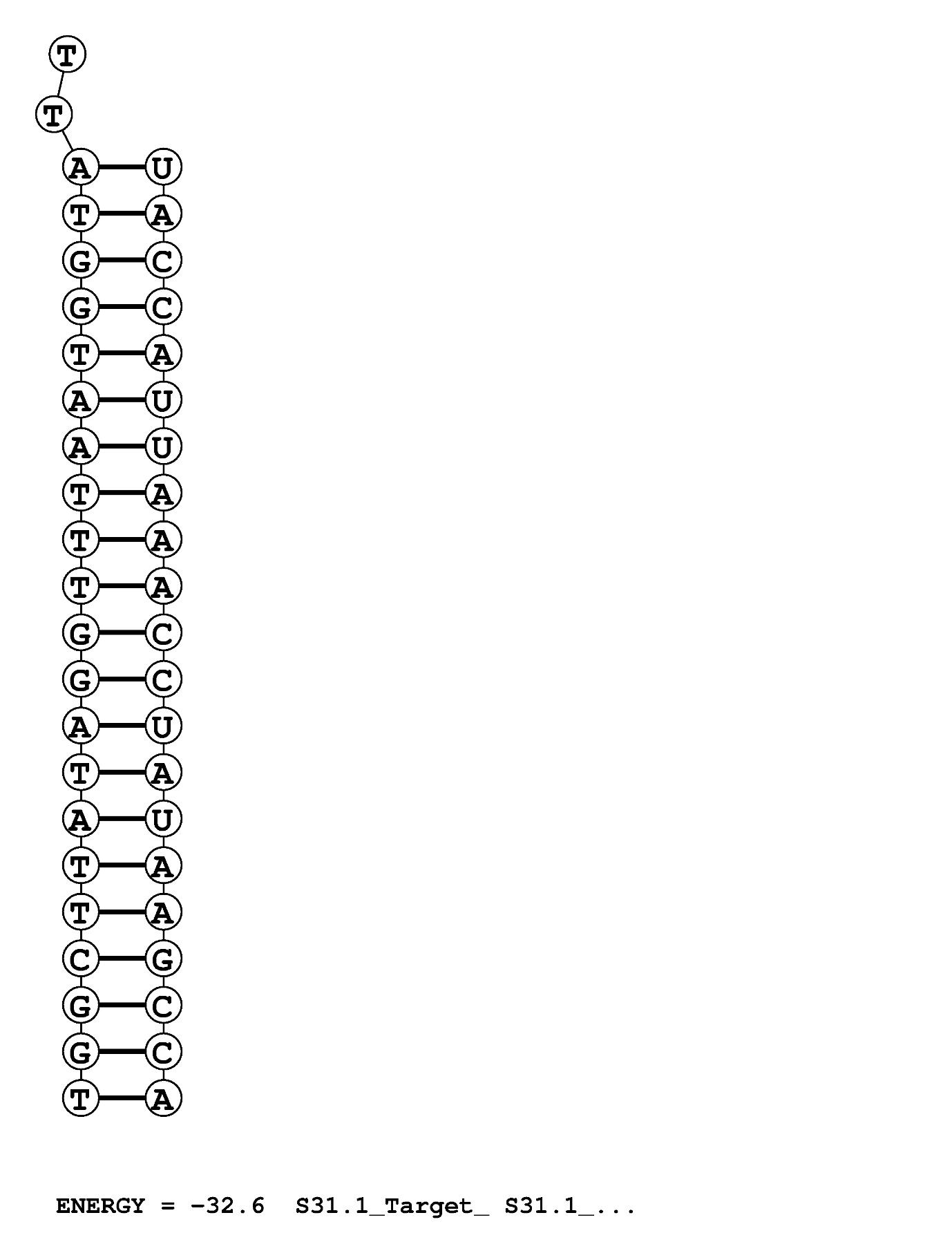


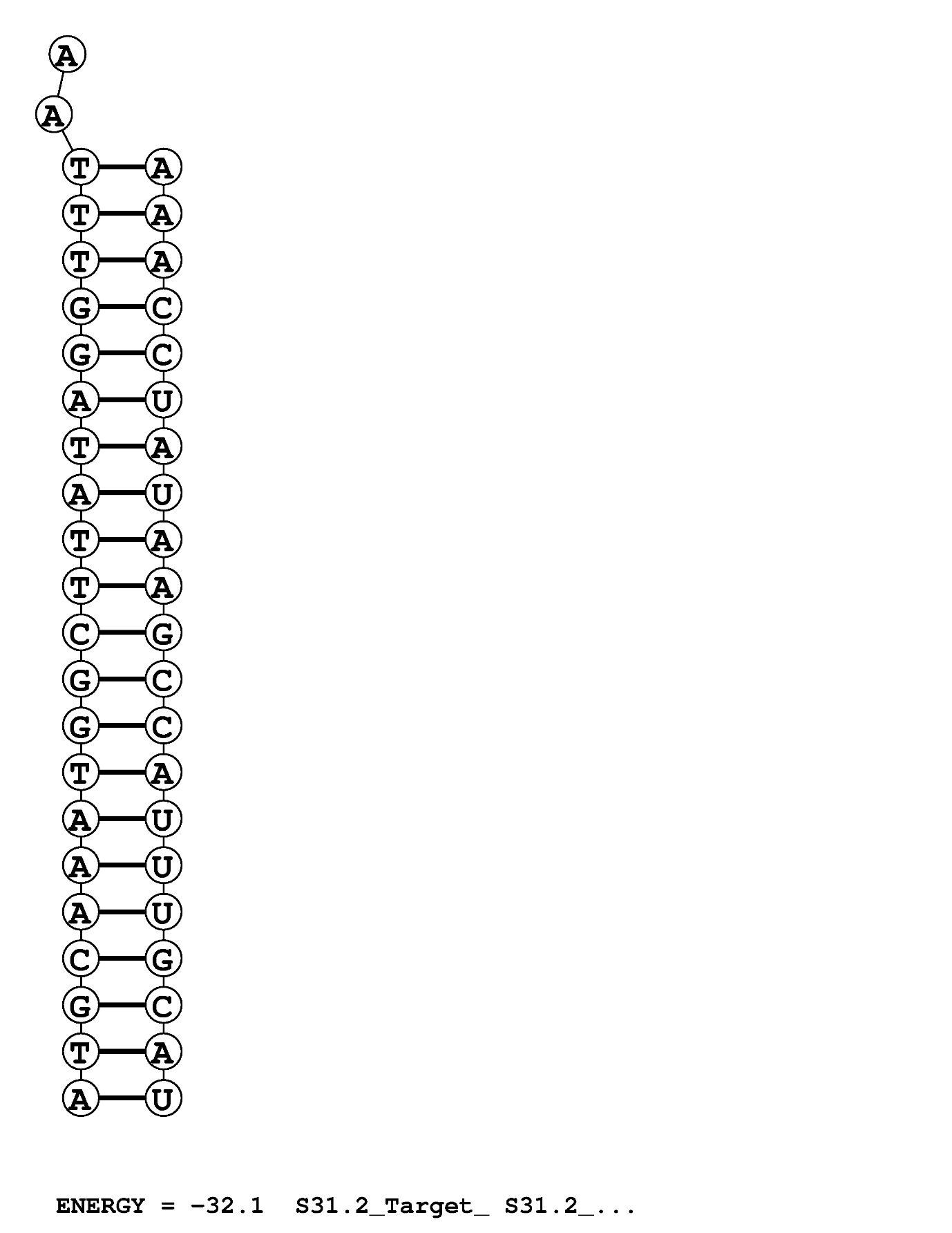


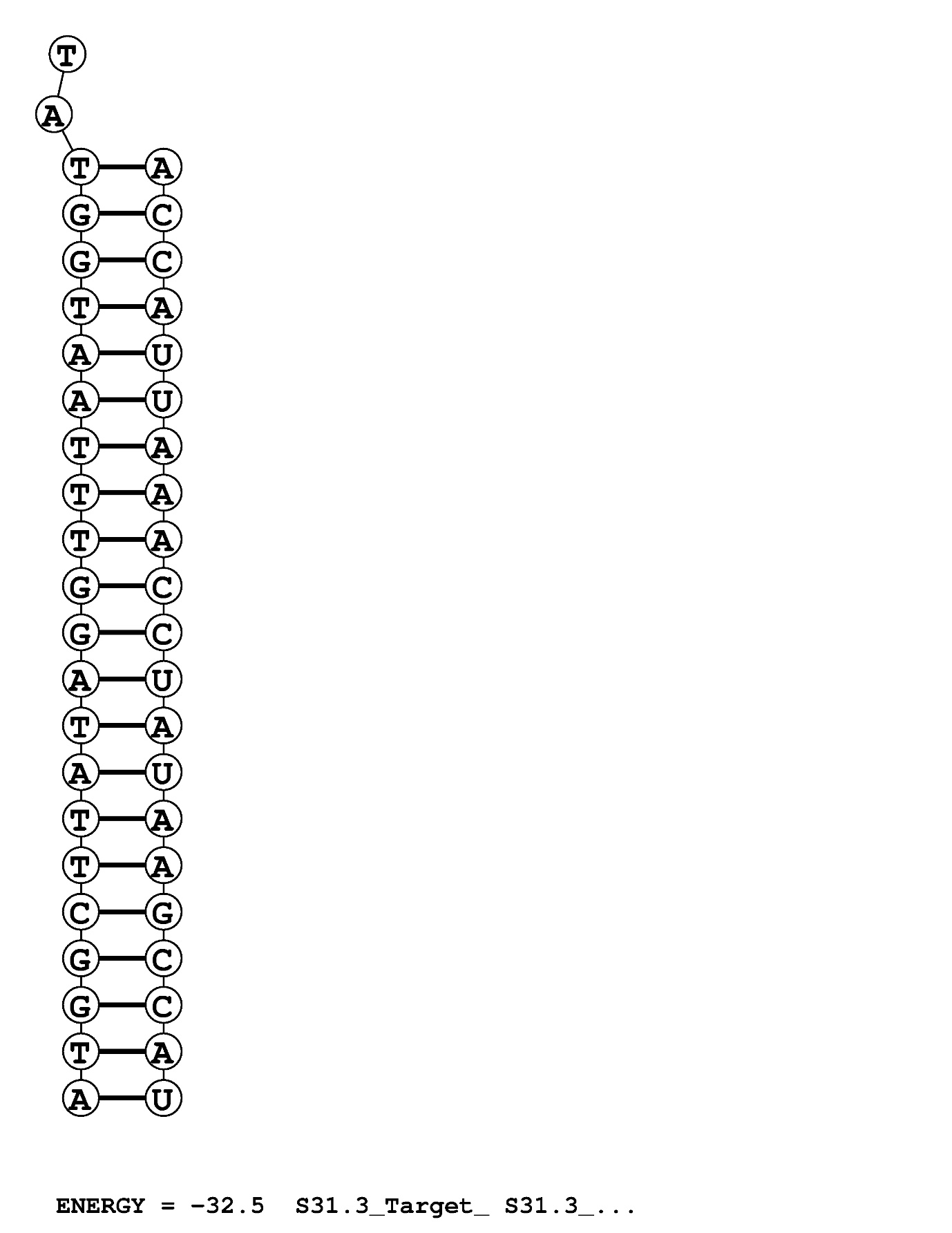


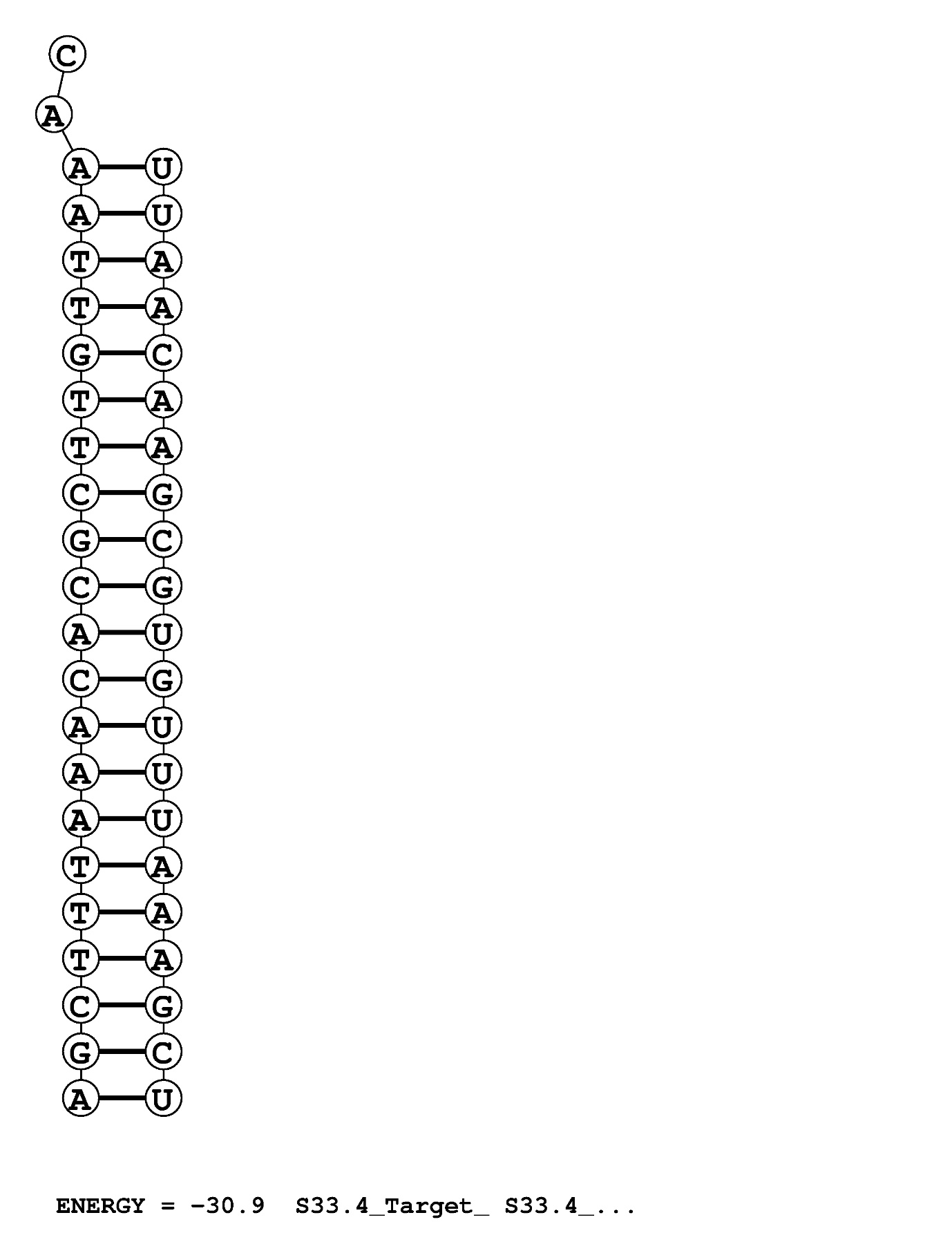


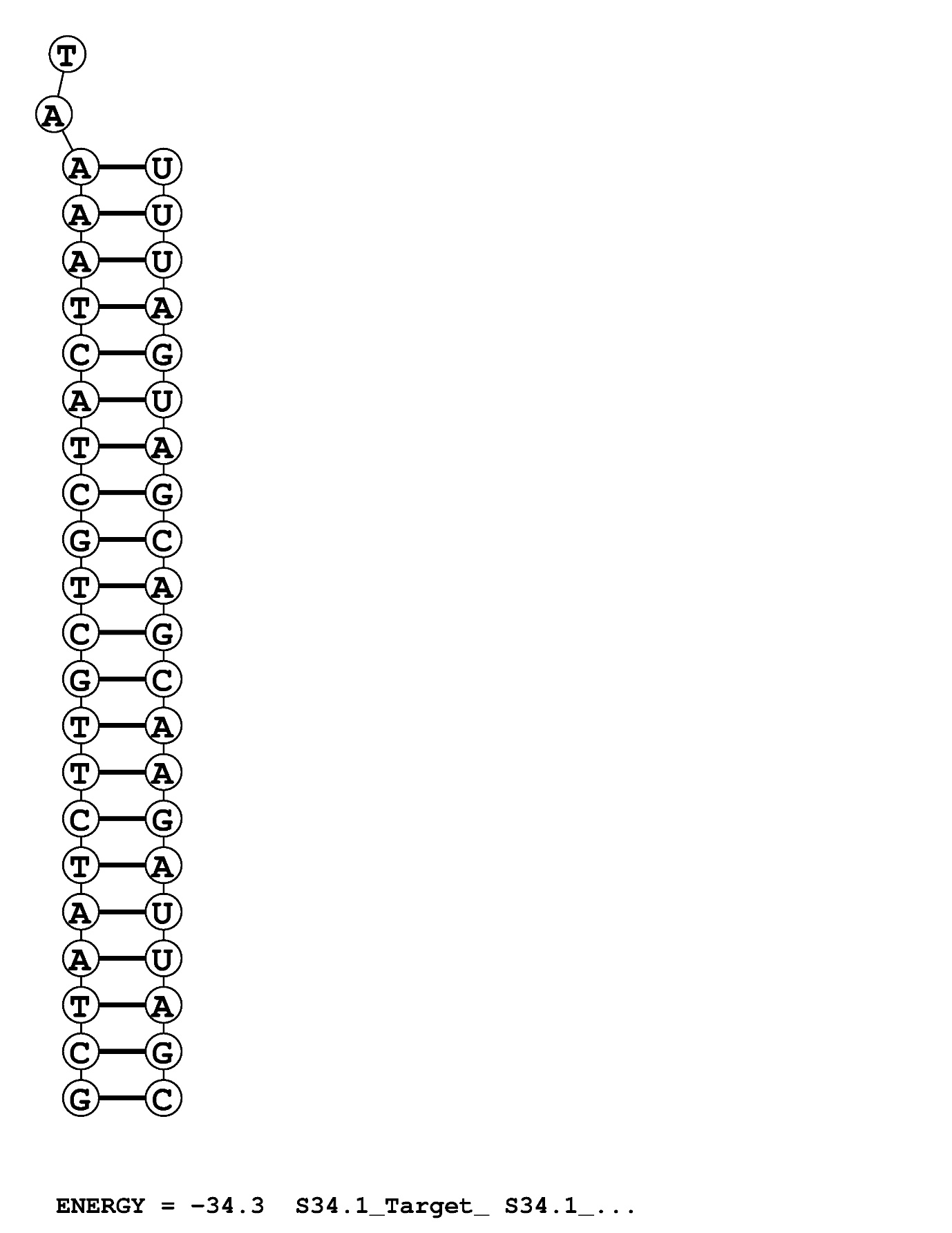


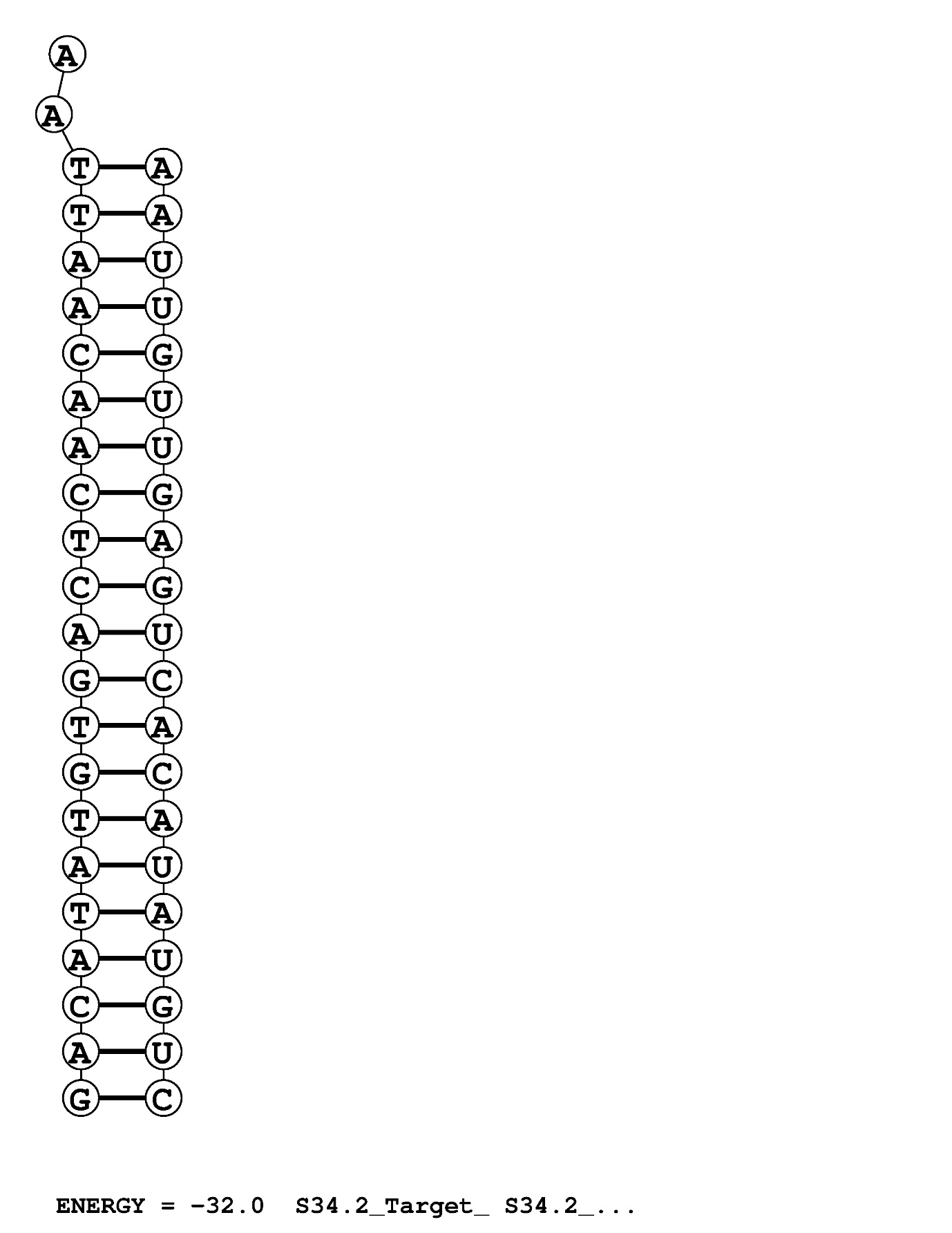


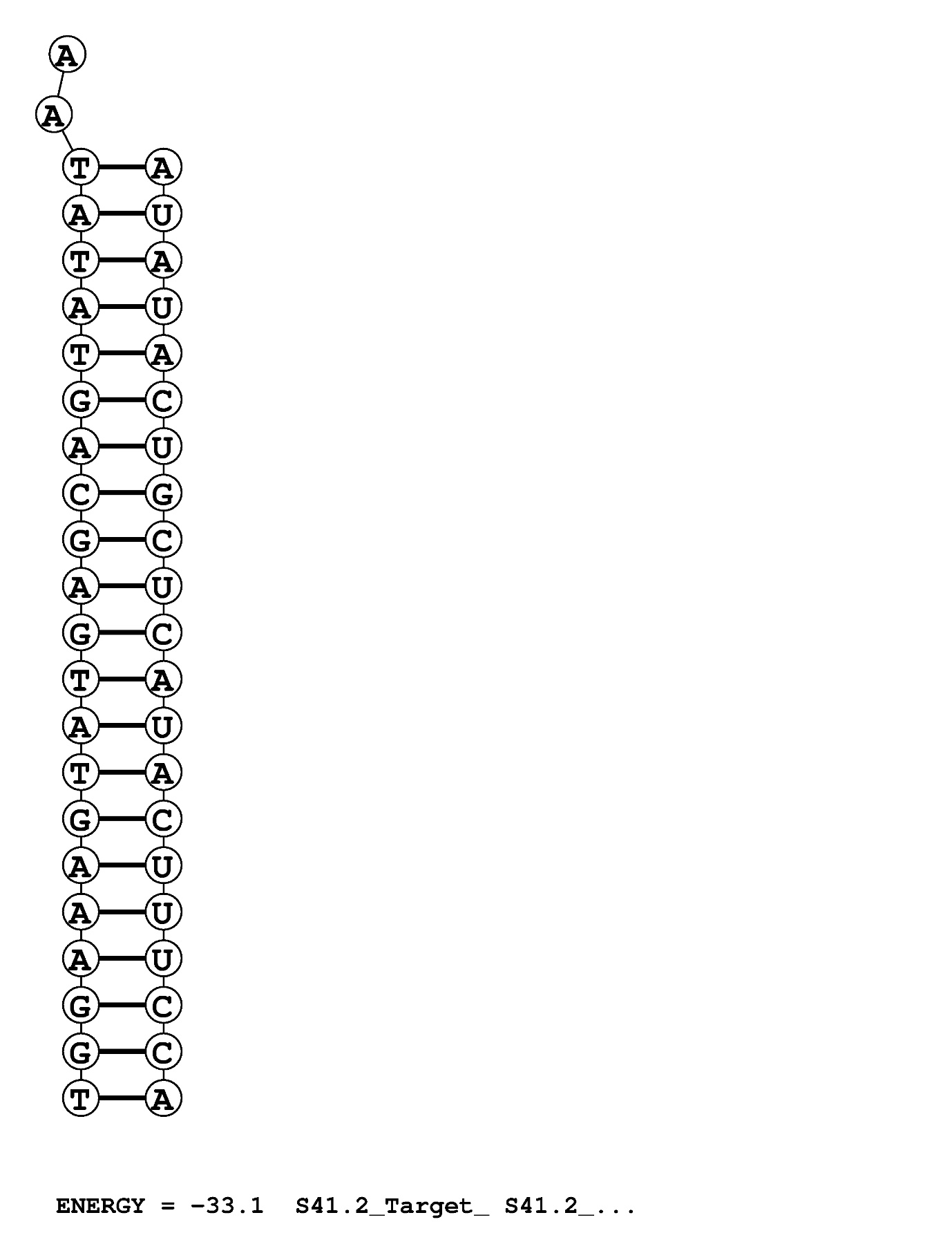

Supplement: Supplementary file 25 — Additional file 25: Supplementary Fig. S2 a–c. Lowest free energy structures of guide strands of siRNAs of M, N & S genes and their corresponding target regions and their energy values. [file 43141_2022_346_MOESM25_ESM.docx]
